# Sympatric *Pieris* butterfly species exhibit a high conservation of chemoreceptors

**DOI:** 10.3389/fncel.2023.1155405

**Published:** 2023-05-11

**Authors:** Qi Wang, Marcel Dicke, Alexander Haverkamp

**Affiliations:** Laboratory of Entomology, Wageningen University and Research, Wageningen, Netherlands

**Keywords:** *Pieris*, sympatric speciation, sensory drive, chemoreceptors, genome and transcriptome, lepidoptera

## Abstract

Sensory processes have often been argued to play a central role in the selection of ecological niches and in the formation of new species. Butterflies are among the best studied animal groups with regards to their evolutionary and behavioral ecology and thereby offer an attractive system to investigate the role of chemosensory genes in sympatric speciation. We focus on two *Pieris* butterflies with overlapping host-plant ranges: *P. brassicae* and *P. rapae*. Host-plant choice in lepidopterans is largely based on their olfactory and gustatory senses. Although the chemosensory responses of the two species have been well characterized at the behavioral and physiological levels, little is known about their chemoreceptor genes. Here, we compared the chemosensory genes of *P. brassicae* and *P. rapae* to investigate whether differences in these genes might have contributed to their evolutionary separation. We identified a total of 130 and 122 chemoreceptor genes in the *P. brassicae* genome and antennal transcriptome, respectively. Similarly, 133 and 124 chemoreceptors were identified in the *P. rapae* genome and antennal transcriptome. We found some chemoreceptors being differentially expressed in the antennal transcriptomes of the two species. The motifs and gene structures of chemoreceptors were compared between the two species. We show that paralogs share conserved motifs and orthologs have similar gene structures. Our study therefore found surprisingly few differences in the numbers, sequence identities and gene structures between the two species, indicating that the ecological differences between these two butterflies might be more related to a quantitative shift in the expression of orthologous genes than to the evolution of novel receptors as has been found in other insects. Our molecular data supplement the wealth of behavioral and ecological studies on these two species and will thereby help to better understand the role of chemoreceptor genes in the evolution of lepidopterans.

## 1. Introduction

Sympatric speciation has been of major interest in evolutionary biology as it allows to investigate selective pressures in the absence of geographical constraints (Fitzpatrick et al., 2008; Foote, 2018). Butterflies and moths have a long history as model species, both for ecology-driven speciation as well as for speciation through sexual selection (Kawahara et al., 2019; De Pasqual et al., 2021).

Changes in the chemosensory system have often been argued to be major drivers of speciation of plant-feeding insects as these heavily rely on chemical cues for selecting an optimal host-plant and for finding mates (Knolhoff and Heckel, 2014; Khallaf and Knaden, 2022). In the genus *Drosophila*, several cases of host specialization have occurred, among which the adaption of *D. sechellia* to the toxic noni fruit has been analyzed most thoroughly. This host switch from a broad range of decaying fruits to a single normally toxic plant was accompanied by a loss of one specific chemoreceptor, point mutations in another receptor as well as changes in the expression levels of several receptor genes (Auer et al., 2020). Similarly, the fly *Scaptomyza flava*, which is a close relative to *Drosophila* within the family Drosophilidae, has recently evolved an herbivorous life style. This switch from microbe-feeding to a plant-based diet was accompanied by a loss or pseudogenization of several olfactory genes related to the detection of microbial volatiles and a gene duplication event for another receptor involved in the detection of plant volatiles (Goldman-Huertas et al., 2015). In lepidopterans, chemoreceptor genes have been extensively analyzed in different moths’ species. The two closely related species *Helicoverpa armigera* and *H. assulta*, for example, have strongly diverged in their host-plant range. While *H. armigera* is a worldwide pest with over 200 known host-plants, *H. assulta* is restricted to plants of the Solanaceous family. Interestingly, these two species also differ in their set of expressed antennal chemoreceptors with several unique genes in both species (Zhang et al., 2015). Taken together, these examples across different insect orders indicate that both rapid gene loss, as well as gene duplication events accompanied by changes in chemoreceptor expression levels are common hallmarks of host-plant shifts and other ecological adaptions in insects.

Therefore, the comparison of chemoreceptors occurring in closely related species can reveal important information on the differences between these species with regards to their host-plant selection or sexual communication. Chemoreceptors in insects can be classified into three classes: odorant receptors (ORs), ionotropic receptors (IRs), and gustatory receptors (GRs). Although a large number of studies have identified many chemoreceptors in different species of Diptera, Lepidoptera, Hemiptera, and Hymenoptera, the specific function of these receptors often remains unknown (Howlett et al., 2012; Zhou et al., 2012; Engsontia et al., 2014; Koenig et al., 2015; Wang et al., 2015; Zhao et al., 2016, 2020).

Before the large-scale application of genome sequencing in insects, transcriptome analysis helped tremendously in identifying chemoreceptors in different insect species (Zhu J. Y. et al., 2018; Wan et al., 2019). Yet, transcriptomes on their own are usually not sufficient to reveal all chemoreceptors given that some chemoreceptors have a very low abundance. Hence, the combination of genome and transcriptome data greatly benefits the identification of chemoreceptors (Montagné et al., 2015).

The cabbage white butterflies, *Pieris* spp., are important model species with regards to the co-evolutionary arms-race with their host plants, their natural enemies and their adaptations to novel environments (Edger et al., 2015; Sikkink et al., 2017; Zhu F. et al., 2018; Okamura et al., 2019). *Pieris brassicae* and *P. rapae* are very closely related species sharing comparable appearances, host plants and geographical distributions (Feltwell, 1982; Ryan et al., 2019; EPPO, 2022). Both species have characterized chemosensory systems and are able to detect similar volatiles and tastants in their environment (van Loon et al., 1992a; Miles et al., 2005). Specifically, the two species use glucosinolates as cues during caterpillar feeding and ovipositing in female butterflies, leading to similar choice of host-plant species (Smallegange et al., 2007). Moreover, both species occur in highly similar habitat types with regards to climate and succession levels. Although the two *Pieris* species share many behavioral and physiological similarities, they also show some distinctions. For instance, *P. brassicae* butterflies deposit clustered eggs and the caterpillars feed gregariously on their host plant, while *P. rapae* butterflies lay their eggs individually and the caterpillars feed solitarily on their host plant (Davies and Gilbert, 1985). In addition, the two species have quite different anti-aphrodisiac pheromone components. *P. brassicae* employs brassica lactone (Yildizhan et al., 2009) as sex pheromone and benzyl cyanide (Andersson et al., 2003) as an anti-aphrodisiac pheromone, which repels males from mating with gravid females, while *P. rapae* uses ferrulactone (Yildizhan et al., 2009) as sex pheromone and, methyl salicylate and indole as anti-aphrodisiac pheromone components (Andersson et al., 2003). The similarities and differences between the two *Pieris* species make the comparison of chemoreceptor genes valuable to understand the evolution and divergence of chemoreception in butterfly species.

It is widely acknowledged that chemoreceptors for host-plant seeking, feeding and pheromone perception drive sympatric speciation across different insect orders (Smadja and Butlin, 2009; Khallaf and Knaden, 2022). To unravel the role of the chemosensory system in defining the ecological niches utilized by the two *Pieris* species, we identified chemoreceptors in the *P. brassicae* and *P. rapae* genomes by homolog searching with queries from other lepidopteran species and conducted a phylogenetic analysis with the identified chemoreceptor sequences of the two *Pieris* species and seven other lepidopterans. The genome-guided antennal transcriptomes of both *Pieris* species were assembled and the chemoreceptors were identified as well. Moreover, homolog comparisons of each of the three chemoreceptor families in the two *Pieris* species were performed by constructing phylogenetic trees, illustrating the gene structures and searching conserved motifs to find similarities and differences within the same gene family. The gene expression levels of chemoreceptors were also compared to potentially explain the ecological overlaps and differences between the two *Pieris* species.

## 2. Materials and methods

### 2.1. Insects and tissue collection

Insects were taken from a laboratory colony of the Laboratory of Entomology, Wageningen University and Research, Netherlands. Caterpillars were reared on Brussels sprouts plants, *Brassica oleracea* var. *gemmifera* cv. Cyrus, Brussels sprouts at 22 ± 2°C, RH 70 ± 10%, light 14 h: dark 10 h until pupation. After eclosion butterflies are transferred to another cage and fed with 5% sugar water at the same environmental condition. Brussels sprouts plants were provided to collect eggs.

Adult male and female butterflies were reared in the same cage for 3 days to ensure mating. Males and females were collected to dissect antennae for transcriptome analysis. Antennae from five male and five female butterflies were pooled for each biological replicate. Three biological replicates were collected for the antennal transcriptomes of *P. brassicae* and *P. rapae*.

### 2.2. RNA isolation

Antennal samples were ground in Eppendorf tubes in liquid nitrogen and isolated and purified with ZYMO Quick-RNA/Insect Kit (ZYMO, Irvine, CA, USA). After the manual grinding, 450 μL lysis buffer was added. The RNA samples were isolated and purified according to the manual, followed by dissolving the RNA samples in 20 μL RNase-free H_2_O through centrifugation. The final RNA samples were stored at −80°C. After that the RNA concentrations were determined using a DeNovix (Wilmington, NC, USA) spectrophotometer and RNA integrity was determined by denaturing gel electrophoresis. Paired-end sequencing (150 bp) was performed by Illumina NovaSeq6000 platform in the Novogene UK sequencing center (London).

### 2.3. Average nucleotide identity measurement

Genomes of four different *Pieris* species: *P. brassicae* (GCA_942653925, MPI Chemical Ecology and GCA_905147105.1, Wellcome Sanger Institute), *P. rapae* (GCA_001856805.1) (Shen et al., 2016), *P. napi* (GCA_905475465.1), *P. macdunnoughi* (GCA_905332375.1), as well as the genomes of the outgroup butterflies *Heliconius melpomene* (GCA_000313835.2), and *Danaus plexippus* (GCA_009731565.1), *Manduca sexta* (GCF_014839805.1), *Spodoptera litura* (GCF_002706865.1) and *Phoebis sennae* (GCA_001586405.1) were collected to evaluate genome identity. The average nucleotide identity (ANI) was measured using fastANI v1.33 (Jain et al., 2018) using default settings. The detailed information of genomes that were used in this study is shown in Supplementary material 1.

### 2.4. Transcriptome assembly

The transcriptome was assembled via a genome-guided assembly method. The raw data of different transcriptomes were filtered by Fastp v0.20.0 (Chen et al., 2018) to remove short and low-quality reads and adaptors, the filtered data was subsequently assessed by FastQC v0.11.9 (Andrews, 2010). The filtered data was mapped to the corresponding genomes of *P. brassicae* (GCA_942653925) and *P. rapae* (GCA_001856805.1) with Hisat2 v2.1.0 by reporting alignments tailored for transcript assemblers (Kim et al., 2015), followed by transforming and sorting the mapped reads by SAMtools v1.10 (Li et al., 2009), the genome-guided assembly and quantification was achieved by StringTie v2.1.3b using default parameters (Pertea et al., 2015) and non-guided assembly was achieved by Trinity v2.8.6 (Grabherr et al., 2011). Transcripts per million (TPM) was employed to measure the expression levels of chemoreceptor genes in the antennal transcriptomes. In order to compare the expression of chemoreceptors between *P. brassicae* and *P. rapae*, we normalized the expression values of all the chemoreceptors to *tubulin* and *rsps20*, based on previous studies (Shakeel et al., 2015; Cusumano et al., 2018; Yin et al., 2020; Yang et al., 2021).

### 2.5. Identification of chemoreceptors

The protein sequences of ORs were collected from eight lepidopteran species: *Bombyx mori* (Tanaka et al., 2009), *M. sexta* (Koenig et al., 2015), *S. littoralis* (Walker et al., 2019), *S. exigua* (Zhang et al., 2018), *H. melpomene* (Heliconius Genome, 2012), *Plutella xylostella* (Engsontia et al., 2014), *D. plexippus* (Zhan et al., 2011), and *Helicoverpa armigera* (Liu et al., 2014), as queries to do homolog searching against the genomes of *P. brassicae* and *P. rapae*. The protein sequences of GRs were collected from nine lepidopteran species, i.e., *B. mori* (Wanner and Robertson, 2008), *M. sexta* (Koenig et al., 2015), *S. littoralis* (Walker et al., 2019), *S. exigua* (Zhang et al., 2018), *H. melpomene* (Briscoe et al., 2013), *P. xylostella* (Engsontia et al., 2014), and *D. plexippus* (Zhan et al., 2011; Briscoe et al., 2013), *H. armigera* (Liu et al., 2014) and *Cydia pomonella* (Walker et al., 2016), as queries for homolog searching. The protein sequences of IRs were collected from a total of 62 lepidopteran species, originating from three different datasets: IR sequences of 29 lepidopteran species were taken from the study by Liu et al. (2018) and IRs of 32 lepidopteran species were collected from the study by Yin et al. (2021), in addition, we also included the sequences of *S. littoralis* (Walker et al., 2019) as queries to search against the genomes of *P. brassicae* and *P. rapae* to find the candidate orthologs. Another butterfly species *Ph. sennae* (GCA_001586405.1) was introduced as an outgroup of *Pieris* to analyze the gene duplication cases.

Exonerate v2.2.0 (Slater and Birney, 2005) was applied to search against the genomes of *P. brassicae* (GCA_942653925) and *P. rapae* (GCA_001856805.1) with the collected queries to identify chemoreceptors in the two species, using the protein2genome model. Three best matched results were outputted for each query. The search results were sorted by scores. The targets sharing over 100 amino acids in a row and locating closely on the same scaffold were considered as the same receptor gene. The sequences with the highest scores were extracted, and the redundant alignment results and sequence lengths less than 100 amino acids were removed. The output results were collected as queries to further search in both genome-guided and non-guided transcriptome assemblies. The transcriptome searching results were then filtered by removing redundant and short results which are shorter than 100 amino acids. The transcripts were aligned to determine if some of them are from the same genes when they share 50 amino acids in a row. If any, the overlapped sequences will be assembled. The output transcripts and those outputs having no transcriptome targets were collected to search in NCBI database by blastp to determine if the outputs are full-length sequences. If not, the first high-similarity targets will be collected to repeat the previous steps until no longer sequence can be acquired. The identified chemosensory genes were also filtered with pfam domains (7tm_6 for ORs, Lig_chan for IRs, and 7tm_7 for GRs) to ensure the identified sequences are chemoreceptors. Diamond v0.9.25 (Buchfink et al., 2015) was later employed to identify potential chemoreceptors in the antennal transcriptomes by searching against these assemblies with an *e*-value of 10^–5^ using the identified chemoreceptors from the genomes as queries.

The protein sequences of identified chemoreceptors were collected to search against the genomes of *S. litura*, *Papilio glaucus* (GCA_000931545.1), *Ph. sennae* (GCA_001586405.1), *Vanessa tameamea* (GCF_002938995.1), *Delias pasithoe* (GCA_010014985.1), and *Calepholis nemesis* (GCA_002245505.1) in addition to the *P. brassicae* and *P. rapae* genomes, to identify the potential gene duplication cases using Exonerate v2.2.0 (Slater and Birney, 2005). The searching was performed under the protein2genome model and only the best matched target for each query was reported.

### 2.6. Phylogenetic analysis

The protein sequences of ORs, IRs and GRs from the seven lepidopteran species *B. mori*, *M. sexta*, *S. littoralis*, *S. exigua*, *H. melpomene*, *P. xylostella*, and *D. plexippus* were collected for phylogenetic analysis. The collected chemoreceptor sequences together with those of *P. brassicae* and *P. rapae* were aligned by MAFFT v7.475 (Katoh and Standley, 2013). The alignment output was imported into FastTree v2.1.11 (Price et al., 2010) for phylogenetic tree construction under the default settings with the maximum likelihood method. Olfactory receptors were rooted to Orco genes, while IRs were rooted to the iGluR clade and GRs were rooted to the sugar receptor clade. The output of FastTree was visualized and presented by the iTOL online tool v6.5.8 (Letunic and Bork, 2019).

### 2.7. Illustration of gene structures and motifs

The protein sequences of the identified chemoreceptors from the genomes were collected and imported to The MEME Suite version 5.4.1 (Bailey et al., 2015). The settings of discovery mode, sequence alphabet and site distribution were set to default, the number of motifs that should be found was set to 10. The gene structure information of the chemoreceptors was obtained by searching against the corresponding genomes (GCA_942653925 and GCA_001856805.1) with Exonerate v2.2.0 (Slater and Birney, 2005). The output of motif searching, gene structure as well as phylogenetic tree of chemoreceptors of the two *Pieris* species were integrated using TBtools v1.09876 (Chen et al., 2020). Transmembrane domains of all *Piers* chemoreceptors that we identified were predicted by TOPCONS (Tsirigos et al., 2015) and TMHMM 2.0 (Möller et al., 2001).

## 3. Results

### 3.1. Average nucleotide identity among butterfly species

Average nucleotide identity was measured to evaluate the closeness of the phylogenetic relationship among butterflies and especially for *P. brassicae* and *P. rapae*. The *P. brassicae* genome of (GCA_942653925) has a very high identity with the latest released genome version of *P. brassicae* (GCA_905147105.1) by the Wellcome Sanger Institute of 99.14%, confirming the validity of both genome assemblies. *P. brassicae* shares relatively high similarities with the other three species belonging to the genus *Pieris*: 86.95, 86.84, and 86.01% with *P. macdunnoughi*, *P. napi*, and *P. rapae*, respectively, indicative of a very close relationship among the four *Pieris* species. The ANI decreases with increasing phylogenetic distance within the Lepidoptera (Table 1). For instance, the *P. brassicae* genome is less identical with *D. plexippus* (75.57%), *H. melpomene* (75.34%), *S. litura* (75.32%), and *M. sexta* (75.12%). *Ph. sennae* also belongs to the Pieridae and has a slightly higher ANI (76.23%) with the four *Pieris* species than those species belonging to other families.

**TABLE 1:** Average nucleotide identity (ANI, %) matrix of multiple lepidopteran species.

	*Pieris brassicae* (MPI-CE)	*Pieris brassicae* (Wellcome)	*Pieris macdunnoughi*	*Pieris napi*	*Pieris rapae*	*Phoebis sennae*	*Danaus plexippus*	*Heliconius melpomene*	*Spodoptera litura*
*Pieris brassicae* (MPI-CE)	–								
Pieris brassicae (Wellcome)	99.1419	–							
*Pieris macdunnoughi*	86.9454	86.9118	–						
*Pieris napi*	86.8398	86.8383	93.0917	–					
*Pieris rapae*	86.0091	85.9859	86.6698	86.6747	–				
*Phoebis sennae*	76.2277	76.2132	76.3453	76.3768	76.4574	–			
*Danaus plexippus*	75.5695	75.511	75.661	75.6576	75.5971	75.6983	–		
*Heliconius melpomene*	75.3448	75.3294	75.4328	75.4214	75.436	75.6557	75.921	–	
*Spodoptera litura*	75.3224	75.2882	75.5038	75.512	75.423	75.7212	75.5742	75.4558	–
*Manduca sexta*	75.1239	75.1206	75.2343	75.2412	75.2142	75.4476	75.2769	75.3143	75.9012

### 3.2. Identification of ORs

**FIGURE 1:**
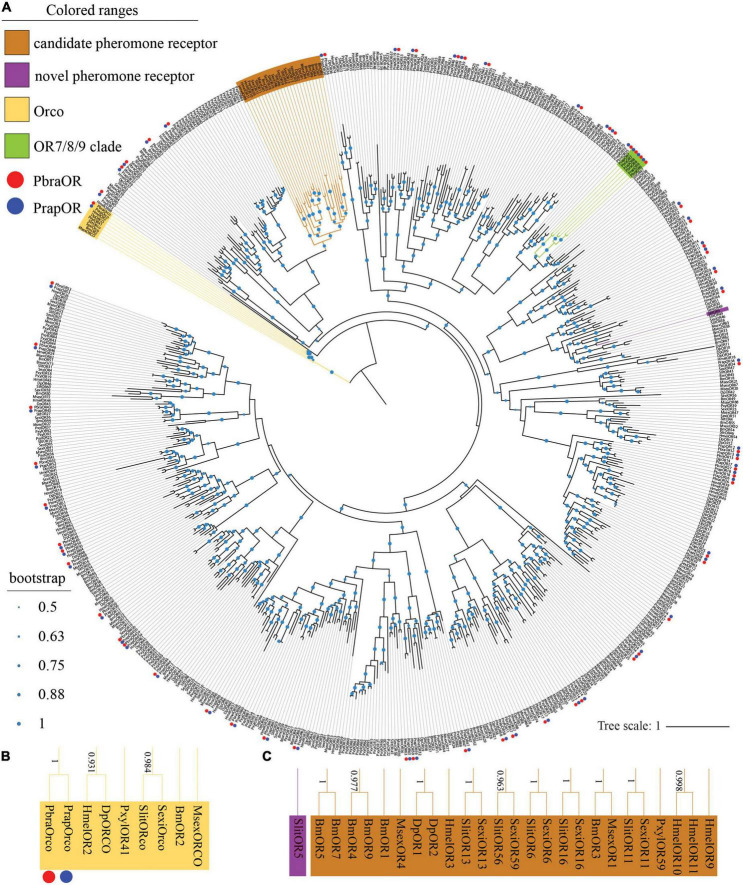
Phylogenetic tree of lepidopteran odorant receptor genes. **(A)** The phylogeny was rooted using the *Orcos*. *PbraOR*, *Pieris brassicae* OR; *PrapOR*, *P. rapae* OR; *BmOR*, *Bombyx mori* OR; *MsexOR*, *Manduca sexta* OR; *DpOR*, *Danaus plexippus* OR; *PxylOR*, *Plutella xylostella* OR; *HmelOR*, *Heliconius melpomene* OR; *SexiOR*, *Spodoptera exigua*; *SlitOR*, and *S. littoralis*. The candidate pheromone receptor clade is labeled in light brown, the novel pheromone receptor clade was labeled in purple, the *Orco* clade is labeled in yellow and the OR7/8/9 clade is labeled in green. The green dots in the middle of the branches of the phylogenetic tree indicate the bootstrap values. The scale bar represents the number of substitutions per site. The *Orco* clade and candidate pheromone receptor/novel pheromone receptor clade are magnified in panels **(B,C)**, respectively, the bootstrap values higher than 0.5 are shown alongside the branches. *PbraORs* and *PrapORs* are labeled with red and blue dots, respectively.

All identified ORs (see Supplementary materials 2, 3) are named after *D. plexippus* because the ORs of *P. brassicae* and *P. rapae* share a relatively close relationship with *D. plexippus* according to phylogenetic analysis (Figure 1A). A total of 60 ORs were identified in both *P. brassicae* and *P. rapae* (Figure 1A) including the conserved odorant receptor coreceptor (Orco). The clustering of *PbraOrco* and *PrapOrco* with the Orco genes of other lepidopteran species was supported by high bootstrap values (Figure 1B). The reported pheromone receptors of moths such as *B. mori*, *M. sexta*, *S. littoralis*, *S. exigua*, and *P. xylostella* also cluster (Figure 1C), and show four *HmelORs* and one *DpleOR* as potential novel pheromone receptors. No pheromone receptors of *P. brassicae* and *P. rapae* were found in the pheromone receptor clade. Some gene duplication cases were found in the phylogenetic tree as well, such as the OR7/8/9 clade which is formed by the OR7, OR8, and OR9 of the two species with high bootstrap values.

### 3.3. Identification of IRs

**FIGURE 2:**
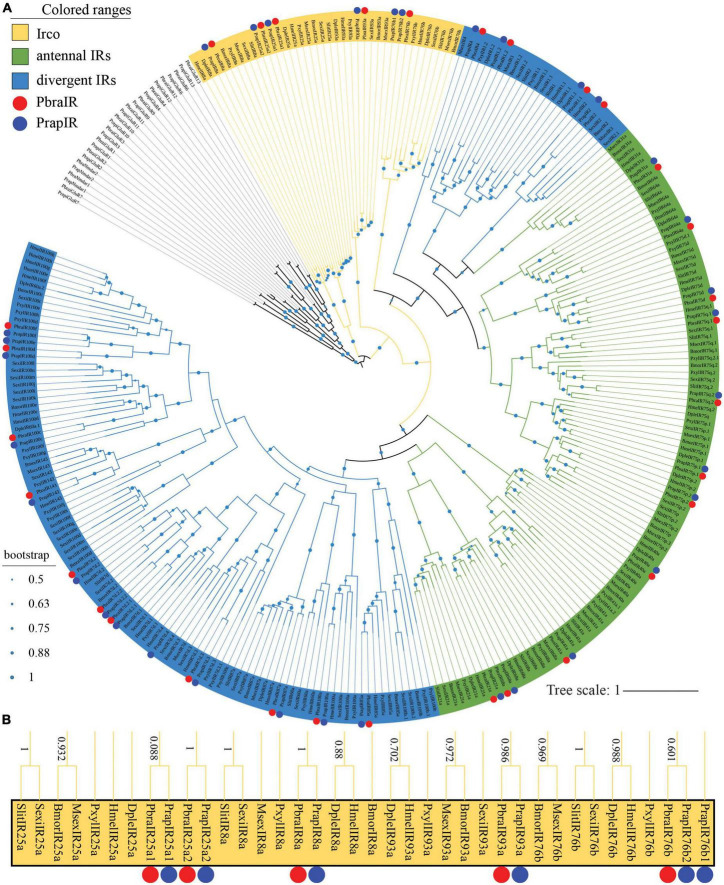
Phylogenetic tree of lepidopteran ionotropic receptor genes. **(A)** The phylogeny was rooted using iGluRs of *P. brassicae* and *P. rapae*. *PbraIR*, *Pieris brassicae* IR; *PrapIR*, *P. rapae* IR; *BmorIR*, *Bombyx mori* IR; *MsexIR*, *Manduca sexta* IR; *DpleIR*, *Danaus plexippus* IR; *PxylIR*, *Plutella xylostella* IR; *HmelIR*, *Heliconius melpomene* IR; *SexiIR*, *Spodoptera exigua* IR; *SlitIR*, and *S. littoralis* IR. The *Irco* clade is labeled in yellow, the antennal IR clade is labeled in green and the divergent IR clade is labeled in blue. The green dots in the middle of the branches of the phylogenetic tree indicate the bootstrap values that are higher than 0.5. The scale bar represents the number of substitutions per site. **(B)** *Irco* clade is magnified, and bootstrap values higher than 0.5 are shown alongside the branches. *PbraIRs* and *PrapIRs* are labeled with red and blue dots, respectively.

Thirty-one *PbraIRs* and 34 *PrapIRs* (see Supplementary materials 2, 3) were identified from the *P. brassicae* and *P. rapae* genome, respectively. *PbraIRs* and *PrapIRs* were named after *H. melpomene* since *HmelIRs* are closer to most *PbraIRs* and *PrapIRs* in the phylogenetic tree compared to IRs from other species (Figure 2A). Sixteen out of the 31 *PbraIRs* and 17 out of the 34 *PrapIRs* were annotated in the antennal IRs clade (Figure 2A, yellow and green in the phylogenetic tree). Moreover, five *PbraIRs* (*PbraIR8a*, *PbraIR25a*, *PbraIR25a*, *PbraIR76b*, and *PbraIR93a*) and six *PrapIRs* (*PrapIR8a*, *PrapIR25a1*, *PrapIR25a2*, *Prap76b1*, *PrapIR76b2*, and *PrapIR93a*) were identified as ionotropic receptor co-receptors (Ircos) (Figure 2B). *PrapIR76b1* and *PrapIR76b2* share a very high similarity of more than 90%, their corresponding DNA sequences have a length of 2,072 bp on the same scaffold with the same direction, although there is only one *IR76b* gene found in *P. brassicae* genome. *PrapIR25a1* and *PrapIR25a2* share a lower identity of 76% with a 1,509 bp interval and, *PbraIR25a1* and *PbraIR25a2* share a 75% similarity with a 10,008 bp interval on the same scaffold with the same direction. Fifteen out of 31 *PbraIRs* and 17 out of 34 *PrapIRs* were annotated in divergent IRs clade in blue in the phylogenetic tree (Figure 2). The orthologs of *PrapIR7d*.4 and *PrapIR100e* were not found in the *P. brassicae* genome. Thirty-two out of 34 *PrapIRs* have been identified previously (Liu et al., 2018), the two newly identified genes are *PrapIR4* and *PrapIR25a2*.

### 3.4. Identification of GRs

**FIGURE 3:**
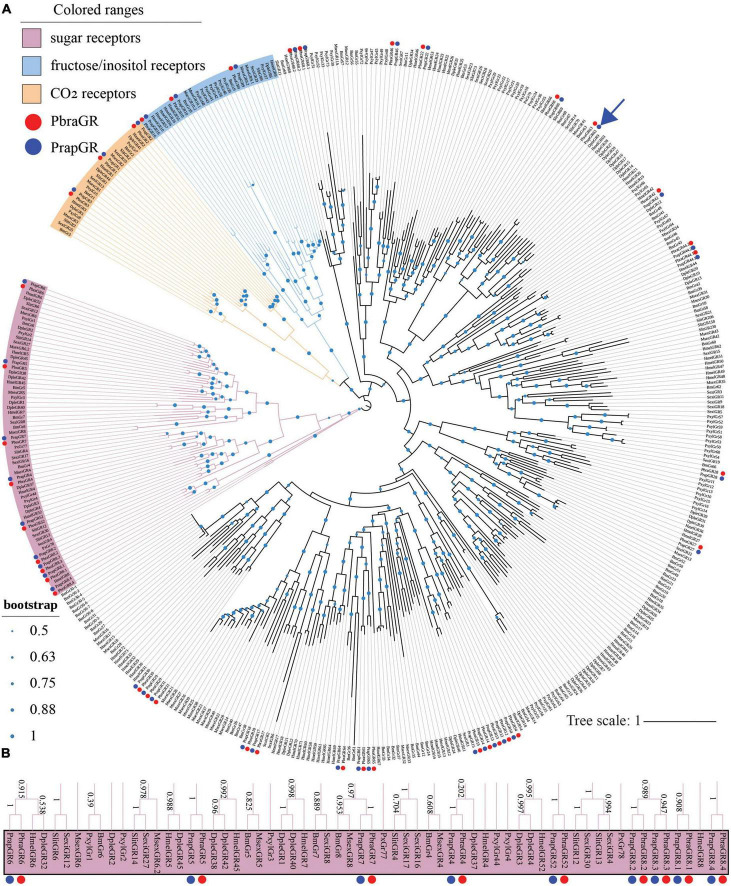
Phylogenetic tree of lepidopteran gustatory receptors. The phylogeny was rooted using sugar receptors. *PbraGR*, *Pieris brassicae* GR; *PrapGR*, *P. rapae* GR; *BmGR*, *Bombyx mori* GR; *MsexGR*, *Manduca sexta* GR; *DpleIR*, *Danaus plexippus* GR; *PxylGR*, *Plutella xylostella* GR; *HmelGR*, *Heliconius melpomene* GR; *SexiGR*, *Spodoptera exigua* GR; *SlitGR*, and *S. littoralis* GR. **(A)** The CO_2_ receptor clade is labeled in yellow; the sugar receptor clade is labeled in magenta; the fructose/inositol clade is labeled in blue; the deorphanized sinigrin receptor in *P. rapae* (Yang et al., 2021) is indicated by a blue arrow. The green dots in the middle of the branches of the phylogenetic tree indicate the bootstrap values higher than 0.5. The scale bar represents the number of substitutions per site. **(B)** The sugar receptor clade is magnified, and bootstrap values are shown alongside the branches.

Thirty-nine GRs (see Supplementary materials 2, 3) were identified in both the *P. brassicae* and *P. rapae* genomes. All identified GRs were named after *H. melpomene* because most *PbraGRs* and *PrapGRs* clustered with *HmelGRs* (Figure 3A), just like the IRs presented above. Based on former publications (Wanner and Robertson, 2008; Yang et al., 2020), three *PbraGRs* and *PrapGRs* were annotated as CO_2_ receptors (Figure 3A, in yellow), three *PbraGRs* and *PrapGRs* were annotated as fructose/inositol receptors (Figure 3A, in blue) and nine *PbraGRs* and *PrapGRs* were annotated as sugar receptors (Figures 3A, B in pink) and a sinigrin receptor of *P. rapae* (Yang et al., 2021) was indicated by the blue arrow in Figure 3A.

### 3.5. Gene structure and conserved motif of chemoreceptors

**FIGURE 4:**
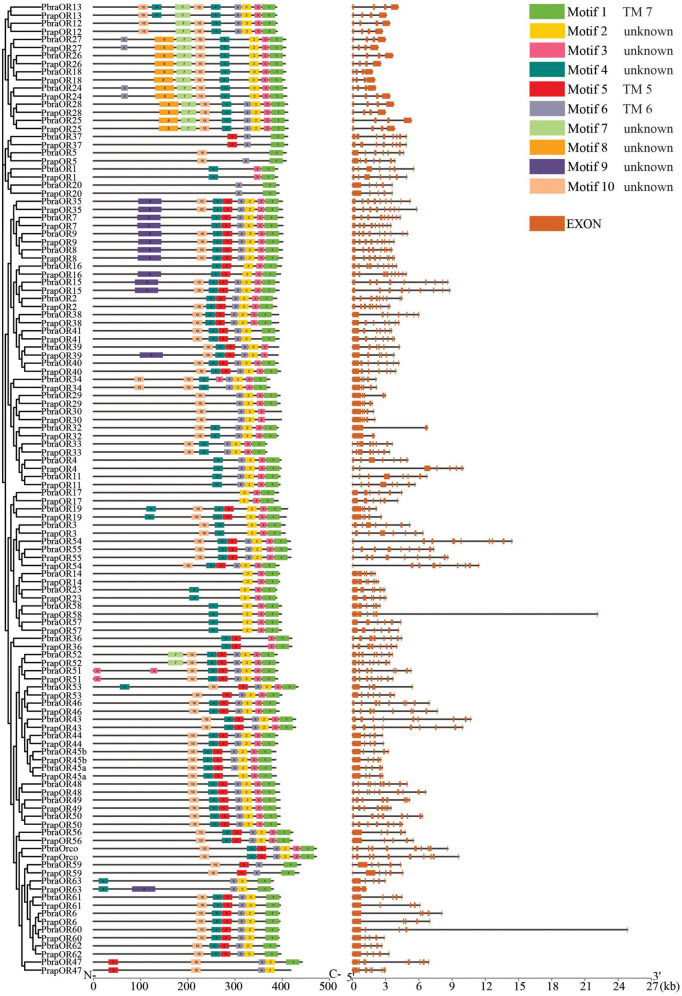
Conserved motifs and arrangements and gene structures of odorant receptors of *Pieris brassicae* and *P. rapae*. Scale bars under conserved motifs and gene structures represent the protein length from N- to C- terminus and the transcript length from the 5′ end to the 3′ end. The legend indicates the conserved motifs that are found in the chemoreceptors and the exons that are found in the transcript.

**FIGURE 5:**
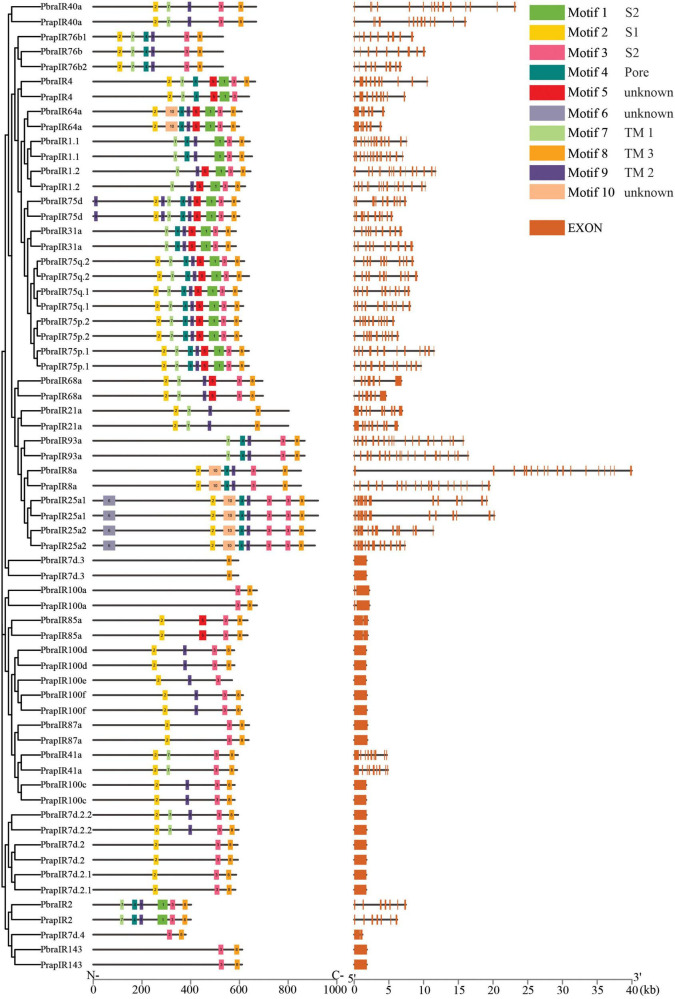
Conserved motifs and gene structures of ionotropic receptors of *Pieris brassicae* and *P. rapae*. Scale bars under conserved motifs and gene structures represent the number of amino acids and the number of nucleotides from the 5′ end to the 3′ end.

**FIGURE 6:**
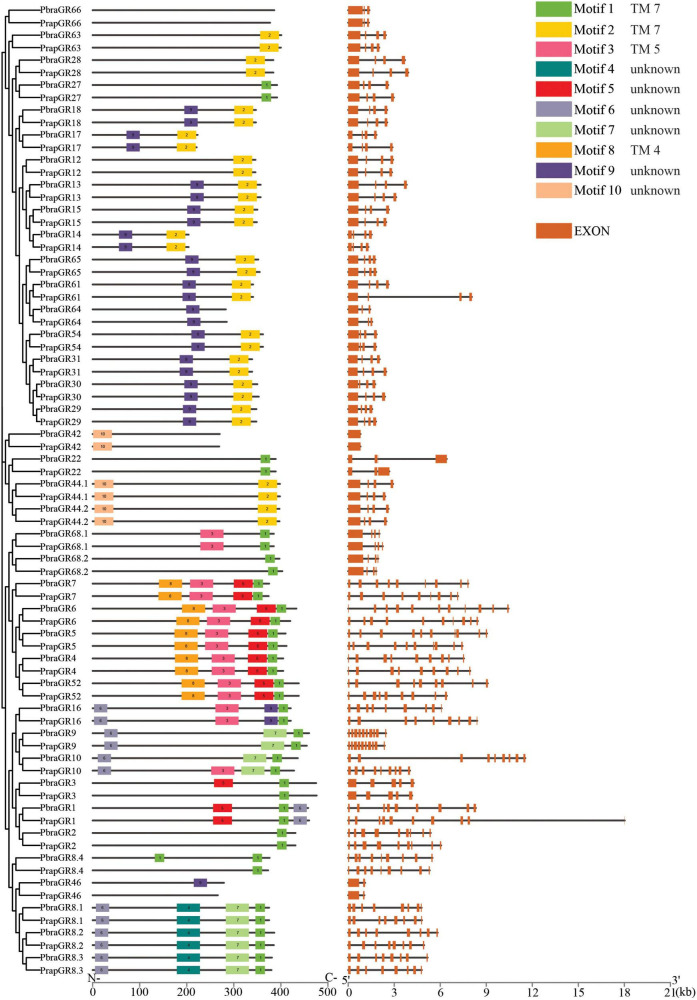
Conserved motifs and gene structures of gustatory receptors of *Pieris brassicae* and *P. rapae*. Scale bars under conserved motifs and gene structures represent the number of amino acids and the number of nucleotides from the 5′ end to the 3′ end.

Based on the chemoreceptor sequences identified from the genomes of the two *Pieris* species, we compared the full-length gene structures and analyzed the conserved protein motifs and the arrangements of motifs. We found that most chemoreceptors have a comparable gene structure with other orthologs and with the same number of introns but sometimes with different lengths. For example, *PrapOR58* and *PbraOR60* have much longer introns than their orthologs (Figure 4). Interestingly, we found that most of IRs in the divergent IRs clade annotated in Figure 3 have no intron at all, such as *IR7d.3*, *IR100c*, *IR100d*, *IR100e*, *IR100f*, *IR87a*, *IR7d.2*, *IR7d.2.1*, *IR7d.2.2*, and *IR143*; three IRs, i.e., *IR85a*, *IR100a*, and *IR7d.4*, have only one very short intron. Notably, the orthologs having no or only one short intron share highly similarly conserved motifs and arrangements. The other IRs in the divergent IRs clade, i.e., *IR1.1*, *IR1.2*, *IR2*, and *IR4*, have multiple introns similar to those in antennal IRs clade (Figure 5). However, we also found some exceptions in GRs that showed different number of introns, specifically *PbraGR1* has 8 introns while *PrapGR1* has 9 introns (Figure 6).

Multiple motifs (sequences see Supplementary material 4) are found in the chemoreceptor protein sequences. Motifs 1, 2, 3, 4, and 5 are the most abundant conserved motifs and are also found in most ORs at similar position of the sequences in both species (Figure 4). According to our transmembrane domain prediction results (Supplementary material 5), we found that ORs exhibit six to seven transmembrane domains. Interestingly, the location of Motif 1 is highly consistent with that of the last transmembrane (TM) domain 7, location of Motif 6 overlaps with TM 6, and Motif 5 covers TM 5 in most ORs. The other conserved motifs are located in intracellular or extracellular loops.

In the IRs Motif 8 is present in all sequences, while Motif 2 is found in most IR sequences. In contrast, Motif 6 is only present in sequences of IR25a1/2 of both species (Figure 5), which is located in amino-terminal domain (ATD). According to the transmembrane predictions, IRs have three to four transmembrane domains. Here, locations of identified conserved motifs are highly consistent with known domains in IRs (Benton et al., 2009; Croset et al., 2010). Motifs 7, 9, and 8 are TM 1, TM 2, and TM 3, respectively. Motif 4 overlaps with the pore domain (P). In addition, Motif 2 was found to locate in ligand-binding domain (LBD) S1 and, Motif 1, and Motif 3 were found to locate in LBD S2.

Compared to ORs and IRs which have four to nine conserved motifs, GRs have fewer conserved motifs in their sequences; for instance, no conserved motif has been found in GR6 and over half of the GRs have only 1–2 conserved motifs (Figure 6). The most common motifs 1 and 2 are only found in about half of the GR sequences. Transmembrane domain prediction results showed that most GRs have six to eight transmembrane domains. Locations of Motif 1 and Motif 2 match well with TM 7 (or the last transmembrane domain) of GRs. Motif 3 and Motif 8 covers TM 5 and TM 4, respectively while the two conserved motifs did not strictly match with the transmembrane domains of GRs.

### 3.6. Gene duplication and microsynteny

Based on the identification and phylogenetic analysis of chemoreceptors, we found some gene duplication cases. Clade *OR18*/*OR26*/*OR27*, clade *OR48*/*OR49*/*OR50*, and clade *OR7*/*OR8*/*OR9* are positioned closely on the same scaffold and share high similarities of protein sequences in both *Pieris* species. Similar cases can also be found in IRs and GRs. *IR25a1*/*IR25a2* and *IR100d/IR100f* of both species and *PrapIR76b1*/*PrapIR76b2* are placed closely on the same scaffold and show a very high sequence identity. This was also found for *GR8.1*/*GR8.2*/*GR8.3*. Interestingly, while most chemoreceptors mentioned above have the same number of exons, *OR49*/*OR50* have eight exons and are different from OR48 which has ten exons.

**FIGURE 7:**
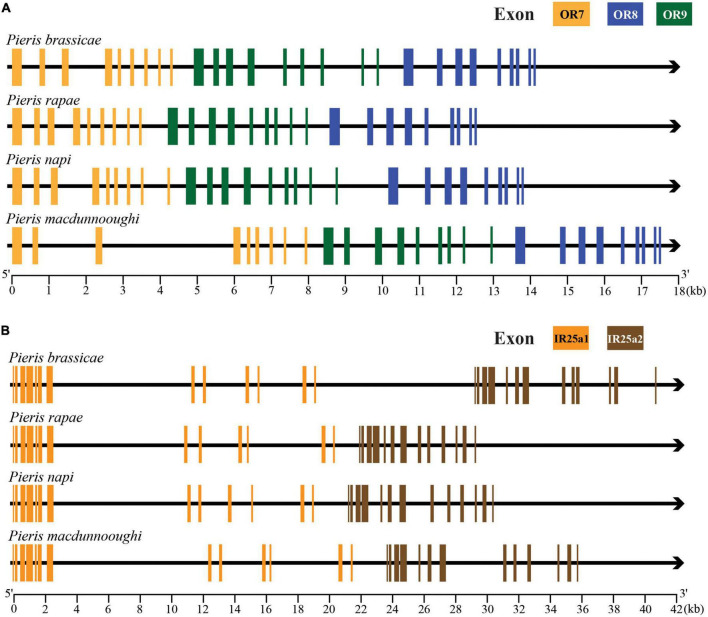
Microsyntenic patterns of OR7/8/9 and IR25a in *Pieris*. **(A)** Gene structures and positions of clade *OR7*/*OR8*/*OR9*. Exons of OR7, OR8, and OR9 are labeled in yellow, blue and green, respectively. **(B)** Gene structures and positions of clade *IR25a1*/*IR25a2*. The exons of the IR25a1 and IR25a2 are labeled in orange and brown, respectively. The black arrows indicate introns and directions from 5′ end to the 3′ end. The scales at the bottom of gene structures indicate the length of depicted genes in nucleotides.

In order to clearly elucidate the potential gene duplication and microsyntenic pattern of chemoreceptors in *Pieris* species, we selected *OR7*/*OR8*/*OR9* (Figure 7A) and *IR25a1*/*IR25a2* (Figure 7B) as targets to extract their relative positions and gene structures in *P. brassicae*, *P. rapae*, *P. napi*, and *P. macdunnoughi*. We found that the four selected *Pieris* species have these target genes, and these paralogs are closely located on the same scaffold in the same order and orthologs share the same number of exons and even share similar exon lengths. In contrast, the ortholog of *OR7* is not present in the *Pieris* outgroup species *Ph. sennae* and no *IR25a* duplication was found in any of the selected non-*Pieris* lepidopteran species (*S. litura*, *Pa. glaucus*, *V. tameamea*, *Ph. sennae, D. pasithoe*, and *C. nemesis*).

### 3.7. Expression of chemoreceptors in adult transcriptome

**FIGURE 8:**
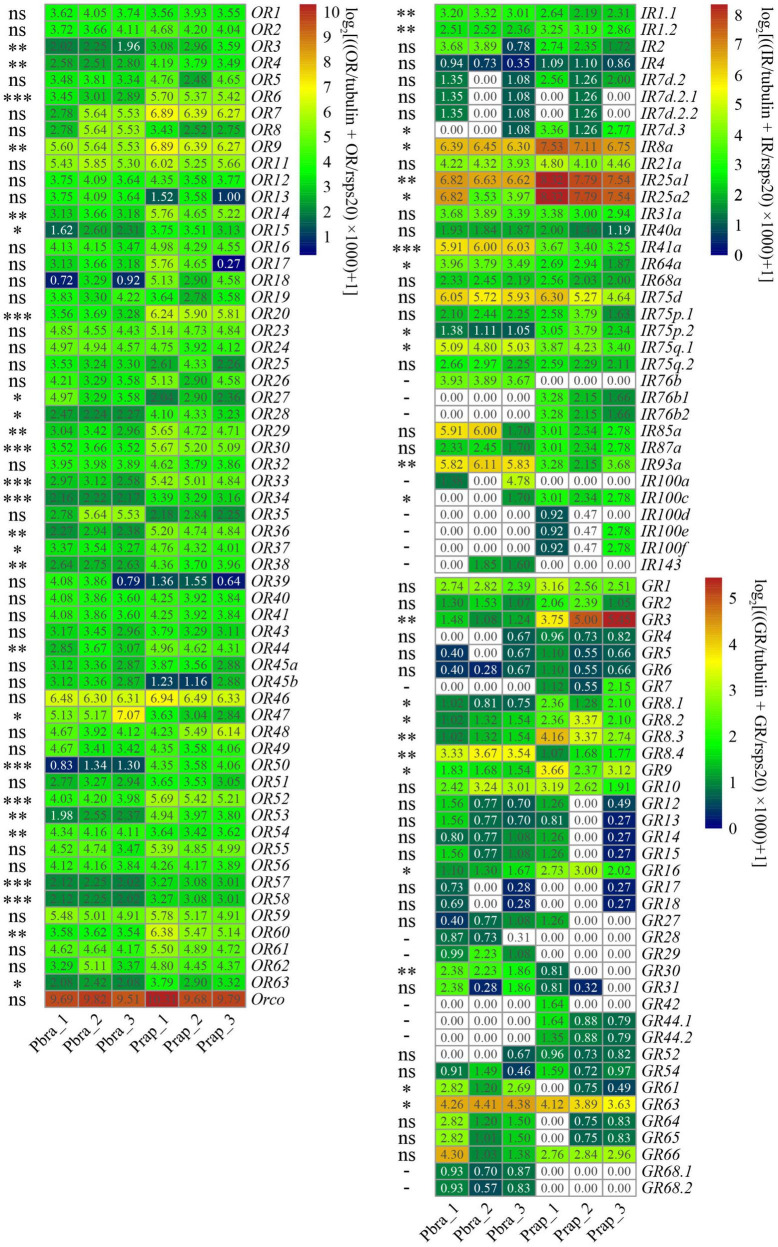
Heatmap of transcriptome of genes encoding chemoreceptors in antennae of *Pieris brassicae* and *P. rapae*. The TPM values were normalized by log_2_ [(TPM _chemoreceptor_/TPM *_*tubulin*_* + TPM _chemoreceptor_/TPM *_*rsps*20_*) × 1,000 + 1]. The scales of transformed normalized heatmap values of ORs, IRs, and GRs are 1–10, 0–8, and 0–5, respectively. The white cells indicate that the genes were not found in the transcriptome. values less than 2.0 are in white for ORs and values less than 1.0 are in white for IRs and GRs. Significant difference was detected by *t*-test with normalized TPM values and labeled as * (*P* < 0.05), ^**^ (*P* < 0.01), ^***^ (*P* < 0.001) or ns (no significance, *P* > 0.05), –(significant difference was not calculated because the genes were not found).

We compared the chemoreceptor identification results of the *de novo* assembly and genome-guided assembly and found the results are identical, although *de novo* assembly did not always reveal the complete sequences. Sixty *PbraORs* and 60 *PrapORs* are identified in the adult antennal transcriptome of *P. brassicae* and *P. rapae*, respectively, Orco is the highest expressed gene among chemoreceptors in the transcriptomes. However, out of the 60 genes, the expression levels of 25 ORs were found to be significantly different between the transcriptomes of the two *Pieris* species. Twenty-two of the 25 differently expressed *ORs* showed higher expression levels in the *P. rapae* antennal transcriptome, *OR27*, *OR47*, and *OR54* had higher expression levels in the *P. brassicae* antennal transcriptome (Figure 8).

Twenty-nine and 31 *IRs* were identified from the *P. brassicae* and *P. rapae* adult antennal transcriptomes, respectively. Ircos *PbraIR8a*, *PbraIR25a1*, *PbraIR93a* and those IRs in the antennal IRs clade *PbraIR41a*, *PbraIR75d*, *PbraIR75q.1*, and *PbraIR85a* were highly expressed in the antenna of *P. brassicae*. Out of these, 12 IRs were found differently expressed between the two transcriptomes. Seven (*IR1.2*, *IR7d.3*, *IR8a*, *IR25a1*, *IR25a2*, *IR75p.2*, and *IR100c*) out of the 12 *IRs* were found to be higher expressed in the *P. rapae* antennal transcriptome, such as IR8a, *IR25a1*, and *IR25a2* than *P. brassicae* and five of the 12 IRs (*IR1.1*, *IR41a*, *IR64a*, IR75q.1, and *IR93a*) had higher expression levels in the *P. brassicae* antennal transcriptome. In addition, *IR76b1, IR76b2*, and IR *100d*/*e*/*f* were not found in the antennal transcriptome of *P. brassicae*; some of these genes (*IR76b1*, *IR76b2*, and *IR100e*) were only found in the *P. rapae* genome. *IR76b*, *IR100a*, and *IR143* are exclusively found in the *P. brassicae* transcriptome (Figure 8).

In total, 33 GRs were identified from both the *P. brassicae* and the *P. rapae* adult antennal transcriptome, although several genes were only identified in one of the two species. Compared to ORs and IRs, GRs have comparatively lower expression in the adult antennal transcriptomes of both species according to normalized TPM values. The sinigrin receptor *GR63* was highly expressed in both antennal transcriptomes of *P. brassicae* and *P. rapae*. Ten *GRs* were found differently expressed between the two transcriptomes. Six (*GR3*, *GR8.1*, *GR8.2*, *GR8.3*, *GR9*, and *GR16*) of these ten *GRs* showed higher expression in the *P. rapae* antennal transcriptome, the other four GRs (*GR8.4*, *GR30*, *GR61*, and *GR63*) showed higher expression in the *P. brassicae* antennal transcriptome. Notably, four *PbraGRs* (*PbraGR7*, *PbraGR42, PbraGR44.1*, and *PbraGR44.2*) and four *PrapGRs* (*PrapGR28*, *PrapGR29*, *PrapGR68.1*, and *PrapGR68.2*) were not detected in the corresponding antennal transcriptomes. These undetected GRs are displayed in white cells in Figure 8.

## 4. Discussion

In order to clarify the role that chemoreception might play in the ecological adaptation of *P. brassicae* and *P. rapae*, we compared the qualitative and quantitative differences in the receptor sets of these two species through genome and transcriptome analyses. In this study we measured the ANI among several butterfly and moth species and found that *Pieris* species share relatively high sequence identities in comparison to the other lepidopterans. *P. brassicae* and *P. rapae* are closely related species, having a similar geographical distribution and host-plant range, but differ in their oviposition strategy and their larval feeding behavior (Feltwell, 1982; Davies and Gilbert, 1985; Ryan et al., 2019). Here, we identified 60 *PbraORs*, 31 *PbraIRs*, and 39 *PbraGRs* from the *P. brassicae* genome and 60 *PrapORs*, 34 *PrapIRs*, and 39 *PrapGRs* from the *P. rapae* genome by searching against genomes with lepidopteran homologs. The gene structures and conserved motifs of chemoreceptors in the two *Pieris* species are identical to their orthologs in the other species. We then analyzed the adult transcriptome data based on the identified chemoreceptors in the genomes of the two *Pieris* species. We found that 60 ORs, 29 IRs, and 33 GRs were expressed in the *P. brassicae* antennae, while 60 ORs, 31 IRs, and 33 GRs were expressed in the antennae of *P. rapae*. The co-receptors such as *Orco*, *IR8a*, and *IR25a* were also found in the transcriptome with very high expression levels. For most chemoreceptor genes homologs were found in both antennal transcriptomes. However, the expression levels of some specific receptor genes differed between the transcriptomes of the two species.

The electrophysiological and behavioral responses to odorants and tastants of the two *Pieris* species have been extensively investigated over the last decades. These studies showed different sensitivities to amino acids and volatiles for *P. brassicae* and *P. rapae*. Specifically, histidine, phenylalanine, and tryptophan are the amino acids that elicit the strongest responses in the lateral sensilla of *P. brassicae* caterpillars while the three amino acids caused the weakest responses in the same sensillum of *P. rapae* (van Loon and van Eeuwijk, 1989). Similarly, electroantennogram (EAG) analysis showed that *P. brassicae* and *P. rapae* butterflies responded to the same tested chemicals, but with a different responsiveness: trans-hex-2-enal elicited the strongest response in *P. brassicae* whereas *P. rapae* showed similarly strong responses to trans-hex-2-enal, hexan-1-ol, and hexanal without significant difference among the three chemicals (van Loon et al., 1992b). However, the mechanism underlying the different responses to these compounds is still unclear. In our study we found significant differences in the expression of several gustatory and ORs and it is plausible that these differences are responsible for the observed contrasts in the chemosensory responses of the two species (Figure 8).

In recent years, identification of chemoreceptors has been performed extensively in a wide range of insect species and some receptors have been functionally characterized as well. The glucosinolates such as glucobrassicin and sinigrin strongly elicit egg-laying behavior in *P. rapae* and *P. brassicae* (Renwick and Radke, 1983; Traynier and Truscott, 1991; van Loon et al., 1992a). Sinigrin was recently found as an agonist of a gustatory receptor in *P. rapae*. The sinigrin receptor GR28 (named *GR63* in this study) was characterized at a molecular level by expression in *Xenopus* oocytes and two-electrode voltage clamp recordings, showing that GR28/GR63 recognizes sinigrin specifically (Yang et al., 2021). Interestingly, we found that *GR63* is one of the most highly expressed GR genes in both *P. brassicae* and *P. rapae*, with similar expression levels in both species, which corresponds with their similar host-plant choice (Du et al., 1995).

Functional characteristics of IRs seem to be much more divergent than ORs and GRs and even go beyond chemoreception (Wicher and Miazzi, 2021). For example, the *Drosophila* IR25a is involved in setting the circadian clock by detecting temperature through sensors on the antennae (Chen et al., 2015) and sensing moisture (Knecht et al., 2017). ORs could have originated from GRs in arthropods (Robertson et al., 2003; Robertson, 2019) while IRs are already expressed in the olfactory organs of protostomes, indicating that IRs could be as old as the protostomes and *IR25a* could be the oldest one (Croset et al., 2010). Interestingly we found that two *IR25a* paralogs are located on the same scaffold in close proximity in *Pieris* and we therefore designated the *IR25a* paralogs as a duplication event. This *IR25a* duplication only occurred in the four *Pieris* species (*P. brassicae*, *P. rapae*, *P. napi*, and *P. macdunnoughi*) which we tested here, but not in any of the other butterfly and moth species that we analyzed. Beyond *Pieris*, *Irco* duplications were also found in parasitoid wasp *Microplitis mediator* (Wang et al., 2015; Wang S. N. et al., 2016) and *Aphidius gifuensis* (Kang et al., 2017). Here we speculate that the second *IR25a* gene in *Pieris* evolved after the speciation from the last common *Pieris* ancestors, but before the divergence of *Pieris*. The physiological difference between the two *IR25a* genes is still unclear and needs to be clarified in the future, although we speculate that the differentiation of *IR25a* in the *Pieris* species would improve the efficiency of their interaction with both the biotic as well the abiotic environment considering the multiple functions of this receptor in insects (Ni et al., 2016; Shan et al., 2019).

The evolution of chemoreceptor families is very complicated and often related to specific ecological pressures (Hansson and Stensmyr, 2011). Similar to the duplication of *IR25a*, we also found cases in other chemoreceptor families, such as the clade *OR7*/*OR8*/*OR9*. These duplications were also found in other *Pieris* species, but not in other Pieridae species, which suggests that these selected genes also arose in the last common ancestor of the *Pieris* species. Gene duplication events in olfactory receptors are often related to specific adaptations such as a shift in diet preference (Goldman-Huertas et al., 2015) and it is conceivable that the fact that this specific duplication which can be found in all *Pieris* species, but not in the close relatives, might be related to some of the multiple adaptation events that took place in the co-evolution of the Pieridae and the Brassicales plants (Edger et al., 2015).

Similarly, to the role gene duplication plays for natural selection, sex pheromone perception has been argued to have strongly contributed to speciation in some lepidopterans, especially moths (Smadja and Butlin, 2009; Leary et al., 2012). Pheromone receptors (PRs) were found and investigated in many species of various insect orders already (Sakurai et al., 2004; Wicher et al., 2017; Wang et al., 2021). However, we did not find any potential PR in the *Pieris* species in this study, even though our phylogenetic analysis did detect all the previously identified pheromone receptors in the species we used for our comparison (Kaissling and Kasang, 1978; Chisholm et al., 1983; Tumlinson et al., 1989; Kalinová et al., 2001). Until now, the pheromone components of only a few butterfly species have been reported (Meinwald et al., 1969), among them *P. brassicae* and *P. rapae*. The large cabbage white, *P. brassicae*, uses brassica lactone as a sex pheromone and benzyl cyanide as an anti-aphrodisiac compound, while the small cabbage white, *P. rapae*, uses ferrulactone as a sex pheromone and methyl salicylate and indole as anti- aphrodisiacs (Andersson et al., 2003; Yildizhan et al., 2009). The identified pheromone components of the different butterfly species analyzed so far show a diversity of chemical structures and are mostly unrelated to known sex pheromone components of moths. In addition, no butterfly PR has been identified and functionally deorphanized although abundant PRs have been characterized in different moth species (Krieger et al., 2005; Mitsuno et al., 2008). It is therefore likely that the butterfly PRs are not phylogenetically closely related to the moths’ PRs, but might rather be an independent lineage that detect compounds with a different chemical structure (Bastin-Heline et al., 2019).

In addition to the molecular basis of pheromone perception, the ORs for the recognition of general odors such as plant volatiles and floral odorants in moths have been extensively deorphanized (Tanaka et al., 2009; Cao et al., 2016; de Fouchier et al., 2017; Liu et al., 2020); however, in butterflies, the other large group of lepidopterans, no ORs have been deorphanized so far. This knowledge gap makes it challenging to comprehensively assess the significance of chemoreception in butterflies and hinders a comparison between butterflies and moths, which would help to better understand the divergence between these two groups.

According to previous reports, ORs are highly divergent among insects, sharing low identities with their paralogs even in the same species (Clyne et al., 1999). However, we found that the paralogs among the two *Pieris* species share highly conserved motifs and a highly similar motif layout, indicating that chemoreceptors are relatively conserved between the two species. Compared to ORs, IRs are more conserved across insect species with specific domains such as ATD, ligand-binding domain (LBD), transmembrane domain (TMD) and pore (P), which was also confirmed by our motif searching results of the IRs. Similar to ORs, GRs were thought to be highly divergent (Clyne et al., 2000), although we found that GRs share similar motifs and motif layouts with their paralogs in the two *Pieris* species with only a few exceptions found in this study. This suggests that GRs are also rather conserved chemoreceptors between the two *Pieris* species from the perspective of their protein sequences (Robertson, 2019). These similarities in the gene structure and in the number of chemoreceptor genes in the two *Pieris* species contrasts to some extent with the rapid gene loss and duplication of chemoreceptor genes that has been found in some *Drosophila* species (Gardiner et al., 2008; Goldman-Huertas et al., 2015; Auer et al., 2020). However, in many of the well-studied examples the different fly species are not only ecological but also geographically separated (Khallaf and Knaden, 2022). This makes it difficult to directly compare the evolution of sensory genes in drosophilid flies with the two *Pieris* species, which are not only overlapping in large parts of their geographic range, but also with regards to their behavioral ecology. Interestingly, the differential expression of genes instead of the *de novo* gene gain could also lead to the divergence of populations within the same species (Sikkink et al., 2017).

The behavior of insects is greatly influenced by the expression of genes encoding for chemoreceptors. The silencing or knocking out of chemoreceptor genes can greatly change the physiology and behavior of insects across different orders (Trible et al., 2017; Yan et al., 2017; Fandino et al., 2019). The higher expression of genes encoding for GRs in female butterflies also facilitates finding an optimal oviposition site (Briscoe et al., 2013). In our study, most chemoreceptors had similar expression levels in the two *Pieris* species and we speculate that those genes that had similar expression levels, conserved motifs and gene structures, represent a conserved set of chemoreceptors, which are involved in the behavioral traits shared between both species while the differential expression of certain other chemoreceptor orthologs could be involved in more species specific adaptations (Teets et al., 2012; Birnbaum and Abbot, 2020). To fully identify all candidate chemoreceptors in the two *Pieris* butterfly species, we employed antennal transcriptomic data, which included both male and female antennae samples. Although, combing male and female tissues has provided us with greater certainty on the identification of individual genes, we were unable to compare differential expression of chemoreceptors between the sexes of the two species. Analyzing the sex-biased expression in butterfly chemoreceptors will be an interesting topic for future research, especially in comparison to similarly biased receptors in different moths’ species.

In this study, we identified and systemically compared the chemoreceptors of *P. brassicae* and *P. rapae* based on genomic data and antennal transcriptomes. By searching for conserved motifs of chemoreceptors and gene structures, we found that although all chemoreceptors are highly similar to their orthologs in other closely related species, several genes varied significantly in their expression levels. The comparison of the chemoreceptors between these two closely related species, therefore, highlights both the similarities and differences in the structure and expression of chemoreceptors and provides a molecular basis for any further investigation on the evolution of these two ecologically and agriculturally important butterflies.

## Data availability statement

The datasets presented in this study can be found in NCBI online database. Accession numbers of sequences and BioProject can be found in this article/Supplementary material.

## Author contributions

QW collected and analyzed the data, with the support of AH, and wrote the initial draft of the manuscript. All authors contributed to the final revision and involved in the initial planning and design of the study.
