# Supplementary material for: Sympatric Pieris butterfly species exhibit a high conservation of chemoreceptors
Source: Front Cell Neurosci. 2023 May 11;17:1155405. doi: 10.3389/fncel.2023.1155405 (PMC10210156; doi:10.3389/fncel.2023.1155405)

**Conserved motif sequences of ORs**

1.
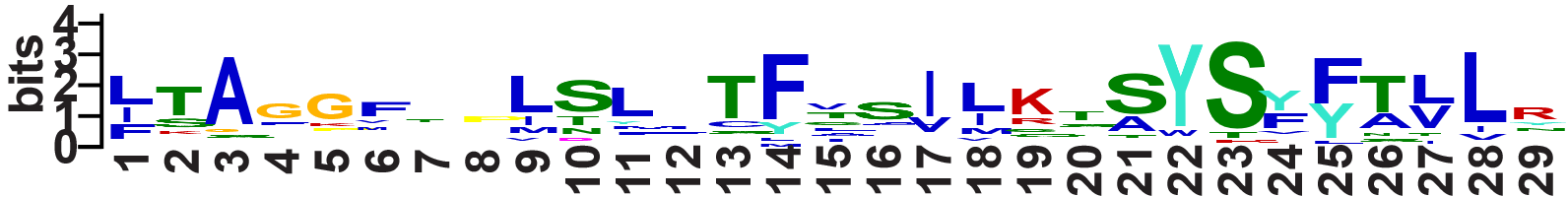


2.
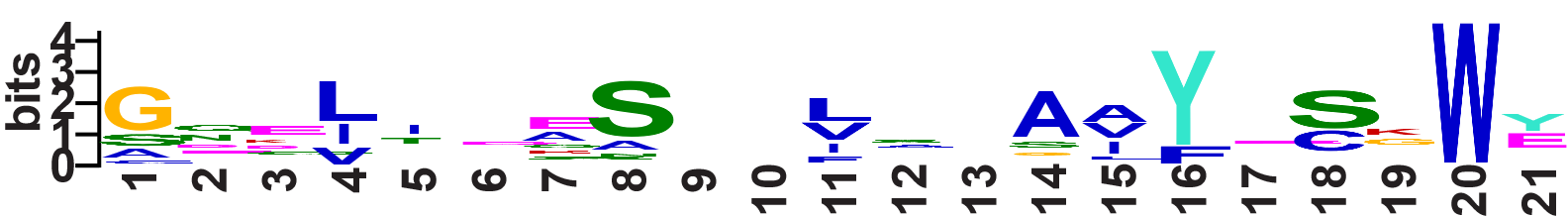


3.
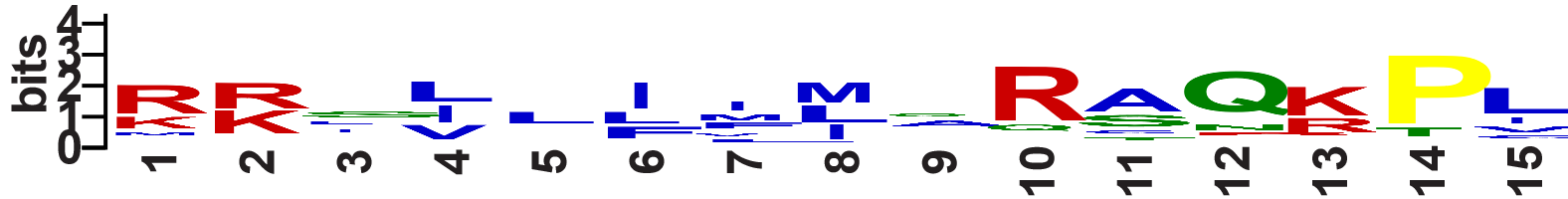


4.
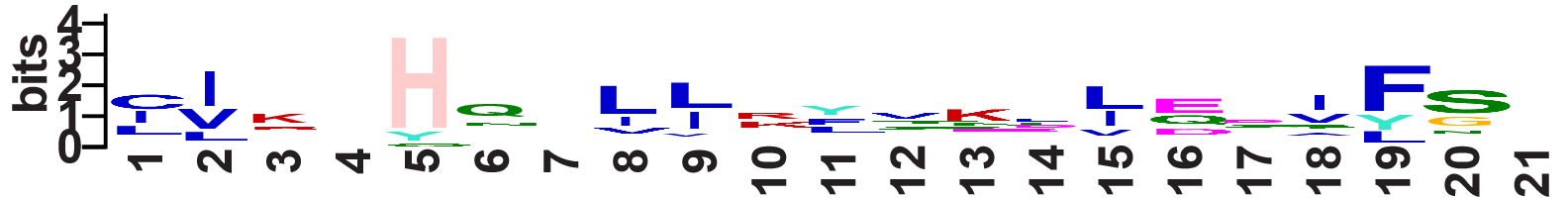


5.
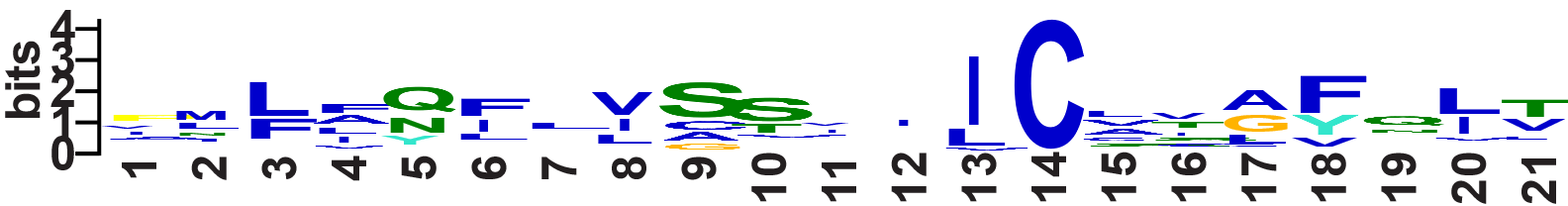


6.
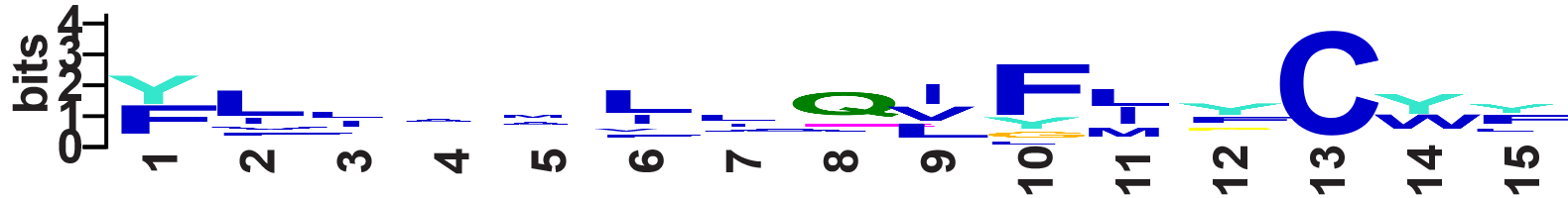


7.
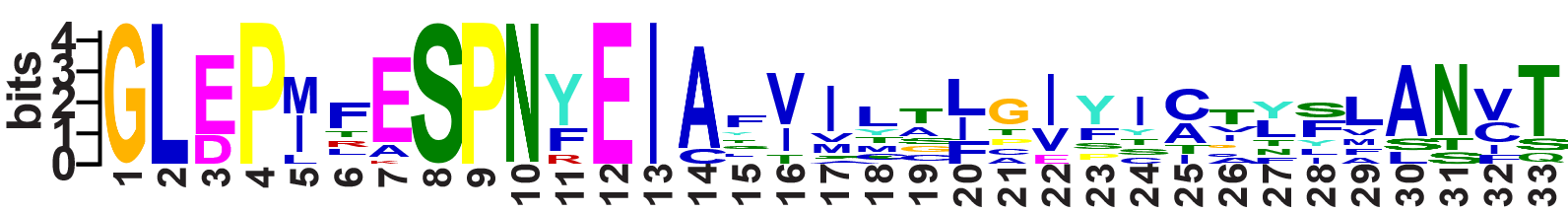


8.
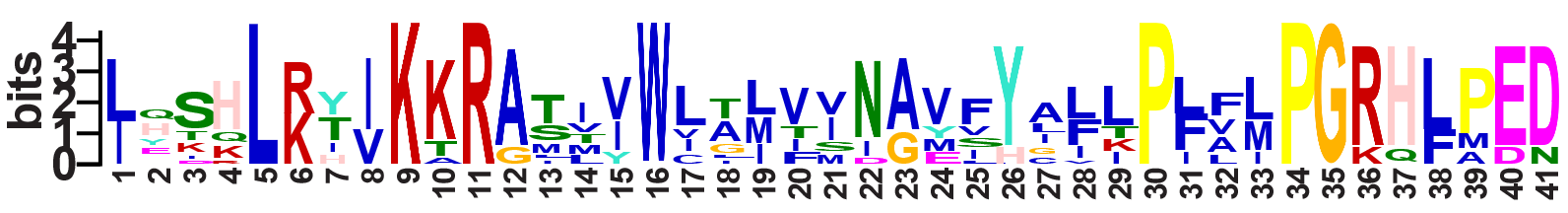


9.
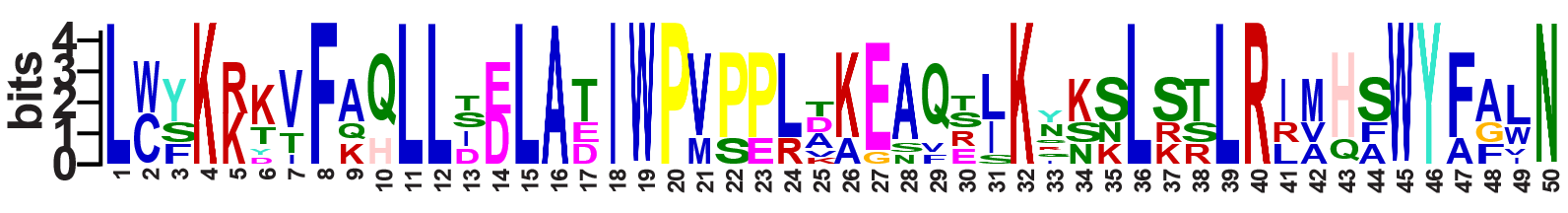


10.
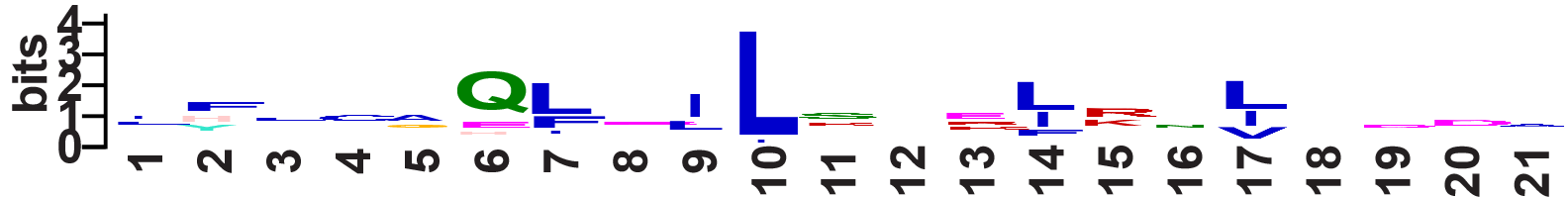


**Conserved motif seqeuences of IRs**

1.
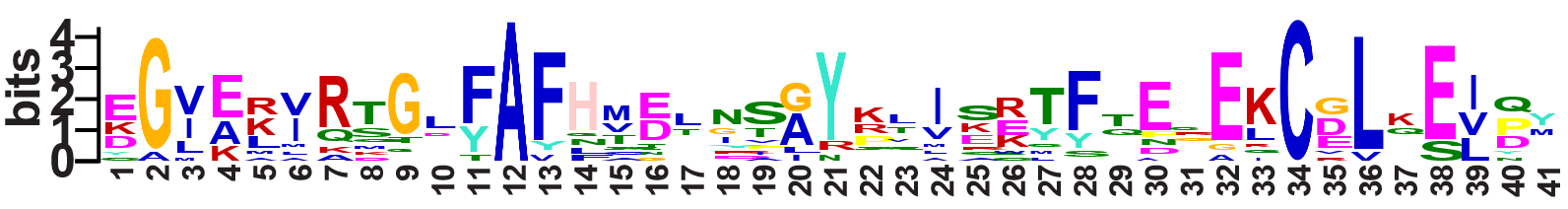


2.
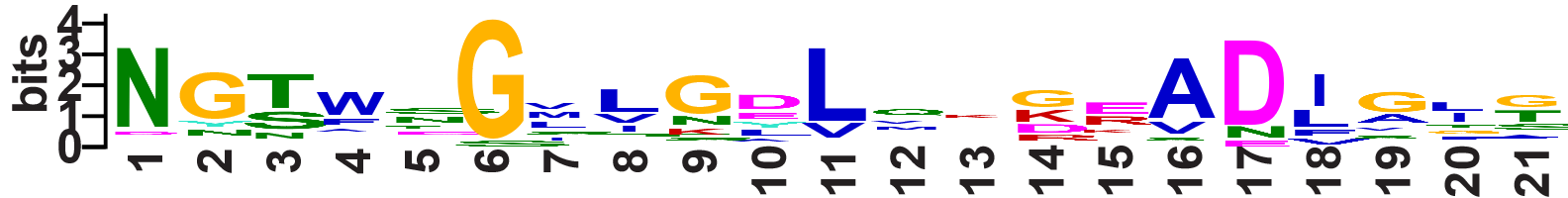


3.
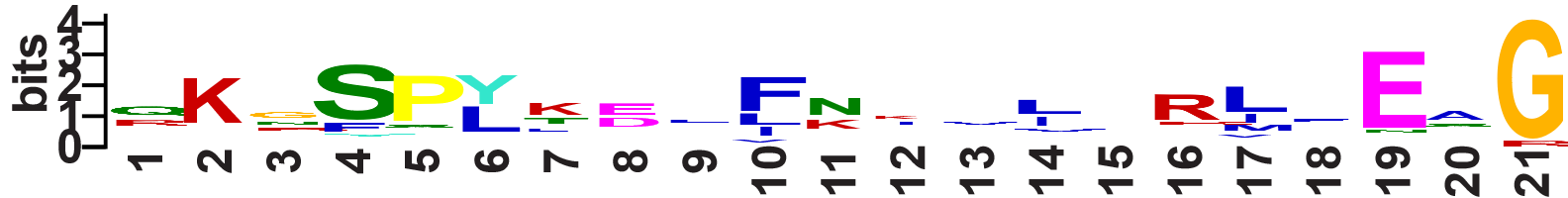


4.
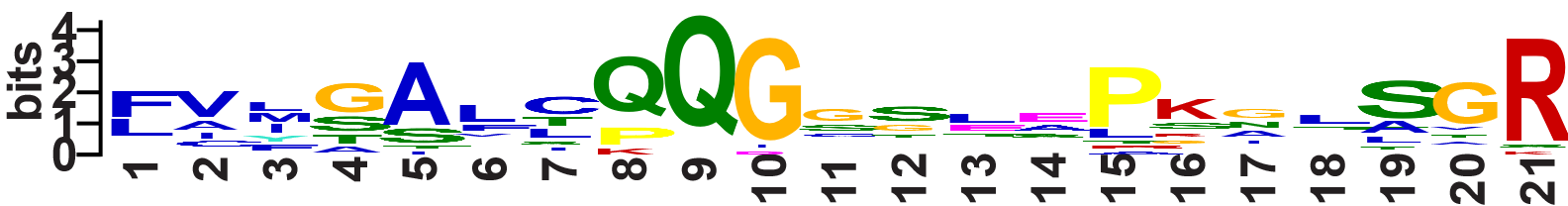


5.
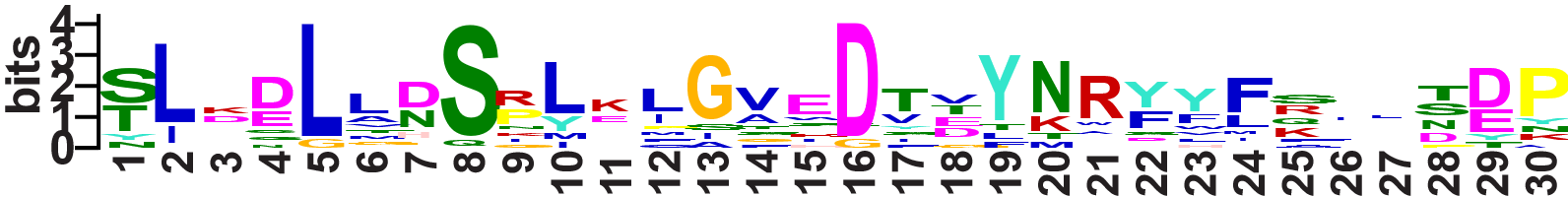


6.
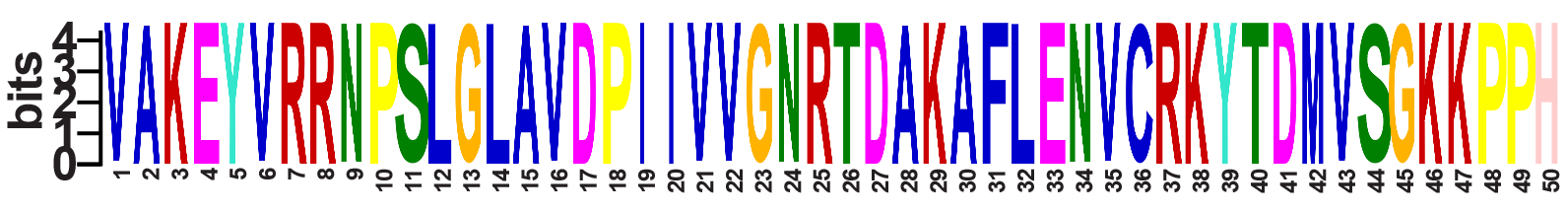


7.
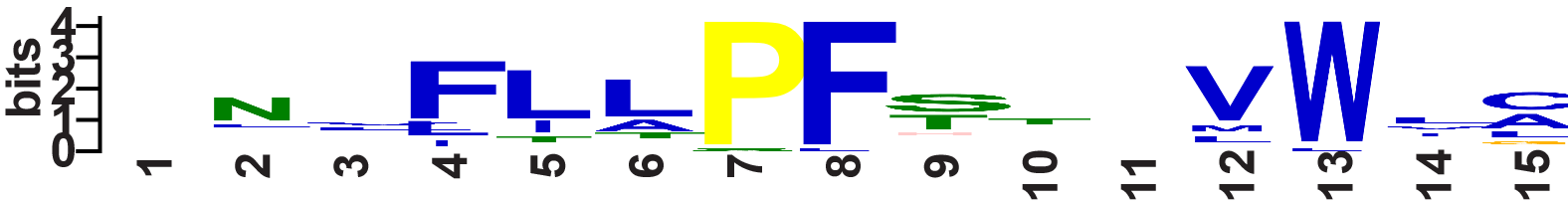


8.
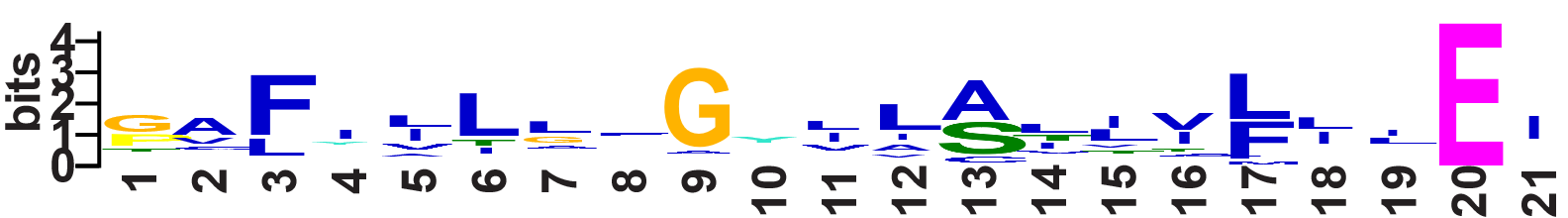


9.
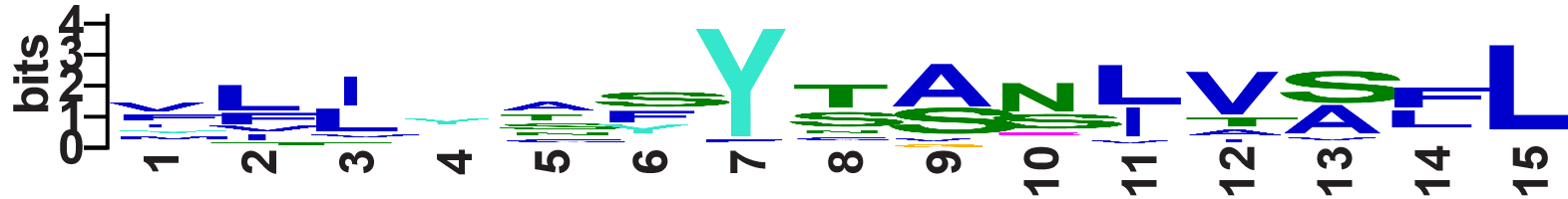


10.
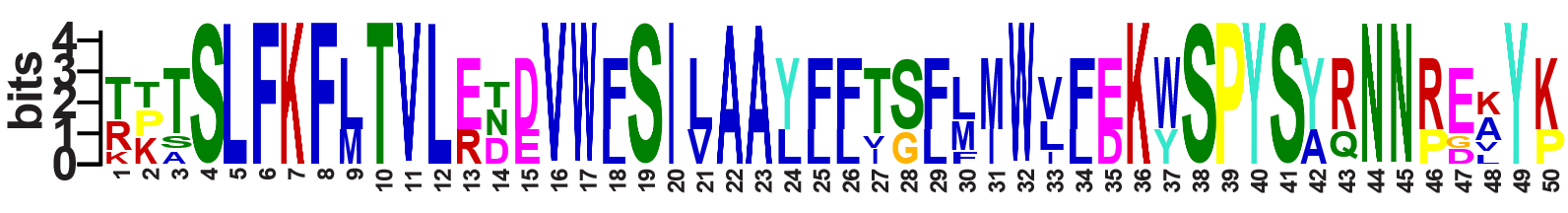


**Conserved motif seqeuences of GRs**

1.
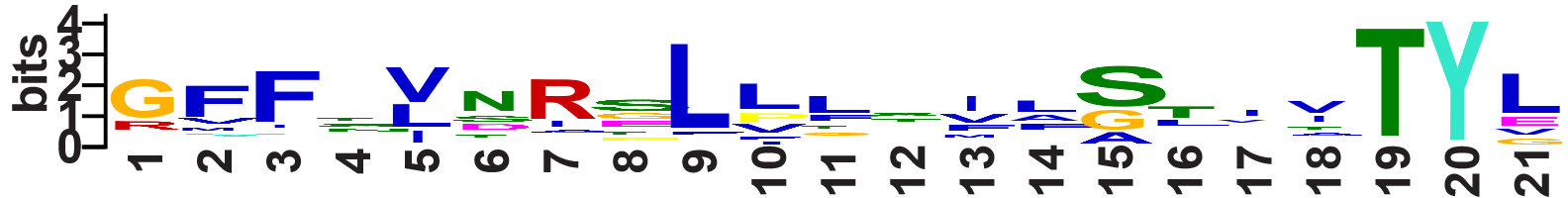


2.
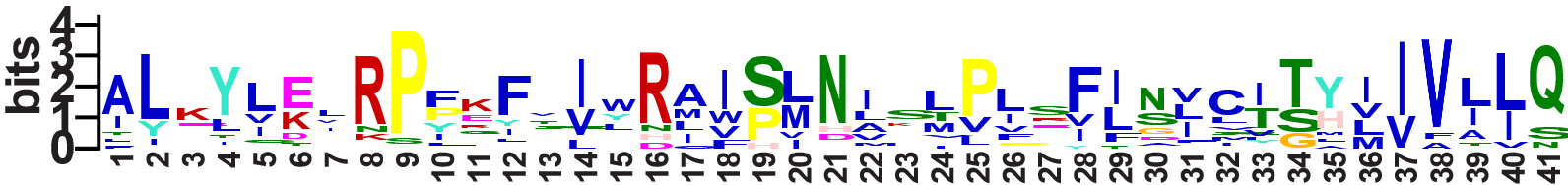


3.
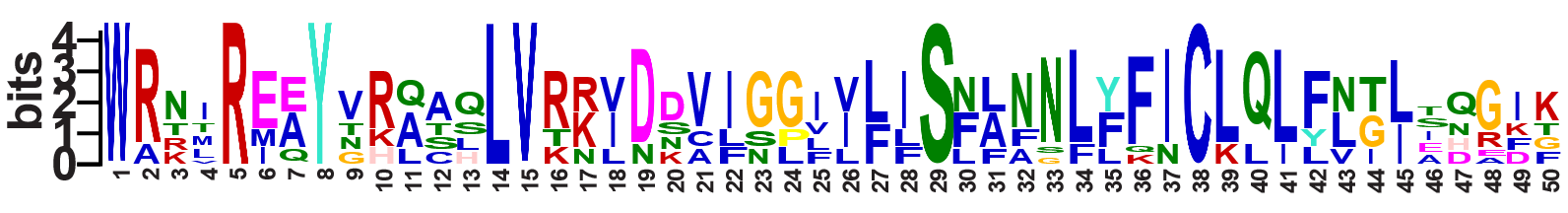


4.
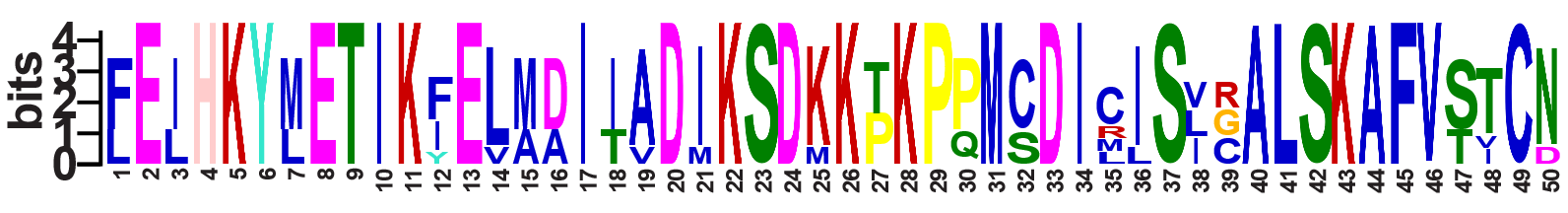


5.
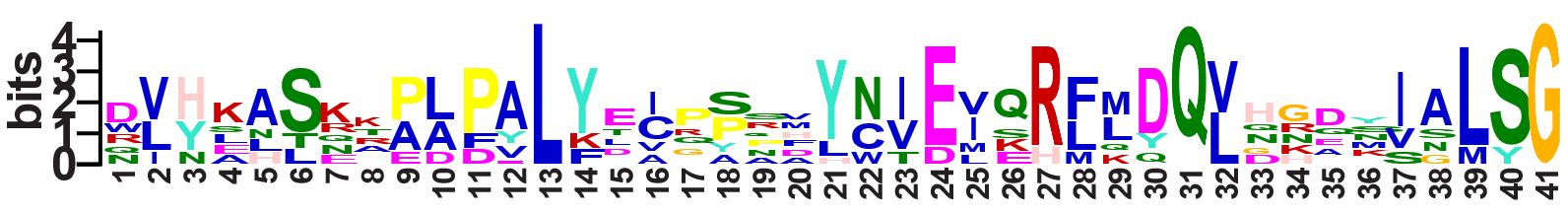


6.
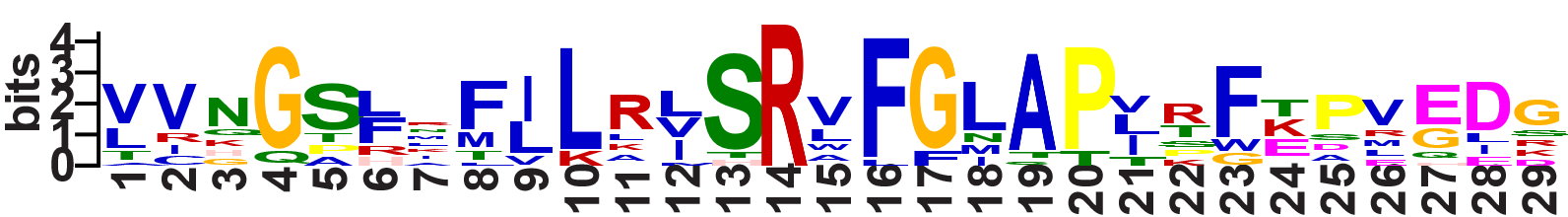


7.
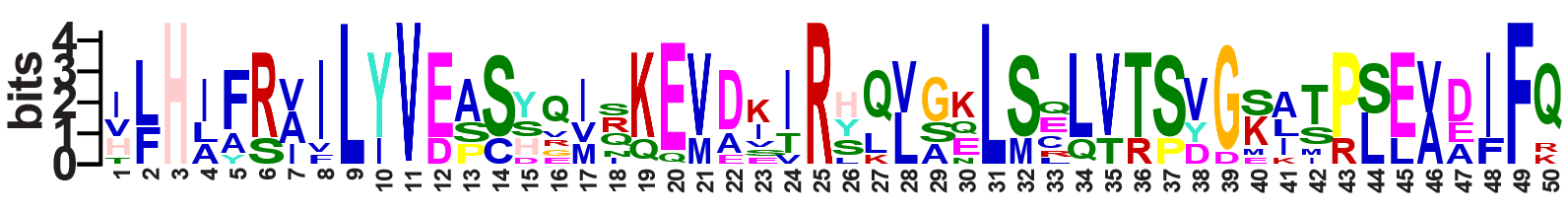


8.
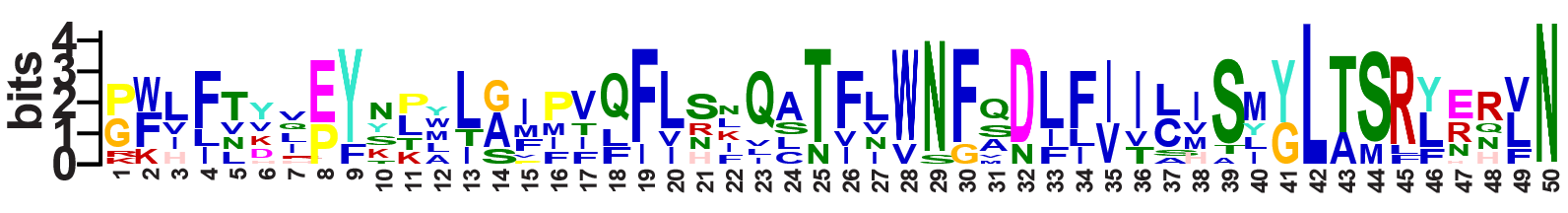


9.
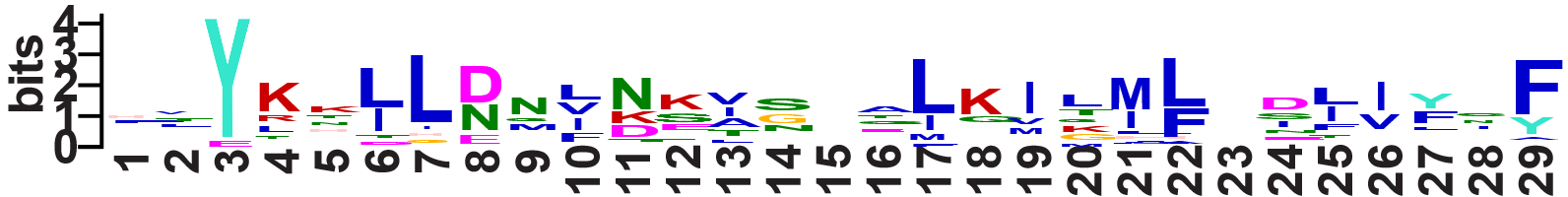


10.
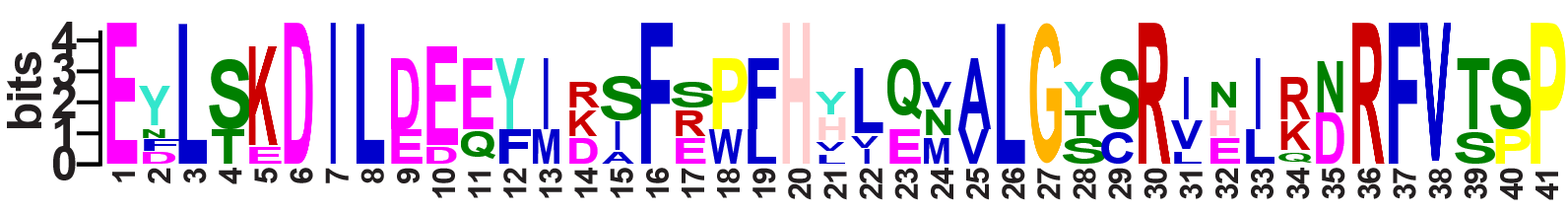

Supplement: Supplementary file 4 [file Data_Sheet_1.ZIP › S4_conserved motifs/S4_Conserved motif sequences.docx]
