# Supplementary material for: Sympatric Pieris butterfly species exhibit a high conservation of chemoreceptors
Source: Front Cell Neurosci. 2023 May 11;17:1155405. doi: 10.3389/fncel.2023.1155405 (PMC10210156; doi:10.3389/fncel.2023.1155405)
Supplement: Supplementary file 5 [file Data_Sheet_2.ZIP › S5_Transmembrane domain prediction/Chemoreceptor_TMHMM.html]

TMHMM result


## TMHMM result

---

```
# PbraOR55 Length: 417
# PbraOR55 Number of predicted TMHs:  5
# PbraOR55 Exp number of AAs in TMHs: 106.1281
# PbraOR55 Exp number, first 60 AAs:  9.02998
# PbraOR55 Total prob of N-in:        0.27553
PbraOR55	TMHMM2.0	outside	     1    50
PbraOR55	TMHMM2.0	TMhelix	    51    73
PbraOR55	TMHMM2.0	inside	    74   144
PbraOR55	TMHMM2.0	TMhelix	   145   164
PbraOR55	TMHMM2.0	outside	   165   183
PbraOR55	TMHMM2.0	TMhelix	   184   206
PbraOR55	TMHMM2.0	inside	   207   284
PbraOR55	TMHMM2.0	TMhelix	   285   307
PbraOR55	TMHMM2.0	outside	   308   316
PbraOR55	TMHMM2.0	TMhelix	   317   334
PbraOR55	TMHMM2.0	inside	   335   417
```

# plot in postscript,
script for making the plot in gnuplot,
data for plot

---

```
# PbraOR54 Length: 416
# PbraOR54 Number of predicted TMHs:  4
# PbraOR54 Exp number of AAs in TMHs: 96.85112
# PbraOR54 Exp number, first 60 AAs:  5.29418
# PbraOR54 Total prob of N-in:        0.72401
PbraOR54	TMHMM2.0	inside	     1   143
PbraOR54	TMHMM2.0	TMhelix	   144   163
PbraOR54	TMHMM2.0	outside	   164   182
PbraOR54	TMHMM2.0	TMhelix	   183   205
PbraOR54	TMHMM2.0	inside	   206   283
PbraOR54	TMHMM2.0	TMhelix	   284   306
PbraOR54	TMHMM2.0	outside	   307   315
PbraOR54	TMHMM2.0	TMhelix	   316   333
PbraOR54	TMHMM2.0	inside	   334   416
```

# plot in postscript,
script for making the plot in gnuplot,
data for plot

---

```
# PbraOR36 Length: 419
# PbraOR36 Number of predicted TMHs:  6
# PbraOR36 Exp number of AAs in TMHs: 126.00808
# PbraOR36 Exp number, first 60 AAs:  12.22172
# PbraOR36 Total prob of N-in:        0.99748
# PbraOR36 POSSIBLE N-term signal sequence
PbraOR36	TMHMM2.0	inside	     1    46
PbraOR36	TMHMM2.0	TMhelix	    47    69
PbraOR36	TMHMM2.0	outside	    70    83
PbraOR36	TMHMM2.0	TMhelix	    84   106
PbraOR36	TMHMM2.0	inside	   107   147
PbraOR36	TMHMM2.0	TMhelix	   148   170
PbraOR36	TMHMM2.0	outside	   171   196
PbraOR36	TMHMM2.0	TMhelix	   197   219
PbraOR36	TMHMM2.0	inside	   220   292
PbraOR36	TMHMM2.0	TMhelix	   293   315
PbraOR36	TMHMM2.0	outside	   316   324
PbraOR36	TMHMM2.0	TMhelix	   325   347
PbraOR36	TMHMM2.0	inside	   348   419
```

# plot in postscript,
script for making the plot in gnuplot,
data for plot

---

```
# PbraOR30 Length: 397
# PbraOR30 Number of predicted TMHs:  6
# PbraOR30 Exp number of AAs in TMHs: 130.92401
# PbraOR30 Exp number, first 60 AAs:  21.93279
# PbraOR30 Total prob of N-in:        0.94597
# PbraOR30 POSSIBLE N-term signal sequence
PbraOR30	TMHMM2.0	inside	     1    34
PbraOR30	TMHMM2.0	TMhelix	    35    57
PbraOR30	TMHMM2.0	outside	    58    71
PbraOR30	TMHMM2.0	TMhelix	    72    89
PbraOR30	TMHMM2.0	inside	    90   133
PbraOR30	TMHMM2.0	TMhelix	   134   156
PbraOR30	TMHMM2.0	outside	   157   199
PbraOR30	TMHMM2.0	TMhelix	   200   222
PbraOR30	TMHMM2.0	inside	   223   271
PbraOR30	TMHMM2.0	TMhelix	   272   289
PbraOR30	TMHMM2.0	outside	   290   303
PbraOR30	TMHMM2.0	TMhelix	   304   326
PbraOR30	TMHMM2.0	inside	   327   397
```

# plot in postscript,
script for making the plot in gnuplot,
data for plot

---

```
# PbraOR29 Length: 394
# PbraOR29 Number of predicted TMHs:  5
# PbraOR29 Exp number of AAs in TMHs: 126.96066
# PbraOR29 Exp number, first 60 AAs:  21.11204
# PbraOR29 Total prob of N-in:        0.95688
# PbraOR29 POSSIBLE N-term signal sequence
PbraOR29	TMHMM2.0	inside	     1    38
PbraOR29	TMHMM2.0	TMhelix	    39    58
PbraOR29	TMHMM2.0	outside	    59    72
PbraOR29	TMHMM2.0	TMhelix	    73    90
PbraOR29	TMHMM2.0	inside	    91   133
PbraOR29	TMHMM2.0	TMhelix	   134   156
PbraOR29	TMHMM2.0	outside	   157   185
PbraOR29	TMHMM2.0	TMhelix	   186   208
PbraOR29	TMHMM2.0	inside	   209   296
PbraOR29	TMHMM2.0	TMhelix	   297   319
PbraOR29	TMHMM2.0	outside	   320   394
```

# plot in postscript,
script for making the plot in gnuplot,
data for plot

---

```
# PbraOR32 Length: 390
# PbraOR32 Number of predicted TMHs:  6
# PbraOR32 Exp number of AAs in TMHs: 142.49067
# PbraOR32 Exp number, first 60 AAs:  29.71031
# PbraOR32 Total prob of N-in:        0.62000
# PbraOR32 POSSIBLE N-term signal sequence
PbraOR32	TMHMM2.0	inside	     1    34
PbraOR32	TMHMM2.0	TMhelix	    35    57
PbraOR32	TMHMM2.0	outside	    58    60
PbraOR32	TMHMM2.0	TMhelix	    61    83
PbraOR32	TMHMM2.0	inside	    84   132
PbraOR32	TMHMM2.0	TMhelix	   133   155
PbraOR32	TMHMM2.0	outside	   156   187
PbraOR32	TMHMM2.0	TMhelix	   188   207
PbraOR32	TMHMM2.0	inside	   208   266
PbraOR32	TMHMM2.0	TMhelix	   267   289
PbraOR32	TMHMM2.0	outside	   290   298
PbraOR32	TMHMM2.0	TMhelix	   299   321
PbraOR32	TMHMM2.0	inside	   322   390
```

# plot in postscript,
script for making the plot in gnuplot,
data for plot

---

```
# PbraOR33 Length: 366
# PbraOR33 Number of predicted TMHs:  6
# PbraOR33 Exp number of AAs in TMHs: 145.89301
# PbraOR33 Exp number, first 60 AAs:  33.99887
# PbraOR33 Total prob of N-in:        0.99892
# PbraOR33 POSSIBLE N-term signal sequence
PbraOR33	TMHMM2.0	inside	     1    12
PbraOR33	TMHMM2.0	TMhelix	    13    30
PbraOR33	TMHMM2.0	outside	    31    44
PbraOR33	TMHMM2.0	TMhelix	    45    67
PbraOR33	TMHMM2.0	inside	    68   110
PbraOR33	TMHMM2.0	TMhelix	   111   133
PbraOR33	TMHMM2.0	outside	   134   167
PbraOR33	TMHMM2.0	TMhelix	   168   190
PbraOR33	TMHMM2.0	inside	   191   241
PbraOR33	TMHMM2.0	TMhelix	   242   261
PbraOR33	TMHMM2.0	outside	   262   270
PbraOR33	TMHMM2.0	TMhelix	   271   293
PbraOR33	TMHMM2.0	inside	   294   366
```

# plot in postscript,
script for making the plot in gnuplot,
data for plot

---

```
# PbraOR11 Length: 394
# PbraOR11 Number of predicted TMHs:  6
# PbraOR11 Exp number of AAs in TMHs: 132.65842
# PbraOR11 Exp number, first 60 AAs:  21.29734
# PbraOR11 Total prob of N-in:        0.93196
# PbraOR11 POSSIBLE N-term signal sequence
PbraOR11	TMHMM2.0	inside	     1    40
PbraOR11	TMHMM2.0	TMhelix	    41    63
PbraOR11	TMHMM2.0	outside	    64    72
PbraOR11	TMHMM2.0	TMhelix	    73    95
PbraOR11	TMHMM2.0	inside	    96   139
PbraOR11	TMHMM2.0	TMhelix	   140   162
PbraOR11	TMHMM2.0	outside	   163   198
PbraOR11	TMHMM2.0	TMhelix	   199   221
PbraOR11	TMHMM2.0	inside	   222   270
PbraOR11	TMHMM2.0	TMhelix	   271   293
PbraOR11	TMHMM2.0	outside	   294   302
PbraOR11	TMHMM2.0	TMhelix	   303   322
PbraOR11	TMHMM2.0	inside	   323   394
```

# plot in postscript,
script for making the plot in gnuplot,
data for plot

---

```
# PbraOR56 Length: 421
# PbraOR56 Number of predicted TMHs:  6
# PbraOR56 Exp number of AAs in TMHs: 144.27547
# PbraOR56 Exp number, first 60 AAs:  27.62368
# PbraOR56 Total prob of N-in:        0.67343
# PbraOR56 POSSIBLE N-term signal sequence
PbraOR56	TMHMM2.0	inside	     1    33
PbraOR56	TMHMM2.0	TMhelix	    34    56
PbraOR56	TMHMM2.0	outside	    57    70
PbraOR56	TMHMM2.0	TMhelix	    71    93
PbraOR56	TMHMM2.0	inside	    94   127
PbraOR56	TMHMM2.0	TMhelix	   128   150
PbraOR56	TMHMM2.0	outside	   151   195
PbraOR56	TMHMM2.0	TMhelix	   196   218
PbraOR56	TMHMM2.0	inside	   219   291
PbraOR56	TMHMM2.0	TMhelix	   292   314
PbraOR56	TMHMM2.0	outside	   315   323
PbraOR56	TMHMM2.0	TMhelix	   324   346
PbraOR56	TMHMM2.0	inside	   347   421
```

# plot in postscript,
script for making the plot in gnuplot,
data for plot

---

```
# PbraOR34 Length: 372
# PbraOR34 Number of predicted TMHs:  6
# PbraOR34 Exp number of AAs in TMHs: 123.97281
# PbraOR34 Exp number, first 60 AAs:  21.18721
# PbraOR34 Total prob of N-in:        0.95450
# PbraOR34 POSSIBLE N-term signal sequence
PbraOR34	TMHMM2.0	inside	     1    36
PbraOR34	TMHMM2.0	TMhelix	    37    59
PbraOR34	TMHMM2.0	outside	    60    73
PbraOR34	TMHMM2.0	TMhelix	    74    89
PbraOR34	TMHMM2.0	inside	    90   118
PbraOR34	TMHMM2.0	TMhelix	   119   141
PbraOR34	TMHMM2.0	outside	   142   160
PbraOR34	TMHMM2.0	TMhelix	   161   183
PbraOR34	TMHMM2.0	inside	   184   246
PbraOR34	TMHMM2.0	TMhelix	   247   269
PbraOR34	TMHMM2.0	outside	   270   273
PbraOR34	TMHMM2.0	TMhelix	   274   296
PbraOR34	TMHMM2.0	inside	   297   372
```

# plot in postscript,
script for making the plot in gnuplot,
data for plot

---

```
# PbraOR14 Length: 393
# PbraOR14 Number of predicted TMHs:  6
# PbraOR14 Exp number of AAs in TMHs: 130.09451
# PbraOR14 Exp number, first 60 AAs:  22.57624
# PbraOR14 Total prob of N-in:        0.97116
# PbraOR14 POSSIBLE N-term signal sequence
PbraOR14	TMHMM2.0	inside	     1    37
PbraOR14	TMHMM2.0	TMhelix	    38    60
PbraOR14	TMHMM2.0	outside	    61    74
PbraOR14	TMHMM2.0	TMhelix	    75    94
PbraOR14	TMHMM2.0	inside	    95   127
PbraOR14	TMHMM2.0	TMhelix	   128   150
PbraOR14	TMHMM2.0	outside	   151   185
PbraOR14	TMHMM2.0	TMhelix	   186   208
PbraOR14	TMHMM2.0	inside	   209   266
PbraOR14	TMHMM2.0	TMhelix	   267   289
PbraOR14	TMHMM2.0	outside	   290   293
PbraOR14	TMHMM2.0	TMhelix	   294   316
PbraOR14	TMHMM2.0	inside	   317   393
```

# plot in postscript,
script for making the plot in gnuplot,
data for plot

---

```
# PbraOR58 Length: 397
# PbraOR58 Number of predicted TMHs:  7
# PbraOR58 Exp number of AAs in TMHs: 149.32797
# PbraOR58 Exp number, first 60 AAs:  20.12721
# PbraOR58 Total prob of N-in:        0.99720
# PbraOR58 POSSIBLE N-term signal sequence
PbraOR58	TMHMM2.0	inside	     1    39
PbraOR58	TMHMM2.0	TMhelix	    40    62
PbraOR58	TMHMM2.0	outside	    63    71
PbraOR58	TMHMM2.0	TMhelix	    72    91
PbraOR58	TMHMM2.0	inside	    92   126
PbraOR58	TMHMM2.0	TMhelix	   127   146
PbraOR58	TMHMM2.0	outside	   147   181
PbraOR58	TMHMM2.0	TMhelix	   182   204
PbraOR58	TMHMM2.0	inside	   205   261
PbraOR58	TMHMM2.0	TMhelix	   262   284
PbraOR58	TMHMM2.0	outside	   285   293
PbraOR58	TMHMM2.0	TMhelix	   294   316
PbraOR58	TMHMM2.0	inside	   317   370
PbraOR58	TMHMM2.0	TMhelix	   371   393
PbraOR58	TMHMM2.0	outside	   394   397
```

# plot in postscript,
script for making the plot in gnuplot,
data for plot

---

```
# PbraOR57 Length: 397
# PbraOR57 Number of predicted TMHs:  8
# PbraOR57 Exp number of AAs in TMHs: 161.40239
# PbraOR57 Exp number, first 60 AAs:  21.44652
# PbraOR57 Total prob of N-in:        0.96532
# PbraOR57 POSSIBLE N-term signal sequence
PbraOR57	TMHMM2.0	inside	     1    39
PbraOR57	TMHMM2.0	TMhelix	    40    62
PbraOR57	TMHMM2.0	outside	    63    71
PbraOR57	TMHMM2.0	TMhelix	    72    91
PbraOR57	TMHMM2.0	inside	    92   124
PbraOR57	TMHMM2.0	TMhelix	   125   143
PbraOR57	TMHMM2.0	outside	   144   152
PbraOR57	TMHMM2.0	TMhelix	   153   175
PbraOR57	TMHMM2.0	inside	   176   181
PbraOR57	TMHMM2.0	TMhelix	   182   204
PbraOR57	TMHMM2.0	outside	   205   260
PbraOR57	TMHMM2.0	TMhelix	   261   283
PbraOR57	TMHMM2.0	inside	   284   295
PbraOR57	TMHMM2.0	TMhelix	   296   318
PbraOR57	TMHMM2.0	outside	   319   370
PbraOR57	TMHMM2.0	TMhelix	   371   393
PbraOR57	TMHMM2.0	inside	   394   397
```

# plot in postscript,
script for making the plot in gnuplot,
data for plot

---

```
# PbraOR17 Length: 391
# PbraOR17 Number of predicted TMHs:  6
# PbraOR17 Exp number of AAs in TMHs: 140.32881
# PbraOR17 Exp number, first 60 AAs:  16.02229
# PbraOR17 Total prob of N-in:        0.99936
# PbraOR17 POSSIBLE N-term signal sequence
PbraOR17	TMHMM2.0	inside	     1    42
PbraOR17	TMHMM2.0	TMhelix	    43    65
PbraOR17	TMHMM2.0	outside	    66    79
PbraOR17	TMHMM2.0	TMhelix	    80    99
PbraOR17	TMHMM2.0	inside	   100   127
PbraOR17	TMHMM2.0	TMhelix	   128   150
PbraOR17	TMHMM2.0	outside	   151   186
PbraOR17	TMHMM2.0	TMhelix	   187   209
PbraOR17	TMHMM2.0	inside	   210   255
PbraOR17	TMHMM2.0	TMhelix	   256   278
PbraOR17	TMHMM2.0	outside	   279   287
PbraOR17	TMHMM2.0	TMhelix	   288   310
PbraOR17	TMHMM2.0	inside	   311   391
```

# plot in postscript,
script for making the plot in gnuplot,
data for plot

---

```
# PbraOR3 Length: 405
# PbraOR3 Number of predicted TMHs:  5
# PbraOR3 Exp number of AAs in TMHs: 130.71551
# PbraOR3 Exp number, first 60 AAs:  28.99301
# PbraOR3 Total prob of N-in:        0.37242
# PbraOR3 POSSIBLE N-term signal sequence
PbraOR3	TMHMM2.0	inside	     1    44
PbraOR3	TMHMM2.0	TMhelix	    45    67
PbraOR3	TMHMM2.0	outside	    68    76
PbraOR3	TMHMM2.0	TMhelix	    77    99
PbraOR3	TMHMM2.0	inside	   100   137
PbraOR3	TMHMM2.0	TMhelix	   138   160
PbraOR3	TMHMM2.0	outside	   161   194
PbraOR3	TMHMM2.0	TMhelix	   195   217
PbraOR3	TMHMM2.0	inside	   218   304
PbraOR3	TMHMM2.0	TMhelix	   305   327
PbraOR3	TMHMM2.0	outside	   328   405
```

# plot in postscript,
script for making the plot in gnuplot,
data for plot

---

```
# PbraOR37 Length: 410
# PbraOR37 Number of predicted TMHs:  7
# PbraOR37 Exp number of AAs in TMHs: 159.74688
# PbraOR37 Exp number, first 60 AAs:  22.6983
# PbraOR37 Total prob of N-in:        0.97013
# PbraOR37 POSSIBLE N-term signal sequence
PbraOR37	TMHMM2.0	inside	     1    30
PbraOR37	TMHMM2.0	TMhelix	    31    53
PbraOR37	TMHMM2.0	outside	    54    62
PbraOR37	TMHMM2.0	TMhelix	    63    85
PbraOR37	TMHMM2.0	inside	    86   144
PbraOR37	TMHMM2.0	TMhelix	   145   167
PbraOR37	TMHMM2.0	outside	   168   190
PbraOR37	TMHMM2.0	TMhelix	   191   213
PbraOR37	TMHMM2.0	inside	   214   284
PbraOR37	TMHMM2.0	TMhelix	   285   307
PbraOR37	TMHMM2.0	outside	   308   316
PbraOR37	TMHMM2.0	TMhelix	   317   335
PbraOR37	TMHMM2.0	inside	   336   376
PbraOR37	TMHMM2.0	TMhelix	   377   394
PbraOR37	TMHMM2.0	outside	   395   410
```

# plot in postscript,
script for making the plot in gnuplot,
data for plot

---

```
# PbraOR20 Length: 392
# PbraOR20 Number of predicted TMHs:  6
# PbraOR20 Exp number of AAs in TMHs: 128.02693
# PbraOR20 Exp number, first 60 AAs:  8.8161
# PbraOR20 Total prob of N-in:        0.99177
PbraOR20	TMHMM2.0	inside	     1    49
PbraOR20	TMHMM2.0	TMhelix	    50    69
PbraOR20	TMHMM2.0	outside	    70    83
PbraOR20	TMHMM2.0	TMhelix	    84   102
PbraOR20	TMHMM2.0	inside	   103   140
PbraOR20	TMHMM2.0	TMhelix	   141   163
PbraOR20	TMHMM2.0	outside	   164   203
PbraOR20	TMHMM2.0	TMhelix	   204   226
PbraOR20	TMHMM2.0	inside	   227   268
PbraOR20	TMHMM2.0	TMhelix	   269   291
PbraOR20	TMHMM2.0	outside	   292   294
PbraOR20	TMHMM2.0	TMhelix	   295   317
PbraOR20	TMHMM2.0	inside	   318   392
```

# plot in postscript,
script for making the plot in gnuplot,
data for plot

---

```
# PbraOR1 Length: 389
# PbraOR1 Number of predicted TMHs:  6
# PbraOR1 Exp number of AAs in TMHs: 145.62589
# PbraOR1 Exp number, first 60 AAs:  37.64634
# PbraOR1 Total prob of N-in:        0.98546
# PbraOR1 POSSIBLE N-term signal sequence
PbraOR1	TMHMM2.0	inside	     1    11
PbraOR1	TMHMM2.0	TMhelix	    12    34
PbraOR1	TMHMM2.0	outside	    35    43
PbraOR1	TMHMM2.0	TMhelix	    44    66
PbraOR1	TMHMM2.0	inside	    67   101
PbraOR1	TMHMM2.0	TMhelix	   102   124
PbraOR1	TMHMM2.0	outside	   125   169
PbraOR1	TMHMM2.0	TMhelix	   170   192
PbraOR1	TMHMM2.0	inside	   193   258
PbraOR1	TMHMM2.0	TMhelix	   259   281
PbraOR1	TMHMM2.0	outside	   282   295
PbraOR1	TMHMM2.0	TMhelix	   296   318
PbraOR1	TMHMM2.0	inside	   319   389
```

# plot in postscript,
script for making the plot in gnuplot,
data for plot

---

```
# PbraOR13 Length: 387
# PbraOR13 Number of predicted TMHs:  6
# PbraOR13 Exp number of AAs in TMHs: 141.64037
# PbraOR13 Exp number, first 60 AAs:  16.15285
# PbraOR13 Total prob of N-in:        0.49392
# PbraOR13 POSSIBLE N-term signal sequence
PbraOR13	TMHMM2.0	inside	     1    44
PbraOR13	TMHMM2.0	TMhelix	    45    67
PbraOR13	TMHMM2.0	outside	    68    81
PbraOR13	TMHMM2.0	TMhelix	    82    99
PbraOR13	TMHMM2.0	inside	   100   187
PbraOR13	TMHMM2.0	TMhelix	   188   210
PbraOR13	TMHMM2.0	outside	   211   266
PbraOR13	TMHMM2.0	TMhelix	   267   289
PbraOR13	TMHMM2.0	inside	   290   293
PbraOR13	TMHMM2.0	TMhelix	   294   313
PbraOR13	TMHMM2.0	outside	   314   362
PbraOR13	TMHMM2.0	TMhelix	   363   385
PbraOR13	TMHMM2.0	inside	   386   387
```

# plot in postscript,
script for making the plot in gnuplot,
data for plot

---

```
# PbraOR12 Length: 388
# PbraOR12 Number of predicted TMHs:  5
# PbraOR12 Exp number of AAs in TMHs: 135.35782
# PbraOR12 Exp number, first 60 AAs:  20.11386
# PbraOR12 Total prob of N-in:        0.75223
# PbraOR12 POSSIBLE N-term signal sequence
PbraOR12	TMHMM2.0	inside	     1    46
PbraOR12	TMHMM2.0	TMhelix	    47    69
PbraOR12	TMHMM2.0	outside	    70   139
PbraOR12	TMHMM2.0	TMhelix	   140   157
PbraOR12	TMHMM2.0	inside	   158   267
PbraOR12	TMHMM2.0	TMhelix	   268   290
PbraOR12	TMHMM2.0	outside	   291   293
PbraOR12	TMHMM2.0	TMhelix	   294   316
PbraOR12	TMHMM2.0	inside	   317   356
PbraOR12	TMHMM2.0	TMhelix	   357   379
PbraOR12	TMHMM2.0	outside	   380   388
```

# plot in postscript,
script for making the plot in gnuplot,
data for plot

---

```
# PbraOR24 Length: 408
# PbraOR24 Number of predicted TMHs:  6
# PbraOR24 Exp number of AAs in TMHs: 141.29265
# PbraOR24 Exp number, first 60 AAs:  23.65033
# PbraOR24 Total prob of N-in:        0.64613
# PbraOR24 POSSIBLE N-term signal sequence
PbraOR24	TMHMM2.0	inside	     1    43
PbraOR24	TMHMM2.0	TMhelix	    44    66
PbraOR24	TMHMM2.0	outside	    67    80
PbraOR24	TMHMM2.0	TMhelix	    81   103
PbraOR24	TMHMM2.0	inside	   104   142
PbraOR24	TMHMM2.0	TMhelix	   143   165
PbraOR24	TMHMM2.0	outside	   166   196
PbraOR24	TMHMM2.0	TMhelix	   197   219
PbraOR24	TMHMM2.0	inside	   220   286
PbraOR24	TMHMM2.0	TMhelix	   287   309
PbraOR24	TMHMM2.0	outside	   310   374
PbraOR24	TMHMM2.0	TMhelix	   375   397
PbraOR24	TMHMM2.0	inside	   398   408
```

# plot in postscript,
script for making the plot in gnuplot,
data for plot

---

```
# PbraOR25 Length: 411
# PbraOR25 Number of predicted TMHs:  5
# PbraOR25 Exp number of AAs in TMHs: 132.14102
# PbraOR25 Exp number, first 60 AAs:  8.73993
# PbraOR25 Total prob of N-in:        0.99206
PbraOR25	TMHMM2.0	inside	     1    53
PbraOR25	TMHMM2.0	TMhelix	    54    76
PbraOR25	TMHMM2.0	outside	    77    85
PbraOR25	TMHMM2.0	TMhelix	    86   108
PbraOR25	TMHMM2.0	inside	   109   150
PbraOR25	TMHMM2.0	TMhelix	   151   173
PbraOR25	TMHMM2.0	outside	   174   197
PbraOR25	TMHMM2.0	TMhelix	   198   220
PbraOR25	TMHMM2.0	inside	   221   289
PbraOR25	TMHMM2.0	TMhelix	   290   312
PbraOR25	TMHMM2.0	outside	   313   411
```

# plot in postscript,
script for making the plot in gnuplot,
data for plot

---

```
# PbraOR26 Length: 405
# PbraOR26 Number of predicted TMHs:  5
# PbraOR26 Exp number of AAs in TMHs: 131.00215
# PbraOR26 Exp number, first 60 AAs:  16.88068
# PbraOR26 Total prob of N-in:        0.99954
# PbraOR26 POSSIBLE N-term signal sequence
PbraOR26	TMHMM2.0	inside	     1    43
PbraOR26	TMHMM2.0	TMhelix	    44    66
PbraOR26	TMHMM2.0	outside	    67    80
PbraOR26	TMHMM2.0	TMhelix	    81   103
PbraOR26	TMHMM2.0	inside	   104   141
PbraOR26	TMHMM2.0	TMhelix	   142   164
PbraOR26	TMHMM2.0	outside	   165   188
PbraOR26	TMHMM2.0	TMhelix	   189   211
PbraOR26	TMHMM2.0	inside	   212   292
PbraOR26	TMHMM2.0	TMhelix	   293   315
PbraOR26	TMHMM2.0	outside	   316   405
```

# plot in postscript,
script for making the plot in gnuplot,
data for plot

---

```
# PbraOR27 Length: 406
# PbraOR27 Number of predicted TMHs:  6
# PbraOR27 Exp number of AAs in TMHs: 131.40241
# PbraOR27 Exp number, first 60 AAs:  17.41768
# PbraOR27 Total prob of N-in:        0.99880
# PbraOR27 POSSIBLE N-term signal sequence
PbraOR27	TMHMM2.0	inside	     1    43
PbraOR27	TMHMM2.0	TMhelix	    44    66
PbraOR27	TMHMM2.0	outside	    67    75
PbraOR27	TMHMM2.0	TMhelix	    76    98
PbraOR27	TMHMM2.0	inside	    99   141
PbraOR27	TMHMM2.0	TMhelix	   142   164
PbraOR27	TMHMM2.0	outside	   165   183
PbraOR27	TMHMM2.0	TMhelix	   184   206
PbraOR27	TMHMM2.0	inside	   207   292
PbraOR27	TMHMM2.0	TMhelix	   293   315
PbraOR27	TMHMM2.0	outside	   316   373
PbraOR27	TMHMM2.0	TMhelix	   374   396
PbraOR27	TMHMM2.0	inside	   397   406
```

# plot in postscript,
script for making the plot in gnuplot,
data for plot

---

```
# PbraOR18 Length: 406
# PbraOR18 Number of predicted TMHs:  5
# PbraOR18 Exp number of AAs in TMHs: 132.87845
# PbraOR18 Exp number, first 60 AAs:  16.47081
# PbraOR18 Total prob of N-in:        0.99932
# PbraOR18 POSSIBLE N-term signal sequence
PbraOR18	TMHMM2.0	inside	     1    43
PbraOR18	TMHMM2.0	TMhelix	    44    66
PbraOR18	TMHMM2.0	outside	    67    80
PbraOR18	TMHMM2.0	TMhelix	    81   103
PbraOR18	TMHMM2.0	inside	   104   141
PbraOR18	TMHMM2.0	TMhelix	   142   164
PbraOR18	TMHMM2.0	outside	   165   188
PbraOR18	TMHMM2.0	TMhelix	   189   211
PbraOR18	TMHMM2.0	inside	   212   371
PbraOR18	TMHMM2.0	TMhelix	   372   394
PbraOR18	TMHMM2.0	outside	   395   406
```

# plot in postscript,
script for making the plot in gnuplot,
data for plot

---

```
# PbraOR19 Length: 410
# PbraOR19 Number of predicted TMHs:  5
# PbraOR19 Exp number of AAs in TMHs: 127.9809
# PbraOR19 Exp number, first 60 AAs:  24.8465
# PbraOR19 Total prob of N-in:        0.79285
# PbraOR19 POSSIBLE N-term signal sequence
PbraOR19	TMHMM2.0	inside	     1    34
PbraOR19	TMHMM2.0	TMhelix	    35    57
PbraOR19	TMHMM2.0	outside	    58    66
PbraOR19	TMHMM2.0	TMhelix	    67    89
PbraOR19	TMHMM2.0	inside	    90   133
PbraOR19	TMHMM2.0	TMhelix	   134   156
PbraOR19	TMHMM2.0	outside	   157   183
PbraOR19	TMHMM2.0	TMhelix	   184   206
PbraOR19	TMHMM2.0	inside	   207   279
PbraOR19	TMHMM2.0	TMhelix	   280   302
PbraOR19	TMHMM2.0	outside	   303   410
```

# plot in postscript,
script for making the plot in gnuplot,
data for plot

---

```
# PbraOR38 Length: 391
# PbraOR38 Number of predicted TMHs:  6
# PbraOR38 Exp number of AAs in TMHs: 122.42832
# PbraOR38 Exp number, first 60 AAs:  18.51315
# PbraOR38 Total prob of N-in:        0.71878
# PbraOR38 POSSIBLE N-term signal sequence
PbraOR38	TMHMM2.0	inside	     1    36
PbraOR38	TMHMM2.0	TMhelix	    37    56
PbraOR38	TMHMM2.0	outside	    57    65
PbraOR38	TMHMM2.0	TMhelix	    66    88
PbraOR38	TMHMM2.0	inside	    89   131
PbraOR38	TMHMM2.0	TMhelix	   132   154
PbraOR38	TMHMM2.0	outside	   155   185
PbraOR38	TMHMM2.0	TMhelix	   186   208
PbraOR38	TMHMM2.0	inside	   209   260
PbraOR38	TMHMM2.0	TMhelix	   261   280
PbraOR38	TMHMM2.0	outside	   281   284
PbraOR38	TMHMM2.0	TMhelix	   285   307
PbraOR38	TMHMM2.0	inside	   308   391
```

# plot in postscript,
script for making the plot in gnuplot,
data for plot

---

```
# PbraOR40 Length: 390
# PbraOR40 Number of predicted TMHs:  6
# PbraOR40 Exp number of AAs in TMHs: 137.86561
# PbraOR40 Exp number, first 60 AAs:  23.14915
# PbraOR40 Total prob of N-in:        0.89867
# PbraOR40 POSSIBLE N-term signal sequence
PbraOR40	TMHMM2.0	inside	     1    12
PbraOR40	TMHMM2.0	TMhelix	    13    35
PbraOR40	TMHMM2.0	outside	    36    64
PbraOR40	TMHMM2.0	TMhelix	    65    87
PbraOR40	TMHMM2.0	inside	    88   130
PbraOR40	TMHMM2.0	TMhelix	   131   153
PbraOR40	TMHMM2.0	outside	   154   186
PbraOR40	TMHMM2.0	TMhelix	   187   209
PbraOR40	TMHMM2.0	inside	   210   265
PbraOR40	TMHMM2.0	TMhelix	   266   288
PbraOR40	TMHMM2.0	outside	   289   292
PbraOR40	TMHMM2.0	TMhelix	   293   315
PbraOR40	TMHMM2.0	inside	   316   390
```

# plot in postscript,
script for making the plot in gnuplot,
data for plot

---

```
# PbraOR41 Length: 392
# PbraOR41 Number of predicted TMHs:  7
# PbraOR41 Exp number of AAs in TMHs: 147.56432
# PbraOR41 Exp number, first 60 AAs:  22.80981
# PbraOR41 Total prob of N-in:        0.99990
# PbraOR41 POSSIBLE N-term signal sequence
PbraOR41	TMHMM2.0	inside	     1    34
PbraOR41	TMHMM2.0	TMhelix	    35    57
PbraOR41	TMHMM2.0	outside	    58    66
PbraOR41	TMHMM2.0	TMhelix	    67    89
PbraOR41	TMHMM2.0	inside	    90   133
PbraOR41	TMHMM2.0	TMhelix	   134   156
PbraOR41	TMHMM2.0	outside	   157   192
PbraOR41	TMHMM2.0	TMhelix	   193   215
PbraOR41	TMHMM2.0	inside	   216   265
PbraOR41	TMHMM2.0	TMhelix	   266   288
PbraOR41	TMHMM2.0	outside	   289   291
PbraOR41	TMHMM2.0	TMhelix	   292   314
PbraOR41	TMHMM2.0	inside	   315   365
PbraOR41	TMHMM2.0	TMhelix	   366   385
PbraOR41	TMHMM2.0	outside	   386   392
```

# plot in postscript,
script for making the plot in gnuplot,
data for plot

---

```
# PbraOR15 Length: 392
# PbraOR15 Number of predicted TMHs:  6
# PbraOR15 Exp number of AAs in TMHs: 132.16342
# PbraOR15 Exp number, first 60 AAs:  22.21941
# PbraOR15 Total prob of N-in:        0.99684
# PbraOR15 POSSIBLE N-term signal sequence
PbraOR15	TMHMM2.0	inside	     1    35
PbraOR15	TMHMM2.0	TMhelix	    36    58
PbraOR15	TMHMM2.0	outside	    59    67
PbraOR15	TMHMM2.0	TMhelix	    68    90
PbraOR15	TMHMM2.0	inside	    91   132
PbraOR15	TMHMM2.0	TMhelix	   133   155
PbraOR15	TMHMM2.0	outside	   156   197
PbraOR15	TMHMM2.0	TMhelix	   198   220
PbraOR15	TMHMM2.0	inside	   221   265
PbraOR15	TMHMM2.0	TMhelix	   266   288
PbraOR15	TMHMM2.0	outside	   289   297
PbraOR15	TMHMM2.0	TMhelix	   298   315
PbraOR15	TMHMM2.0	inside	   316   392
```

# plot in postscript,
script for making the plot in gnuplot,
data for plot

---

```
# PbraOR16 Length: 397
# PbraOR16 Number of predicted TMHs:  7
# PbraOR16 Exp number of AAs in TMHs: 149.1609
# PbraOR16 Exp number, first 60 AAs:  16.99378
# PbraOR16 Total prob of N-in:        0.75945
# PbraOR16 POSSIBLE N-term signal sequence
PbraOR16	TMHMM2.0	inside	     1    43
PbraOR16	TMHMM2.0	TMhelix	    44    65
PbraOR16	TMHMM2.0	outside	    66    74
PbraOR16	TMHMM2.0	TMhelix	    75    97
PbraOR16	TMHMM2.0	inside	    98   138
PbraOR16	TMHMM2.0	TMhelix	   139   161
PbraOR16	TMHMM2.0	outside	   162   175
PbraOR16	TMHMM2.0	TMhelix	   176   198
PbraOR16	TMHMM2.0	inside	   199   204
PbraOR16	TMHMM2.0	TMhelix	   205   227
PbraOR16	TMHMM2.0	outside	   228   272
PbraOR16	TMHMM2.0	TMhelix	   273   295
PbraOR16	TMHMM2.0	inside	   296   301
PbraOR16	TMHMM2.0	TMhelix	   302   321
PbraOR16	TMHMM2.0	outside	   322   397
```

# plot in postscript,
script for making the plot in gnuplot,
data for plot

---

```
# PbraOR35 Length: 399
# PbraOR35 Number of predicted TMHs:  5
# PbraOR35 Exp number of AAs in TMHs: 128.21554
# PbraOR35 Exp number, first 60 AAs:  19.31713
# PbraOR35 Total prob of N-in:        0.70441
# PbraOR35 POSSIBLE N-term signal sequence
PbraOR35	TMHMM2.0	inside	     1    41
PbraOR35	TMHMM2.0	TMhelix	    42    64
PbraOR35	TMHMM2.0	outside	    65   139
PbraOR35	TMHMM2.0	TMhelix	   140   162
PbraOR35	TMHMM2.0	inside	   163   192
PbraOR35	TMHMM2.0	TMhelix	   193   215
PbraOR35	TMHMM2.0	outside	   216   273
PbraOR35	TMHMM2.0	TMhelix	   274   296
PbraOR35	TMHMM2.0	inside	   297   376
PbraOR35	TMHMM2.0	TMhelix	   377   396
PbraOR35	TMHMM2.0	outside	   397   399
```

# plot in postscript,
script for making the plot in gnuplot,
data for plot

---

```
# PbraOR7 Length: 400
# PbraOR7 Number of predicted TMHs:  7
# PbraOR7 Exp number of AAs in TMHs: 145.62378
# PbraOR7 Exp number, first 60 AAs:  16.86754
# PbraOR7 Total prob of N-in:        0.96995
# PbraOR7 POSSIBLE N-term signal sequence
PbraOR7	TMHMM2.0	inside	     1    44
PbraOR7	TMHMM2.0	TMhelix	    45    67
PbraOR7	TMHMM2.0	outside	    68    76
PbraOR7	TMHMM2.0	TMhelix	    77    96
PbraOR7	TMHMM2.0	inside	    97   134
PbraOR7	TMHMM2.0	TMhelix	   135   152
PbraOR7	TMHMM2.0	outside	   153   185
PbraOR7	TMHMM2.0	TMhelix	   186   208
PbraOR7	TMHMM2.0	inside	   209   270
PbraOR7	TMHMM2.0	TMhelix	   271   293
PbraOR7	TMHMM2.0	outside	   294   297
PbraOR7	TMHMM2.0	TMhelix	   298   320
PbraOR7	TMHMM2.0	inside	   321   360
PbraOR7	TMHMM2.0	TMhelix	   361   383
PbraOR7	TMHMM2.0	outside	   384   400
```

# plot in postscript,
script for making the plot in gnuplot,
data for plot

---

```
# PbraOR8 Length: 399
# PbraOR8 Number of predicted TMHs:  5
# PbraOR8 Exp number of AAs in TMHs: 130.84177
# PbraOR8 Exp number, first 60 AAs:  19.99733
# PbraOR8 Total prob of N-in:        0.89083
# PbraOR8 POSSIBLE N-term signal sequence
PbraOR8	TMHMM2.0	inside	     1    42
PbraOR8	TMHMM2.0	TMhelix	    43    65
PbraOR8	TMHMM2.0	outside	    66    74
PbraOR8	TMHMM2.0	TMhelix	    75    97
PbraOR8	TMHMM2.0	inside	    98   273
PbraOR8	TMHMM2.0	TMhelix	   274   296
PbraOR8	TMHMM2.0	outside	   297   305
PbraOR8	TMHMM2.0	TMhelix	   306   322
PbraOR8	TMHMM2.0	inside	   323   373
PbraOR8	TMHMM2.0	TMhelix	   374   396
PbraOR8	TMHMM2.0	outside	   397   399
```

# plot in postscript,
script for making the plot in gnuplot,
data for plot

---

```
# PbraOR9 Length: 400
# PbraOR9 Number of predicted TMHs:  6
# PbraOR9 Exp number of AAs in TMHs: 146.46003
# PbraOR9 Exp number, first 60 AAs:  17.91417
# PbraOR9 Total prob of N-in:        0.92517
# PbraOR9 POSSIBLE N-term signal sequence
PbraOR9	TMHMM2.0	inside	     1    42
PbraOR9	TMHMM2.0	TMhelix	    43    65
PbraOR9	TMHMM2.0	outside	    66    74
PbraOR9	TMHMM2.0	TMhelix	    75    97
PbraOR9	TMHMM2.0	inside	    98   140
PbraOR9	TMHMM2.0	TMhelix	   141   163
PbraOR9	TMHMM2.0	outside	   164   204
PbraOR9	TMHMM2.0	TMhelix	   205   227
PbraOR9	TMHMM2.0	inside	   228   274
PbraOR9	TMHMM2.0	TMhelix	   275   297
PbraOR9	TMHMM2.0	outside	   298   363
PbraOR9	TMHMM2.0	TMhelix	   364   383
PbraOR9	TMHMM2.0	inside	   384   400
```

# plot in postscript,
script for making the plot in gnuplot,
data for plot

---

```
# PbraOrco Length: 471
# PbraOrco Number of predicted TMHs:  7
# PbraOrco Exp number of AAs in TMHs: 155.12462
# PbraOrco Exp number, first 60 AAs:  15.37107
# PbraOrco Total prob of N-in:        0.98954
# PbraOrco POSSIBLE N-term signal sequence
PbraOrco	TMHMM2.0	inside	     1    45
PbraOrco	TMHMM2.0	TMhelix	    46    65
PbraOrco	TMHMM2.0	outside	    66    74
PbraOrco	TMHMM2.0	TMhelix	    75    97
PbraOrco	TMHMM2.0	inside	    98   135
PbraOrco	TMHMM2.0	TMhelix	   136   158
PbraOrco	TMHMM2.0	outside	   159   194
PbraOrco	TMHMM2.0	TMhelix	   195   217
PbraOrco	TMHMM2.0	inside	   218   346
PbraOrco	TMHMM2.0	TMhelix	   347   364
PbraOrco	TMHMM2.0	outside	   365   373
PbraOrco	TMHMM2.0	TMhelix	   374   396
PbraOrco	TMHMM2.0	inside	   397   444
PbraOrco	TMHMM2.0	TMhelix	   445   467
PbraOrco	TMHMM2.0	outside	   468   471
```

# plot in postscript,
script for making the plot in gnuplot,
data for plot

---

```
# PbraOR47 Length: 441
# PbraOR47 Number of predicted TMHs:  7
# PbraOR47 Exp number of AAs in TMHs: 154.29581
# PbraOR47 Exp number, first 60 AAs:  26.02584
# PbraOR47 Total prob of N-in:        0.65905
# PbraOR47 POSSIBLE N-term signal sequence
PbraOR47	TMHMM2.0	inside	     1    39
PbraOR47	TMHMM2.0	TMhelix	    40    62
PbraOR47	TMHMM2.0	outside	    63    76
PbraOR47	TMHMM2.0	TMhelix	    77    96
PbraOR47	TMHMM2.0	inside	    97   133
PbraOR47	TMHMM2.0	TMhelix	   134   156
PbraOR47	TMHMM2.0	outside	   157   189
PbraOR47	TMHMM2.0	TMhelix	   190   212
PbraOR47	TMHMM2.0	inside	   213   316
PbraOR47	TMHMM2.0	TMhelix	   317   339
PbraOR47	TMHMM2.0	outside	   340   348
PbraOR47	TMHMM2.0	TMhelix	   349   371
PbraOR47	TMHMM2.0	inside	   372   413
PbraOR47	TMHMM2.0	TMhelix	   414   436
PbraOR47	TMHMM2.0	outside	   437   441
```

# plot in postscript,
script for making the plot in gnuplot,
data for plot

---

```
# PbraOR59 Length: 438
# PbraOR59 Number of predicted TMHs:  5
# PbraOR59 Exp number of AAs in TMHs: 119.72121
# PbraOR59 Exp number, first 60 AAs:  1.20924
# PbraOR59 Total prob of N-in:        0.37506
PbraOR59	TMHMM2.0	outside	     1    59
PbraOR59	TMHMM2.0	TMhelix	    60    82
PbraOR59	TMHMM2.0	inside	    83   167
PbraOR59	TMHMM2.0	TMhelix	   168   190
PbraOR59	TMHMM2.0	outside	   191   215
PbraOR59	TMHMM2.0	TMhelix	   216   238
PbraOR59	TMHMM2.0	inside	   239   309
PbraOR59	TMHMM2.0	TMhelix	   310   329
PbraOR59	TMHMM2.0	outside	   330   333
PbraOR59	TMHMM2.0	TMhelix	   334   356
PbraOR59	TMHMM2.0	inside	   357   438
```

# plot in postscript,
script for making the plot in gnuplot,
data for plot

---

```
# PbraOR63 Length: 380
# PbraOR63 Number of predicted TMHs:  6
# PbraOR63 Exp number of AAs in TMHs: 133.28406
# PbraOR63 Exp number, first 60 AAs:  21.4363
# PbraOR63 Total prob of N-in:        0.61149
# PbraOR63 POSSIBLE N-term signal sequence
PbraOR63	TMHMM2.0	inside	     1    38
PbraOR63	TMHMM2.0	TMhelix	    39    61
PbraOR63	TMHMM2.0	outside	    62    65
PbraOR63	TMHMM2.0	TMhelix	    66    83
PbraOR63	TMHMM2.0	inside	    84   123
PbraOR63	TMHMM2.0	TMhelix	   124   141
PbraOR63	TMHMM2.0	outside	   142   186
PbraOR63	TMHMM2.0	TMhelix	   187   209
PbraOR63	TMHMM2.0	inside	   210   255
PbraOR63	TMHMM2.0	TMhelix	   256   278
PbraOR63	TMHMM2.0	outside	   279   281
PbraOR63	TMHMM2.0	TMhelix	   282   304
PbraOR63	TMHMM2.0	inside	   305   380
```

# plot in postscript,
script for making the plot in gnuplot,
data for plot

---

```
# PbraOR61 Length: 395
# PbraOR61 Number of predicted TMHs:  7
# PbraOR61 Exp number of AAs in TMHs: 151.93317
# PbraOR61 Exp number, first 60 AAs:  18.29344
# PbraOR61 Total prob of N-in:        0.93108
# PbraOR61 POSSIBLE N-term signal sequence
PbraOR61	TMHMM2.0	inside	     1    40
PbraOR61	TMHMM2.0	TMhelix	    41    63
PbraOR61	TMHMM2.0	outside	    64    75
PbraOR61	TMHMM2.0	TMhelix	    76    93
PbraOR61	TMHMM2.0	inside	    94   131
PbraOR61	TMHMM2.0	TMhelix	   132   154
PbraOR61	TMHMM2.0	outside	   155   191
PbraOR61	TMHMM2.0	TMhelix	   192   223
PbraOR61	TMHMM2.0	inside	   224   269
PbraOR61	TMHMM2.0	TMhelix	   270   292
PbraOR61	TMHMM2.0	outside	   293   301
PbraOR61	TMHMM2.0	TMhelix	   302   324
PbraOR61	TMHMM2.0	inside	   325   371
PbraOR61	TMHMM2.0	TMhelix	   372   394
PbraOR61	TMHMM2.0	outside	   395   395
```

# plot in postscript,
script for making the plot in gnuplot,
data for plot

---

```
# PbraOR62 Length: 393
# PbraOR62 Number of predicted TMHs:  7
# PbraOR62 Exp number of AAs in TMHs: 145.56689
# PbraOR62 Exp number, first 60 AAs:  22.13046
# PbraOR62 Total prob of N-in:        0.20784
# PbraOR62 POSSIBLE N-term signal sequence
PbraOR62	TMHMM2.0	outside	     1    31
PbraOR62	TMHMM2.0	TMhelix	    32    54
PbraOR62	TMHMM2.0	inside	    55    60
PbraOR62	TMHMM2.0	TMhelix	    61    83
PbraOR62	TMHMM2.0	outside	    84   124
PbraOR62	TMHMM2.0	TMhelix	   125   147
PbraOR62	TMHMM2.0	inside	   148   177
PbraOR62	TMHMM2.0	TMhelix	   178   200
PbraOR62	TMHMM2.0	outside	   201   262
PbraOR62	TMHMM2.0	TMhelix	   263   285
PbraOR62	TMHMM2.0	inside	   286   297
PbraOR62	TMHMM2.0	TMhelix	   298   320
PbraOR62	TMHMM2.0	outside	   321   365
PbraOR62	TMHMM2.0	TMhelix	   366   388
PbraOR62	TMHMM2.0	inside	   389   393
```

# plot in postscript,
script for making the plot in gnuplot,
data for plot

---

```
# PbraOR6 Length: 393
# PbraOR6 Number of predicted TMHs:  6
# PbraOR6 Exp number of AAs in TMHs: 139.76105
# PbraOR6 Exp number, first 60 AAs:  20.12829
# PbraOR6 Total prob of N-in:        0.82728
# PbraOR6 POSSIBLE N-term signal sequence
PbraOR6	TMHMM2.0	inside	     1    39
PbraOR6	TMHMM2.0	TMhelix	    40    62
PbraOR6	TMHMM2.0	outside	    63    74
PbraOR6	TMHMM2.0	TMhelix	    75    92
PbraOR6	TMHMM2.0	inside	    93   131
PbraOR6	TMHMM2.0	TMhelix	   132   151
PbraOR6	TMHMM2.0	outside	   152   197
PbraOR6	TMHMM2.0	TMhelix	   198   220
PbraOR6	TMHMM2.0	inside	   221   269
PbraOR6	TMHMM2.0	TMhelix	   270   292
PbraOR6	TMHMM2.0	outside	   293   301
PbraOR6	TMHMM2.0	TMhelix	   302   324
PbraOR6	TMHMM2.0	inside	   325   393
```

# plot in postscript,
script for making the plot in gnuplot,
data for plot

---

```
# PbraOR60 Length: 392
# PbraOR60 Number of predicted TMHs:  6
# PbraOR60 Exp number of AAs in TMHs: 132.98623
# PbraOR60 Exp number, first 60 AAs:  21.15865
# PbraOR60 Total prob of N-in:        0.18972
# PbraOR60 POSSIBLE N-term signal sequence
PbraOR60	TMHMM2.0	outside	     1    37
PbraOR60	TMHMM2.0	TMhelix	    38    60
PbraOR60	TMHMM2.0	inside	    61   127
PbraOR60	TMHMM2.0	TMhelix	   128   150
PbraOR60	TMHMM2.0	outside	   151   188
PbraOR60	TMHMM2.0	TMhelix	   189   211
PbraOR60	TMHMM2.0	inside	   212   271
PbraOR60	TMHMM2.0	TMhelix	   272   291
PbraOR60	TMHMM2.0	outside	   292   300
PbraOR60	TMHMM2.0	TMhelix	   301   320
PbraOR60	TMHMM2.0	inside	   321   362
PbraOR60	TMHMM2.0	TMhelix	   363   382
PbraOR60	TMHMM2.0	outside	   383   392
```

# plot in postscript,
script for making the plot in gnuplot,
data for plot

---

```
# PbraOR48 Length: 393
# PbraOR48 Number of predicted TMHs:  7
# PbraOR48 Exp number of AAs in TMHs: 152.66162
# PbraOR48 Exp number, first 60 AAs:  23.60465
# PbraOR48 Total prob of N-in:        0.88651
# PbraOR48 POSSIBLE N-term signal sequence
PbraOR48	TMHMM2.0	inside	     1    36
PbraOR48	TMHMM2.0	TMhelix	    37    56
PbraOR48	TMHMM2.0	outside	    57    70
PbraOR48	TMHMM2.0	TMhelix	    71    93
PbraOR48	TMHMM2.0	inside	    94   127
PbraOR48	TMHMM2.0	TMhelix	   128   150
PbraOR48	TMHMM2.0	outside	   151   179
PbraOR48	TMHMM2.0	TMhelix	   180   202
PbraOR48	TMHMM2.0	inside	   203   261
PbraOR48	TMHMM2.0	TMhelix	   262   284
PbraOR48	TMHMM2.0	outside	   285   288
PbraOR48	TMHMM2.0	TMhelix	   289   311
PbraOR48	TMHMM2.0	inside	   312   355
PbraOR48	TMHMM2.0	TMhelix	   356   375
PbraOR48	TMHMM2.0	outside	   376   393
```

# plot in postscript,
script for making the plot in gnuplot,
data for plot

---

```
# PbraOR49 Length: 394
# PbraOR49 Number of predicted TMHs:  3
# PbraOR49 Exp number of AAs in TMHs: 109.88983
# PbraOR49 Exp number, first 60 AAs:  20.34301
# PbraOR49 Total prob of N-in:        0.97514
# PbraOR49 POSSIBLE N-term signal sequence
PbraOR49	TMHMM2.0	inside	     1    42
PbraOR49	TMHMM2.0	TMhelix	    43    65
PbraOR49	TMHMM2.0	outside	    66    69
PbraOR49	TMHMM2.0	TMhelix	    70    92
PbraOR49	TMHMM2.0	inside	    93   126
PbraOR49	TMHMM2.0	TMhelix	   127   149
PbraOR49	TMHMM2.0	outside	   150   394
```

# plot in postscript,
script for making the plot in gnuplot,
data for plot

---

```
# PbraOR50 Length: 394
# PbraOR50 Number of predicted TMHs:  4
# PbraOR50 Exp number of AAs in TMHs: 107.38502
# PbraOR50 Exp number, first 60 AAs:  42.42378
# PbraOR50 Total prob of N-in:        0.09163
# PbraOR50 POSSIBLE N-term signal sequence
PbraOR50	TMHMM2.0	outside	     1     9
PbraOR50	TMHMM2.0	TMhelix	    10    32
PbraOR50	TMHMM2.0	inside	    33    36
PbraOR50	TMHMM2.0	TMhelix	    37    59
PbraOR50	TMHMM2.0	outside	    60    73
PbraOR50	TMHMM2.0	TMhelix	    74    93
PbraOR50	TMHMM2.0	inside	    94   131
PbraOR50	TMHMM2.0	TMhelix	   132   151
PbraOR50	TMHMM2.0	outside	   152   394
```

# plot in postscript,
script for making the plot in gnuplot,
data for plot

---

```
# PbraOR51 Length: 389
# PbraOR51 Number of predicted TMHs:  6
# PbraOR51 Exp number of AAs in TMHs: 132.73656
# PbraOR51 Exp number, first 60 AAs:  21.32471
# PbraOR51 Total prob of N-in:        0.98721
# PbraOR51 POSSIBLE N-term signal sequence
PbraOR51	TMHMM2.0	inside	     1    36
PbraOR51	TMHMM2.0	TMhelix	    37    59
PbraOR51	TMHMM2.0	outside	    60    68
PbraOR51	TMHMM2.0	TMhelix	    69    88
PbraOR51	TMHMM2.0	inside	    89   123
PbraOR51	TMHMM2.0	TMhelix	   124   146
PbraOR51	TMHMM2.0	outside	   147   180
PbraOR51	TMHMM2.0	TMhelix	   181   203
PbraOR51	TMHMM2.0	inside	   204   264
PbraOR51	TMHMM2.0	TMhelix	   265   287
PbraOR51	TMHMM2.0	outside	   288   291
PbraOR51	TMHMM2.0	TMhelix	   292   314
PbraOR51	TMHMM2.0	inside	   315   389
```

# plot in postscript,
script for making the plot in gnuplot,
data for plot

---

```
# PbraOR52 Length: 388
# PbraOR52 Number of predicted TMHs:  5
# PbraOR52 Exp number of AAs in TMHs: 120.56516
# PbraOR52 Exp number, first 60 AAs:  23.15577
# PbraOR52 Total prob of N-in:        0.58639
# PbraOR52 POSSIBLE N-term signal sequence
PbraOR52	TMHMM2.0	outside	     1    36
PbraOR52	TMHMM2.0	TMhelix	    37    59
PbraOR52	TMHMM2.0	inside	    60   122
PbraOR52	TMHMM2.0	TMhelix	   123   145
PbraOR52	TMHMM2.0	outside	   146   170
PbraOR52	TMHMM2.0	TMhelix	   171   190
PbraOR52	TMHMM2.0	inside	   191   262
PbraOR52	TMHMM2.0	TMhelix	   263   285
PbraOR52	TMHMM2.0	outside	   286   289
PbraOR52	TMHMM2.0	TMhelix	   290   312
PbraOR52	TMHMM2.0	inside	   313   388
```

# plot in postscript,
script for making the plot in gnuplot,
data for plot

---

```
# PbraOR44 Length: 389
# PbraOR44 Number of predicted TMHs:  8
# PbraOR44 Exp number of AAs in TMHs: 168.63152
# PbraOR44 Exp number, first 60 AAs:  22.44685
# PbraOR44 Total prob of N-in:        0.98982
# PbraOR44 POSSIBLE N-term signal sequence
PbraOR44	TMHMM2.0	inside	     1    29
PbraOR44	TMHMM2.0	TMhelix	    30    52
PbraOR44	TMHMM2.0	outside	    53    61
PbraOR44	TMHMM2.0	TMhelix	    62    84
PbraOR44	TMHMM2.0	inside	    85   125
PbraOR44	TMHMM2.0	TMhelix	   126   148
PbraOR44	TMHMM2.0	outside	   149   167
PbraOR44	TMHMM2.0	TMhelix	   168   187
PbraOR44	TMHMM2.0	inside	   188   193
PbraOR44	TMHMM2.0	TMhelix	   194   212
PbraOR44	TMHMM2.0	outside	   213   264
PbraOR44	TMHMM2.0	TMhelix	   265   287
PbraOR44	TMHMM2.0	inside	   288   293
PbraOR44	TMHMM2.0	TMhelix	   294   316
PbraOR44	TMHMM2.0	outside	   317   362
PbraOR44	TMHMM2.0	TMhelix	   363   385
PbraOR44	TMHMM2.0	inside	   386   389
```

# plot in postscript,
script for making the plot in gnuplot,
data for plot

---

```
# PbraOR45a Length: 385
# PbraOR45a Number of predicted TMHs:  7
# PbraOR45a Exp number of AAs in TMHs: 160.99665
# PbraOR45a Exp number, first 60 AAs:  40.45578
# PbraOR45a Total prob of N-in:        0.94942
# PbraOR45a POSSIBLE N-term signal sequence
PbraOR45a	TMHMM2.0	inside	     1     6
PbraOR45a	TMHMM2.0	TMhelix	     7    24
PbraOR45a	TMHMM2.0	outside	    25    33
PbraOR45a	TMHMM2.0	TMhelix	    34    56
PbraOR45a	TMHMM2.0	inside	    57    62
PbraOR45a	TMHMM2.0	TMhelix	    63    85
PbraOR45a	TMHMM2.0	outside	    86   139
PbraOR45a	TMHMM2.0	TMhelix	   140   162
PbraOR45a	TMHMM2.0	inside	   163   186
PbraOR45a	TMHMM2.0	TMhelix	   187   209
PbraOR45a	TMHMM2.0	outside	   210   253
PbraOR45a	TMHMM2.0	TMhelix	   254   276
PbraOR45a	TMHMM2.0	inside	   277   282
PbraOR45a	TMHMM2.0	TMhelix	   283   305
PbraOR45a	TMHMM2.0	outside	   306   385
```

# plot in postscript,
script for making the plot in gnuplot,
data for plot

---

```
# PbraOR45b Length: 385
# PbraOR45b Number of predicted TMHs:  6
# PbraOR45b Exp number of AAs in TMHs: 153.49028
# PbraOR45b Exp number, first 60 AAs:  32.66734
# PbraOR45b Total prob of N-in:        0.77449
# PbraOR45b POSSIBLE N-term signal sequence
PbraOR45b	TMHMM2.0	inside	     1     6
PbraOR45b	TMHMM2.0	TMhelix	     7    24
PbraOR45b	TMHMM2.0	outside	    25    33
PbraOR45b	TMHMM2.0	TMhelix	    34    56
PbraOR45b	TMHMM2.0	inside	    57   127
PbraOR45b	TMHMM2.0	TMhelix	   128   150
PbraOR45b	TMHMM2.0	outside	   151   169
PbraOR45b	TMHMM2.0	TMhelix	   170   192
PbraOR45b	TMHMM2.0	inside	   193   248
PbraOR45b	TMHMM2.0	TMhelix	   249   271
PbraOR45b	TMHMM2.0	outside	   272   285
PbraOR45b	TMHMM2.0	TMhelix	   286   308
PbraOR45b	TMHMM2.0	inside	   309   385
```

# plot in postscript,
script for making the plot in gnuplot,
data for plot

---

```
# PbraOR53 Length: 432
# PbraOR53 Number of predicted TMHs:  4
# PbraOR53 Exp number of AAs in TMHs: 99.47587
# PbraOR53 Exp number, first 60 AAs:  3.91016
# PbraOR53 Total prob of N-in:        0.52448
PbraOR53	TMHMM2.0	inside	     1   166
PbraOR53	TMHMM2.0	TMhelix	   167   189
PbraOR53	TMHMM2.0	outside	   190   222
PbraOR53	TMHMM2.0	TMhelix	   223   245
PbraOR53	TMHMM2.0	inside	   246   302
PbraOR53	TMHMM2.0	TMhelix	   303   325
PbraOR53	TMHMM2.0	outside	   326   334
PbraOR53	TMHMM2.0	TMhelix	   335   357
PbraOR53	TMHMM2.0	inside	   358   432
```

# plot in postscript,
script for making the plot in gnuplot,
data for plot

---

```
# PbraOR46 Length: 392
# PbraOR46 Number of predicted TMHs:  5
# PbraOR46 Exp number of AAs in TMHs: 116.30122
# PbraOR46 Exp number, first 60 AAs:  18.27889
# PbraOR46 Total prob of N-in:        0.57974
# PbraOR46 POSSIBLE N-term signal sequence
PbraOR46	TMHMM2.0	outside	     1    35
PbraOR46	TMHMM2.0	TMhelix	    36    53
PbraOR46	TMHMM2.0	inside	    54   129
PbraOR46	TMHMM2.0	TMhelix	   130   152
PbraOR46	TMHMM2.0	outside	   153   171
PbraOR46	TMHMM2.0	TMhelix	   172   191
PbraOR46	TMHMM2.0	inside	   192   263
PbraOR46	TMHMM2.0	TMhelix	   264   286
PbraOR46	TMHMM2.0	outside	   287   295
PbraOR46	TMHMM2.0	TMhelix	   296   318
PbraOR46	TMHMM2.0	inside	   319   392
```

# plot in postscript,
script for making the plot in gnuplot,
data for plot

---

```
# PbraOR43 Length: 427
# PbraOR43 Number of predicted TMHs:  6
# PbraOR43 Exp number of AAs in TMHs: 136.89397
# PbraOR43 Exp number, first 60 AAs:  4.25323
# PbraOR43 Total prob of N-in:        0.90309
PbraOR43	TMHMM2.0	inside	     1    58
PbraOR43	TMHMM2.0	TMhelix	    59    81
PbraOR43	TMHMM2.0	outside	    82    95
PbraOR43	TMHMM2.0	TMhelix	    96   118
PbraOR43	TMHMM2.0	inside	   119   154
PbraOR43	TMHMM2.0	TMhelix	   155   177
PbraOR43	TMHMM2.0	outside	   178   223
PbraOR43	TMHMM2.0	TMhelix	   224   246
PbraOR43	TMHMM2.0	inside	   247   296
PbraOR43	TMHMM2.0	TMhelix	   297   319
PbraOR43	TMHMM2.0	outside	   320   328
PbraOR43	TMHMM2.0	TMhelix	   329   351
PbraOR43	TMHMM2.0	inside	   352   427
```

# plot in postscript,
script for making the plot in gnuplot,
data for plot

---

```
# PbraOR28 Length: 410
# PbraOR28 Number of predicted TMHs:  5
# PbraOR28 Exp number of AAs in TMHs: 119.41654
# PbraOR28 Exp number, first 60 AAs:  9.09127
# PbraOR28 Total prob of N-in:        0.85505
PbraOR28	TMHMM2.0	inside	     1    48
PbraOR28	TMHMM2.0	TMhelix	    49    71
PbraOR28	TMHMM2.0	outside	    72    85
PbraOR28	TMHMM2.0	TMhelix	    86   108
PbraOR28	TMHMM2.0	inside	   109   151
PbraOR28	TMHMM2.0	TMhelix	   152   174
PbraOR28	TMHMM2.0	outside	   175   205
PbraOR28	TMHMM2.0	TMhelix	   206   228
PbraOR28	TMHMM2.0	inside	   229   289
PbraOR28	TMHMM2.0	TMhelix	   290   312
PbraOR28	TMHMM2.0	outside	   313   410
```

# plot in postscript,
script for making the plot in gnuplot,
data for plot

---

```
# PbraOR2 Length: 387
# PbraOR2 Number of predicted TMHs:  6
# PbraOR2 Exp number of AAs in TMHs: 150.51871
# PbraOR2 Exp number, first 60 AAs:  22.5443
# PbraOR2 Total prob of N-in:        0.99967
# PbraOR2 POSSIBLE N-term signal sequence
PbraOR2	TMHMM2.0	inside	     1    37
PbraOR2	TMHMM2.0	TMhelix	    38    60
PbraOR2	TMHMM2.0	outside	    61    69
PbraOR2	TMHMM2.0	TMhelix	    70    89
PbraOR2	TMHMM2.0	inside	    90   132
PbraOR2	TMHMM2.0	TMhelix	   133   155
PbraOR2	TMHMM2.0	outside	   156   180
PbraOR2	TMHMM2.0	TMhelix	   181   203
PbraOR2	TMHMM2.0	inside	   204   260
PbraOR2	TMHMM2.0	TMhelix	   261   283
PbraOR2	TMHMM2.0	outside	   284   292
PbraOR2	TMHMM2.0	TMhelix	   293   310
PbraOR2	TMHMM2.0	inside	   311   387
```

# plot in postscript,
script for making the plot in gnuplot,
data for plot

---

```
# PbraOR23 Length: 387
# PbraOR23 Number of predicted TMHs:  4
# PbraOR23 Exp number of AAs in TMHs: 96.1509900000001
# PbraOR23 Exp number, first 60 AAs:  19.74472
# PbraOR23 Total prob of N-in:        0.98422
# PbraOR23 POSSIBLE N-term signal sequence
PbraOR23	TMHMM2.0	inside	     1    37
PbraOR23	TMHMM2.0	TMhelix	    38    57
PbraOR23	TMHMM2.0	outside	    58    71
PbraOR23	TMHMM2.0	TMhelix	    72    91
PbraOR23	TMHMM2.0	inside	    92   123
PbraOR23	TMHMM2.0	TMhelix	   124   146
PbraOR23	TMHMM2.0	outside	   147   182
PbraOR23	TMHMM2.0	TMhelix	   183   205
PbraOR23	TMHMM2.0	inside	   206   387
```

# plot in postscript,
script for making the plot in gnuplot,
data for plot

---

```
# PbraOR39 Length: 391
# PbraOR39 Number of predicted TMHs:  7
# PbraOR39 Exp number of AAs in TMHs: 150.61832
# PbraOR39 Exp number, first 60 AAs:  32.69185
# PbraOR39 Total prob of N-in:        0.25073
# PbraOR39 POSSIBLE N-term signal sequence
PbraOR39	TMHMM2.0	outside	     1    12
PbraOR39	TMHMM2.0	TMhelix	    13    30
PbraOR39	TMHMM2.0	inside	    31    42
PbraOR39	TMHMM2.0	TMhelix	    43    65
PbraOR39	TMHMM2.0	outside	    66    74
PbraOR39	TMHMM2.0	TMhelix	    75    97
PbraOR39	TMHMM2.0	inside	    98   150
PbraOR39	TMHMM2.0	TMhelix	   151   173
PbraOR39	TMHMM2.0	outside	   174   207
PbraOR39	TMHMM2.0	TMhelix	   208   230
PbraOR39	TMHMM2.0	inside	   231   280
PbraOR39	TMHMM2.0	TMhelix	   281   303
PbraOR39	TMHMM2.0	outside	   304   307
PbraOR39	TMHMM2.0	TMhelix	   308   330
PbraOR39	TMHMM2.0	inside	   331   391
```

# plot in postscript,
script for making the plot in gnuplot,
data for plot

---

```
# PbraOR4 Length: 396
# PbraOR4 Number of predicted TMHs:  6
# PbraOR4 Exp number of AAs in TMHs: 128.79739
# PbraOR4 Exp number, first 60 AAs:  19.09208
# PbraOR4 Total prob of N-in:        0.92945
# PbraOR4 POSSIBLE N-term signal sequence
PbraOR4	TMHMM2.0	inside	     1    41
PbraOR4	TMHMM2.0	TMhelix	    42    64
PbraOR4	TMHMM2.0	outside	    65    83
PbraOR4	TMHMM2.0	TMhelix	    84   106
PbraOR4	TMHMM2.0	inside	   107   141
PbraOR4	TMHMM2.0	TMhelix	   142   164
PbraOR4	TMHMM2.0	outside	   165   198
PbraOR4	TMHMM2.0	TMhelix	   199   221
PbraOR4	TMHMM2.0	inside	   222   272
PbraOR4	TMHMM2.0	TMhelix	   273   295
PbraOR4	TMHMM2.0	outside	   296   304
PbraOR4	TMHMM2.0	TMhelix	   305   324
PbraOR4	TMHMM2.0	inside	   325   396
```

# plot in postscript,
script for making the plot in gnuplot,
data for plot

---

```
# PbraOR5 Length: 407
# PbraOR5 Number of predicted TMHs:  7
# PbraOR5 Exp number of AAs in TMHs: 157.08135
# PbraOR5 Exp number, first 60 AAs:  21.97094
# PbraOR5 Total prob of N-in:        0.99467
# PbraOR5 POSSIBLE N-term signal sequence
PbraOR5	TMHMM2.0	inside	     1    30
PbraOR5	TMHMM2.0	TMhelix	    31    53
PbraOR5	TMHMM2.0	outside	    54    62
PbraOR5	TMHMM2.0	TMhelix	    63    85
PbraOR5	TMHMM2.0	inside	    86   141
PbraOR5	TMHMM2.0	TMhelix	   142   161
PbraOR5	TMHMM2.0	outside	   162   198
PbraOR5	TMHMM2.0	TMhelix	   199   221
PbraOR5	TMHMM2.0	inside	   222   280
PbraOR5	TMHMM2.0	TMhelix	   281   303
PbraOR5	TMHMM2.0	outside	   304   312
PbraOR5	TMHMM2.0	TMhelix	   313   335
PbraOR5	TMHMM2.0	inside	   336   360
PbraOR5	TMHMM2.0	TMhelix	   361   383
PbraOR5	TMHMM2.0	outside	   384   407
```

# plot in postscript,
script for making the plot in gnuplot,
data for plot

---

```
# PrapOR55 Length: 417
# PrapOR55 Number of predicted TMHs:  5
# PrapOR55 Exp number of AAs in TMHs: 108.50504
# PrapOR55 Exp number, first 60 AAs:  10.15712
# PrapOR55 Total prob of N-in:        0.33004
# PrapOR55 POSSIBLE N-term signal sequence
PrapOR55	TMHMM2.0	outside	     1    50
PrapOR55	TMHMM2.0	TMhelix	    51    73
PrapOR55	TMHMM2.0	inside	    74   140
PrapOR55	TMHMM2.0	TMhelix	   141   163
PrapOR55	TMHMM2.0	outside	   164   184
PrapOR55	TMHMM2.0	TMhelix	   185   207
PrapOR55	TMHMM2.0	inside	   208   284
PrapOR55	TMHMM2.0	TMhelix	   285   307
PrapOR55	TMHMM2.0	outside	   308   316
PrapOR55	TMHMM2.0	TMhelix	   317   334
PrapOR55	TMHMM2.0	inside	   335   417
```

# plot in postscript,
script for making the plot in gnuplot,
data for plot

---

```
# PrapOR54 Length: 392
# PrapOR54 Number of predicted TMHs:  4
# PrapOR54 Exp number of AAs in TMHs: 107.69433
# PrapOR54 Exp number, first 60 AAs:  17.5719
# PrapOR54 Total prob of N-in:        0.66849
# PrapOR54 POSSIBLE N-term signal sequence
PrapOR54	TMHMM2.0	inside	     1   113
PrapOR54	TMHMM2.0	TMhelix	   114   136
PrapOR54	TMHMM2.0	outside	   137   158
PrapOR54	TMHMM2.0	TMhelix	   159   181
PrapOR54	TMHMM2.0	inside	   182   259
PrapOR54	TMHMM2.0	TMhelix	   260   282
PrapOR54	TMHMM2.0	outside	   283   291
PrapOR54	TMHMM2.0	TMhelix	   292   309
PrapOR54	TMHMM2.0	inside	   310   392
```

# plot in postscript,
script for making the plot in gnuplot,
data for plot

---

```
# PrapOR36 Length: 419
# PrapOR36 Number of predicted TMHs:  6
# PrapOR36 Exp number of AAs in TMHs: 124.3753
# PrapOR36 Exp number, first 60 AAs:  12.62174
# PrapOR36 Total prob of N-in:        0.99811
# PrapOR36 POSSIBLE N-term signal sequence
PrapOR36	TMHMM2.0	inside	     1    46
PrapOR36	TMHMM2.0	TMhelix	    47    69
PrapOR36	TMHMM2.0	outside	    70    83
PrapOR36	TMHMM2.0	TMhelix	    84   106
PrapOR36	TMHMM2.0	inside	   107   147
PrapOR36	TMHMM2.0	TMhelix	   148   170
PrapOR36	TMHMM2.0	outside	   171   196
PrapOR36	TMHMM2.0	TMhelix	   197   219
PrapOR36	TMHMM2.0	inside	   220   292
PrapOR36	TMHMM2.0	TMhelix	   293   315
PrapOR36	TMHMM2.0	outside	   316   324
PrapOR36	TMHMM2.0	TMhelix	   325   347
PrapOR36	TMHMM2.0	inside	   348   419
```

# plot in postscript,
script for making the plot in gnuplot,
data for plot

---

```
# PrapOR30 Length: 397
# PrapOR30 Number of predicted TMHs:  6
# PrapOR30 Exp number of AAs in TMHs: 134.79836
# PrapOR30 Exp number, first 60 AAs:  22.46196
# PrapOR30 Total prob of N-in:        0.33710
# PrapOR30 POSSIBLE N-term signal sequence
PrapOR30	TMHMM2.0	outside	     1    35
PrapOR30	TMHMM2.0	TMhelix	    36    58
PrapOR30	TMHMM2.0	inside	    59    69
PrapOR30	TMHMM2.0	TMhelix	    70    92
PrapOR30	TMHMM2.0	outside	    93   131
PrapOR30	TMHMM2.0	TMhelix	   132   154
PrapOR30	TMHMM2.0	inside	   155   191
PrapOR30	TMHMM2.0	TMhelix	   192   214
PrapOR30	TMHMM2.0	outside	   215   268
PrapOR30	TMHMM2.0	TMhelix	   269   291
PrapOR30	TMHMM2.0	inside	   292   303
PrapOR30	TMHMM2.0	TMhelix	   304   326
PrapOR30	TMHMM2.0	outside	   327   397
```

# plot in postscript,
script for making the plot in gnuplot,
data for plot

---

```
# PrapOR29 Length: 394
# PrapOR29 Number of predicted TMHs:  6
# PrapOR29 Exp number of AAs in TMHs: 132.00491
# PrapOR29 Exp number, first 60 AAs:  21.52252
# PrapOR29 Total prob of N-in:        0.97037
# PrapOR29 POSSIBLE N-term signal sequence
PrapOR29	TMHMM2.0	inside	     1    35
PrapOR29	TMHMM2.0	TMhelix	    36    58
PrapOR29	TMHMM2.0	outside	    59    72
PrapOR29	TMHMM2.0	TMhelix	    73    90
PrapOR29	TMHMM2.0	inside	    91   133
PrapOR29	TMHMM2.0	TMhelix	   134   156
PrapOR29	TMHMM2.0	outside	   157   185
PrapOR29	TMHMM2.0	TMhelix	   186   208
PrapOR29	TMHMM2.0	inside	   209   296
PrapOR29	TMHMM2.0	TMhelix	   297   319
PrapOR29	TMHMM2.0	outside	   320   349
PrapOR29	TMHMM2.0	TMhelix	   350   372
PrapOR29	TMHMM2.0	inside	   373   394
```

# plot in postscript,
script for making the plot in gnuplot,
data for plot

---

```
# PrapOR32 Length: 390
# PrapOR32 Number of predicted TMHs:  6
# PrapOR32 Exp number of AAs in TMHs: 140.09277
# PrapOR32 Exp number, first 60 AAs:  27.03606
# PrapOR32 Total prob of N-in:        0.69939
# PrapOR32 POSSIBLE N-term signal sequence
PrapOR32	TMHMM2.0	inside	     1    34
PrapOR32	TMHMM2.0	TMhelix	    35    57
PrapOR32	TMHMM2.0	outside	    58    60
PrapOR32	TMHMM2.0	TMhelix	    61    83
PrapOR32	TMHMM2.0	inside	    84   132
PrapOR32	TMHMM2.0	TMhelix	   133   155
PrapOR32	TMHMM2.0	outside	   156   187
PrapOR32	TMHMM2.0	TMhelix	   188   207
PrapOR32	TMHMM2.0	inside	   208   266
PrapOR32	TMHMM2.0	TMhelix	   267   289
PrapOR32	TMHMM2.0	outside	   290   298
PrapOR32	TMHMM2.0	TMhelix	   299   321
PrapOR32	TMHMM2.0	inside	   322   390
```

# plot in postscript,
script for making the plot in gnuplot,
data for plot

---

```
# PrapOR33 Length: 366
# PrapOR33 Number of predicted TMHs:  7
# PrapOR33 Exp number of AAs in TMHs: 151.43024
# PrapOR33 Exp number, first 60 AAs:  34.2322
# PrapOR33 Total prob of N-in:        0.99956
# PrapOR33 POSSIBLE N-term signal sequence
PrapOR33	TMHMM2.0	inside	     1    12
PrapOR33	TMHMM2.0	TMhelix	    13    30
PrapOR33	TMHMM2.0	outside	    31    44
PrapOR33	TMHMM2.0	TMhelix	    45    67
PrapOR33	TMHMM2.0	inside	    68   110
PrapOR33	TMHMM2.0	TMhelix	   111   133
PrapOR33	TMHMM2.0	outside	   134   167
PrapOR33	TMHMM2.0	TMhelix	   168   190
PrapOR33	TMHMM2.0	inside	   191   241
PrapOR33	TMHMM2.0	TMhelix	   242   261
PrapOR33	TMHMM2.0	outside	   262   270
PrapOR33	TMHMM2.0	TMhelix	   271   293
PrapOR33	TMHMM2.0	inside	   294   336
PrapOR33	TMHMM2.0	TMhelix	   337   359
PrapOR33	TMHMM2.0	outside	   360   366
```

# plot in postscript,
script for making the plot in gnuplot,
data for plot

---

```
# PrapOR11 Length: 394
# PrapOR11 Number of predicted TMHs:  6
# PrapOR11 Exp number of AAs in TMHs: 134.27223
# PrapOR11 Exp number, first 60 AAs:  22.33604
# PrapOR11 Total prob of N-in:        0.87051
# PrapOR11 POSSIBLE N-term signal sequence
PrapOR11	TMHMM2.0	inside	     1    40
PrapOR11	TMHMM2.0	TMhelix	    41    63
PrapOR11	TMHMM2.0	outside	    64    72
PrapOR11	TMHMM2.0	TMhelix	    73    95
PrapOR11	TMHMM2.0	inside	    96   139
PrapOR11	TMHMM2.0	TMhelix	   140   162
PrapOR11	TMHMM2.0	outside	   163   198
PrapOR11	TMHMM2.0	TMhelix	   199   221
PrapOR11	TMHMM2.0	inside	   222   270
PrapOR11	TMHMM2.0	TMhelix	   271   293
PrapOR11	TMHMM2.0	outside	   294   302
PrapOR11	TMHMM2.0	TMhelix	   303   322
PrapOR11	TMHMM2.0	inside	   323   394
```

# plot in postscript,
script for making the plot in gnuplot,
data for plot

---

```
# PrapOR56 Length: 420
# PrapOR56 Number of predicted TMHs:  6
# PrapOR56 Exp number of AAs in TMHs: 140.84273
# PrapOR56 Exp number, first 60 AAs:  23.88722
# PrapOR56 Total prob of N-in:        0.86674
# PrapOR56 POSSIBLE N-term signal sequence
PrapOR56	TMHMM2.0	inside	     1    33
PrapOR56	TMHMM2.0	TMhelix	    34    56
PrapOR56	TMHMM2.0	outside	    57    70
PrapOR56	TMHMM2.0	TMhelix	    71    93
PrapOR56	TMHMM2.0	inside	    94   127
PrapOR56	TMHMM2.0	TMhelix	   128   150
PrapOR56	TMHMM2.0	outside	   151   195
PrapOR56	TMHMM2.0	TMhelix	   196   218
PrapOR56	TMHMM2.0	inside	   219   290
PrapOR56	TMHMM2.0	TMhelix	   291   313
PrapOR56	TMHMM2.0	outside	   314   322
PrapOR56	TMHMM2.0	TMhelix	   323   345
PrapOR56	TMHMM2.0	inside	   346   420
```

# plot in postscript,
script for making the plot in gnuplot,
data for plot

---

```
# PrapOR34 Length: 372
# PrapOR34 Number of predicted TMHs:  5
# PrapOR34 Exp number of AAs in TMHs: 126.07932
# PrapOR34 Exp number, first 60 AAs:  21.34986
# PrapOR34 Total prob of N-in:        0.94808
# PrapOR34 POSSIBLE N-term signal sequence
PrapOR34	TMHMM2.0	inside	     1    37
PrapOR34	TMHMM2.0	TMhelix	    38    60
PrapOR34	TMHMM2.0	outside	    61   114
PrapOR34	TMHMM2.0	TMhelix	   115   137
PrapOR34	TMHMM2.0	inside	   138   171
PrapOR34	TMHMM2.0	TMhelix	   172   194
PrapOR34	TMHMM2.0	outside	   195   245
PrapOR34	TMHMM2.0	TMhelix	   246   268
PrapOR34	TMHMM2.0	inside	   269   274
PrapOR34	TMHMM2.0	TMhelix	   275   297
PrapOR34	TMHMM2.0	outside	   298   372
```

# plot in postscript,
script for making the plot in gnuplot,
data for plot

---

```
# PrapOR14 Length: 393
# PrapOR14 Number of predicted TMHs:  6
# PrapOR14 Exp number of AAs in TMHs: 130.37123
# PrapOR14 Exp number, first 60 AAs:  22.47788
# PrapOR14 Total prob of N-in:        0.96132
# PrapOR14 POSSIBLE N-term signal sequence
PrapOR14	TMHMM2.0	inside	     1    37
PrapOR14	TMHMM2.0	TMhelix	    38    60
PrapOR14	TMHMM2.0	outside	    61    74
PrapOR14	TMHMM2.0	TMhelix	    75    94
PrapOR14	TMHMM2.0	inside	    95   127
PrapOR14	TMHMM2.0	TMhelix	   128   150
PrapOR14	TMHMM2.0	outside	   151   185
PrapOR14	TMHMM2.0	TMhelix	   186   208
PrapOR14	TMHMM2.0	inside	   209   265
PrapOR14	TMHMM2.0	TMhelix	   266   288
PrapOR14	TMHMM2.0	outside	   289   292
PrapOR14	TMHMM2.0	TMhelix	   293   315
PrapOR14	TMHMM2.0	inside	   316   393
```

# plot in postscript,
script for making the plot in gnuplot,
data for plot

---

```
# PrapOR58 Length: 397
# PrapOR58 Number of predicted TMHs:  7
# PrapOR58 Exp number of AAs in TMHs: 147.39202
# PrapOR58 Exp number, first 60 AAs:  20.2472
# PrapOR58 Total prob of N-in:        0.99582
# PrapOR58 POSSIBLE N-term signal sequence
PrapOR58	TMHMM2.0	inside	     1    39
PrapOR58	TMHMM2.0	TMhelix	    40    62
PrapOR58	TMHMM2.0	outside	    63    71
PrapOR58	TMHMM2.0	TMhelix	    72    91
PrapOR58	TMHMM2.0	inside	    92   126
PrapOR58	TMHMM2.0	TMhelix	   127   146
PrapOR58	TMHMM2.0	outside	   147   181
PrapOR58	TMHMM2.0	TMhelix	   182   204
PrapOR58	TMHMM2.0	inside	   205   261
PrapOR58	TMHMM2.0	TMhelix	   262   284
PrapOR58	TMHMM2.0	outside	   285   293
PrapOR58	TMHMM2.0	TMhelix	   294   316
PrapOR58	TMHMM2.0	inside	   317   370
PrapOR58	TMHMM2.0	TMhelix	   371   393
PrapOR58	TMHMM2.0	outside	   394   397
```

# plot in postscript,
script for making the plot in gnuplot,
data for plot

---

```
# PrapOR57 Length: 397
# PrapOR57 Number of predicted TMHs:  6
# PrapOR57 Exp number of AAs in TMHs: 147.62583
# PrapOR57 Exp number, first 60 AAs:  20.84086
# PrapOR57 Total prob of N-in:        0.99103
# PrapOR57 POSSIBLE N-term signal sequence
PrapOR57	TMHMM2.0	inside	     1    39
PrapOR57	TMHMM2.0	TMhelix	    40    62
PrapOR57	TMHMM2.0	outside	    63    71
PrapOR57	TMHMM2.0	TMhelix	    72    91
PrapOR57	TMHMM2.0	inside	    92   124
PrapOR57	TMHMM2.0	TMhelix	   125   143
PrapOR57	TMHMM2.0	outside	   144   180
PrapOR57	TMHMM2.0	TMhelix	   181   203
PrapOR57	TMHMM2.0	inside	   204   260
PrapOR57	TMHMM2.0	TMhelix	   261   283
PrapOR57	TMHMM2.0	outside	   284   292
PrapOR57	TMHMM2.0	TMhelix	   293   315
PrapOR57	TMHMM2.0	inside	   316   397
```

# plot in postscript,
script for making the plot in gnuplot,
data for plot

---

```
# PrapOR17 Length: 390
# PrapOR17 Number of predicted TMHs:  6
# PrapOR17 Exp number of AAs in TMHs: 142.37659
# PrapOR17 Exp number, first 60 AAs:  16.12137
# PrapOR17 Total prob of N-in:        0.99891
# PrapOR17 POSSIBLE N-term signal sequence
PrapOR17	TMHMM2.0	inside	     1    41
PrapOR17	TMHMM2.0	TMhelix	    42    64
PrapOR17	TMHMM2.0	outside	    65    78
PrapOR17	TMHMM2.0	TMhelix	    79    98
PrapOR17	TMHMM2.0	inside	    99   126
PrapOR17	TMHMM2.0	TMhelix	   127   149
PrapOR17	TMHMM2.0	outside	   150   185
PrapOR17	TMHMM2.0	TMhelix	   186   208
PrapOR17	TMHMM2.0	inside	   209   254
PrapOR17	TMHMM2.0	TMhelix	   255   277
PrapOR17	TMHMM2.0	outside	   278   286
PrapOR17	TMHMM2.0	TMhelix	   287   309
PrapOR17	TMHMM2.0	inside	   310   390
```

# plot in postscript,
script for making the plot in gnuplot,
data for plot

---

```
# PrapOR3 Length: 405
# PrapOR3 Number of predicted TMHs:  5
# PrapOR3 Exp number of AAs in TMHs: 130.59046
# PrapOR3 Exp number, first 60 AAs:  28.72638
# PrapOR3 Total prob of N-in:        0.37213
# PrapOR3 POSSIBLE N-term signal sequence
PrapOR3	TMHMM2.0	inside	     1    44
PrapOR3	TMHMM2.0	TMhelix	    45    67
PrapOR3	TMHMM2.0	outside	    68    76
PrapOR3	TMHMM2.0	TMhelix	    77    99
PrapOR3	TMHMM2.0	inside	   100   137
PrapOR3	TMHMM2.0	TMhelix	   138   160
PrapOR3	TMHMM2.0	outside	   161   194
PrapOR3	TMHMM2.0	TMhelix	   195   217
PrapOR3	TMHMM2.0	inside	   218   304
PrapOR3	TMHMM2.0	TMhelix	   305   327
PrapOR3	TMHMM2.0	outside	   328   405
```

# plot in postscript,
script for making the plot in gnuplot,
data for plot

---

```
# PrapOR37 Length: 410
# PrapOR37 Number of predicted TMHs:  7
# PrapOR37 Exp number of AAs in TMHs: 156.4505
# PrapOR37 Exp number, first 60 AAs:  23.0362
# PrapOR37 Total prob of N-in:        0.98192
# PrapOR37 POSSIBLE N-term signal sequence
PrapOR37	TMHMM2.0	inside	     1    29
PrapOR37	TMHMM2.0	TMhelix	    30    52
PrapOR37	TMHMM2.0	outside	    53    61
PrapOR37	TMHMM2.0	TMhelix	    62    84
PrapOR37	TMHMM2.0	inside	    85   144
PrapOR37	TMHMM2.0	TMhelix	   145   167
PrapOR37	TMHMM2.0	outside	   168   190
PrapOR37	TMHMM2.0	TMhelix	   191   213
PrapOR37	TMHMM2.0	inside	   214   284
PrapOR37	TMHMM2.0	TMhelix	   285   307
PrapOR37	TMHMM2.0	outside	   308   316
PrapOR37	TMHMM2.0	TMhelix	   317   335
PrapOR37	TMHMM2.0	inside	   336   376
PrapOR37	TMHMM2.0	TMhelix	   377   394
PrapOR37	TMHMM2.0	outside	   395   410
```

# plot in postscript,
script for making the plot in gnuplot,
data for plot

---

```
# PrapOR20 Length: 392
# PrapOR20 Number of predicted TMHs:  6
# PrapOR20 Exp number of AAs in TMHs: 131.32572
# PrapOR20 Exp number, first 60 AAs:  8.20287
# PrapOR20 Total prob of N-in:        0.98729
PrapOR20	TMHMM2.0	inside	     1    54
PrapOR20	TMHMM2.0	TMhelix	    55    72
PrapOR20	TMHMM2.0	outside	    73    81
PrapOR20	TMHMM2.0	TMhelix	    82   101
PrapOR20	TMHMM2.0	inside	   102   139
PrapOR20	TMHMM2.0	TMhelix	   140   162
PrapOR20	TMHMM2.0	outside	   163   176
PrapOR20	TMHMM2.0	TMhelix	   177   199
PrapOR20	TMHMM2.0	inside	   200   205
PrapOR20	TMHMM2.0	TMhelix	   206   228
PrapOR20	TMHMM2.0	outside	   229   267
PrapOR20	TMHMM2.0	TMhelix	   268   290
PrapOR20	TMHMM2.0	inside	   291   392
```

# plot in postscript,
script for making the plot in gnuplot,
data for plot

---

```
# PrapOR1 Length: 389
# PrapOR1 Number of predicted TMHs:  6
# PrapOR1 Exp number of AAs in TMHs: 146.11481
# PrapOR1 Exp number, first 60 AAs:  38.01021
# PrapOR1 Total prob of N-in:        0.98789
# PrapOR1 POSSIBLE N-term signal sequence
PrapOR1	TMHMM2.0	inside	     1    11
PrapOR1	TMHMM2.0	TMhelix	    12    34
PrapOR1	TMHMM2.0	outside	    35    43
PrapOR1	TMHMM2.0	TMhelix	    44    66
PrapOR1	TMHMM2.0	inside	    67   101
PrapOR1	TMHMM2.0	TMhelix	   102   124
PrapOR1	TMHMM2.0	outside	   125   169
PrapOR1	TMHMM2.0	TMhelix	   170   192
PrapOR1	TMHMM2.0	inside	   193   258
PrapOR1	TMHMM2.0	TMhelix	   259   281
PrapOR1	TMHMM2.0	outside	   282   295
PrapOR1	TMHMM2.0	TMhelix	   296   318
PrapOR1	TMHMM2.0	inside	   319   389
```

# plot in postscript,
script for making the plot in gnuplot,
data for plot

---

```
# PrapOR13 Length: 387
# PrapOR13 Number of predicted TMHs:  7
# PrapOR13 Exp number of AAs in TMHs: 151.89582
# PrapOR13 Exp number, first 60 AAs:  22.21447
# PrapOR13 Total prob of N-in:        0.36173
# PrapOR13 POSSIBLE N-term signal sequence
PrapOR13	TMHMM2.0	outside	     1    43
PrapOR13	TMHMM2.0	TMhelix	    44    66
PrapOR13	TMHMM2.0	inside	    67    77
PrapOR13	TMHMM2.0	TMhelix	    78   100
PrapOR13	TMHMM2.0	outside	   101   138
PrapOR13	TMHMM2.0	TMhelix	   139   156
PrapOR13	TMHMM2.0	inside	   157   187
PrapOR13	TMHMM2.0	TMhelix	   188   210
PrapOR13	TMHMM2.0	outside	   211   266
PrapOR13	TMHMM2.0	TMhelix	   267   289
PrapOR13	TMHMM2.0	inside	   290   293
PrapOR13	TMHMM2.0	TMhelix	   294   316
PrapOR13	TMHMM2.0	outside	   317   362
PrapOR13	TMHMM2.0	TMhelix	   363   385
PrapOR13	TMHMM2.0	inside	   386   387
```

# plot in postscript,
script for making the plot in gnuplot,
data for plot

---

```
# PrapOR12 Length: 388
# PrapOR12 Number of predicted TMHs:  5
# PrapOR12 Exp number of AAs in TMHs: 128.30548
# PrapOR12 Exp number, first 60 AAs:  18.03164
# PrapOR12 Total prob of N-in:        0.58174
# PrapOR12 POSSIBLE N-term signal sequence
PrapOR12	TMHMM2.0	inside	     1    46
PrapOR12	TMHMM2.0	TMhelix	    47    69
PrapOR12	TMHMM2.0	outside	    70    83
PrapOR12	TMHMM2.0	TMhelix	    84   101
PrapOR12	TMHMM2.0	inside	   102   267
PrapOR12	TMHMM2.0	TMhelix	   268   290
PrapOR12	TMHMM2.0	outside	   291   293
PrapOR12	TMHMM2.0	TMhelix	   294   316
PrapOR12	TMHMM2.0	inside	   317   363
PrapOR12	TMHMM2.0	TMhelix	   364   386
PrapOR12	TMHMM2.0	outside	   387   388
```

# plot in postscript,
script for making the plot in gnuplot,
data for plot

---

```
# PrapOR24 Length: 408
# PrapOR24 Number of predicted TMHs:  6
# PrapOR24 Exp number of AAs in TMHs: 145.87917
# PrapOR24 Exp number, first 60 AAs:  27.51253
# PrapOR24 Total prob of N-in:        0.44335
# PrapOR24 POSSIBLE N-term signal sequence
PrapOR24	TMHMM2.0	inside	     1    43
PrapOR24	TMHMM2.0	TMhelix	    44    66
PrapOR24	TMHMM2.0	outside	    67    80
PrapOR24	TMHMM2.0	TMhelix	    81   103
PrapOR24	TMHMM2.0	inside	   104   142
PrapOR24	TMHMM2.0	TMhelix	   143   165
PrapOR24	TMHMM2.0	outside	   166   196
PrapOR24	TMHMM2.0	TMhelix	   197   219
PrapOR24	TMHMM2.0	inside	   220   286
PrapOR24	TMHMM2.0	TMhelix	   287   309
PrapOR24	TMHMM2.0	outside	   310   373
PrapOR24	TMHMM2.0	TMhelix	   374   396
PrapOR24	TMHMM2.0	inside	   397   408
```

# plot in postscript,
script for making the plot in gnuplot,
data for plot

---

```
# PrapOR25 Length: 411
# PrapOR25 Number of predicted TMHs:  5
# PrapOR25 Exp number of AAs in TMHs: 123.06648
# PrapOR25 Exp number, first 60 AAs:  10.44039
# PrapOR25 Total prob of N-in:        0.99730
# PrapOR25 POSSIBLE N-term signal sequence
PrapOR25	TMHMM2.0	inside	     1    48
PrapOR25	TMHMM2.0	TMhelix	    49    71
PrapOR25	TMHMM2.0	outside	    72    85
PrapOR25	TMHMM2.0	TMhelix	    86   108
PrapOR25	TMHMM2.0	inside	   109   150
PrapOR25	TMHMM2.0	TMhelix	   151   173
PrapOR25	TMHMM2.0	outside	   174   197
PrapOR25	TMHMM2.0	TMhelix	   198   220
PrapOR25	TMHMM2.0	inside	   221   296
PrapOR25	TMHMM2.0	TMhelix	   297   319
PrapOR25	TMHMM2.0	outside	   320   411
```

# plot in postscript,
script for making the plot in gnuplot,
data for plot

---

```
# PrapOR26 Length: 405
# PrapOR26 Number of predicted TMHs:  5
# PrapOR26 Exp number of AAs in TMHs: 130.25546
# PrapOR26 Exp number, first 60 AAs:  16.92096
# PrapOR26 Total prob of N-in:        0.99877
# PrapOR26 POSSIBLE N-term signal sequence
PrapOR26	TMHMM2.0	inside	     1    43
PrapOR26	TMHMM2.0	TMhelix	    44    66
PrapOR26	TMHMM2.0	outside	    67    80
PrapOR26	TMHMM2.0	TMhelix	    81   103
PrapOR26	TMHMM2.0	inside	   104   141
PrapOR26	TMHMM2.0	TMhelix	   142   164
PrapOR26	TMHMM2.0	outside	   165   188
PrapOR26	TMHMM2.0	TMhelix	   189   211
PrapOR26	TMHMM2.0	inside	   212   292
PrapOR26	TMHMM2.0	TMhelix	   293   315
PrapOR26	TMHMM2.0	outside	   316   405
```

# plot in postscript,
script for making the plot in gnuplot,
data for plot

---

```
# PrapOR27 Length: 406
# PrapOR27 Number of predicted TMHs:  4
# PrapOR27 Exp number of AAs in TMHs: 118.72553
# PrapOR27 Exp number, first 60 AAs:  19.61538
# PrapOR27 Total prob of N-in:        0.99730
# PrapOR27 POSSIBLE N-term signal sequence
PrapOR27	TMHMM2.0	inside	     1    39
PrapOR27	TMHMM2.0	TMhelix	    40    62
PrapOR27	TMHMM2.0	outside	    63    76
PrapOR27	TMHMM2.0	TMhelix	    77    99
PrapOR27	TMHMM2.0	inside	   100   141
PrapOR27	TMHMM2.0	TMhelix	   142   164
PrapOR27	TMHMM2.0	outside	   165   186
PrapOR27	TMHMM2.0	TMhelix	   187   209
PrapOR27	TMHMM2.0	inside	   210   406
```

# plot in postscript,
script for making the plot in gnuplot,
data for plot

---

```
# PrapOR18 Length: 404
# PrapOR18 Number of predicted TMHs:  4
# PrapOR18 Exp number of AAs in TMHs: 132.36107
# PrapOR18 Exp number, first 60 AAs:  16.03949
# PrapOR18 Total prob of N-in:        0.99927
# PrapOR18 POSSIBLE N-term signal sequence
PrapOR18	TMHMM2.0	inside	     1    45
PrapOR18	TMHMM2.0	TMhelix	    46    68
PrapOR18	TMHMM2.0	outside	    69    77
PrapOR18	TMHMM2.0	TMhelix	    78   100
PrapOR18	TMHMM2.0	inside	   101   139
PrapOR18	TMHMM2.0	TMhelix	   140   162
PrapOR18	TMHMM2.0	outside	   163   186
PrapOR18	TMHMM2.0	TMhelix	   187   209
PrapOR18	TMHMM2.0	inside	   210   404
```

# plot in postscript,
script for making the plot in gnuplot,
data for plot

---

```
# PrapOR19 Length: 407
# PrapOR19 Number of predicted TMHs:  5
# PrapOR19 Exp number of AAs in TMHs: 114.72473
# PrapOR19 Exp number, first 60 AAs:  23.40851
# PrapOR19 Total prob of N-in:        0.92994
# PrapOR19 POSSIBLE N-term signal sequence
PrapOR19	TMHMM2.0	inside	     1    36
PrapOR19	TMHMM2.0	TMhelix	    37    59
PrapOR19	TMHMM2.0	outside	    60    63
PrapOR19	TMHMM2.0	TMhelix	    64    86
PrapOR19	TMHMM2.0	inside	    87   130
PrapOR19	TMHMM2.0	TMhelix	   131   153
PrapOR19	TMHMM2.0	outside	   154   180
PrapOR19	TMHMM2.0	TMhelix	   181   203
PrapOR19	TMHMM2.0	inside	   204   276
PrapOR19	TMHMM2.0	TMhelix	   277   299
PrapOR19	TMHMM2.0	outside	   300   407
```

# plot in postscript,
script for making the plot in gnuplot,
data for plot

---

```
# PrapOR38 Length: 391
# PrapOR38 Number of predicted TMHs:  6
# PrapOR38 Exp number of AAs in TMHs: 132.06597
# PrapOR38 Exp number, first 60 AAs:  23.90376
# PrapOR38 Total prob of N-in:        0.65083
# PrapOR38 POSSIBLE N-term signal sequence
PrapOR38	TMHMM2.0	inside	     1    36
PrapOR38	TMHMM2.0	TMhelix	    37    56
PrapOR38	TMHMM2.0	outside	    57    65
PrapOR38	TMHMM2.0	TMhelix	    66    88
PrapOR38	TMHMM2.0	inside	    89   130
PrapOR38	TMHMM2.0	TMhelix	   131   153
PrapOR38	TMHMM2.0	outside	   154   162
PrapOR38	TMHMM2.0	TMhelix	   163   185
PrapOR38	TMHMM2.0	inside	   186   189
PrapOR38	TMHMM2.0	TMhelix	   190   212
PrapOR38	TMHMM2.0	outside	   213   284
PrapOR38	TMHMM2.0	TMhelix	   285   307
PrapOR38	TMHMM2.0	inside	   308   391
```

# plot in postscript,
script for making the plot in gnuplot,
data for plot

---

```
# PrapOR40 Length: 395
# PrapOR40 Number of predicted TMHs:  6
# PrapOR40 Exp number of AAs in TMHs: 136.92199
# PrapOR40 Exp number, first 60 AAs:  22.09772
# PrapOR40 Total prob of N-in:        0.91503
# PrapOR40 POSSIBLE N-term signal sequence
PrapOR40	TMHMM2.0	inside	     1    34
PrapOR40	TMHMM2.0	TMhelix	    35    57
PrapOR40	TMHMM2.0	outside	    58    69
PrapOR40	TMHMM2.0	TMhelix	    70    92
PrapOR40	TMHMM2.0	inside	    93   138
PrapOR40	TMHMM2.0	TMhelix	   139   161
PrapOR40	TMHMM2.0	outside	   162   196
PrapOR40	TMHMM2.0	TMhelix	   197   219
PrapOR40	TMHMM2.0	inside	   220   270
PrapOR40	TMHMM2.0	TMhelix	   271   293
PrapOR40	TMHMM2.0	outside	   294   297
PrapOR40	TMHMM2.0	TMhelix	   298   320
PrapOR40	TMHMM2.0	inside	   321   395
```

# plot in postscript,
script for making the plot in gnuplot,
data for plot

---

```
# PrapOR41 Length: 392
# PrapOR41 Number of predicted TMHs:  7
# PrapOR41 Exp number of AAs in TMHs: 147.85593
# PrapOR41 Exp number, first 60 AAs:  22.81413
# PrapOR41 Total prob of N-in:        0.99976
# PrapOR41 POSSIBLE N-term signal sequence
PrapOR41	TMHMM2.0	inside	     1    34
PrapOR41	TMHMM2.0	TMhelix	    35    57
PrapOR41	TMHMM2.0	outside	    58    66
PrapOR41	TMHMM2.0	TMhelix	    67    89
PrapOR41	TMHMM2.0	inside	    90   133
PrapOR41	TMHMM2.0	TMhelix	   134   156
PrapOR41	TMHMM2.0	outside	   157   192
PrapOR41	TMHMM2.0	TMhelix	   193   215
PrapOR41	TMHMM2.0	inside	   216   265
PrapOR41	TMHMM2.0	TMhelix	   266   288
PrapOR41	TMHMM2.0	outside	   289   291
PrapOR41	TMHMM2.0	TMhelix	   292   314
PrapOR41	TMHMM2.0	inside	   315   365
PrapOR41	TMHMM2.0	TMhelix	   366   385
PrapOR41	TMHMM2.0	outside	   386   392
```

# plot in postscript,
script for making the plot in gnuplot,
data for plot

---

```
# PrapOR15 Length: 392
# PrapOR15 Number of predicted TMHs:  6
# PrapOR15 Exp number of AAs in TMHs: 131.25816
# PrapOR15 Exp number, first 60 AAs:  22.11284
# PrapOR15 Total prob of N-in:        0.99469
# PrapOR15 POSSIBLE N-term signal sequence
PrapOR15	TMHMM2.0	inside	     1    37
PrapOR15	TMHMM2.0	TMhelix	    38    60
PrapOR15	TMHMM2.0	outside	    61    69
PrapOR15	TMHMM2.0	TMhelix	    70    89
PrapOR15	TMHMM2.0	inside	    90   132
PrapOR15	TMHMM2.0	TMhelix	   133   155
PrapOR15	TMHMM2.0	outside	   156   197
PrapOR15	TMHMM2.0	TMhelix	   198   220
PrapOR15	TMHMM2.0	inside	   221   265
PrapOR15	TMHMM2.0	TMhelix	   266   288
PrapOR15	TMHMM2.0	outside	   289   297
PrapOR15	TMHMM2.0	TMhelix	   298   315
PrapOR15	TMHMM2.0	inside	   316   392
```

# plot in postscript,
script for making the plot in gnuplot,
data for plot

---

```
# PrapOR16 Length: 395
# PrapOR16 Number of predicted TMHs:  6
# PrapOR16 Exp number of AAs in TMHs: 137.91662
# PrapOR16 Exp number, first 60 AAs:  18.09983
# PrapOR16 Total prob of N-in:        0.31824
# PrapOR16 POSSIBLE N-term signal sequence
PrapOR16	TMHMM2.0	outside	     1    41
PrapOR16	TMHMM2.0	TMhelix	    42    64
PrapOR16	TMHMM2.0	inside	    65   138
PrapOR16	TMHMM2.0	TMhelix	   139   161
PrapOR16	TMHMM2.0	outside	   162   175
PrapOR16	TMHMM2.0	TMhelix	   176   198
PrapOR16	TMHMM2.0	inside	   199   204
PrapOR16	TMHMM2.0	TMhelix	   205   227
PrapOR16	TMHMM2.0	outside	   228   270
PrapOR16	TMHMM2.0	TMhelix	   271   293
PrapOR16	TMHMM2.0	inside	   294   299
PrapOR16	TMHMM2.0	TMhelix	   300   319
PrapOR16	TMHMM2.0	outside	   320   395
```

# plot in postscript,
script for making the plot in gnuplot,
data for plot

---

```
# PrapOR35 Length: 399
# PrapOR35 Number of predicted TMHs:  5
# PrapOR35 Exp number of AAs in TMHs: 130.11049
# PrapOR35 Exp number, first 60 AAs:  19.54284
# PrapOR35 Total prob of N-in:        0.77379
# PrapOR35 POSSIBLE N-term signal sequence
PrapOR35	TMHMM2.0	inside	     1    41
PrapOR35	TMHMM2.0	TMhelix	    42    64
PrapOR35	TMHMM2.0	outside	    65   139
PrapOR35	TMHMM2.0	TMhelix	   140   162
PrapOR35	TMHMM2.0	inside	   163   192
PrapOR35	TMHMM2.0	TMhelix	   193   215
PrapOR35	TMHMM2.0	outside	   216   273
PrapOR35	TMHMM2.0	TMhelix	   274   296
PrapOR35	TMHMM2.0	inside	   297   376
PrapOR35	TMHMM2.0	TMhelix	   377   396
PrapOR35	TMHMM2.0	outside	   397   399
```

# plot in postscript,
script for making the plot in gnuplot,
data for plot

---

```
# PrapOR7 Length: 400
# PrapOR7 Number of predicted TMHs:  7
# PrapOR7 Exp number of AAs in TMHs: 149.67148
# PrapOR7 Exp number, first 60 AAs:  18.87908
# PrapOR7 Total prob of N-in:        0.97207
# PrapOR7 POSSIBLE N-term signal sequence
PrapOR7	TMHMM2.0	inside	     1    39
PrapOR7	TMHMM2.0	TMhelix	    40    59
PrapOR7	TMHMM2.0	outside	    60    73
PrapOR7	TMHMM2.0	TMhelix	    74    96
PrapOR7	TMHMM2.0	inside	    97   134
PrapOR7	TMHMM2.0	TMhelix	   135   152
PrapOR7	TMHMM2.0	outside	   153   200
PrapOR7	TMHMM2.0	TMhelix	   201   223
PrapOR7	TMHMM2.0	inside	   224   270
PrapOR7	TMHMM2.0	TMhelix	   271   293
PrapOR7	TMHMM2.0	outside	   294   297
PrapOR7	TMHMM2.0	TMhelix	   298   320
PrapOR7	TMHMM2.0	inside	   321   360
PrapOR7	TMHMM2.0	TMhelix	   361   383
PrapOR7	TMHMM2.0	outside	   384   400
```

# plot in postscript,
script for making the plot in gnuplot,
data for plot

---

```
# PrapOR8 Length: 399
# PrapOR8 Number of predicted TMHs:  4
# PrapOR8 Exp number of AAs in TMHs: 127.56453
# PrapOR8 Exp number, first 60 AAs:  19.18184
# PrapOR8 Total prob of N-in:        0.92950
# PrapOR8 POSSIBLE N-term signal sequence
PrapOR8	TMHMM2.0	inside	     1    42
PrapOR8	TMHMM2.0	TMhelix	    43    65
PrapOR8	TMHMM2.0	outside	    66    74
PrapOR8	TMHMM2.0	TMhelix	    75    97
PrapOR8	TMHMM2.0	inside	    98   273
PrapOR8	TMHMM2.0	TMhelix	   274   296
PrapOR8	TMHMM2.0	outside	   297   372
PrapOR8	TMHMM2.0	TMhelix	   373   395
PrapOR8	TMHMM2.0	inside	   396   399
```

# plot in postscript,
script for making the plot in gnuplot,
data for plot

---

```
# PrapOR9 Length: 400
# PrapOR9 Number of predicted TMHs:  6
# PrapOR9 Exp number of AAs in TMHs: 145.71366
# PrapOR9 Exp number, first 60 AAs:  17.22497
# PrapOR9 Total prob of N-in:        0.93082
# PrapOR9 POSSIBLE N-term signal sequence
PrapOR9	TMHMM2.0	inside	     1    42
PrapOR9	TMHMM2.0	TMhelix	    43    65
PrapOR9	TMHMM2.0	outside	    66    74
PrapOR9	TMHMM2.0	TMhelix	    75    97
PrapOR9	TMHMM2.0	inside	    98   140
PrapOR9	TMHMM2.0	TMhelix	   141   163
PrapOR9	TMHMM2.0	outside	   164   204
PrapOR9	TMHMM2.0	TMhelix	   205   227
PrapOR9	TMHMM2.0	inside	   228   274
PrapOR9	TMHMM2.0	TMhelix	   275   297
PrapOR9	TMHMM2.0	outside	   298   363
PrapOR9	TMHMM2.0	TMhelix	   364   383
PrapOR9	TMHMM2.0	inside	   384   400
```

# plot in postscript,
script for making the plot in gnuplot,
data for plot

---

```
# PrapOrco Length: 471
# PrapOrco Number of predicted TMHs:  7
# PrapOrco Exp number of AAs in TMHs: 155.61751
# PrapOrco Exp number, first 60 AAs:  15.2314
# PrapOrco Total prob of N-in:        0.97922
# PrapOrco POSSIBLE N-term signal sequence
PrapOrco	TMHMM2.0	inside	     1    45
PrapOrco	TMHMM2.0	TMhelix	    46    65
PrapOrco	TMHMM2.0	outside	    66    74
PrapOrco	TMHMM2.0	TMhelix	    75    97
PrapOrco	TMHMM2.0	inside	    98   135
PrapOrco	TMHMM2.0	TMhelix	   136   158
PrapOrco	TMHMM2.0	outside	   159   194
PrapOrco	TMHMM2.0	TMhelix	   195   217
PrapOrco	TMHMM2.0	inside	   218   346
PrapOrco	TMHMM2.0	TMhelix	   347   364
PrapOrco	TMHMM2.0	outside	   365   373
PrapOrco	TMHMM2.0	TMhelix	   374   396
PrapOrco	TMHMM2.0	inside	   397   444
PrapOrco	TMHMM2.0	TMhelix	   445   467
PrapOrco	TMHMM2.0	outside	   468   471
```

# plot in postscript,
script for making the plot in gnuplot,
data for plot

---

```
# PrapOR47 Length: 417
# PrapOR47 Number of predicted TMHs:  6
# PrapOR47 Exp number of AAs in TMHs: 131.17647
# PrapOR47 Exp number, first 60 AAs:  19.982
# PrapOR47 Total prob of N-in:        0.97409
# PrapOR47 POSSIBLE N-term signal sequence
PrapOR47	TMHMM2.0	inside	     1    39
PrapOR47	TMHMM2.0	TMhelix	    40    62
PrapOR47	TMHMM2.0	outside	    63    76
PrapOR47	TMHMM2.0	TMhelix	    77    96
PrapOR47	TMHMM2.0	inside	    97   133
PrapOR47	TMHMM2.0	TMhelix	   134   156
PrapOR47	TMHMM2.0	outside	   157   189
PrapOR47	TMHMM2.0	TMhelix	   190   212
PrapOR47	TMHMM2.0	inside	   213   315
PrapOR47	TMHMM2.0	TMhelix	   316   338
PrapOR47	TMHMM2.0	outside	   339   347
PrapOR47	TMHMM2.0	TMhelix	   348   370
PrapOR47	TMHMM2.0	inside	   371   417
```

# plot in postscript,
script for making the plot in gnuplot,
data for plot

---

```
# PrapOR59 Length: 434
# PrapOR59 Number of predicted TMHs:  4
# PrapOR59 Exp number of AAs in TMHs: 120.01396
# PrapOR59 Exp number, first 60 AAs:  4.20549
# PrapOR59 Total prob of N-in:        0.77540
PrapOR59	TMHMM2.0	outside	     1    55
PrapOR59	TMHMM2.0	TMhelix	    56    78
PrapOR59	TMHMM2.0	inside	    79   163
PrapOR59	TMHMM2.0	TMhelix	   164   186
PrapOR59	TMHMM2.0	outside	   187   210
PrapOR59	TMHMM2.0	TMhelix	   211   233
PrapOR59	TMHMM2.0	inside	   234   304
PrapOR59	TMHMM2.0	TMhelix	   305   327
PrapOR59	TMHMM2.0	outside	   328   434
```

# plot in postscript,
script for making the plot in gnuplot,
data for plot

---

```
# PrapOR63 Length: 380
# PrapOR63 Number of predicted TMHs:  6
# PrapOR63 Exp number of AAs in TMHs: 130.07987
# PrapOR63 Exp number, first 60 AAs:  19.83558
# PrapOR63 Total prob of N-in:        0.57599
# PrapOR63 POSSIBLE N-term signal sequence
PrapOR63	TMHMM2.0	inside	     1    38
PrapOR63	TMHMM2.0	TMhelix	    39    61
PrapOR63	TMHMM2.0	outside	    62    65
PrapOR63	TMHMM2.0	TMhelix	    66    83
PrapOR63	TMHMM2.0	inside	    84   123
PrapOR63	TMHMM2.0	TMhelix	   124   141
PrapOR63	TMHMM2.0	outside	   142   186
PrapOR63	TMHMM2.0	TMhelix	   187   209
PrapOR63	TMHMM2.0	inside	   210   255
PrapOR63	TMHMM2.0	TMhelix	   256   278
PrapOR63	TMHMM2.0	outside	   279   281
PrapOR63	TMHMM2.0	TMhelix	   282   304
PrapOR63	TMHMM2.0	inside	   305   380
```

# plot in postscript,
script for making the plot in gnuplot,
data for plot

---

```
# PrapOR61 Length: 395
# PrapOR61 Number of predicted TMHs:  6
# PrapOR61 Exp number of AAs in TMHs: 145.18944
# PrapOR61 Exp number, first 60 AAs:  18.3956
# PrapOR61 Total prob of N-in:        0.90310
# PrapOR61 POSSIBLE N-term signal sequence
PrapOR61	TMHMM2.0	inside	     1    40
PrapOR61	TMHMM2.0	TMhelix	    41    63
PrapOR61	TMHMM2.0	outside	    64    75
PrapOR61	TMHMM2.0	TMhelix	    76    93
PrapOR61	TMHMM2.0	inside	    94   132
PrapOR61	TMHMM2.0	TMhelix	   133   154
PrapOR61	TMHMM2.0	outside	   155   191
PrapOR61	TMHMM2.0	TMhelix	   192   223
PrapOR61	TMHMM2.0	inside	   224   269
PrapOR61	TMHMM2.0	TMhelix	   270   292
PrapOR61	TMHMM2.0	outside	   293   301
PrapOR61	TMHMM2.0	TMhelix	   302   324
PrapOR61	TMHMM2.0	inside	   325   395
```

# plot in postscript,
script for making the plot in gnuplot,
data for plot

---

```
# PrapOR62 Length: 396
# PrapOR62 Number of predicted TMHs:  7
# PrapOR62 Exp number of AAs in TMHs: 148.01185
# PrapOR62 Exp number, first 60 AAs:  22.15827
# PrapOR62 Total prob of N-in:        0.11113
# PrapOR62 POSSIBLE N-term signal sequence
PrapOR62	TMHMM2.0	outside	     1    34
PrapOR62	TMHMM2.0	TMhelix	    35    57
PrapOR62	TMHMM2.0	inside	    58    63
PrapOR62	TMHMM2.0	TMhelix	    64    86
PrapOR62	TMHMM2.0	outside	    87   127
PrapOR62	TMHMM2.0	TMhelix	   128   150
PrapOR62	TMHMM2.0	inside	   151   180
PrapOR62	TMHMM2.0	TMhelix	   181   203
PrapOR62	TMHMM2.0	outside	   204   265
PrapOR62	TMHMM2.0	TMhelix	   266   288
PrapOR62	TMHMM2.0	inside	   289   300
PrapOR62	TMHMM2.0	TMhelix	   301   323
PrapOR62	TMHMM2.0	outside	   324   368
PrapOR62	TMHMM2.0	TMhelix	   369   391
PrapOR62	TMHMM2.0	inside	   392   396
```

# plot in postscript,
script for making the plot in gnuplot,
data for plot

---

```
# PrapOR6 Length: 394
# PrapOR6 Number of predicted TMHs:  6
# PrapOR6 Exp number of AAs in TMHs: 141.34803
# PrapOR6 Exp number, first 60 AAs:  21.5545
# PrapOR6 Total prob of N-in:        0.79541
# PrapOR6 POSSIBLE N-term signal sequence
PrapOR6	TMHMM2.0	inside	     1    39
PrapOR6	TMHMM2.0	TMhelix	    40    62
PrapOR6	TMHMM2.0	outside	    63    74
PrapOR6	TMHMM2.0	TMhelix	    75    92
PrapOR6	TMHMM2.0	inside	    93   131
PrapOR6	TMHMM2.0	TMhelix	   132   151
PrapOR6	TMHMM2.0	outside	   152   197
PrapOR6	TMHMM2.0	TMhelix	   198   220
PrapOR6	TMHMM2.0	inside	   221   270
PrapOR6	TMHMM2.0	TMhelix	   271   293
PrapOR6	TMHMM2.0	outside	   294   302
PrapOR6	TMHMM2.0	TMhelix	   303   322
PrapOR6	TMHMM2.0	inside	   323   394
```

# plot in postscript,
script for making the plot in gnuplot,
data for plot

---

```
# PrapOR60 Length: 392
# PrapOR60 Number of predicted TMHs:  4
# PrapOR60 Exp number of AAs in TMHs: 123.1538
# PrapOR60 Exp number, first 60 AAs:  16.65783
# PrapOR60 Total prob of N-in:        0.41143
# PrapOR60 POSSIBLE N-term signal sequence
PrapOR60	TMHMM2.0	inside	     1   130
PrapOR60	TMHMM2.0	TMhelix	   131   153
PrapOR60	TMHMM2.0	outside	   154   188
PrapOR60	TMHMM2.0	TMhelix	   189   211
PrapOR60	TMHMM2.0	inside	   212   271
PrapOR60	TMHMM2.0	TMhelix	   272   291
PrapOR60	TMHMM2.0	outside	   292   300
PrapOR60	TMHMM2.0	TMhelix	   301   320
PrapOR60	TMHMM2.0	inside	   321   392
```

# plot in postscript,
script for making the plot in gnuplot,
data for plot

---

```
# PrapOR48 Length: 393
# PrapOR48 Number of predicted TMHs:  6
# PrapOR48 Exp number of AAs in TMHs: 148.33217
# PrapOR48 Exp number, first 60 AAs:  22.75905
# PrapOR48 Total prob of N-in:        0.95098
# PrapOR48 POSSIBLE N-term signal sequence
PrapOR48	TMHMM2.0	inside	     1    36
PrapOR48	TMHMM2.0	TMhelix	    37    59
PrapOR48	TMHMM2.0	outside	    60    68
PrapOR48	TMHMM2.0	TMhelix	    69    91
PrapOR48	TMHMM2.0	inside	    92   127
PrapOR48	TMHMM2.0	TMhelix	   128   150
PrapOR48	TMHMM2.0	outside	   151   179
PrapOR48	TMHMM2.0	TMhelix	   180   202
PrapOR48	TMHMM2.0	inside	   203   263
PrapOR48	TMHMM2.0	TMhelix	   264   286
PrapOR48	TMHMM2.0	outside	   287   290
PrapOR48	TMHMM2.0	TMhelix	   291   313
PrapOR48	TMHMM2.0	inside	   314   393
```

# plot in postscript,
script for making the plot in gnuplot,
data for plot

---

```
# PrapOR49 Length: 394
# PrapOR49 Number of predicted TMHs:  4
# PrapOR49 Exp number of AAs in TMHs: 104.07682
# PrapOR49 Exp number, first 60 AAs:  20.06458
# PrapOR49 Total prob of N-in:        0.93438
# PrapOR49 POSSIBLE N-term signal sequence
PrapOR49	TMHMM2.0	inside	     1    42
PrapOR49	TMHMM2.0	TMhelix	    43    65
PrapOR49	TMHMM2.0	outside	    66    69
PrapOR49	TMHMM2.0	TMhelix	    70    92
PrapOR49	TMHMM2.0	inside	    93   126
PrapOR49	TMHMM2.0	TMhelix	   127   149
PrapOR49	TMHMM2.0	outside	   150   268
PrapOR49	TMHMM2.0	TMhelix	   269   288
PrapOR49	TMHMM2.0	inside	   289   394
```

# plot in postscript,
script for making the plot in gnuplot,
data for plot

---

```
# PrapOR50 Length: 394
# PrapOR50 Number of predicted TMHs:  4
# PrapOR50 Exp number of AAs in TMHs: 109.60279
# PrapOR50 Exp number, first 60 AAs:  40.3643
# PrapOR50 Total prob of N-in:        0.16334
# PrapOR50 POSSIBLE N-term signal sequence
PrapOR50	TMHMM2.0	outside	     1     9
PrapOR50	TMHMM2.0	TMhelix	    10    32
PrapOR50	TMHMM2.0	inside	    33    36
PrapOR50	TMHMM2.0	TMhelix	    37    59
PrapOR50	TMHMM2.0	outside	    60    73
PrapOR50	TMHMM2.0	TMhelix	    74    93
PrapOR50	TMHMM2.0	inside	    94   129
PrapOR50	TMHMM2.0	TMhelix	   130   152
PrapOR50	TMHMM2.0	outside	   153   394
```

# plot in postscript,
script for making the plot in gnuplot,
data for plot

---

```
# PrapOR51 Length: 389
# PrapOR51 Number of predicted TMHs:  6
# PrapOR51 Exp number of AAs in TMHs: 133.12593
# PrapOR51 Exp number, first 60 AAs:  21.3184
# PrapOR51 Total prob of N-in:        0.99576
# PrapOR51 POSSIBLE N-term signal sequence
PrapOR51	TMHMM2.0	inside	     1    36
PrapOR51	TMHMM2.0	TMhelix	    37    59
PrapOR51	TMHMM2.0	outside	    60    68
PrapOR51	TMHMM2.0	TMhelix	    69    88
PrapOR51	TMHMM2.0	inside	    89   123
PrapOR51	TMHMM2.0	TMhelix	   124   146
PrapOR51	TMHMM2.0	outside	   147   180
PrapOR51	TMHMM2.0	TMhelix	   181   203
PrapOR51	TMHMM2.0	inside	   204   264
PrapOR51	TMHMM2.0	TMhelix	   265   287
PrapOR51	TMHMM2.0	outside	   288   291
PrapOR51	TMHMM2.0	TMhelix	   292   314
PrapOR51	TMHMM2.0	inside	   315   389
```

# plot in postscript,
script for making the plot in gnuplot,
data for plot

---

```
# PrapOR52 Length: 391
# PrapOR52 Number of predicted TMHs:  6
# PrapOR52 Exp number of AAs in TMHs: 122.80953
# PrapOR52 Exp number, first 60 AAs:  22.90781
# PrapOR52 Total prob of N-in:        0.71637
# PrapOR52 POSSIBLE N-term signal sequence
PrapOR52	TMHMM2.0	inside	     1    36
PrapOR52	TMHMM2.0	TMhelix	    37    59
PrapOR52	TMHMM2.0	outside	    60    68
PrapOR52	TMHMM2.0	TMhelix	    69    86
PrapOR52	TMHMM2.0	inside	    87   122
PrapOR52	TMHMM2.0	TMhelix	   123   145
PrapOR52	TMHMM2.0	outside	   146   170
PrapOR52	TMHMM2.0	TMhelix	   171   190
PrapOR52	TMHMM2.0	inside	   191   262
PrapOR52	TMHMM2.0	TMhelix	   263   285
PrapOR52	TMHMM2.0	outside	   286   289
PrapOR52	TMHMM2.0	TMhelix	   290   312
PrapOR52	TMHMM2.0	inside	   313   391
```

# plot in postscript,
script for making the plot in gnuplot,
data for plot

---

```
# PrapOR44 Length: 389
# PrapOR44 Number of predicted TMHs:  8
# PrapOR44 Exp number of AAs in TMHs: 165.20087
# PrapOR44 Exp number, first 60 AAs:  22.31617
# PrapOR44 Total prob of N-in:        0.99296
# PrapOR44 POSSIBLE N-term signal sequence
PrapOR44	TMHMM2.0	inside	     1    29
PrapOR44	TMHMM2.0	TMhelix	    30    52
PrapOR44	TMHMM2.0	outside	    53    61
PrapOR44	TMHMM2.0	TMhelix	    62    84
PrapOR44	TMHMM2.0	inside	    85   125
PrapOR44	TMHMM2.0	TMhelix	   126   148
PrapOR44	TMHMM2.0	outside	   149   167
PrapOR44	TMHMM2.0	TMhelix	   168   187
PrapOR44	TMHMM2.0	inside	   188   193
PrapOR44	TMHMM2.0	TMhelix	   194   212
PrapOR44	TMHMM2.0	outside	   213   264
PrapOR44	TMHMM2.0	TMhelix	   265   287
PrapOR44	TMHMM2.0	inside	   288   293
PrapOR44	TMHMM2.0	TMhelix	   294   316
PrapOR44	TMHMM2.0	outside	   317   362
PrapOR44	TMHMM2.0	TMhelix	   363   385
PrapOR44	TMHMM2.0	inside	   386   389
```

# plot in postscript,
script for making the plot in gnuplot,
data for plot

---

```
# PrapOR45a Length: 386
# PrapOR45a Number of predicted TMHs:  7
# PrapOR45a Exp number of AAs in TMHs: 163.97868
# PrapOR45a Exp number, first 60 AAs:  40.31441
# PrapOR45a Total prob of N-in:        0.93372
# PrapOR45a POSSIBLE N-term signal sequence
PrapOR45a	TMHMM2.0	inside	     1     6
PrapOR45a	TMHMM2.0	TMhelix	     7    24
PrapOR45a	TMHMM2.0	outside	    25    33
PrapOR45a	TMHMM2.0	TMhelix	    34    56
PrapOR45a	TMHMM2.0	inside	    57   126
PrapOR45a	TMHMM2.0	TMhelix	   127   149
PrapOR45a	TMHMM2.0	outside	   150   181
PrapOR45a	TMHMM2.0	TMhelix	   182   204
PrapOR45a	TMHMM2.0	inside	   205   253
PrapOR45a	TMHMM2.0	TMhelix	   254   276
PrapOR45a	TMHMM2.0	outside	   277   285
PrapOR45a	TMHMM2.0	TMhelix	   286   308
PrapOR45a	TMHMM2.0	inside	   309   339
PrapOR45a	TMHMM2.0	TMhelix	   340   362
PrapOR45a	TMHMM2.0	outside	   363   386
```

# plot in postscript,
script for making the plot in gnuplot,
data for plot

---

```
# PrapOR45b Length: 385
# PrapOR45b Number of predicted TMHs:  7
# PrapOR45b Exp number of AAs in TMHs: 157.82388
# PrapOR45b Exp number, first 60 AAs:  27.24821
# PrapOR45b Total prob of N-in:        0.86629
# PrapOR45b POSSIBLE N-term signal sequence
PrapOR45b	TMHMM2.0	inside	     1     6
PrapOR45b	TMHMM2.0	TMhelix	     7    24
PrapOR45b	TMHMM2.0	outside	    25    33
PrapOR45b	TMHMM2.0	TMhelix	    34    56
PrapOR45b	TMHMM2.0	inside	    57   127
PrapOR45b	TMHMM2.0	TMhelix	   128   150
PrapOR45b	TMHMM2.0	outside	   151   182
PrapOR45b	TMHMM2.0	TMhelix	   183   205
PrapOR45b	TMHMM2.0	inside	   206   252
PrapOR45b	TMHMM2.0	TMhelix	   253   275
PrapOR45b	TMHMM2.0	outside	   276   284
PrapOR45b	TMHMM2.0	TMhelix	   285   307
PrapOR45b	TMHMM2.0	inside	   308   343
PrapOR45b	TMHMM2.0	TMhelix	   344   366
PrapOR45b	TMHMM2.0	outside	   367   385
```

# plot in postscript,
script for making the plot in gnuplot,
data for plot

---

```
# PrapOR53 Length: 398
# PrapOR53 Number of predicted TMHs:  4
# PrapOR53 Exp number of AAs in TMHs: 97.34008
# PrapOR53 Exp number, first 60 AAs:  4.21433
# PrapOR53 Total prob of N-in:        0.52211
PrapOR53	TMHMM2.0	inside	     1   132
PrapOR53	TMHMM2.0	TMhelix	   133   155
PrapOR53	TMHMM2.0	outside	   156   188
PrapOR53	TMHMM2.0	TMhelix	   189   211
PrapOR53	TMHMM2.0	inside	   212   269
PrapOR53	TMHMM2.0	TMhelix	   270   291
PrapOR53	TMHMM2.0	outside	   292   300
PrapOR53	TMHMM2.0	TMhelix	   301   323
PrapOR53	TMHMM2.0	inside	   324   398
```

# plot in postscript,
script for making the plot in gnuplot,
data for plot

---

```
# PrapOR46 Length: 392
# PrapOR46 Number of predicted TMHs:  5
# PrapOR46 Exp number of AAs in TMHs: 120.42366
# PrapOR46 Exp number, first 60 AAs:  19.86727
# PrapOR46 Total prob of N-in:        0.61744
# PrapOR46 POSSIBLE N-term signal sequence
PrapOR46	TMHMM2.0	outside	     1    35
PrapOR46	TMHMM2.0	TMhelix	    36    53
PrapOR46	TMHMM2.0	inside	    54   129
PrapOR46	TMHMM2.0	TMhelix	   130   152
PrapOR46	TMHMM2.0	outside	   153   171
PrapOR46	TMHMM2.0	TMhelix	   172   191
PrapOR46	TMHMM2.0	inside	   192   263
PrapOR46	TMHMM2.0	TMhelix	   264   286
PrapOR46	TMHMM2.0	outside	   287   295
PrapOR46	TMHMM2.0	TMhelix	   296   318
PrapOR46	TMHMM2.0	inside	   319   392
```

# plot in postscript,
script for making the plot in gnuplot,
data for plot

---

```
# PrapOR43 Length: 427
# PrapOR43 Number of predicted TMHs:  6
# PrapOR43 Exp number of AAs in TMHs: 135.90391
# PrapOR43 Exp number, first 60 AAs:  2.72322
# PrapOR43 Total prob of N-in:        0.99165
PrapOR43	TMHMM2.0	inside	     1    58
PrapOR43	TMHMM2.0	TMhelix	    59    81
PrapOR43	TMHMM2.0	outside	    82    95
PrapOR43	TMHMM2.0	TMhelix	    96   118
PrapOR43	TMHMM2.0	inside	   119   154
PrapOR43	TMHMM2.0	TMhelix	   155   177
PrapOR43	TMHMM2.0	outside	   178   223
PrapOR43	TMHMM2.0	TMhelix	   224   246
PrapOR43	TMHMM2.0	inside	   247   296
PrapOR43	TMHMM2.0	TMhelix	   297   319
PrapOR43	TMHMM2.0	outside	   320   328
PrapOR43	TMHMM2.0	TMhelix	   329   351
PrapOR43	TMHMM2.0	inside	   352   427
```

# plot in postscript,
script for making the plot in gnuplot,
data for plot

---

```
# PrapOR28 Length: 410
# PrapOR28 Number of predicted TMHs:  5
# PrapOR28 Exp number of AAs in TMHs: 121.66827
# PrapOR28 Exp number, first 60 AAs:  9.12696
# PrapOR28 Total prob of N-in:        0.88737
PrapOR28	TMHMM2.0	inside	     1    48
PrapOR28	TMHMM2.0	TMhelix	    49    71
PrapOR28	TMHMM2.0	outside	    72    85
PrapOR28	TMHMM2.0	TMhelix	    86   108
PrapOR28	TMHMM2.0	inside	   109   151
PrapOR28	TMHMM2.0	TMhelix	   152   174
PrapOR28	TMHMM2.0	outside	   175   205
PrapOR28	TMHMM2.0	TMhelix	   206   228
PrapOR28	TMHMM2.0	inside	   229   289
PrapOR28	TMHMM2.0	TMhelix	   290   312
PrapOR28	TMHMM2.0	outside	   313   410
```

# plot in postscript,
script for making the plot in gnuplot,
data for plot

---

```
# PrapOR2 Length: 387
# PrapOR2 Number of predicted TMHs:  6
# PrapOR2 Exp number of AAs in TMHs: 144.39759
# PrapOR2 Exp number, first 60 AAs:  22.40753
# PrapOR2 Total prob of N-in:        0.99546
# PrapOR2 POSSIBLE N-term signal sequence
PrapOR2	TMHMM2.0	inside	     1    37
PrapOR2	TMHMM2.0	TMhelix	    38    60
PrapOR2	TMHMM2.0	outside	    61    69
PrapOR2	TMHMM2.0	TMhelix	    70    89
PrapOR2	TMHMM2.0	inside	    90   132
PrapOR2	TMHMM2.0	TMhelix	   133   155
PrapOR2	TMHMM2.0	outside	   156   193
PrapOR2	TMHMM2.0	TMhelix	   194   216
PrapOR2	TMHMM2.0	inside	   217   260
PrapOR2	TMHMM2.0	TMhelix	   261   283
PrapOR2	TMHMM2.0	outside	   284   292
PrapOR2	TMHMM2.0	TMhelix	   293   310
PrapOR2	TMHMM2.0	inside	   311   387
```

# plot in postscript,
script for making the plot in gnuplot,
data for plot

---

```
# PrapOR23 Length: 387
# PrapOR23 Number of predicted TMHs:  4
# PrapOR23 Exp number of AAs in TMHs: 96.0685000000001
# PrapOR23 Exp number, first 60 AAs:  19.44059
# PrapOR23 Total prob of N-in:        0.97511
# PrapOR23 POSSIBLE N-term signal sequence
PrapOR23	TMHMM2.0	inside	     1    40
PrapOR23	TMHMM2.0	TMhelix	    41    58
PrapOR23	TMHMM2.0	outside	    59    72
PrapOR23	TMHMM2.0	TMhelix	    73    90
PrapOR23	TMHMM2.0	inside	    91   124
PrapOR23	TMHMM2.0	TMhelix	   125   142
PrapOR23	TMHMM2.0	outside	   143   177
PrapOR23	TMHMM2.0	TMhelix	   178   200
PrapOR23	TMHMM2.0	inside	   201   387
```

# plot in postscript,
script for making the plot in gnuplot,
data for plot

---

```
# PrapOR39 Length: 390
# PrapOR39 Number of predicted TMHs:  6
# PrapOR39 Exp number of AAs in TMHs: 144.30416
# PrapOR39 Exp number, first 60 AAs:  26.14662
# PrapOR39 Total prob of N-in:        0.42792
# PrapOR39 POSSIBLE N-term signal sequence
PrapOR39	TMHMM2.0	inside	     1    43
PrapOR39	TMHMM2.0	TMhelix	    44    66
PrapOR39	TMHMM2.0	outside	    67    75
PrapOR39	TMHMM2.0	TMhelix	    76    98
PrapOR39	TMHMM2.0	inside	    99   145
PrapOR39	TMHMM2.0	TMhelix	   146   168
PrapOR39	TMHMM2.0	outside	   169   206
PrapOR39	TMHMM2.0	TMhelix	   207   229
PrapOR39	TMHMM2.0	inside	   230   279
PrapOR39	TMHMM2.0	TMhelix	   280   302
PrapOR39	TMHMM2.0	outside	   303   306
PrapOR39	TMHMM2.0	TMhelix	   307   329
PrapOR39	TMHMM2.0	inside	   330   390
```

# plot in postscript,
script for making the plot in gnuplot,
data for plot

---

```
# PrapOR4 Length: 396
# PrapOR4 Number of predicted TMHs:  6
# PrapOR4 Exp number of AAs in TMHs: 129.30797
# PrapOR4 Exp number, first 60 AAs:  19.55942
# PrapOR4 Total prob of N-in:        0.87848
# PrapOR4 POSSIBLE N-term signal sequence
PrapOR4	TMHMM2.0	inside	     1    41
PrapOR4	TMHMM2.0	TMhelix	    42    64
PrapOR4	TMHMM2.0	outside	    65    83
PrapOR4	TMHMM2.0	TMhelix	    84   106
PrapOR4	TMHMM2.0	inside	   107   141
PrapOR4	TMHMM2.0	TMhelix	   142   164
PrapOR4	TMHMM2.0	outside	   165   198
PrapOR4	TMHMM2.0	TMhelix	   199   221
PrapOR4	TMHMM2.0	inside	   222   272
PrapOR4	TMHMM2.0	TMhelix	   273   295
PrapOR4	TMHMM2.0	outside	   296   304
PrapOR4	TMHMM2.0	TMhelix	   305   324
PrapOR4	TMHMM2.0	inside	   325   396
```

# plot in postscript,
script for making the plot in gnuplot,
data for plot

---

```
# PrapOR5 Length: 407
# PrapOR5 Number of predicted TMHs:  7
# PrapOR5 Exp number of AAs in TMHs: 152.65154
# PrapOR5 Exp number, first 60 AAs:  21.68195
# PrapOR5 Total prob of N-in:        0.99574
# PrapOR5 POSSIBLE N-term signal sequence
PrapOR5	TMHMM2.0	inside	     1    33
PrapOR5	TMHMM2.0	TMhelix	    34    53
PrapOR5	TMHMM2.0	outside	    54    62
PrapOR5	TMHMM2.0	TMhelix	    63    85
PrapOR5	TMHMM2.0	inside	    86   141
PrapOR5	TMHMM2.0	TMhelix	   142   161
PrapOR5	TMHMM2.0	outside	   162   198
PrapOR5	TMHMM2.0	TMhelix	   199   221
PrapOR5	TMHMM2.0	inside	   222   280
PrapOR5	TMHMM2.0	TMhelix	   281   303
PrapOR5	TMHMM2.0	outside	   304   312
PrapOR5	TMHMM2.0	TMhelix	   313   332
PrapOR5	TMHMM2.0	inside	   333   361
PrapOR5	TMHMM2.0	TMhelix	   362   384
PrapOR5	TMHMM2.0	outside	   385   407
```

# plot in postscript,
script for making the plot in gnuplot,
data for plot

---

```
# PbraIR100a Length: 671
# PbraIR100a Number of predicted TMHs:  6
# PbraIR100a Exp number of AAs in TMHs: 127.43216
# PbraIR100a Exp number, first 60 AAs:  22.96516
# PbraIR100a Total prob of N-in:        0.00815
# PbraIR100a POSSIBLE N-term signal sequence
PbraIR100a	TMHMM2.0	outside	     1    14
PbraIR100a	TMHMM2.0	TMhelix	    15    37
PbraIR100a	TMHMM2.0	inside	    38   121
PbraIR100a	TMHMM2.0	TMhelix	   122   144
PbraIR100a	TMHMM2.0	outside	   145   148
PbraIR100a	TMHMM2.0	TMhelix	   149   171
PbraIR100a	TMHMM2.0	inside	   172   396
PbraIR100a	TMHMM2.0	TMhelix	   397   416
PbraIR100a	TMHMM2.0	outside	   417   457
PbraIR100a	TMHMM2.0	TMhelix	   458   480
PbraIR100a	TMHMM2.0	inside	   481   634
PbraIR100a	TMHMM2.0	TMhelix	   635   657
PbraIR100a	TMHMM2.0	outside	   658   671
```

# plot in postscript,
script for making the plot in gnuplot,
data for plot

---

```
# PbraIR21a Length: 802
# PbraIR21a Number of predicted TMHs:  4
# PbraIR21a Exp number of AAs in TMHs: 87.4416
# PbraIR21a Exp number, first 60 AAs:  0.12827
# PbraIR21a Total prob of N-in:        0.43038
PbraIR21a	TMHMM2.0	inside	     1   395
PbraIR21a	TMHMM2.0	TMhelix	   396   415
PbraIR21a	TMHMM2.0	outside	   416   434
PbraIR21a	TMHMM2.0	TMhelix	   435   454
PbraIR21a	TMHMM2.0	inside	   455   465
PbraIR21a	TMHMM2.0	TMhelix	   466   488
PbraIR21a	TMHMM2.0	outside	   489   669
PbraIR21a	TMHMM2.0	TMhelix	   670   692
PbraIR21a	TMHMM2.0	inside	   693   802
```

# plot in postscript,
script for making the plot in gnuplot,
data for plot

---

```
# PbraIR68a Length: 695
# PbraIR68a Number of predicted TMHs:  4
# PbraIR68a Exp number of AAs in TMHs: 93.4751800000002
# PbraIR68a Exp number, first 60 AAs:  3.71544
# PbraIR68a Total prob of N-in:        0.17223
PbraIR68a	TMHMM2.0	outside	     1   357
PbraIR68a	TMHMM2.0	TMhelix	   358   380
PbraIR68a	TMHMM2.0	inside	   381   405
PbraIR68a	TMHMM2.0	TMhelix	   406   428
PbraIR68a	TMHMM2.0	outside	   429   437
PbraIR68a	TMHMM2.0	TMhelix	   438   457
PbraIR68a	TMHMM2.0	inside	   458   645
PbraIR68a	TMHMM2.0	TMhelix	   646   668
PbraIR68a	TMHMM2.0	outside	   669   695
```

# plot in postscript,
script for making the plot in gnuplot,
data for plot

---

```
# PbraIR40a Length: 668
# PbraIR40a Number of predicted TMHs:  3
# PbraIR40a Exp number of AAs in TMHs: 67.9737800000001
# PbraIR40a Exp number, first 60 AAs:  0.0027
# PbraIR40a Total prob of N-in:        0.00551
PbraIR40a	TMHMM2.0	outside	     1   311
PbraIR40a	TMHMM2.0	TMhelix	   312   334
PbraIR40a	TMHMM2.0	inside	   335   377
PbraIR40a	TMHMM2.0	TMhelix	   378   397
PbraIR40a	TMHMM2.0	outside	   398   594
PbraIR40a	TMHMM2.0	TMhelix	   595   617
PbraIR40a	TMHMM2.0	inside	   618   668
```

# plot in postscript,
script for making the plot in gnuplot,
data for plot

---

```
# PbraIR41a Length: 593
# PbraIR41a Number of predicted TMHs:  3
# PbraIR41a Exp number of AAs in TMHs: 63.5918500000001
# PbraIR41a Exp number, first 60 AAs:  0.00057
# PbraIR41a Total prob of N-in:        0.00072
PbraIR41a	TMHMM2.0	outside	     1   309
PbraIR41a	TMHMM2.0	TMhelix	   310   332
PbraIR41a	TMHMM2.0	inside	   333   365
PbraIR41a	TMHMM2.0	TMhelix	   366   388
PbraIR41a	TMHMM2.0	outside	   389   560
PbraIR41a	TMHMM2.0	TMhelix	   561   583
PbraIR41a	TMHMM2.0	inside	   584   593
```

# plot in postscript,
script for making the plot in gnuplot,
data for plot

---

```
# PbraIR76b Length: 532
# PbraIR76b Number of predicted TMHs:  4
# PbraIR76b Exp number of AAs in TMHs: 78.1322300000001
# PbraIR76b Exp number, first 60 AAs:  0.00546
# PbraIR76b Total prob of N-in:        0.02353
PbraIR76b	TMHMM2.0	outside	     1   166
PbraIR76b	TMHMM2.0	TMhelix	   167   186
PbraIR76b	TMHMM2.0	inside	   187   198
PbraIR76b	TMHMM2.0	TMhelix	   199   221
PbraIR76b	TMHMM2.0	outside	   222   230
PbraIR76b	TMHMM2.0	TMhelix	   231   253
PbraIR76b	TMHMM2.0	inside	   254   426
PbraIR76b	TMHMM2.0	TMhelix	   427   449
PbraIR76b	TMHMM2.0	outside	   450   532
```

# plot in postscript,
script for making the plot in gnuplot,
data for plot

---

```
# PbraIR93a Length: 867
# PbraIR93a Number of predicted TMHs:  3
# PbraIR93a Exp number of AAs in TMHs: 75.2237100000001
# PbraIR93a Exp number, first 60 AAs:  0.01249
# PbraIR93a Total prob of N-in:        0.00301
PbraIR93a	TMHMM2.0	outside	     1   554
PbraIR93a	TMHMM2.0	TMhelix	   555   577
PbraIR93a	TMHMM2.0	inside	   578   624
PbraIR93a	TMHMM2.0	TMhelix	   625   647
PbraIR93a	TMHMM2.0	outside	   648   828
PbraIR93a	TMHMM2.0	TMhelix	   829   851
PbraIR93a	TMHMM2.0	inside	   852   867
```

# plot in postscript,
script for making the plot in gnuplot,
data for plot

---

```
# PbraIR8a Length: 852
# PbraIR8a Number of predicted TMHs:  3
# PbraIR8a Exp number of AAs in TMHs: 81.6823500000001
# PbraIR8a Exp number, first 60 AAs:  16.10649
# PbraIR8a Total prob of N-in:        0.76578
# PbraIR8a POSSIBLE N-term signal sequence
PbraIR8a	TMHMM2.0	outside	     1   490
PbraIR8a	TMHMM2.0	TMhelix	   491   509
PbraIR8a	TMHMM2.0	inside	   510   563
PbraIR8a	TMHMM2.0	TMhelix	   564   586
PbraIR8a	TMHMM2.0	outside	   587   778
PbraIR8a	TMHMM2.0	TMhelix	   779   801
PbraIR8a	TMHMM2.0	inside	   802   852
```

# plot in postscript,
script for making the plot in gnuplot,
data for plot

---

```
# PbraIR25a1 Length: 922
# PbraIR25a1 Number of predicted TMHs:  3
# PbraIR25a1 Exp number of AAs in TMHs: 65.09327
# PbraIR25a1 Exp number, first 60 AAs:  0.09033
# PbraIR25a1 Total prob of N-in:        0.00597
PbraIR25a1	TMHMM2.0	outside	     1   550
PbraIR25a1	TMHMM2.0	TMhelix	   551   569
PbraIR25a1	TMHMM2.0	inside	   570   625
PbraIR25a1	TMHMM2.0	TMhelix	   626   648
PbraIR25a1	TMHMM2.0	outside	   649   848
PbraIR25a1	TMHMM2.0	TMhelix	   849   871
PbraIR25a1	TMHMM2.0	inside	   872   922
```

# plot in postscript,
script for making the plot in gnuplot,
data for plot

---

```
# PbraIR25a2 Length: 909
# PbraIR25a2 Number of predicted TMHs:  3
# PbraIR25a2 Exp number of AAs in TMHs: 65.33761
# PbraIR25a2 Exp number, first 60 AAs:  0.00579999999999999
# PbraIR25a2 Total prob of N-in:        0.00328
PbraIR25a2	TMHMM2.0	outside	     1   549
PbraIR25a2	TMHMM2.0	TMhelix	   550   568
PbraIR25a2	TMHMM2.0	inside	   569   621
PbraIR25a2	TMHMM2.0	TMhelix	   622   644
PbraIR25a2	TMHMM2.0	outside	   645   845
PbraIR25a2	TMHMM2.0	TMhelix	   846   868
PbraIR25a2	TMHMM2.0	inside	   869   909
```

# plot in postscript,
script for making the plot in gnuplot,
data for plot

---

```
# PbraIR85a Length: 633
# PbraIR85a Number of predicted TMHs:  2
# PbraIR85a Exp number of AAs in TMHs: 61.81534
# PbraIR85a Exp number, first 60 AAs:  0.16809
# PbraIR85a Total prob of N-in:        0.16768
PbraIR85a	TMHMM2.0	outside	     1   335
PbraIR85a	TMHMM2.0	TMhelix	   336   358
PbraIR85a	TMHMM2.0	inside	   359   593
PbraIR85a	TMHMM2.0	TMhelix	   594   616
PbraIR85a	TMHMM2.0	outside	   617   633
```

# plot in postscript,
script for making the plot in gnuplot,
data for plot

---

```
# PbraIR143 Length: 611
# PbraIR143 Number of predicted TMHs:  2
# PbraIR143 Exp number of AAs in TMHs: 58.89431
# PbraIR143 Exp number, first 60 AAs:  0.00015
# PbraIR143 Total prob of N-in:        0.03126
PbraIR143	TMHMM2.0	outside	     1   330
PbraIR143	TMHMM2.0	TMhelix	   331   348
PbraIR143	TMHMM2.0	inside	   349   580
PbraIR143	TMHMM2.0	TMhelix	   581   603
PbraIR143	TMHMM2.0	outside	   604   611
```

# plot in postscript,
script for making the plot in gnuplot,
data for plot

---

```
# PbraIR7d.3 Length: 595
# PbraIR7d.3 Number of predicted TMHs:  3
# PbraIR7d.3 Exp number of AAs in TMHs: 62.88179
# PbraIR7d.3 Exp number, first 60 AAs:  0.00051
# PbraIR7d.3 Total prob of N-in:        0.00418
PbraIR7d.3	TMHMM2.0	outside	     1   310
PbraIR7d.3	TMHMM2.0	TMhelix	   311   333
PbraIR7d.3	TMHMM2.0	inside	   334   361
PbraIR7d.3	TMHMM2.0	TMhelix	   362   384
PbraIR7d.3	TMHMM2.0	outside	   385   549
PbraIR7d.3	TMHMM2.0	TMhelix	   550   572
PbraIR7d.3	TMHMM2.0	inside	   573   595
```

# plot in postscript,
script for making the plot in gnuplot,
data for plot

---

```
# PbraIR7d.2 Length: 592
# PbraIR7d.2 Number of predicted TMHs:  4
# PbraIR7d.2 Exp number of AAs in TMHs: 84.39516
# PbraIR7d.2 Exp number, first 60 AAs:  18.97377
# PbraIR7d.2 Total prob of N-in:        0.05242
# PbraIR7d.2 POSSIBLE N-term signal sequence
PbraIR7d.2	TMHMM2.0	outside	     1    29
PbraIR7d.2	TMHMM2.0	TMhelix	    30    52
PbraIR7d.2	TMHMM2.0	inside	    53   315
PbraIR7d.2	TMHMM2.0	TMhelix	   316   334
PbraIR7d.2	TMHMM2.0	outside	   335   376
PbraIR7d.2	TMHMM2.0	TMhelix	   377   399
PbraIR7d.2	TMHMM2.0	inside	   400   552
PbraIR7d.2	TMHMM2.0	TMhelix	   553   572
PbraIR7d.2	TMHMM2.0	outside	   573   592
```

# plot in postscript,
script for making the plot in gnuplot,
data for plot

---

```
# PbraIR7d.2.1 Length: 586
# PbraIR7d.2.1 Number of predicted TMHs:  3
# PbraIR7d.2.1 Exp number of AAs in TMHs: 95.97218
# PbraIR7d.2.1 Exp number, first 60 AAs:  12.89797
# PbraIR7d.2.1 Total prob of N-in:        0.06905
# PbraIR7d.2.1 POSSIBLE N-term signal sequence
PbraIR7d.2.1	TMHMM2.0	outside	     1   310
PbraIR7d.2.1	TMHMM2.0	TMhelix	   311   333
PbraIR7d.2.1	TMHMM2.0	inside	   334   373
PbraIR7d.2.1	TMHMM2.0	TMhelix	   374   393
PbraIR7d.2.1	TMHMM2.0	outside	   394   548
PbraIR7d.2.1	TMHMM2.0	TMhelix	   549   571
PbraIR7d.2.1	TMHMM2.0	inside	   572   586
```

# plot in postscript,
script for making the plot in gnuplot,
data for plot

---

```
# PbraIR87a Length: 639
# PbraIR87a Number of predicted TMHs:  4
# PbraIR87a Exp number of AAs in TMHs: 86.3547000000001
# PbraIR87a Exp number, first 60 AAs:  3.92558
# PbraIR87a Total prob of N-in:        0.14957
PbraIR87a	TMHMM2.0	outside	     1   361
PbraIR87a	TMHMM2.0	TMhelix	   362   379
PbraIR87a	TMHMM2.0	inside	   380   391
PbraIR87a	TMHMM2.0	TMhelix	   392   411
PbraIR87a	TMHMM2.0	outside	   412   420
PbraIR87a	TMHMM2.0	TMhelix	   421   443
PbraIR87a	TMHMM2.0	inside	   444   599
PbraIR87a	TMHMM2.0	TMhelix	   600   622
PbraIR87a	TMHMM2.0	outside	   623   639
```

# plot in postscript,
script for making the plot in gnuplot,
data for plot

---

```
# PbraIR100c Length: 580
# PbraIR100c Number of predicted TMHs:  3
# PbraIR100c Exp number of AAs in TMHs: 74.85637
# PbraIR100c Exp number, first 60 AAs:  0.38521
# PbraIR100c Total prob of N-in:        0.33126
PbraIR100c	TMHMM2.0	outside	     1   319
PbraIR100c	TMHMM2.0	TMhelix	   320   339
PbraIR100c	TMHMM2.0	inside	   340   370
PbraIR100c	TMHMM2.0	TMhelix	   371   393
PbraIR100c	TMHMM2.0	outside	   394   548
PbraIR100c	TMHMM2.0	TMhelix	   549   571
PbraIR100c	TMHMM2.0	inside	   572   580
```

# plot in postscript,
script for making the plot in gnuplot,
data for plot

---

```
# PbraIR100d Length: 578
# PbraIR100d Number of predicted TMHs:  3
# PbraIR100d Exp number of AAs in TMHs: 86.65597
# PbraIR100d Exp number, first 60 AAs:  1.52892
# PbraIR100d Total prob of N-in:        0.14819
PbraIR100d	TMHMM2.0	outside	     1   307
PbraIR100d	TMHMM2.0	TMhelix	   308   330
PbraIR100d	TMHMM2.0	inside	   331   359
PbraIR100d	TMHMM2.0	TMhelix	   360   382
PbraIR100d	TMHMM2.0	outside	   383   544
PbraIR100d	TMHMM2.0	TMhelix	   545   567
PbraIR100d	TMHMM2.0	inside	   568   578
```

# plot in postscript,
script for making the plot in gnuplot,
data for plot

---

```
# PbraIR100f Length: 614
# PbraIR100f Number of predicted TMHs:  3
# PbraIR100f Exp number of AAs in TMHs: 68.5665300000001
# PbraIR100f Exp number, first 60 AAs:  2.40587
# PbraIR100f Total prob of N-in:        0.12017
PbraIR100f	TMHMM2.0	outside	     1   352
PbraIR100f	TMHMM2.0	TMhelix	   353   375
PbraIR100f	TMHMM2.0	inside	   376   406
PbraIR100f	TMHMM2.0	TMhelix	   407   429
PbraIR100f	TMHMM2.0	outside	   430   586
PbraIR100f	TMHMM2.0	TMhelix	   587   609
PbraIR100f	TMHMM2.0	inside	   610   614
```

# plot in postscript,
script for making the plot in gnuplot,
data for plot

---

```
# PbraIR31a Length: 585
# PbraIR31a Number of predicted TMHs:  3
# PbraIR31a Exp number of AAs in TMHs: 76.9290100000001
# PbraIR31a Exp number, first 60 AAs:  9.0982
# PbraIR31a Total prob of N-in:        0.39660
PbraIR31a	TMHMM2.0	outside	     1   296
PbraIR31a	TMHMM2.0	TMhelix	   297   319
PbraIR31a	TMHMM2.0	inside	   320   359
PbraIR31a	TMHMM2.0	TMhelix	   360   382
PbraIR31a	TMHMM2.0	outside	   383   545
PbraIR31a	TMHMM2.0	TMhelix	   546   568
PbraIR31a	TMHMM2.0	inside	   569   585
```

# plot in postscript,
script for making the plot in gnuplot,
data for plot

---

```
# PbraIR4 Length: 664
# PbraIR4 Number of predicted TMHs:  3
# PbraIR4 Exp number of AAs in TMHs: 66.9996000000001
# PbraIR4 Exp number, first 60 AAs:  0.02079
# PbraIR4 Total prob of N-in:        0.01313
PbraIR4	TMHMM2.0	outside	     1   369
PbraIR4	TMHMM2.0	TMhelix	   370   392
PbraIR4	TMHMM2.0	inside	   393   429
PbraIR4	TMHMM2.0	TMhelix	   430   452
PbraIR4	TMHMM2.0	outside	   453   620
PbraIR4	TMHMM2.0	TMhelix	   621   643
PbraIR4	TMHMM2.0	inside	   644   664
```

# plot in postscript,
script for making the plot in gnuplot,
data for plot

---

```
# PbraIR2 Length: 401
# PbraIR2 Number of predicted TMHs:  3
# PbraIR2 Exp number of AAs in TMHs: 73.07248
# PbraIR2 Exp number, first 60 AAs:  0.03641
# PbraIR2 Total prob of N-in:        0.68521
PbraIR2	TMHMM2.0	inside	     1   113
PbraIR2	TMHMM2.0	TMhelix	   114   133
PbraIR2	TMHMM2.0	outside	   134   181
PbraIR2	TMHMM2.0	TMhelix	   182   204
PbraIR2	TMHMM2.0	inside	   205   362
PbraIR2	TMHMM2.0	TMhelix	   363   385
PbraIR2	TMHMM2.0	outside	   386   401
```

# plot in postscript,
script for making the plot in gnuplot,
data for plot

---

```
# PbraIR64a Length: 609
# PbraIR64a Number of predicted TMHs:  3
# PbraIR64a Exp number of AAs in TMHs: 67.8895600000001
# PbraIR64a Exp number, first 60 AAs:  0.99951
# PbraIR64a Total prob of N-in:        0.05348
PbraIR64a	TMHMM2.0	outside	     1   309
PbraIR64a	TMHMM2.0	TMhelix	   310   332
PbraIR64a	TMHMM2.0	inside	   333   368
PbraIR64a	TMHMM2.0	TMhelix	   369   391
PbraIR64a	TMHMM2.0	outside	   392   566
PbraIR64a	TMHMM2.0	TMhelix	   567   589
PbraIR64a	TMHMM2.0	inside	   590   609
```

# plot in postscript,
script for making the plot in gnuplot,
data for plot

---

```
# PbraIR1.1 Length: 642
# PbraIR1.1 Number of predicted TMHs:  4
# PbraIR1.1 Exp number of AAs in TMHs: 89.8173900000001
# PbraIR1.1 Exp number, first 60 AAs:  20.58765
# PbraIR1.1 Total prob of N-in:        0.87607
# PbraIR1.1 POSSIBLE N-term signal sequence
PbraIR1.1	TMHMM2.0	inside	     1     2
PbraIR1.1	TMHMM2.0	TMhelix	     3    25
PbraIR1.1	TMHMM2.0	outside	    26   340
PbraIR1.1	TMHMM2.0	TMhelix	   341   363
PbraIR1.1	TMHMM2.0	inside	   364   405
PbraIR1.1	TMHMM2.0	TMhelix	   406   428
PbraIR1.1	TMHMM2.0	outside	   429   600
PbraIR1.1	TMHMM2.0	TMhelix	   601   623
PbraIR1.1	TMHMM2.0	inside	   624   642
```

# plot in postscript,
script for making the plot in gnuplot,
data for plot

---

```
# PbraIR1.2 Length: 645
# PbraIR1.2 Number of predicted TMHs:  3
# PbraIR1.2 Exp number of AAs in TMHs: 71.5019000000001
# PbraIR1.2 Exp number, first 60 AAs:  6.88666
# PbraIR1.2 Total prob of N-in:        0.33625
PbraIR1.2	TMHMM2.0	outside	     1   350
PbraIR1.2	TMHMM2.0	TMhelix	   351   370
PbraIR1.2	TMHMM2.0	inside	   371   413
PbraIR1.2	TMHMM2.0	TMhelix	   414   436
PbraIR1.2	TMHMM2.0	outside	   437   601
PbraIR1.2	TMHMM2.0	TMhelix	   602   624
PbraIR1.2	TMHMM2.0	inside	   625   645
```

# plot in postscript,
script for making the plot in gnuplot,
data for plot

---

```
# PbraIR75d Length: 600
# PbraIR75d Number of predicted TMHs:  3
# PbraIR75d Exp number of AAs in TMHs: 68.7670200000001
# PbraIR75d Exp number, first 60 AAs:  1.75406
# PbraIR75d Total prob of N-in:        0.04633
PbraIR75d	TMHMM2.0	outside	     1   315
PbraIR75d	TMHMM2.0	TMhelix	   316   338
PbraIR75d	TMHMM2.0	inside	   339   374
PbraIR75d	TMHMM2.0	TMhelix	   375   397
PbraIR75d	TMHMM2.0	outside	   398   571
PbraIR75d	TMHMM2.0	TMhelix	   572   594
PbraIR75d	TMHMM2.0	inside	   595   600
```

# plot in postscript,
script for making the plot in gnuplot,
data for plot

---

```
# PbraIR75q.1 Length: 608
# PbraIR75q.1 Number of predicted TMHs:  3
# PbraIR75q.1 Exp number of AAs in TMHs: 67.8585300000001
# PbraIR75q.1 Exp number, first 60 AAs:  0.14399
# PbraIR75q.1 Total prob of N-in:        0.01237
PbraIR75q.1	TMHMM2.0	outside	     1   315
PbraIR75q.1	TMHMM2.0	TMhelix	   316   338
PbraIR75q.1	TMHMM2.0	inside	   339   383
PbraIR75q.1	TMHMM2.0	TMhelix	   384   406
PbraIR75q.1	TMHMM2.0	outside	   407   573
PbraIR75q.1	TMHMM2.0	TMhelix	   574   596
PbraIR75q.1	TMHMM2.0	inside	   597   608
```

# plot in postscript,
script for making the plot in gnuplot,
data for plot

---

```
# PbraIR75q.2 Length: 619
# PbraIR75q.2 Number of predicted TMHs:  3
# PbraIR75q.2 Exp number of AAs in TMHs: 67.9203400000001
# PbraIR75q.2 Exp number, first 60 AAs:  0.01248
# PbraIR75q.2 Total prob of N-in:        0.00303
PbraIR75q.2	TMHMM2.0	outside	     1   321
PbraIR75q.2	TMHMM2.0	TMhelix	   322   344
PbraIR75q.2	TMHMM2.0	inside	   345   392
PbraIR75q.2	TMHMM2.0	TMhelix	   393   415
PbraIR75q.2	TMHMM2.0	outside	   416   582
PbraIR75q.2	TMHMM2.0	TMhelix	   583   605
PbraIR75q.2	TMHMM2.0	inside	   606   619
```

# plot in postscript,
script for making the plot in gnuplot,
data for plot

---

```
# PbraIR75p.2 Length: 607
# PbraIR75p.2 Number of predicted TMHs:  3
# PbraIR75p.2 Exp number of AAs in TMHs: 68.5412500000002
# PbraIR75p.2 Exp number, first 60 AAs:  0.02144
# PbraIR75p.2 Total prob of N-in:        0.03661
PbraIR75p.2	TMHMM2.0	outside	     1   322
PbraIR75p.2	TMHMM2.0	TMhelix	   323   345
PbraIR75p.2	TMHMM2.0	inside	   346   389
PbraIR75p.2	TMHMM2.0	TMhelix	   390   412
PbraIR75p.2	TMHMM2.0	outside	   413   578
PbraIR75p.2	TMHMM2.0	TMhelix	   579   601
PbraIR75p.2	TMHMM2.0	inside	   602   607
```

# plot in postscript,
script for making the plot in gnuplot,
data for plot

---

```
# PbraIR75p.1 Length: 638
# PbraIR75p.1 Number of predicted TMHs:  3
# PbraIR75p.1 Exp number of AAs in TMHs: 69.97485
# PbraIR75p.1 Exp number, first 60 AAs:  0.1473
# PbraIR75p.1 Total prob of N-in:        0.02415
PbraIR75p.1	TMHMM2.0	outside	     1   343
PbraIR75p.1	TMHMM2.0	TMhelix	   344   366
PbraIR75p.1	TMHMM2.0	inside	   367   411
PbraIR75p.1	TMHMM2.0	TMhelix	   412   434
PbraIR75p.1	TMHMM2.0	outside	   435   598
PbraIR75p.1	TMHMM2.0	TMhelix	   599   621
PbraIR75p.1	TMHMM2.0	inside	   622   638
```

# plot in postscript,
script for making the plot in gnuplot,
data for plot

---

```
# PbraIR7d.2.2 Length: 594
# PbraIR7d.2.2 Number of predicted TMHs:  5
# PbraIR7d.2.2 Exp number of AAs in TMHs: 120.81004
# PbraIR7d.2.2 Exp number, first 60 AAs:  21.86673
# PbraIR7d.2.2 Total prob of N-in:        0.01630
# PbraIR7d.2.2 POSSIBLE N-term signal sequence
PbraIR7d.2.2	TMHMM2.0	outside	     1    33
PbraIR7d.2.2	TMHMM2.0	TMhelix	    34    56
PbraIR7d.2.2	TMHMM2.0	inside	    57   286
PbraIR7d.2.2	TMHMM2.0	TMhelix	   287   301
PbraIR7d.2.2	TMHMM2.0	outside	   302   315
PbraIR7d.2.2	TMHMM2.0	TMhelix	   316   338
PbraIR7d.2.2	TMHMM2.0	inside	   339   382
PbraIR7d.2.2	TMHMM2.0	TMhelix	   383   405
PbraIR7d.2.2	TMHMM2.0	outside	   406   560
PbraIR7d.2.2	TMHMM2.0	TMhelix	   561   583
PbraIR7d.2.2	TMHMM2.0	inside	   584   594
```

# plot in postscript,
script for making the plot in gnuplot,
data for plot

---

```
# PrapIR100a Length: 671
# PrapIR100a Number of predicted TMHs:  4
# PrapIR100a Exp number of AAs in TMHs: 111.71077
# PrapIR100a Exp number, first 60 AAs:  20.64542
# PrapIR100a Total prob of N-in:        0.50295
# PrapIR100a POSSIBLE N-term signal sequence
PrapIR100a	TMHMM2.0	inside	     1    20
PrapIR100a	TMHMM2.0	TMhelix	    21    39
PrapIR100a	TMHMM2.0	outside	    40   396
PrapIR100a	TMHMM2.0	TMhelix	   397   416
PrapIR100a	TMHMM2.0	inside	   417   451
PrapIR100a	TMHMM2.0	TMhelix	   452   474
PrapIR100a	TMHMM2.0	outside	   475   634
PrapIR100a	TMHMM2.0	TMhelix	   635   657
PrapIR100a	TMHMM2.0	inside	   658   671
```

# plot in postscript,
script for making the plot in gnuplot,
data for plot

---

```
# PrapIR21a Length: 800
# PrapIR21a Number of predicted TMHs:  4
# PrapIR21a Exp number of AAs in TMHs: 85.82059
# PrapIR21a Exp number, first 60 AAs:  0.26694
# PrapIR21a Total prob of N-in:        0.49477
PrapIR21a	TMHMM2.0	inside	     1   392
PrapIR21a	TMHMM2.0	TMhelix	   393   412
PrapIR21a	TMHMM2.0	outside	   413   431
PrapIR21a	TMHMM2.0	TMhelix	   432   451
PrapIR21a	TMHMM2.0	inside	   452   462
PrapIR21a	TMHMM2.0	TMhelix	   463   485
PrapIR21a	TMHMM2.0	outside	   486   666
PrapIR21a	TMHMM2.0	TMhelix	   667   689
PrapIR21a	TMHMM2.0	inside	   690   800
```

# plot in postscript,
script for making the plot in gnuplot,
data for plot

---

```
# PrapIR68a Length: 696
# PrapIR68a Number of predicted TMHs:  4
# PrapIR68a Exp number of AAs in TMHs: 93.6234900000001
# PrapIR68a Exp number, first 60 AAs:  4.41209
# PrapIR68a Total prob of N-in:        0.18823
PrapIR68a	TMHMM2.0	outside	     1   357
PrapIR68a	TMHMM2.0	TMhelix	   358   380
PrapIR68a	TMHMM2.0	inside	   381   405
PrapIR68a	TMHMM2.0	TMhelix	   406   428
PrapIR68a	TMHMM2.0	outside	   429   437
PrapIR68a	TMHMM2.0	TMhelix	   438   457
PrapIR68a	TMHMM2.0	inside	   458   643
PrapIR68a	TMHMM2.0	TMhelix	   644   666
PrapIR68a	TMHMM2.0	outside	   667   696
```

# plot in postscript,
script for making the plot in gnuplot,
data for plot

---

```
# PrapIR40a Length: 668
# PrapIR40a Number of predicted TMHs:  3
# PrapIR40a Exp number of AAs in TMHs: 68.9182900000001
# PrapIR40a Exp number, first 60 AAs:  0.00657
# PrapIR40a Total prob of N-in:        0.00338
PrapIR40a	TMHMM2.0	outside	     1   311
PrapIR40a	TMHMM2.0	TMhelix	   312   334
PrapIR40a	TMHMM2.0	inside	   335   377
PrapIR40a	TMHMM2.0	TMhelix	   378   397
PrapIR40a	TMHMM2.0	outside	   398   594
PrapIR40a	TMHMM2.0	TMhelix	   595   617
PrapIR40a	TMHMM2.0	inside	   618   668
```

# plot in postscript,
script for making the plot in gnuplot,
data for plot

---

```
# PrapIR41a Length: 590
# PrapIR41a Number of predicted TMHs:  2
# PrapIR41a Exp number of AAs in TMHs: 62.3132400000001
# PrapIR41a Exp number, first 60 AAs:  0.000880000000000001
# PrapIR41a Total prob of N-in:        0.00088
PrapIR41a	TMHMM2.0	outside	     1   308
PrapIR41a	TMHMM2.0	TMhelix	   309   331
PrapIR41a	TMHMM2.0	inside	   332   554
PrapIR41a	TMHMM2.0	TMhelix	   555   577
PrapIR41a	TMHMM2.0	outside	   578   590
```

# plot in postscript,
script for making the plot in gnuplot,
data for plot

---

```
# PrapIR76b2 Length: 532
# PrapIR76b2 Number of predicted TMHs:  4
# PrapIR76b2 Exp number of AAs in TMHs: 80.09324
# PrapIR76b2 Exp number, first 60 AAs:  0.0188
# PrapIR76b2 Total prob of N-in:        0.02067
PrapIR76b2	TMHMM2.0	outside	     1   166
PrapIR76b2	TMHMM2.0	TMhelix	   167   186
PrapIR76b2	TMHMM2.0	inside	   187   197
PrapIR76b2	TMHMM2.0	TMhelix	   198   220
PrapIR76b2	TMHMM2.0	outside	   221   229
PrapIR76b2	TMHMM2.0	TMhelix	   230   252
PrapIR76b2	TMHMM2.0	inside	   253   426
PrapIR76b2	TMHMM2.0	TMhelix	   427   449
PrapIR76b2	TMHMM2.0	outside	   450   532
```

# plot in postscript,
script for making the plot in gnuplot,
data for plot

---

```
# PrapIR76b1 Length: 532
# PrapIR76b1 Number of predicted TMHs:  4
# PrapIR76b1 Exp number of AAs in TMHs: 80.41691
# PrapIR76b1 Exp number, first 60 AAs:  0.01017
# PrapIR76b1 Total prob of N-in:        0.02160
PrapIR76b1	TMHMM2.0	outside	     1   166
PrapIR76b1	TMHMM2.0	TMhelix	   167   186
PrapIR76b1	TMHMM2.0	inside	   187   197
PrapIR76b1	TMHMM2.0	TMhelix	   198   220
PrapIR76b1	TMHMM2.0	outside	   221   229
PrapIR76b1	TMHMM2.0	TMhelix	   230   252
PrapIR76b1	TMHMM2.0	inside	   253   426
PrapIR76b1	TMHMM2.0	TMhelix	   427   449
PrapIR76b1	TMHMM2.0	outside	   450   532
```

# plot in postscript,
script for making the plot in gnuplot,
data for plot

---

```
# PrapIR93a Length: 867
# PrapIR93a Number of predicted TMHs:  3
# PrapIR93a Exp number of AAs in TMHs: 81.18398
# PrapIR93a Exp number, first 60 AAs:  0.00497
# PrapIR93a Total prob of N-in:        0.00389
PrapIR93a	TMHMM2.0	outside	     1   554
PrapIR93a	TMHMM2.0	TMhelix	   555   577
PrapIR93a	TMHMM2.0	inside	   578   624
PrapIR93a	TMHMM2.0	TMhelix	   625   647
PrapIR93a	TMHMM2.0	outside	   648   827
PrapIR93a	TMHMM2.0	TMhelix	   828   850
PrapIR93a	TMHMM2.0	inside	   851   867
```

# plot in postscript,
script for making the plot in gnuplot,
data for plot

---

```
# PrapIR8a Length: 852
# PrapIR8a Number of predicted TMHs:  3
# PrapIR8a Exp number of AAs in TMHs: 82.2741
# PrapIR8a Exp number, first 60 AAs:  16.72575
# PrapIR8a Total prob of N-in:        0.77028
# PrapIR8a POSSIBLE N-term signal sequence
PrapIR8a	TMHMM2.0	outside	     1   490
PrapIR8a	TMHMM2.0	TMhelix	   491   509
PrapIR8a	TMHMM2.0	inside	   510   563
PrapIR8a	TMHMM2.0	TMhelix	   564   586
PrapIR8a	TMHMM2.0	outside	   587   778
PrapIR8a	TMHMM2.0	TMhelix	   779   801
PrapIR8a	TMHMM2.0	inside	   802   852
```

# plot in postscript,
script for making the plot in gnuplot,
data for plot

---

```
# PrapIR25a1 Length: 922
# PrapIR25a1 Number of predicted TMHs:  3
# PrapIR25a1 Exp number of AAs in TMHs: 64.9720200000001
# PrapIR25a1 Exp number, first 60 AAs:  0.00799
# PrapIR25a1 Total prob of N-in:        0.00198
PrapIR25a1	TMHMM2.0	outside	     1   550
PrapIR25a1	TMHMM2.0	TMhelix	   551   569
PrapIR25a1	TMHMM2.0	inside	   570   625
PrapIR25a1	TMHMM2.0	TMhelix	   626   648
PrapIR25a1	TMHMM2.0	outside	   649   848
PrapIR25a1	TMHMM2.0	TMhelix	   849   871
PrapIR25a1	TMHMM2.0	inside	   872   922
```

# plot in postscript,
script for making the plot in gnuplot,
data for plot

---

```
# PrapIR25a2 Length: 909
# PrapIR25a2 Number of predicted TMHs:  3
# PrapIR25a2 Exp number of AAs in TMHs: 80.5117600000001
# PrapIR25a2 Exp number, first 60 AAs:  0.0396500000000001
# PrapIR25a2 Total prob of N-in:        0.00525
PrapIR25a2	TMHMM2.0	outside	     1   548
PrapIR25a2	TMHMM2.0	TMhelix	   549   567
PrapIR25a2	TMHMM2.0	inside	   568   623
PrapIR25a2	TMHMM2.0	TMhelix	   624   646
PrapIR25a2	TMHMM2.0	outside	   647   844
PrapIR25a2	TMHMM2.0	TMhelix	   845   867
PrapIR25a2	TMHMM2.0	inside	   868   909
```

# plot in postscript,
script for making the plot in gnuplot,
data for plot

---

```
# PrapIR85a Length: 633
# PrapIR85a Number of predicted TMHs:  2
# PrapIR85a Exp number of AAs in TMHs: 70.2934
# PrapIR85a Exp number, first 60 AAs:  3.99849
# PrapIR85a Total prob of N-in:        0.25382
PrapIR85a	TMHMM2.0	outside	     1   335
PrapIR85a	TMHMM2.0	TMhelix	   336   358
PrapIR85a	TMHMM2.0	inside	   359   593
PrapIR85a	TMHMM2.0	TMhelix	   594   616
PrapIR85a	TMHMM2.0	outside	   617   633
```

# plot in postscript,
script for making the plot in gnuplot,
data for plot

---

```
# PrapIR143 Length: 610
# PrapIR143 Number of predicted TMHs:  3
# PrapIR143 Exp number of AAs in TMHs: 68.9410800000001
# PrapIR143 Exp number, first 60 AAs:  0.0338900000000001
# PrapIR143 Total prob of N-in:        0.09294
PrapIR143	TMHMM2.0	outside	     1   332
PrapIR143	TMHMM2.0	TMhelix	   333   350
PrapIR143	TMHMM2.0	inside	   351   393
PrapIR143	TMHMM2.0	TMhelix	   394   416
PrapIR143	TMHMM2.0	outside	   417   582
PrapIR143	TMHMM2.0	TMhelix	   583   605
PrapIR143	TMHMM2.0	inside	   606   610
```

# plot in postscript,
script for making the plot in gnuplot,
data for plot

---

```
# PrapIR7d.3 Length: 595
# PrapIR7d.3 Number of predicted TMHs:  3
# PrapIR7d.3 Exp number of AAs in TMHs: 64.80558
# PrapIR7d.3 Exp number, first 60 AAs:  0.00668
# PrapIR7d.3 Total prob of N-in:        0.00172
PrapIR7d.3	TMHMM2.0	outside	     1   311
PrapIR7d.3	TMHMM2.0	TMhelix	   312   334
PrapIR7d.3	TMHMM2.0	inside	   335   362
PrapIR7d.3	TMHMM2.0	TMhelix	   363   385
PrapIR7d.3	TMHMM2.0	outside	   386   550
PrapIR7d.3	TMHMM2.0	TMhelix	   551   573
PrapIR7d.3	TMHMM2.0	inside	   574   595
```

# plot in postscript,
script for making the plot in gnuplot,
data for plot

---

```
# PrapIR7d.2 Length: 593
# PrapIR7d.2 Number of predicted TMHs:  3
# PrapIR7d.2 Exp number of AAs in TMHs: 88.30449
# PrapIR7d.2 Exp number, first 60 AAs:  15.02878
# PrapIR7d.2 Total prob of N-in:        0.06272
# PrapIR7d.2 POSSIBLE N-term signal sequence
PrapIR7d.2	TMHMM2.0	outside	     1   317
PrapIR7d.2	TMHMM2.0	TMhelix	   318   340
PrapIR7d.2	TMHMM2.0	inside	   341   377
PrapIR7d.2	TMHMM2.0	TMhelix	   378   400
PrapIR7d.2	TMHMM2.0	outside	   401   547
PrapIR7d.2	TMHMM2.0	TMhelix	   548   570
PrapIR7d.2	TMHMM2.0	inside	   571   593
```

# plot in postscript,
script for making the plot in gnuplot,
data for plot

---

```
# PrapIR7d.2.1 Length: 583
# PrapIR7d.2.1 Number of predicted TMHs:  4
# PrapIR7d.2.1 Exp number of AAs in TMHs: 80.78282
# PrapIR7d.2.1 Exp number, first 60 AAs:  1.68448
# PrapIR7d.2.1 Total prob of N-in:        0.04799
PrapIR7d.2.1	TMHMM2.0	outside	     1   312
PrapIR7d.2.1	TMHMM2.0	TMhelix	   313   331
PrapIR7d.2.1	TMHMM2.0	inside	   332   343
PrapIR7d.2.1	TMHMM2.0	TMhelix	   344   366
PrapIR7d.2.1	TMHMM2.0	outside	   367   375
PrapIR7d.2.1	TMHMM2.0	TMhelix	   376   398
PrapIR7d.2.1	TMHMM2.0	inside	   399   543
PrapIR7d.2.1	TMHMM2.0	TMhelix	   544   566
PrapIR7d.2.1	TMHMM2.0	outside	   567   583
```

# plot in postscript,
script for making the plot in gnuplot,
data for plot

---

```
# PrapIR87a Length: 637
# PrapIR87a Number of predicted TMHs:  4
# PrapIR87a Exp number of AAs in TMHs: 92.2791500000002
# PrapIR87a Exp number, first 60 AAs:  4.97905
# PrapIR87a Total prob of N-in:        0.30669
PrapIR87a	TMHMM2.0	inside	     1   354
PrapIR87a	TMHMM2.0	TMhelix	   355   377
PrapIR87a	TMHMM2.0	outside	   378   391
PrapIR87a	TMHMM2.0	TMhelix	   392   411
PrapIR87a	TMHMM2.0	inside	   412   417
PrapIR87a	TMHMM2.0	TMhelix	   418   440
PrapIR87a	TMHMM2.0	outside	   441   601
PrapIR87a	TMHMM2.0	TMhelix	   602   624
PrapIR87a	TMHMM2.0	inside	   625   637
```

# plot in postscript,
script for making the plot in gnuplot,
data for plot

---

```
# PrapIR100c Length: 580
# PrapIR100c Number of predicted TMHs:  3
# PrapIR100c Exp number of AAs in TMHs: 67.45479
# PrapIR100c Exp number, first 60 AAs:  0.05457
# PrapIR100c Total prob of N-in:        0.02227
PrapIR100c	TMHMM2.0	outside	     1   319
PrapIR100c	TMHMM2.0	TMhelix	   320   339
PrapIR100c	TMHMM2.0	inside	   340   370
PrapIR100c	TMHMM2.0	TMhelix	   371   393
PrapIR100c	TMHMM2.0	outside	   394   549
PrapIR100c	TMHMM2.0	TMhelix	   550   572
PrapIR100c	TMHMM2.0	inside	   573   580
```

# plot in postscript,
script for making the plot in gnuplot,
data for plot

---

```
# PrapIR100d Length: 579
# PrapIR100d Number of predicted TMHs:  3
# PrapIR100d Exp number of AAs in TMHs: 81.61498
# PrapIR100d Exp number, first 60 AAs:  0.71341
# PrapIR100d Total prob of N-in:        0.12107
PrapIR100d	TMHMM2.0	outside	     1   307
PrapIR100d	TMHMM2.0	TMhelix	   308   330
PrapIR100d	TMHMM2.0	inside	   331   360
PrapIR100d	TMHMM2.0	TMhelix	   361   383
PrapIR100d	TMHMM2.0	outside	   384   545
PrapIR100d	TMHMM2.0	TMhelix	   546   568
PrapIR100d	TMHMM2.0	inside	   569   579
```

# plot in postscript,
script for making the plot in gnuplot,
data for plot

---

```
# PrapIR100f Length: 609
# PrapIR100f Number of predicted TMHs:  4
# PrapIR100f Exp number of AAs in TMHs: 81.8053299999999
# PrapIR100f Exp number, first 60 AAs:  17.4076
# PrapIR100f Total prob of N-in:        0.66831
# PrapIR100f POSSIBLE N-term signal sequence
PrapIR100f	TMHMM2.0	inside	     1    33
PrapIR100f	TMHMM2.0	TMhelix	    34    56
PrapIR100f	TMHMM2.0	outside	    57   352
PrapIR100f	TMHMM2.0	TMhelix	   353   375
PrapIR100f	TMHMM2.0	inside	   376   406
PrapIR100f	TMHMM2.0	TMhelix	   407   429
PrapIR100f	TMHMM2.0	outside	   430   585
PrapIR100f	TMHMM2.0	TMhelix	   586   605
PrapIR100f	TMHMM2.0	inside	   606   609
```

# plot in postscript,
script for making the plot in gnuplot,
data for plot

---

```
# PrapIR100e Length: 569
# PrapIR100e Number of predicted TMHs:  2
# PrapIR100e Exp number of AAs in TMHs: 55.88931
# PrapIR100e Exp number, first 60 AAs:  11.16991
# PrapIR100e Total prob of N-in:        0.51977
# PrapIR100e POSSIBLE N-term signal sequence
PrapIR100e	TMHMM2.0	outside	     1   325
PrapIR100e	TMHMM2.0	TMhelix	   326   348
PrapIR100e	TMHMM2.0	inside	   349   379
PrapIR100e	TMHMM2.0	TMhelix	   380   402
PrapIR100e	TMHMM2.0	outside	   403   569
```

# plot in postscript,
script for making the plot in gnuplot,
data for plot

---

```
# PrapIR31a Length: 585
# PrapIR31a Number of predicted TMHs:  3
# PrapIR31a Exp number of AAs in TMHs: 79.3642700000002
# PrapIR31a Exp number, first 60 AAs:  11.31137
# PrapIR31a Total prob of N-in:        0.52976
# PrapIR31a POSSIBLE N-term signal sequence
PrapIR31a	TMHMM2.0	outside	     1   296
PrapIR31a	TMHMM2.0	TMhelix	   297   319
PrapIR31a	TMHMM2.0	inside	   320   359
PrapIR31a	TMHMM2.0	TMhelix	   360   382
PrapIR31a	TMHMM2.0	outside	   383   545
PrapIR31a	TMHMM2.0	TMhelix	   546   568
PrapIR31a	TMHMM2.0	inside	   569   585
```

# plot in postscript,
script for making the plot in gnuplot,
data for plot

---

```
# PrapIR4 Length: 640
# PrapIR4 Number of predicted TMHs:  2
# PrapIR4 Exp number of AAs in TMHs: 53.3194100000001
# PrapIR4 Exp number, first 60 AAs:  0.00984
# PrapIR4 Total prob of N-in:        0.02888
PrapIR4	TMHMM2.0	outside	     1   372
PrapIR4	TMHMM2.0	TMhelix	   373   395
PrapIR4	TMHMM2.0	inside	   396   432
PrapIR4	TMHMM2.0	TMhelix	   433   455
PrapIR4	TMHMM2.0	outside	   456   640
```

# plot in postscript,
script for making the plot in gnuplot,
data for plot

---

```
# PrapIR2 Length: 400
# PrapIR2 Number of predicted TMHs:  3
# PrapIR2 Exp number of AAs in TMHs: 72.76339
# PrapIR2 Exp number, first 60 AAs:  0.1228
# PrapIR2 Total prob of N-in:        0.64118
PrapIR2	TMHMM2.0	inside	     1   109
PrapIR2	TMHMM2.0	TMhelix	   110   132
PrapIR2	TMHMM2.0	outside	   133   180
PrapIR2	TMHMM2.0	TMhelix	   181   203
PrapIR2	TMHMM2.0	inside	   204   362
PrapIR2	TMHMM2.0	TMhelix	   363   385
PrapIR2	TMHMM2.0	outside	   386   400
```

# plot in postscript,
script for making the plot in gnuplot,
data for plot

---

```
# PrapIR64a Length: 598
# PrapIR64a Number of predicted TMHs:  3
# PrapIR64a Exp number of AAs in TMHs: 70.36227
# PrapIR64a Exp number, first 60 AAs:  3.45603
# PrapIR64a Total prob of N-in:        0.16970
PrapIR64a	TMHMM2.0	outside	     1   309
PrapIR64a	TMHMM2.0	TMhelix	   310   332
PrapIR64a	TMHMM2.0	inside	   333   368
PrapIR64a	TMHMM2.0	TMhelix	   369   391
PrapIR64a	TMHMM2.0	outside	   392   566
PrapIR64a	TMHMM2.0	TMhelix	   567   589
PrapIR64a	TMHMM2.0	inside	   590   598
```

# plot in postscript,
script for making the plot in gnuplot,
data for plot

---

```
# PrapIR1.1 Length: 651
# PrapIR1.1 Number of predicted TMHs:  3
# PrapIR1.1 Exp number of AAs in TMHs: 81.94801
# PrapIR1.1 Exp number, first 60 AAs:  13.26472
# PrapIR1.1 Total prob of N-in:        0.48457
# PrapIR1.1 POSSIBLE N-term signal sequence
PrapIR1.1	TMHMM2.0	outside	     1   340
PrapIR1.1	TMHMM2.0	TMhelix	   341   363
PrapIR1.1	TMHMM2.0	inside	   364   405
PrapIR1.1	TMHMM2.0	TMhelix	   406   428
PrapIR1.1	TMHMM2.0	outside	   429   603
PrapIR1.1	TMHMM2.0	TMhelix	   604   626
PrapIR1.1	TMHMM2.0	inside	   627   651
```

# plot in postscript,
script for making the plot in gnuplot,
data for plot

---

```
# PrapIR1.2 Length: 623
# PrapIR1.2 Number of predicted TMHs:  3
# PrapIR1.2 Exp number of AAs in TMHs: 64.89462
# PrapIR1.2 Exp number, first 60 AAs:  0.0659
# PrapIR1.2 Total prob of N-in:        0.00392
PrapIR1.2	TMHMM2.0	outside	     1   328
PrapIR1.2	TMHMM2.0	TMhelix	   329   348
PrapIR1.2	TMHMM2.0	inside	   349   391
PrapIR1.2	TMHMM2.0	TMhelix	   392   414
PrapIR1.2	TMHMM2.0	outside	   415   579
PrapIR1.2	TMHMM2.0	TMhelix	   580   602
PrapIR1.2	TMHMM2.0	inside	   603   623
```

# plot in postscript,
script for making the plot in gnuplot,
data for plot

---

```
# PrapIR75d Length: 599
# PrapIR75d Number of predicted TMHs:  3
# PrapIR75d Exp number of AAs in TMHs: 67.21787
# PrapIR75d Exp number, first 60 AAs:  0.510110000000001
# PrapIR75d Total prob of N-in:        0.02181
PrapIR75d	TMHMM2.0	outside	     1   315
PrapIR75d	TMHMM2.0	TMhelix	   316   338
PrapIR75d	TMHMM2.0	inside	   339   374
PrapIR75d	TMHMM2.0	TMhelix	   375   397
PrapIR75d	TMHMM2.0	outside	   398   567
PrapIR75d	TMHMM2.0	TMhelix	   568   590
PrapIR75d	TMHMM2.0	inside	   591   599
```

# plot in postscript,
script for making the plot in gnuplot,
data for plot

---

```
# PrapIR75q.1 Length: 615
# PrapIR75q.1 Number of predicted TMHs:  3
# PrapIR75q.1 Exp number of AAs in TMHs: 70.85769
# PrapIR75q.1 Exp number, first 60 AAs:  1.21493
# PrapIR75q.1 Total prob of N-in:        0.11580
PrapIR75q.1	TMHMM2.0	outside	     1   321
PrapIR75q.1	TMHMM2.0	TMhelix	   322   344
PrapIR75q.1	TMHMM2.0	inside	   345   389
PrapIR75q.1	TMHMM2.0	TMhelix	   390   412
PrapIR75q.1	TMHMM2.0	outside	   413   580
PrapIR75q.1	TMHMM2.0	TMhelix	   581   603
PrapIR75q.1	TMHMM2.0	inside	   604   615
```

# plot in postscript,
script for making the plot in gnuplot,
data for plot

---

```
# PrapIR75q.2 Length: 639
# PrapIR75q.2 Number of predicted TMHs:  3
# PrapIR75q.2 Exp number of AAs in TMHs: 71.73161
# PrapIR75q.2 Exp number, first 60 AAs:  3.47712
# PrapIR75q.2 Total prob of N-in:        0.23110
PrapIR75q.2	TMHMM2.0	outside	     1   328
PrapIR75q.2	TMHMM2.0	TMhelix	   329   351
PrapIR75q.2	TMHMM2.0	inside	   352   399
PrapIR75q.2	TMHMM2.0	TMhelix	   400   422
PrapIR75q.2	TMHMM2.0	outside	   423   589
PrapIR75q.2	TMHMM2.0	TMhelix	   590   612
PrapIR75q.2	TMHMM2.0	inside	   613   639
```

# plot in postscript,
script for making the plot in gnuplot,
data for plot

---

```
# PrapIR75p.2 Length: 608
# PrapIR75p.2 Number of predicted TMHs:  3
# PrapIR75p.2 Exp number of AAs in TMHs: 71.7869900000001
# PrapIR75p.2 Exp number, first 60 AAs:  3.13176
# PrapIR75p.2 Total prob of N-in:        0.17071
PrapIR75p.2	TMHMM2.0	outside	     1   323
PrapIR75p.2	TMHMM2.0	TMhelix	   324   346
PrapIR75p.2	TMHMM2.0	inside	   347   390
PrapIR75p.2	TMHMM2.0	TMhelix	   391   413
PrapIR75p.2	TMHMM2.0	outside	   414   579
PrapIR75p.2	TMHMM2.0	TMhelix	   580   602
PrapIR75p.2	TMHMM2.0	inside	   603   608
```

# plot in postscript,
script for making the plot in gnuplot,
data for plot

---

```
# PrapIR75p.1 Length: 638
# PrapIR75p.1 Number of predicted TMHs:  3
# PrapIR75p.1 Exp number of AAs in TMHs: 69.81763
# PrapIR75p.1 Exp number, first 60 AAs:  0.66431
# PrapIR75p.1 Total prob of N-in:        0.04124
PrapIR75p.1	TMHMM2.0	outside	     1   343
PrapIR75p.1	TMHMM2.0	TMhelix	   344   366
PrapIR75p.1	TMHMM2.0	inside	   367   411
PrapIR75p.1	TMHMM2.0	TMhelix	   412   434
PrapIR75p.1	TMHMM2.0	outside	   435   598
PrapIR75p.1	TMHMM2.0	TMhelix	   599   621
PrapIR75p.1	TMHMM2.0	inside	   622   638
```

# plot in postscript,
script for making the plot in gnuplot,
data for plot

---

```
# PrapIR7d.2.2 Length: 596
# PrapIR7d.2.2 Number of predicted TMHs:  5
# PrapIR7d.2.2 Exp number of AAs in TMHs: 129.98648
# PrapIR7d.2.2 Exp number, first 60 AAs:  22.11813
# PrapIR7d.2.2 Total prob of N-in:        0.01667
# PrapIR7d.2.2 POSSIBLE N-term signal sequence
PrapIR7d.2.2	TMHMM2.0	outside	     1    33
PrapIR7d.2.2	TMHMM2.0	TMhelix	    34    56
PrapIR7d.2.2	TMHMM2.0	inside	    57   318
PrapIR7d.2.2	TMHMM2.0	TMhelix	   319   338
PrapIR7d.2.2	TMHMM2.0	outside	   339   357
PrapIR7d.2.2	TMHMM2.0	TMhelix	   358   377
PrapIR7d.2.2	TMHMM2.0	inside	   378   383
PrapIR7d.2.2	TMHMM2.0	TMhelix	   384   406
PrapIR7d.2.2	TMHMM2.0	outside	   407   560
PrapIR7d.2.2	TMHMM2.0	TMhelix	   561   583
PrapIR7d.2.2	TMHMM2.0	inside	   584   596
```

# plot in postscript,
script for making the plot in gnuplot,
data for plot

---

```
# PrapIR7d.4 Length: 380
# PrapIR7d.4 Number of predicted TMHs:  1
# PrapIR7d.4 Exp number of AAs in TMHs: 52.7356599999999
# PrapIR7d.4 Exp number, first 60 AAs:  14.38215
# PrapIR7d.4 Total prob of N-in:        0.28946
# PrapIR7d.4 POSSIBLE N-term signal sequence
PrapIR7d.4	TMHMM2.0	outside	     1   353
PrapIR7d.4	TMHMM2.0	TMhelix	   354   376
PrapIR7d.4	TMHMM2.0	inside	   377   380
```

# plot in postscript,
script for making the plot in gnuplot,
data for plot

---

```
# PbraGR54 Length: 361
# PbraGR54 Number of predicted TMHs:  7
# PbraGR54 Exp number of AAs in TMHs: 146.49288
# PbraGR54 Exp number, first 60 AAs:  38.38192
# PbraGR54 Total prob of N-in:        0.92823
# PbraGR54 POSSIBLE N-term signal sequence
PbraGR54	TMHMM2.0	inside	     1    11
PbraGR54	TMHMM2.0	TMhelix	    12    31
PbraGR54	TMHMM2.0	outside	    32    40
PbraGR54	TMHMM2.0	TMhelix	    41    63
PbraGR54	TMHMM2.0	inside	    64   114
PbraGR54	TMHMM2.0	TMhelix	   115   132
PbraGR54	TMHMM2.0	outside	   133   141
PbraGR54	TMHMM2.0	TMhelix	   142   164
PbraGR54	TMHMM2.0	inside	   165   231
PbraGR54	TMHMM2.0	TMhelix	   232   254
PbraGR54	TMHMM2.0	outside	   255   263
PbraGR54	TMHMM2.0	TMhelix	   264   286
PbraGR54	TMHMM2.0	inside	   287   332
PbraGR54	TMHMM2.0	TMhelix	   333   355
PbraGR54	TMHMM2.0	outside	   356   361
```

# plot in postscript,
script for making the plot in gnuplot,
data for plot

---

```
# PbraGR29 Length: 347
# PbraGR29 Number of predicted TMHs:  7
# PbraGR29 Exp number of AAs in TMHs: 154.95787
# PbraGR29 Exp number, first 60 AAs:  21.27526
# PbraGR29 Total prob of N-in:        0.87062
# PbraGR29 POSSIBLE N-term signal sequence
PbraGR29	TMHMM2.0	inside	     1    37
PbraGR29	TMHMM2.0	TMhelix	    38    55
PbraGR29	TMHMM2.0	outside	    56    69
PbraGR29	TMHMM2.0	TMhelix	    70    92
PbraGR29	TMHMM2.0	inside	    93    98
PbraGR29	TMHMM2.0	TMhelix	    99   121
PbraGR29	TMHMM2.0	outside	   122   130
PbraGR29	TMHMM2.0	TMhelix	   131   152
PbraGR29	TMHMM2.0	inside	   153   213
PbraGR29	TMHMM2.0	TMhelix	   214   236
PbraGR29	TMHMM2.0	outside	   237   240
PbraGR29	TMHMM2.0	TMhelix	   241   263
PbraGR29	TMHMM2.0	inside	   264   312
PbraGR29	TMHMM2.0	TMhelix	   313   335
PbraGR29	TMHMM2.0	outside	   336   347
```

# plot in postscript,
script for making the plot in gnuplot,
data for plot

---

```
# PbraGR30 Length: 349
# PbraGR30 Number of predicted TMHs:  6
# PbraGR30 Exp number of AAs in TMHs: 148.59758
# PbraGR30 Exp number, first 60 AAs:  20.89907
# PbraGR30 Total prob of N-in:        0.50255
# PbraGR30 POSSIBLE N-term signal sequence
PbraGR30	TMHMM2.0	outside	     1    56
PbraGR30	TMHMM2.0	TMhelix	    57    76
PbraGR30	TMHMM2.0	inside	    77   104
PbraGR30	TMHMM2.0	TMhelix	   105   127
PbraGR30	TMHMM2.0	outside	   128   136
PbraGR30	TMHMM2.0	TMhelix	   137   159
PbraGR30	TMHMM2.0	inside	   160   213
PbraGR30	TMHMM2.0	TMhelix	   214   236
PbraGR30	TMHMM2.0	outside	   237   250
PbraGR30	TMHMM2.0	TMhelix	   251   273
PbraGR30	TMHMM2.0	inside	   274   315
PbraGR30	TMHMM2.0	TMhelix	   316   338
PbraGR30	TMHMM2.0	outside	   339   349
```

# plot in postscript,
script for making the plot in gnuplot,
data for plot

---

```
# PbraGR8.1 Length: 374
# PbraGR8.1 Number of predicted TMHs:  6
# PbraGR8.1 Exp number of AAs in TMHs: 139.90043
# PbraGR8.1 Exp number, first 60 AAs:  16.37368
# PbraGR8.1 Total prob of N-in:        0.90650
# PbraGR8.1 POSSIBLE N-term signal sequence
PbraGR8.1	TMHMM2.0	inside	     1    45
PbraGR8.1	TMHMM2.0	TMhelix	    46    68
PbraGR8.1	TMHMM2.0	outside	    69    77
PbraGR8.1	TMHMM2.0	TMhelix	    78   100
PbraGR8.1	TMHMM2.0	inside	   101   126
PbraGR8.1	TMHMM2.0	TMhelix	   127   149
PbraGR8.1	TMHMM2.0	outside	   150   158
PbraGR8.1	TMHMM2.0	TMhelix	   159   178
PbraGR8.1	TMHMM2.0	inside	   179   316
PbraGR8.1	TMHMM2.0	TMhelix	   317   339
PbraGR8.1	TMHMM2.0	outside	   340   348
PbraGR8.1	TMHMM2.0	TMhelix	   349   371
PbraGR8.1	TMHMM2.0	inside	   372   374
```

# plot in postscript,
script for making the plot in gnuplot,
data for plot

---

```
# PbraGR8.2 Length: 385
# PbraGR8.2 Number of predicted TMHs:  6
# PbraGR8.2 Exp number of AAs in TMHs: 152.44505
# PbraGR8.2 Exp number, first 60 AAs:  30.04303
# PbraGR8.2 Total prob of N-in:        0.49217
# PbraGR8.2 POSSIBLE N-term signal sequence
PbraGR8.2	TMHMM2.0	inside	     1    43
PbraGR8.2	TMHMM2.0	TMhelix	    44    66
PbraGR8.2	TMHMM2.0	outside	    67    85
PbraGR8.2	TMHMM2.0	TMhelix	    86   108
PbraGR8.2	TMHMM2.0	inside	   109   127
PbraGR8.2	TMHMM2.0	TMhelix	   128   147
PbraGR8.2	TMHMM2.0	outside	   148   151
PbraGR8.2	TMHMM2.0	TMhelix	   152   171
PbraGR8.2	TMHMM2.0	inside	   172   239
PbraGR8.2	TMHMM2.0	TMhelix	   240   262
PbraGR8.2	TMHMM2.0	outside	   263   354
PbraGR8.2	TMHMM2.0	TMhelix	   355   377
PbraGR8.2	TMHMM2.0	inside	   378   385
```

# plot in postscript,
script for making the plot in gnuplot,
data for plot

---

```
# PbraGR8.3 Length: 380
# PbraGR8.3 Number of predicted TMHs:  3
# PbraGR8.3 Exp number of AAs in TMHs: 109.37362
# PbraGR8.3 Exp number, first 60 AAs:  14.05528
# PbraGR8.3 Total prob of N-in:        0.34081
# PbraGR8.3 POSSIBLE N-term signal sequence
PbraGR8.3	TMHMM2.0	outside	     1    47
PbraGR8.3	TMHMM2.0	TMhelix	    48    70
PbraGR8.3	TMHMM2.0	inside	    71   124
PbraGR8.3	TMHMM2.0	TMhelix	   125   147
PbraGR8.3	TMHMM2.0	outside	   148   354
PbraGR8.3	TMHMM2.0	TMhelix	   355   377
PbraGR8.3	TMHMM2.0	inside	   378   380
```

# plot in postscript,
script for making the plot in gnuplot,
data for plot

---

```
# PbraGR28 Length: 383
# PbraGR28 Number of predicted TMHs:  6
# PbraGR28 Exp number of AAs in TMHs: 149.63786
# PbraGR28 Exp number, first 60 AAs:  27.5055
# PbraGR28 Total prob of N-in:        0.30556
# PbraGR28 POSSIBLE N-term signal sequence
PbraGR28	TMHMM2.0	inside	     1    46
PbraGR28	TMHMM2.0	TMhelix	    47    69
PbraGR28	TMHMM2.0	outside	    70    72
PbraGR28	TMHMM2.0	TMhelix	    73    95
PbraGR28	TMHMM2.0	inside	    96   166
PbraGR28	TMHMM2.0	TMhelix	   167   184
PbraGR28	TMHMM2.0	outside	   185   237
PbraGR28	TMHMM2.0	TMhelix	   238   260
PbraGR28	TMHMM2.0	inside	   261   266
PbraGR28	TMHMM2.0	TMhelix	   267   289
PbraGR28	TMHMM2.0	outside	   290   343
PbraGR28	TMHMM2.0	TMhelix	   344   366
PbraGR28	TMHMM2.0	inside	   367   383
```

# plot in postscript,
script for making the plot in gnuplot,
data for plot

---

```
# PbraGR1 Length: 457
# PbraGR1 Number of predicted TMHs:  7
# PbraGR1 Exp number of AAs in TMHs: 161.95572
# PbraGR1 Exp number, first 60 AAs:  2.09608
# PbraGR1 Total prob of N-in:        0.43702
PbraGR1	TMHMM2.0	inside	     1    91
PbraGR1	TMHMM2.0	TMhelix	    92   114
PbraGR1	TMHMM2.0	outside	   115   133
PbraGR1	TMHMM2.0	TMhelix	   134   156
PbraGR1	TMHMM2.0	inside	   157   187
PbraGR1	TMHMM2.0	TMhelix	   188   210
PbraGR1	TMHMM2.0	outside	   211   219
PbraGR1	TMHMM2.0	TMhelix	   220   242
PbraGR1	TMHMM2.0	inside	   243   290
PbraGR1	TMHMM2.0	TMhelix	   291   313
PbraGR1	TMHMM2.0	outside	   314   327
PbraGR1	TMHMM2.0	TMhelix	   328   345
PbraGR1	TMHMM2.0	inside	   346   404
PbraGR1	TMHMM2.0	TMhelix	   405   427
PbraGR1	TMHMM2.0	outside	   428   457
```

# plot in postscript,
script for making the plot in gnuplot,
data for plot

---

```
# PbraGR2 Length: 430
# PbraGR2 Number of predicted TMHs:  7
# PbraGR2 Exp number of AAs in TMHs: 145.42011
# PbraGR2 Exp number, first 60 AAs:  0.01524
# PbraGR2 Total prob of N-in:        0.79211
PbraGR2	TMHMM2.0	inside	     1    97
PbraGR2	TMHMM2.0	TMhelix	    98   120
PbraGR2	TMHMM2.0	outside	   121   129
PbraGR2	TMHMM2.0	TMhelix	   130   152
PbraGR2	TMHMM2.0	inside	   153   186
PbraGR2	TMHMM2.0	TMhelix	   187   209
PbraGR2	TMHMM2.0	outside	   210   218
PbraGR2	TMHMM2.0	TMhelix	   219   236
PbraGR2	TMHMM2.0	inside	   237   289
PbraGR2	TMHMM2.0	TMhelix	   290   309
PbraGR2	TMHMM2.0	outside	   310   323
PbraGR2	TMHMM2.0	TMhelix	   324   341
PbraGR2	TMHMM2.0	inside	   342   400
PbraGR2	TMHMM2.0	TMhelix	   401   423
PbraGR2	TMHMM2.0	outside	   424   430
```

# plot in postscript,
script for making the plot in gnuplot,
data for plot

---

```
# PbraGR3 Length: 474
# PbraGR3 Number of predicted TMHs:  8
# PbraGR3 Exp number of AAs in TMHs: 164.30349
# PbraGR3 Exp number, first 60 AAs:  0.00025
# PbraGR3 Total prob of N-in:        0.86260
PbraGR3	TMHMM2.0	inside	     1    96
PbraGR3	TMHMM2.0	TMhelix	    97   119
PbraGR3	TMHMM2.0	outside	   120   138
PbraGR3	TMHMM2.0	TMhelix	   139   161
PbraGR3	TMHMM2.0	inside	   162   195
PbraGR3	TMHMM2.0	TMhelix	   196   218
PbraGR3	TMHMM2.0	outside	   219   222
PbraGR3	TMHMM2.0	TMhelix	   223   245
PbraGR3	TMHMM2.0	inside	   246   291
PbraGR3	TMHMM2.0	TMhelix	   292   314
PbraGR3	TMHMM2.0	outside	   315   328
PbraGR3	TMHMM2.0	TMhelix	   329   346
PbraGR3	TMHMM2.0	inside	   347   405
PbraGR3	TMHMM2.0	TMhelix	   406   428
PbraGR3	TMHMM2.0	outside	   429   450
PbraGR3	TMHMM2.0	TMhelix	   451   468
PbraGR3	TMHMM2.0	inside	   469   474
```

# plot in postscript,
script for making the plot in gnuplot,
data for plot

---

```
# PbraGR8.4 Length: 375
# PbraGR8.4 Number of predicted TMHs:  4
# PbraGR8.4 Exp number of AAs in TMHs: 102.83329
# PbraGR8.4 Exp number, first 60 AAs:  35.11093
# PbraGR8.4 Total prob of N-in:        0.95433
# PbraGR8.4 POSSIBLE N-term signal sequence
PbraGR8.4	TMHMM2.0	inside	     1    12
PbraGR8.4	TMHMM2.0	TMhelix	    13    32
PbraGR8.4	TMHMM2.0	outside	    33    46
PbraGR8.4	TMHMM2.0	TMhelix	    47    66
PbraGR8.4	TMHMM2.0	inside	    67    96
PbraGR8.4	TMHMM2.0	TMhelix	    97   119
PbraGR8.4	TMHMM2.0	outside	   120   138
PbraGR8.4	TMHMM2.0	TMhelix	   139   161
PbraGR8.4	TMHMM2.0	inside	   162   375
```

# plot in postscript,
script for making the plot in gnuplot,
data for plot

---

```
# PbraGR9 Length: 459
# PbraGR9 Number of predicted TMHs:  7
# PbraGR9 Exp number of AAs in TMHs: 146.60954
# PbraGR9 Exp number, first 60 AAs:  0.77196
# PbraGR9 Total prob of N-in:        0.70602
PbraGR9	TMHMM2.0	inside	     1    61
PbraGR9	TMHMM2.0	TMhelix	    62    84
PbraGR9	TMHMM2.0	outside	    85    98
PbraGR9	TMHMM2.0	TMhelix	    99   121
PbraGR9	TMHMM2.0	inside	   122   157
PbraGR9	TMHMM2.0	TMhelix	   158   180
PbraGR9	TMHMM2.0	outside	   181   194
PbraGR9	TMHMM2.0	TMhelix	   195   217
PbraGR9	TMHMM2.0	inside	   218   320
PbraGR9	TMHMM2.0	TMhelix	   321   343
PbraGR9	TMHMM2.0	outside	   344   352
PbraGR9	TMHMM2.0	TMhelix	   353   372
PbraGR9	TMHMM2.0	inside	   373   431
PbraGR9	TMHMM2.0	TMhelix	   432   454
PbraGR9	TMHMM2.0	outside	   455   459
```

# plot in postscript,
script for making the plot in gnuplot,
data for plot

---

```
# PbraGR16 Length: 420
# PbraGR16 Number of predicted TMHs:  6
# PbraGR16 Exp number of AAs in TMHs: 145.935
# PbraGR16 Exp number, first 60 AAs:  25.08317
# PbraGR16 Total prob of N-in:        0.67894
# PbraGR16 POSSIBLE N-term signal sequence
PbraGR16	TMHMM2.0	inside	     1    41
PbraGR16	TMHMM2.0	TMhelix	    42    64
PbraGR16	TMHMM2.0	outside	    65    78
PbraGR16	TMHMM2.0	TMhelix	    79    96
PbraGR16	TMHMM2.0	inside	    97   130
PbraGR16	TMHMM2.0	TMhelix	   131   153
PbraGR16	TMHMM2.0	outside	   154   167
PbraGR16	TMHMM2.0	TMhelix	   168   190
PbraGR16	TMHMM2.0	inside	   191   285
PbraGR16	TMHMM2.0	TMhelix	   286   308
PbraGR16	TMHMM2.0	outside	   309   322
PbraGR16	TMHMM2.0	TMhelix	   323   345
PbraGR16	TMHMM2.0	inside	   346   420
```

# plot in postscript,
script for making the plot in gnuplot,
data for plot

---

```
# PbraGR46 Length: 279
# PbraGR46 Number of predicted TMHs:  5
# PbraGR46 Exp number of AAs in TMHs: 112.19329
# PbraGR46 Exp number, first 60 AAs:  21.97216
# PbraGR46 Total prob of N-in:        0.97364
# PbraGR46 POSSIBLE N-term signal sequence
PbraGR46	TMHMM2.0	inside	     1    29
PbraGR46	TMHMM2.0	TMhelix	    30    52
PbraGR46	TMHMM2.0	outside	    53    61
PbraGR46	TMHMM2.0	TMhelix	    62    81
PbraGR46	TMHMM2.0	inside	    82   115
PbraGR46	TMHMM2.0	TMhelix	   116   138
PbraGR46	TMHMM2.0	outside	   139   160
PbraGR46	TMHMM2.0	TMhelix	   161   183
PbraGR46	TMHMM2.0	inside	   184   226
PbraGR46	TMHMM2.0	TMhelix	   227   249
PbraGR46	TMHMM2.0	outside	   250   279
```

# plot in postscript,
script for making the plot in gnuplot,
data for plot

---

```
# PbraGR10 Length: 435
# PbraGR10 Number of predicted TMHs:  5
# PbraGR10 Exp number of AAs in TMHs: 133.12389
# PbraGR10 Exp number, first 60 AAs:  17.80794
# PbraGR10 Total prob of N-in:        0.99731
# PbraGR10 POSSIBLE N-term signal sequence
PbraGR10	TMHMM2.0	inside	     1    42
PbraGR10	TMHMM2.0	TMhelix	    43    65
PbraGR10	TMHMM2.0	outside	    66    79
PbraGR10	TMHMM2.0	TMhelix	    80   102
PbraGR10	TMHMM2.0	inside	   103   130
PbraGR10	TMHMM2.0	TMhelix	   131   153
PbraGR10	TMHMM2.0	outside	   154   172
PbraGR10	TMHMM2.0	TMhelix	   173   195
PbraGR10	TMHMM2.0	inside	   196   278
PbraGR10	TMHMM2.0	TMhelix	   279   301
PbraGR10	TMHMM2.0	outside	   302   435
```

# plot in postscript,
script for making the plot in gnuplot,
data for plot

---

```
# PbraGR66 Length: 385
# PbraGR66 Number of predicted TMHs:  5
# PbraGR66 Exp number of AAs in TMHs: 114.2025
# PbraGR66 Exp number, first 60 AAs:  22.47242
# PbraGR66 Total prob of N-in:        0.34073
# PbraGR66 POSSIBLE N-term signal sequence
PbraGR66	TMHMM2.0	outside	     1    19
PbraGR66	TMHMM2.0	TMhelix	    20    42
PbraGR66	TMHMM2.0	inside	    43   121
PbraGR66	TMHMM2.0	TMhelix	   122   144
PbraGR66	TMHMM2.0	outside	   145   158
PbraGR66	TMHMM2.0	TMhelix	   159   181
PbraGR66	TMHMM2.0	inside	   182   245
PbraGR66	TMHMM2.0	TMhelix	   246   268
PbraGR66	TMHMM2.0	outside	   269   282
PbraGR66	TMHMM2.0	TMhelix	   283   305
PbraGR66	TMHMM2.0	inside	   306   385
```

# plot in postscript,
script for making the plot in gnuplot,
data for plot

---

```
# PbraGR68.1 Length: 384
# PbraGR68.1 Number of predicted TMHs:  6
# PbraGR68.1 Exp number of AAs in TMHs: 135.95302
# PbraGR68.1 Exp number, first 60 AAs:  23.92731
# PbraGR68.1 Total prob of N-in:        0.09312
# PbraGR68.1 POSSIBLE N-term signal sequence
PbraGR68.1	TMHMM2.0	outside	     1    14
PbraGR68.1	TMHMM2.0	TMhelix	    15    35
PbraGR68.1	TMHMM2.0	inside	    36    55
PbraGR68.1	TMHMM2.0	TMhelix	    56    78
PbraGR68.1	TMHMM2.0	outside	    79    92
PbraGR68.1	TMHMM2.0	TMhelix	    93   115
PbraGR68.1	TMHMM2.0	inside	   116   146
PbraGR68.1	TMHMM2.0	TMhelix	   147   169
PbraGR68.1	TMHMM2.0	outside	   170   188
PbraGR68.1	TMHMM2.0	TMhelix	   189   211
PbraGR68.1	TMHMM2.0	inside	   212   357
PbraGR68.1	TMHMM2.0	TMhelix	   358   380
PbraGR68.1	TMHMM2.0	outside	   381   384
```

# plot in postscript,
script for making the plot in gnuplot,
data for plot

---

```
# PbraGR68.2 Length: 396
# PbraGR68.2 Number of predicted TMHs:  6
# PbraGR68.2 Exp number of AAs in TMHs: 148.73431
# PbraGR68.2 Exp number, first 60 AAs:  21.01871
# PbraGR68.2 Total prob of N-in:        0.04339
# PbraGR68.2 POSSIBLE N-term signal sequence
PbraGR68.2	TMHMM2.0	outside	     1    19
PbraGR68.2	TMHMM2.0	TMhelix	    20    42
PbraGR68.2	TMHMM2.0	inside	    43    61
PbraGR68.2	TMHMM2.0	TMhelix	    62    84
PbraGR68.2	TMHMM2.0	outside	    85    98
PbraGR68.2	TMHMM2.0	TMhelix	    99   121
PbraGR68.2	TMHMM2.0	inside	   122   149
PbraGR68.2	TMHMM2.0	TMhelix	   150   172
PbraGR68.2	TMHMM2.0	outside	   173   191
PbraGR68.2	TMHMM2.0	TMhelix	   192   214
PbraGR68.2	TMHMM2.0	inside	   215   368
PbraGR68.2	TMHMM2.0	TMhelix	   369   391
PbraGR68.2	TMHMM2.0	outside	   392   396
```

# plot in postscript,
script for making the plot in gnuplot,
data for plot

---

```
# PbraGR22 Length: 388
# PbraGR22 Number of predicted TMHs:  8
# PbraGR22 Exp number of AAs in TMHs: 163.2421
# PbraGR22 Exp number, first 60 AAs:  42.6549
# PbraGR22 Total prob of N-in:        0.55118
# PbraGR22 POSSIBLE N-term signal sequence
PbraGR22	TMHMM2.0	outside	     1     3
PbraGR22	TMHMM2.0	TMhelix	     4    26
PbraGR22	TMHMM2.0	inside	    27    32
PbraGR22	TMHMM2.0	TMhelix	    33    55
PbraGR22	TMHMM2.0	outside	    56   105
PbraGR22	TMHMM2.0	TMhelix	   106   128
PbraGR22	TMHMM2.0	inside	   129   132
PbraGR22	TMHMM2.0	TMhelix	   133   155
PbraGR22	TMHMM2.0	outside	   156   164
PbraGR22	TMHMM2.0	TMhelix	   165   187
PbraGR22	TMHMM2.0	inside	   188   254
PbraGR22	TMHMM2.0	TMhelix	   255   277
PbraGR22	TMHMM2.0	outside	   278   286
PbraGR22	TMHMM2.0	TMhelix	   287   309
PbraGR22	TMHMM2.0	inside	   310   364
PbraGR22	TMHMM2.0	TMhelix	   365   387
PbraGR22	TMHMM2.0	outside	   388   388
```

# plot in postscript,
script for making the plot in gnuplot,
data for plot

---

```
# PbraGR7 Length: 374
# PbraGR7 Number of predicted TMHs:  6
# PbraGR7 Exp number of AAs in TMHs: 130.69408
# PbraGR7 Exp number, first 60 AAs:  33.95346
# PbraGR7 Total prob of N-in:        0.88267
# PbraGR7 POSSIBLE N-term signal sequence
PbraGR7	TMHMM2.0	inside	     1    12
PbraGR7	TMHMM2.0	TMhelix	    13    30
PbraGR7	TMHMM2.0	outside	    31    44
PbraGR7	TMHMM2.0	TMhelix	    45    62
PbraGR7	TMHMM2.0	inside	    63    98
PbraGR7	TMHMM2.0	TMhelix	    99   121
PbraGR7	TMHMM2.0	outside	   122   164
PbraGR7	TMHMM2.0	TMhelix	   165   187
PbraGR7	TMHMM2.0	inside	   188   282
PbraGR7	TMHMM2.0	TMhelix	   283   302
PbraGR7	TMHMM2.0	outside	   303   344
PbraGR7	TMHMM2.0	TMhelix	   345   367
PbraGR7	TMHMM2.0	inside	   368   374
```

# plot in postscript,
script for making the plot in gnuplot,
data for plot

---

```
# PbraGR6 Length: 432
# PbraGR6 Number of predicted TMHs:  8
# PbraGR6 Exp number of AAs in TMHs: 173.1601
# PbraGR6 Exp number, first 60 AAs:  18.41128
# PbraGR6 Total prob of N-in:        0.19811
# PbraGR6 POSSIBLE N-term signal sequence
PbraGR6	TMHMM2.0	outside	     1    31
PbraGR6	TMHMM2.0	TMhelix	    32    54
PbraGR6	TMHMM2.0	inside	    55    66
PbraGR6	TMHMM2.0	TMhelix	    67    89
PbraGR6	TMHMM2.0	outside	    90    98
PbraGR6	TMHMM2.0	TMhelix	    99   118
PbraGR6	TMHMM2.0	inside	   119   151
PbraGR6	TMHMM2.0	TMhelix	   152   174
PbraGR6	TMHMM2.0	outside	   175   209
PbraGR6	TMHMM2.0	TMhelix	   210   232
PbraGR6	TMHMM2.0	inside	   233   275
PbraGR6	TMHMM2.0	TMhelix	   276   298
PbraGR6	TMHMM2.0	outside	   299   325
PbraGR6	TMHMM2.0	TMhelix	   326   348
PbraGR6	TMHMM2.0	inside	   349   393
PbraGR6	TMHMM2.0	TMhelix	   394   416
PbraGR6	TMHMM2.0	outside	   417   432
```

# plot in postscript,
script for making the plot in gnuplot,
data for plot

---

```
# PbraGR5 Length: 409
# PbraGR5 Number of predicted TMHs:  7
# PbraGR5 Exp number of AAs in TMHs: 164.76245
# PbraGR5 Exp number, first 60 AAs:  17.93871
# PbraGR5 Total prob of N-in:        0.73316
# PbraGR5 POSSIBLE N-term signal sequence
PbraGR5	TMHMM2.0	inside	     1    48
PbraGR5	TMHMM2.0	TMhelix	    49    71
PbraGR5	TMHMM2.0	outside	    72    80
PbraGR5	TMHMM2.0	TMhelix	    81   103
PbraGR5	TMHMM2.0	inside	   104   123
PbraGR5	TMHMM2.0	TMhelix	   124   146
PbraGR5	TMHMM2.0	outside	   147   173
PbraGR5	TMHMM2.0	TMhelix	   174   196
PbraGR5	TMHMM2.0	inside	   197   259
PbraGR5	TMHMM2.0	TMhelix	   260   282
PbraGR5	TMHMM2.0	outside	   283   308
PbraGR5	TMHMM2.0	TMhelix	   309   331
PbraGR5	TMHMM2.0	inside	   332   379
PbraGR5	TMHMM2.0	TMhelix	   380   402
PbraGR5	TMHMM2.0	outside	   403   409
```

# plot in postscript,
script for making the plot in gnuplot,
data for plot

---

```
# PbraGR52 Length: 437
# PbraGR52 Number of predicted TMHs:  6
# PbraGR52 Exp number of AAs in TMHs: 142.6179
# PbraGR52 Exp number, first 60 AAs:  9.44154
# PbraGR52 Total prob of N-in:        0.89923
PbraGR52	TMHMM2.0	inside	     1    57
PbraGR52	TMHMM2.0	TMhelix	    58    80
PbraGR52	TMHMM2.0	outside	    81    94
PbraGR52	TMHMM2.0	TMhelix	    95   117
PbraGR52	TMHMM2.0	inside	   118   206
PbraGR52	TMHMM2.0	TMhelix	   207   229
PbraGR52	TMHMM2.0	outside	   230   288
PbraGR52	TMHMM2.0	TMhelix	   289   311
PbraGR52	TMHMM2.0	inside	   312   323
PbraGR52	TMHMM2.0	TMhelix	   324   346
PbraGR52	TMHMM2.0	outside	   347   388
PbraGR52	TMHMM2.0	TMhelix	   389   411
PbraGR52	TMHMM2.0	inside	   412   437
```

# plot in postscript,
script for making the plot in gnuplot,
data for plot

---

```
# PbraGR4 Length: 404
# PbraGR4 Number of predicted TMHs:  6
# PbraGR4 Exp number of AAs in TMHs: 128.66619
# PbraGR4 Exp number, first 60 AAs:  21.76641
# PbraGR4 Total prob of N-in:        0.99311
# PbraGR4 POSSIBLE N-term signal sequence
PbraGR4	TMHMM2.0	inside	     1    35
PbraGR4	TMHMM2.0	TMhelix	    36    58
PbraGR4	TMHMM2.0	outside	    59    77
PbraGR4	TMHMM2.0	TMhelix	    78   100
PbraGR4	TMHMM2.0	inside	   101   133
PbraGR4	TMHMM2.0	TMhelix	   134   153
PbraGR4	TMHMM2.0	outside	   154   182
PbraGR4	TMHMM2.0	TMhelix	   183   205
PbraGR4	TMHMM2.0	inside	   206   275
PbraGR4	TMHMM2.0	TMhelix	   276   298
PbraGR4	TMHMM2.0	outside	   299   307
PbraGR4	TMHMM2.0	TMhelix	   308   330
PbraGR4	TMHMM2.0	inside	   331   404
```

# plot in postscript,
script for making the plot in gnuplot,
data for plot

---

```
# PbraGR42 Length: 270
# PbraGR42 Number of predicted TMHs:  4
# PbraGR42 Exp number of AAs in TMHs: 89.61987
# PbraGR42 Exp number, first 60 AAs:  15.92974
# PbraGR42 Total prob of N-in:        0.95940
# PbraGR42 POSSIBLE N-term signal sequence
PbraGR42	TMHMM2.0	inside	     1    44
PbraGR42	TMHMM2.0	TMhelix	    45    67
PbraGR42	TMHMM2.0	outside	    68    76
PbraGR42	TMHMM2.0	TMhelix	    77    99
PbraGR42	TMHMM2.0	inside	   100   132
PbraGR42	TMHMM2.0	TMhelix	   133   155
PbraGR42	TMHMM2.0	outside	   156   164
PbraGR42	TMHMM2.0	TMhelix	   165   187
PbraGR42	TMHMM2.0	inside	   188   270
```

# plot in postscript,
script for making the plot in gnuplot,
data for plot

---

```
# PbraGR44.1 Length: 397
# PbraGR44.1 Number of predicted TMHs:  7
# PbraGR44.1 Exp number of AAs in TMHs: 157.02122
# PbraGR44.1 Exp number, first 60 AAs:  11.55019
# PbraGR44.1 Total prob of N-in:        0.93154
# PbraGR44.1 POSSIBLE N-term signal sequence
PbraGR44.1	TMHMM2.0	inside	     1    50
PbraGR44.1	TMHMM2.0	TMhelix	    51    70
PbraGR44.1	TMHMM2.0	outside	    71    79
PbraGR44.1	TMHMM2.0	TMhelix	    80   102
PbraGR44.1	TMHMM2.0	inside	   103   139
PbraGR44.1	TMHMM2.0	TMhelix	   140   162
PbraGR44.1	TMHMM2.0	outside	   163   171
PbraGR44.1	TMHMM2.0	TMhelix	   172   194
PbraGR44.1	TMHMM2.0	inside	   195   263
PbraGR44.1	TMHMM2.0	TMhelix	   264   286
PbraGR44.1	TMHMM2.0	outside	   287   300
PbraGR44.1	TMHMM2.0	TMhelix	   301   323
PbraGR44.1	TMHMM2.0	inside	   324   373
PbraGR44.1	TMHMM2.0	TMhelix	   374   396
PbraGR44.1	TMHMM2.0	outside	   397   397
```

# plot in postscript,
script for making the plot in gnuplot,
data for plot

---

```
# PbraGR44.2 Length: 396
# PbraGR44.2 Number of predicted TMHs:  7
# PbraGR44.2 Exp number of AAs in TMHs: 154.96903
# PbraGR44.2 Exp number, first 60 AAs:  10.37951
# PbraGR44.2 Total prob of N-in:        0.97359
# PbraGR44.2 POSSIBLE N-term signal sequence
PbraGR44.2	TMHMM2.0	inside	     1    50
PbraGR44.2	TMHMM2.0	TMhelix	    51    73
PbraGR44.2	TMHMM2.0	outside	    74    77
PbraGR44.2	TMHMM2.0	TMhelix	    78   100
PbraGR44.2	TMHMM2.0	inside	   101   136
PbraGR44.2	TMHMM2.0	TMhelix	   137   156
PbraGR44.2	TMHMM2.0	outside	   157   165
PbraGR44.2	TMHMM2.0	TMhelix	   166   188
PbraGR44.2	TMHMM2.0	inside	   189   266
PbraGR44.2	TMHMM2.0	TMhelix	   267   289
PbraGR44.2	TMHMM2.0	outside	   290   298
PbraGR44.2	TMHMM2.0	TMhelix	   299   321
PbraGR44.2	TMHMM2.0	inside	   322   372
PbraGR44.2	TMHMM2.0	TMhelix	   373   395
PbraGR44.2	TMHMM2.0	outside	   396   396
```

# plot in postscript,
script for making the plot in gnuplot,
data for plot

---

```
# PbraGR27 Length: 391
# PbraGR27 Number of predicted TMHs:  8
# PbraGR27 Exp number of AAs in TMHs: 175.85757
# PbraGR27 Exp number, first 60 AAs:  38.31207
# PbraGR27 Total prob of N-in:        0.04001
# PbraGR27 POSSIBLE N-term signal sequence
PbraGR27	TMHMM2.0	outside	     1    14
PbraGR27	TMHMM2.0	TMhelix	    15    37
PbraGR27	TMHMM2.0	inside	    38    43
PbraGR27	TMHMM2.0	TMhelix	    44    63
PbraGR27	TMHMM2.0	outside	    64    72
PbraGR27	TMHMM2.0	TMhelix	    73    95
PbraGR27	TMHMM2.0	inside	    96   125
PbraGR27	TMHMM2.0	TMhelix	   126   148
PbraGR27	TMHMM2.0	outside	   149   162
PbraGR27	TMHMM2.0	TMhelix	   163   185
PbraGR27	TMHMM2.0	inside	   186   248
PbraGR27	TMHMM2.0	TMhelix	   249   271
PbraGR27	TMHMM2.0	outside	   272   290
PbraGR27	TMHMM2.0	TMhelix	   291   313
PbraGR27	TMHMM2.0	inside	   314   367
PbraGR27	TMHMM2.0	TMhelix	   368   390
PbraGR27	TMHMM2.0	outside	   391   391
```

# plot in postscript,
script for making the plot in gnuplot,
data for plot

---

```
# PbraGR18 Length: 346
# PbraGR18 Number of predicted TMHs:  7
# PbraGR18 Exp number of AAs in TMHs: 142.52341
# PbraGR18 Exp number, first 60 AAs:  25.67412
# PbraGR18 Total prob of N-in:        0.80492
# PbraGR18 POSSIBLE N-term signal sequence
PbraGR18	TMHMM2.0	inside	     1    23
PbraGR18	TMHMM2.0	TMhelix	    24    46
PbraGR18	TMHMM2.0	outside	    47    60
PbraGR18	TMHMM2.0	TMhelix	    61    83
PbraGR18	TMHMM2.0	inside	    84   113
PbraGR18	TMHMM2.0	TMhelix	   114   133
PbraGR18	TMHMM2.0	outside	   134   147
PbraGR18	TMHMM2.0	TMhelix	   148   170
PbraGR18	TMHMM2.0	inside	   171   220
PbraGR18	TMHMM2.0	TMhelix	   221   240
PbraGR18	TMHMM2.0	outside	   241   254
PbraGR18	TMHMM2.0	TMhelix	   255   277
PbraGR18	TMHMM2.0	inside	   278   324
PbraGR18	TMHMM2.0	TMhelix	   325   344
PbraGR18	TMHMM2.0	outside	   345   346
```

# plot in postscript,
script for making the plot in gnuplot,
data for plot

---

```
# PbraGR61 Length: 340
# PbraGR61 Number of predicted TMHs:  6
# PbraGR61 Exp number of AAs in TMHs: 126.14875
# PbraGR61 Exp number, first 60 AAs:  23.80828
# PbraGR61 Total prob of N-in:        0.69283
# PbraGR61 POSSIBLE N-term signal sequence
PbraGR61	TMHMM2.0	inside	     1    33
PbraGR61	TMHMM2.0	TMhelix	    34    51
PbraGR61	TMHMM2.0	outside	    52    60
PbraGR61	TMHMM2.0	TMhelix	    61    78
PbraGR61	TMHMM2.0	inside	    79   106
PbraGR61	TMHMM2.0	TMhelix	   107   129
PbraGR61	TMHMM2.0	outside	   130   212
PbraGR61	TMHMM2.0	TMhelix	   213   235
PbraGR61	TMHMM2.0	inside	   236   241
PbraGR61	TMHMM2.0	TMhelix	   242   264
PbraGR61	TMHMM2.0	outside	   265   320
PbraGR61	TMHMM2.0	TMhelix	   321   338
PbraGR61	TMHMM2.0	inside	   339   340
```

# plot in postscript,
script for making the plot in gnuplot,
data for plot

---

```
# PbraGR65 Length: 351
# PbraGR65 Number of predicted TMHs:  4
# PbraGR65 Exp number of AAs in TMHs: 120.54519
# PbraGR65 Exp number, first 60 AAs:  22.91957
# PbraGR65 Total prob of N-in:        0.70333
# PbraGR65 POSSIBLE N-term signal sequence
PbraGR65	TMHMM2.0	outside	     1    30
PbraGR65	TMHMM2.0	TMhelix	    31    53
PbraGR65	TMHMM2.0	inside	    54   114
PbraGR65	TMHMM2.0	TMhelix	   115   137
PbraGR65	TMHMM2.0	outside	   138   249
PbraGR65	TMHMM2.0	TMhelix	   250   272
PbraGR65	TMHMM2.0	inside	   273   320
PbraGR65	TMHMM2.0	TMhelix	   321   343
PbraGR65	TMHMM2.0	outside	   344   351
```

# plot in postscript,
script for making the plot in gnuplot,
data for plot

---

```
# PbraGR63 Length: 400
# PbraGR63 Number of predicted TMHs:  6
# PbraGR63 Exp number of AAs in TMHs: 138.48097
# PbraGR63 Exp number, first 60 AAs:  20.03919
# PbraGR63 Total prob of N-in:        0.72567
# PbraGR63 POSSIBLE N-term signal sequence
PbraGR63	TMHMM2.0	outside	     1    41
PbraGR63	TMHMM2.0	TMhelix	    42    64
PbraGR63	TMHMM2.0	inside	    65   135
PbraGR63	TMHMM2.0	TMhelix	   136   155
PbraGR63	TMHMM2.0	outside	   156   164
PbraGR63	TMHMM2.0	TMhelix	   165   187
PbraGR63	TMHMM2.0	inside	   188   265
PbraGR63	TMHMM2.0	TMhelix	   266   288
PbraGR63	TMHMM2.0	outside	   289   297
PbraGR63	TMHMM2.0	TMhelix	   298   320
PbraGR63	TMHMM2.0	inside	   321   377
PbraGR63	TMHMM2.0	TMhelix	   378   397
PbraGR63	TMHMM2.0	outside	   398   400
```

# plot in postscript,
script for making the plot in gnuplot,
data for plot

---

```
# PbraGR12 Length: 345
# PbraGR12 Number of predicted TMHs:  7
# PbraGR12 Exp number of AAs in TMHs: 155.77084
# PbraGR12 Exp number, first 60 AAs:  30.41626
# PbraGR12 Total prob of N-in:        0.60388
# PbraGR12 POSSIBLE N-term signal sequence
PbraGR12	TMHMM2.0	inside	     1    24
PbraGR12	TMHMM2.0	TMhelix	    25    47
PbraGR12	TMHMM2.0	outside	    48    61
PbraGR12	TMHMM2.0	TMhelix	    62    81
PbraGR12	TMHMM2.0	inside	    82   101
PbraGR12	TMHMM2.0	TMhelix	   102   124
PbraGR12	TMHMM2.0	outside	   125   138
PbraGR12	TMHMM2.0	TMhelix	   139   161
PbraGR12	TMHMM2.0	inside	   162   212
PbraGR12	TMHMM2.0	TMhelix	   213   235
PbraGR12	TMHMM2.0	outside	   236   244
PbraGR12	TMHMM2.0	TMhelix	   245   267
PbraGR12	TMHMM2.0	inside	   268   315
PbraGR12	TMHMM2.0	TMhelix	   316   338
PbraGR12	TMHMM2.0	outside	   339   345
```

# plot in postscript,
script for making the plot in gnuplot,
data for plot

---

```
# PbraGR15 Length: 349
# PbraGR15 Number of predicted TMHs:  7
# PbraGR15 Exp number of AAs in TMHs: 165.99498
# PbraGR15 Exp number, first 60 AAs:  43.46586
# PbraGR15 Total prob of N-in:        0.09257
# PbraGR15 POSSIBLE N-term signal sequence
PbraGR15	TMHMM2.0	outside	     1     3
PbraGR15	TMHMM2.0	TMhelix	     4    26
PbraGR15	TMHMM2.0	inside	    27    32
PbraGR15	TMHMM2.0	TMhelix	    33    55
PbraGR15	TMHMM2.0	outside	    56    69
PbraGR15	TMHMM2.0	TMhelix	    70    92
PbraGR15	TMHMM2.0	inside	    93   112
PbraGR15	TMHMM2.0	TMhelix	   113   132
PbraGR15	TMHMM2.0	outside	   133   221
PbraGR15	TMHMM2.0	TMhelix	   222   244
PbraGR15	TMHMM2.0	inside	   245   250
PbraGR15	TMHMM2.0	TMhelix	   251   273
PbraGR15	TMHMM2.0	outside	   274   318
PbraGR15	TMHMM2.0	TMhelix	   319   341
PbraGR15	TMHMM2.0	inside	   342   349
```

# plot in postscript,
script for making the plot in gnuplot,
data for plot

---

```
# PbraGR17 Length: 222
# PbraGR17 Number of predicted TMHs:  1
# PbraGR17 Exp number of AAs in TMHs: 57.14978
# PbraGR17 Exp number, first 60 AAs:  15.29528
# PbraGR17 Total prob of N-in:        0.34102
# PbraGR17 POSSIBLE N-term signal sequence
PbraGR17	TMHMM2.0	outside	     1   197
PbraGR17	TMHMM2.0	TMhelix	   198   220
PbraGR17	TMHMM2.0	inside	   221   222
```

# plot in postscript,
script for making the plot in gnuplot,
data for plot

---

```
# PbraGR13 Length: 356
# PbraGR13 Number of predicted TMHs:  7
# PbraGR13 Exp number of AAs in TMHs: 153.42128
# PbraGR13 Exp number, first 60 AAs:  20.41571
# PbraGR13 Total prob of N-in:        0.98374
# PbraGR13 POSSIBLE N-term signal sequence
PbraGR13	TMHMM2.0	inside	     1    31
PbraGR13	TMHMM2.0	TMhelix	    32    49
PbraGR13	TMHMM2.0	outside	    50    63
PbraGR13	TMHMM2.0	TMhelix	    64    86
PbraGR13	TMHMM2.0	inside	    87   106
PbraGR13	TMHMM2.0	TMhelix	   107   129
PbraGR13	TMHMM2.0	outside	   130   148
PbraGR13	TMHMM2.0	TMhelix	   149   171
PbraGR13	TMHMM2.0	inside	   172   227
PbraGR13	TMHMM2.0	TMhelix	   228   250
PbraGR13	TMHMM2.0	outside	   251   254
PbraGR13	TMHMM2.0	TMhelix	   255   277
PbraGR13	TMHMM2.0	inside	   278   325
PbraGR13	TMHMM2.0	TMhelix	   326   348
PbraGR13	TMHMM2.0	outside	   349   356
```

# plot in postscript,
script for making the plot in gnuplot,
data for plot

---

```
# PbraGR31 Length: 338
# PbraGR31 Number of predicted TMHs:  7
# PbraGR31 Exp number of AAs in TMHs: 151.25109
# PbraGR31 Exp number, first 60 AAs:  25.45394
# PbraGR31 Total prob of N-in:        0.99017
# PbraGR31 POSSIBLE N-term signal sequence
PbraGR31	TMHMM2.0	inside	     1    30
PbraGR31	TMHMM2.0	TMhelix	    31    53
PbraGR31	TMHMM2.0	outside	    54    56
PbraGR31	TMHMM2.0	TMhelix	    57    74
PbraGR31	TMHMM2.0	inside	    75    97
PbraGR31	TMHMM2.0	TMhelix	    98   117
PbraGR31	TMHMM2.0	outside	   118   126
PbraGR31	TMHMM2.0	TMhelix	   127   149
PbraGR31	TMHMM2.0	inside	   150   203
PbraGR31	TMHMM2.0	TMhelix	   204   226
PbraGR31	TMHMM2.0	outside	   227   240
PbraGR31	TMHMM2.0	TMhelix	   241   263
PbraGR31	TMHMM2.0	inside	   264   316
PbraGR31	TMHMM2.0	TMhelix	   317   336
PbraGR31	TMHMM2.0	outside	   337   338
```

# plot in postscript,
script for making the plot in gnuplot,
data for plot

---

```
# PbraGR64 Length: 283
# PbraGR64 Number of predicted TMHs:  7
# PbraGR64 Exp number of AAs in TMHs: 146.11561
# PbraGR64 Exp number, first 60 AAs:  35.84945
# PbraGR64 Total prob of N-in:        0.23435
# PbraGR64 POSSIBLE N-term signal sequence
PbraGR64	TMHMM2.0	outside	     1     3
PbraGR64	TMHMM2.0	TMhelix	     4    21
PbraGR64	TMHMM2.0	inside	    22    33
PbraGR64	TMHMM2.0	TMhelix	    34    56
PbraGR64	TMHMM2.0	outside	    57    60
PbraGR64	TMHMM2.0	TMhelix	    61    83
PbraGR64	TMHMM2.0	inside	    84   107
PbraGR64	TMHMM2.0	TMhelix	   108   130
PbraGR64	TMHMM2.0	outside	   131   144
PbraGR64	TMHMM2.0	TMhelix	   145   167
PbraGR64	TMHMM2.0	inside	   168   224
PbraGR64	TMHMM2.0	TMhelix	   225   247
PbraGR64	TMHMM2.0	outside	   248   251
PbraGR64	TMHMM2.0	TMhelix	   252   274
PbraGR64	TMHMM2.0	inside	   275   283
```

# plot in postscript,
script for making the plot in gnuplot,
data for plot

---

```
# PbraGR14 Length: 204
# PbraGR14 Number of predicted TMHs:  3
# PbraGR14 Exp number of AAs in TMHs: 65.1122
# PbraGR14 Exp number, first 60 AAs:  9.55268
# PbraGR14 Total prob of N-in:        0.61174
PbraGR14	TMHMM2.0	inside	     1     6
PbraGR14	TMHMM2.0	TMhelix	     7    24
PbraGR14	TMHMM2.0	outside	    25    74
PbraGR14	TMHMM2.0	TMhelix	    75    97
PbraGR14	TMHMM2.0	inside	    98   180
PbraGR14	TMHMM2.0	TMhelix	   181   203
PbraGR14	TMHMM2.0	outside	   204   204
```

# plot in postscript,
script for making the plot in gnuplot,
data for plot

---

```
# PrapGR54 Length: 361
# PrapGR54 Number of predicted TMHs:  5
# PrapGR54 Exp number of AAs in TMHs: 146.82164
# PrapGR54 Exp number, first 60 AAs:  38.15143
# PrapGR54 Total prob of N-in:        0.96255
# PrapGR54 POSSIBLE N-term signal sequence
PrapGR54	TMHMM2.0	inside	     1    11
PrapGR54	TMHMM2.0	TMhelix	    12    29
PrapGR54	TMHMM2.0	outside	    30    43
PrapGR54	TMHMM2.0	TMhelix	    44    66
PrapGR54	TMHMM2.0	inside	    67   111
PrapGR54	TMHMM2.0	TMhelix	   112   134
PrapGR54	TMHMM2.0	outside	   135   143
PrapGR54	TMHMM2.0	TMhelix	   144   166
PrapGR54	TMHMM2.0	inside	   167   332
PrapGR54	TMHMM2.0	TMhelix	   333   355
PrapGR54	TMHMM2.0	outside	   356   361
```

# plot in postscript,
script for making the plot in gnuplot,
data for plot

---

```
# PrapGR29 Length: 347
# PrapGR29 Number of predicted TMHs:  8
# PrapGR29 Exp number of AAs in TMHs: 165.31598
# PrapGR29 Exp number, first 60 AAs:  34.60003
# PrapGR29 Total prob of N-in:        0.22982
# PrapGR29 POSSIBLE N-term signal sequence
PrapGR29	TMHMM2.0	outside	     1     9
PrapGR29	TMHMM2.0	TMhelix	    10    32
PrapGR29	TMHMM2.0	inside	    33    38
PrapGR29	TMHMM2.0	TMhelix	    39    56
PrapGR29	TMHMM2.0	outside	    57    60
PrapGR29	TMHMM2.0	TMhelix	    61    80
PrapGR29	TMHMM2.0	inside	    81   100
PrapGR29	TMHMM2.0	TMhelix	   101   120
PrapGR29	TMHMM2.0	outside	   121   129
PrapGR29	TMHMM2.0	TMhelix	   130   152
PrapGR29	TMHMM2.0	inside	   153   210
PrapGR29	TMHMM2.0	TMhelix	   211   233
PrapGR29	TMHMM2.0	outside	   234   242
PrapGR29	TMHMM2.0	TMhelix	   243   265
PrapGR29	TMHMM2.0	inside	   266   312
PrapGR29	TMHMM2.0	TMhelix	   313   335
PrapGR29	TMHMM2.0	outside	   336   347
```

# plot in postscript,
script for making the plot in gnuplot,
data for plot

---

```
# PrapGR30 Length: 352
# PrapGR30 Number of predicted TMHs:  7
# PrapGR30 Exp number of AAs in TMHs: 150.05657
# PrapGR30 Exp number, first 60 AAs:  23.07601
# PrapGR30 Total prob of N-in:        0.67077
# PrapGR30 POSSIBLE N-term signal sequence
PrapGR30	TMHMM2.0	inside	     1    30
PrapGR30	TMHMM2.0	TMhelix	    31    50
PrapGR30	TMHMM2.0	outside	    51    64
PrapGR30	TMHMM2.0	TMhelix	    65    87
PrapGR30	TMHMM2.0	inside	    88    99
PrapGR30	TMHMM2.0	TMhelix	   100   122
PrapGR30	TMHMM2.0	outside	   123   136
PrapGR30	TMHMM2.0	TMhelix	   137   159
PrapGR30	TMHMM2.0	inside	   160   213
PrapGR30	TMHMM2.0	TMhelix	   214   236
PrapGR30	TMHMM2.0	outside	   237   250
PrapGR30	TMHMM2.0	TMhelix	   251   273
PrapGR30	TMHMM2.0	inside	   274   315
PrapGR30	TMHMM2.0	TMhelix	   316   338
PrapGR30	TMHMM2.0	outside	   339   352
```

# plot in postscript,
script for making the plot in gnuplot,
data for plot

---

```
# PrapGR8.1 Length: 374
# PrapGR8.1 Number of predicted TMHs:  5
# PrapGR8.1 Exp number of AAs in TMHs: 132.37665
# PrapGR8.1 Exp number, first 60 AAs:  18.48532
# PrapGR8.1 Total prob of N-in:        0.81994
# PrapGR8.1 POSSIBLE N-term signal sequence
PrapGR8.1	TMHMM2.0	inside	     1    45
PrapGR8.1	TMHMM2.0	TMhelix	    46    68
PrapGR8.1	TMHMM2.0	outside	    69    77
PrapGR8.1	TMHMM2.0	TMhelix	    78   100
PrapGR8.1	TMHMM2.0	inside	   101   126
PrapGR8.1	TMHMM2.0	TMhelix	   127   149
PrapGR8.1	TMHMM2.0	outside	   150   158
PrapGR8.1	TMHMM2.0	TMhelix	   159   178
PrapGR8.1	TMHMM2.0	inside	   179   353
PrapGR8.1	TMHMM2.0	TMhelix	   354   373
PrapGR8.1	TMHMM2.0	outside	   374   374
```

# plot in postscript,
script for making the plot in gnuplot,
data for plot

---

```
# PrapGR8.2 Length: 384
# PrapGR8.2 Number of predicted TMHs:  6
# PrapGR8.2 Exp number of AAs in TMHs: 129.85385
# PrapGR8.2 Exp number, first 60 AAs:  26.55233
# PrapGR8.2 Total prob of N-in:        0.75254
# PrapGR8.2 POSSIBLE N-term signal sequence
PrapGR8.2	TMHMM2.0	inside	     1    43
PrapGR8.2	TMHMM2.0	TMhelix	    44    66
PrapGR8.2	TMHMM2.0	outside	    67    75
PrapGR8.2	TMHMM2.0	TMhelix	    76    98
PrapGR8.2	TMHMM2.0	inside	    99   124
PrapGR8.2	TMHMM2.0	TMhelix	   125   147
PrapGR8.2	TMHMM2.0	outside	   148   212
PrapGR8.2	TMHMM2.0	TMhelix	   213   232
PrapGR8.2	TMHMM2.0	inside	   233   238
PrapGR8.2	TMHMM2.0	TMhelix	   239   261
PrapGR8.2	TMHMM2.0	outside	   262   354
PrapGR8.2	TMHMM2.0	TMhelix	   355   377
PrapGR8.2	TMHMM2.0	inside	   378   384
```

# plot in postscript,
script for making the plot in gnuplot,
data for plot

---

```
# PrapGR8.3 Length: 379
# PrapGR8.3 Number of predicted TMHs:  6
# PrapGR8.3 Exp number of AAs in TMHs: 121.73224
# PrapGR8.3 Exp number, first 60 AAs:  26.44879
# PrapGR8.3 Total prob of N-in:        0.24733
# PrapGR8.3 POSSIBLE N-term signal sequence
PrapGR8.3	TMHMM2.0	outside	     1     3
PrapGR8.3	TMHMM2.0	TMhelix	     4    23
PrapGR8.3	TMHMM2.0	inside	    24    43
PrapGR8.3	TMHMM2.0	TMhelix	    44    66
PrapGR8.3	TMHMM2.0	outside	    67    75
PrapGR8.3	TMHMM2.0	TMhelix	    76    98
PrapGR8.3	TMHMM2.0	inside	    99   123
PrapGR8.3	TMHMM2.0	TMhelix	   124   146
PrapGR8.3	TMHMM2.0	outside	   147   239
PrapGR8.3	TMHMM2.0	TMhelix	   240   262
PrapGR8.3	TMHMM2.0	inside	   263   354
PrapGR8.3	TMHMM2.0	TMhelix	   355   374
PrapGR8.3	TMHMM2.0	outside	   375   379
```

# plot in postscript,
script for making the plot in gnuplot,
data for plot

---

```
# PrapGR28 Length: 383
# PrapGR28 Number of predicted TMHs:  7
# PrapGR28 Exp number of AAs in TMHs: 150.01111
# PrapGR28 Exp number, first 60 AAs:  27.43222
# PrapGR28 Total prob of N-in:        0.27700
# PrapGR28 POSSIBLE N-term signal sequence
PrapGR28	TMHMM2.0	outside	     1    21
PrapGR28	TMHMM2.0	TMhelix	    22    44
PrapGR28	TMHMM2.0	inside	    45    50
PrapGR28	TMHMM2.0	TMhelix	    51    73
PrapGR28	TMHMM2.0	outside	    74    76
PrapGR28	TMHMM2.0	TMhelix	    77    95
PrapGR28	TMHMM2.0	inside	    96   129
PrapGR28	TMHMM2.0	TMhelix	   130   152
PrapGR28	TMHMM2.0	outside	   153   161
PrapGR28	TMHMM2.0	TMhelix	   162   179
PrapGR28	TMHMM2.0	inside	   180   237
PrapGR28	TMHMM2.0	TMhelix	   238   260
PrapGR28	TMHMM2.0	outside	   261   345
PrapGR28	TMHMM2.0	TMhelix	   346   368
PrapGR28	TMHMM2.0	inside	   369   383
```

# plot in postscript,
script for making the plot in gnuplot,
data for plot

---

```
# PrapGR1 Length: 459
# PrapGR1 Number of predicted TMHs:  8
# PrapGR1 Exp number of AAs in TMHs: 164.39937
# PrapGR1 Exp number, first 60 AAs:  2.11657
# PrapGR1 Total prob of N-in:        0.34065
PrapGR1	TMHMM2.0	outside	     1    57
PrapGR1	TMHMM2.0	TMhelix	    58    80
PrapGR1	TMHMM2.0	inside	    81    91
PrapGR1	TMHMM2.0	TMhelix	    92   114
PrapGR1	TMHMM2.0	outside	   115   133
PrapGR1	TMHMM2.0	TMhelix	   134   156
PrapGR1	TMHMM2.0	inside	   157   187
PrapGR1	TMHMM2.0	TMhelix	   188   210
PrapGR1	TMHMM2.0	outside	   211   219
PrapGR1	TMHMM2.0	TMhelix	   220   242
PrapGR1	TMHMM2.0	inside	   243   290
PrapGR1	TMHMM2.0	TMhelix	   291   313
PrapGR1	TMHMM2.0	outside	   314   327
PrapGR1	TMHMM2.0	TMhelix	   328   345
PrapGR1	TMHMM2.0	inside	   346   404
PrapGR1	TMHMM2.0	TMhelix	   405   427
PrapGR1	TMHMM2.0	outside	   428   459
```

# plot in postscript,
script for making the plot in gnuplot,
data for plot

---

```
# PrapGR2 Length: 430
# PrapGR2 Number of predicted TMHs:  7
# PrapGR2 Exp number of AAs in TMHs: 145.40975
# PrapGR2 Exp number, first 60 AAs:  0.01515
# PrapGR2 Total prob of N-in:        0.78963
PrapGR2	TMHMM2.0	inside	     1    97
PrapGR2	TMHMM2.0	TMhelix	    98   120
PrapGR2	TMHMM2.0	outside	   121   129
PrapGR2	TMHMM2.0	TMhelix	   130   152
PrapGR2	TMHMM2.0	inside	   153   186
PrapGR2	TMHMM2.0	TMhelix	   187   209
PrapGR2	TMHMM2.0	outside	   210   218
PrapGR2	TMHMM2.0	TMhelix	   219   236
PrapGR2	TMHMM2.0	inside	   237   289
PrapGR2	TMHMM2.0	TMhelix	   290   309
PrapGR2	TMHMM2.0	outside	   310   323
PrapGR2	TMHMM2.0	TMhelix	   324   341
PrapGR2	TMHMM2.0	inside	   342   400
PrapGR2	TMHMM2.0	TMhelix	   401   423
PrapGR2	TMHMM2.0	outside	   424   430
```

# plot in postscript,
script for making the plot in gnuplot,
data for plot

---

```
# PrapGR3 Length: 475
# PrapGR3 Number of predicted TMHs:  6
# PrapGR3 Exp number of AAs in TMHs: 157.64692
# PrapGR3 Exp number, first 60 AAs:  0.00027
# PrapGR3 Total prob of N-in:        0.96575
PrapGR3	TMHMM2.0	inside	     1    96
PrapGR3	TMHMM2.0	TMhelix	    97   119
PrapGR3	TMHMM2.0	outside	   120   138
PrapGR3	TMHMM2.0	TMhelix	   139   161
PrapGR3	TMHMM2.0	inside	   162   195
PrapGR3	TMHMM2.0	TMhelix	   196   218
PrapGR3	TMHMM2.0	outside	   219   222
PrapGR3	TMHMM2.0	TMhelix	   223   245
PrapGR3	TMHMM2.0	inside	   246   291
PrapGR3	TMHMM2.0	TMhelix	   292   314
PrapGR3	TMHMM2.0	outside	   315   328
PrapGR3	TMHMM2.0	TMhelix	   329   346
PrapGR3	TMHMM2.0	inside	   347   475
```

# plot in postscript,
script for making the plot in gnuplot,
data for plot

---

```
# PrapGR8.4 Length: 372
# PrapGR8.4 Number of predicted TMHs:  4
# PrapGR8.4 Exp number of AAs in TMHs: 111.71714
# PrapGR8.4 Exp number, first 60 AAs:  36.48404
# PrapGR8.4 Total prob of N-in:        0.92168
# PrapGR8.4 POSSIBLE N-term signal sequence
PrapGR8.4	TMHMM2.0	inside	     1    12
PrapGR8.4	TMHMM2.0	TMhelix	    13    30
PrapGR8.4	TMHMM2.0	outside	    31    44
PrapGR8.4	TMHMM2.0	TMhelix	    45    67
PrapGR8.4	TMHMM2.0	inside	    68    96
PrapGR8.4	TMHMM2.0	TMhelix	    97   119
PrapGR8.4	TMHMM2.0	outside	   120   138
PrapGR8.4	TMHMM2.0	TMhelix	   139   161
PrapGR8.4	TMHMM2.0	inside	   162   372
```

# plot in postscript,
script for making the plot in gnuplot,
data for plot

---

```
# PrapGR9 Length: 454
# PrapGR9 Number of predicted TMHs:  7
# PrapGR9 Exp number of AAs in TMHs: 147.83671
# PrapGR9 Exp number, first 60 AAs:  1.1753
# PrapGR9 Total prob of N-in:        0.75306
PrapGR9	TMHMM2.0	inside	     1    63
PrapGR9	TMHMM2.0	TMhelix	    64    86
PrapGR9	TMHMM2.0	outside	    87   100
PrapGR9	TMHMM2.0	TMhelix	   101   123
PrapGR9	TMHMM2.0	inside	   124   159
PrapGR9	TMHMM2.0	TMhelix	   160   182
PrapGR9	TMHMM2.0	outside	   183   196
PrapGR9	TMHMM2.0	TMhelix	   197   219
PrapGR9	TMHMM2.0	inside	   220   315
PrapGR9	TMHMM2.0	TMhelix	   316   338
PrapGR9	TMHMM2.0	outside	   339   347
PrapGR9	TMHMM2.0	TMhelix	   348   367
PrapGR9	TMHMM2.0	inside	   368   426
PrapGR9	TMHMM2.0	TMhelix	   427   449
PrapGR9	TMHMM2.0	outside	   450   454
```

# plot in postscript,
script for making the plot in gnuplot,
data for plot

---

```
# PrapGR16 Length: 420
# PrapGR16 Number of predicted TMHs:  6
# PrapGR16 Exp number of AAs in TMHs: 131.38444
# PrapGR16 Exp number, first 60 AAs:  22.89178
# PrapGR16 Total prob of N-in:        0.79303
# PrapGR16 POSSIBLE N-term signal sequence
PrapGR16	TMHMM2.0	inside	     1    41
PrapGR16	TMHMM2.0	TMhelix	    42    64
PrapGR16	TMHMM2.0	outside	    65   131
PrapGR16	TMHMM2.0	TMhelix	   132   153
PrapGR16	TMHMM2.0	inside	   154   172
PrapGR16	TMHMM2.0	TMhelix	   173   195
PrapGR16	TMHMM2.0	outside	   196   284
PrapGR16	TMHMM2.0	TMhelix	   285   306
PrapGR16	TMHMM2.0	inside	   307   318
PrapGR16	TMHMM2.0	TMhelix	   319   341
PrapGR16	TMHMM2.0	outside	   342   399
PrapGR16	TMHMM2.0	TMhelix	   400   419
PrapGR16	TMHMM2.0	inside	   420   420
```

# plot in postscript,
script for making the plot in gnuplot,
data for plot

---

```
# PrapGR46 Length: 266
# PrapGR46 Number of predicted TMHs:  5
# PrapGR46 Exp number of AAs in TMHs: 106.98228
# PrapGR46 Exp number, first 60 AAs:  21.57531
# PrapGR46 Total prob of N-in:        0.99606
# PrapGR46 POSSIBLE N-term signal sequence
PrapGR46	TMHMM2.0	inside	     1    31
PrapGR46	TMHMM2.0	TMhelix	    32    54
PrapGR46	TMHMM2.0	outside	    55    63
PrapGR46	TMHMM2.0	TMhelix	    64    81
PrapGR46	TMHMM2.0	inside	    82   115
PrapGR46	TMHMM2.0	TMhelix	   116   138
PrapGR46	TMHMM2.0	outside	   139   161
PrapGR46	TMHMM2.0	TMhelix	   162   184
PrapGR46	TMHMM2.0	inside	   185   227
PrapGR46	TMHMM2.0	TMhelix	   228   250
PrapGR46	TMHMM2.0	outside	   251   266
```

# plot in postscript,
script for making the plot in gnuplot,
data for plot

---

```
# PrapGR10 Length: 427
# PrapGR10 Number of predicted TMHs:  5
# PrapGR10 Exp number of AAs in TMHs: 126.81091
# PrapGR10 Exp number, first 60 AAs:  17.79756
# PrapGR10 Total prob of N-in:        0.99716
# PrapGR10 POSSIBLE N-term signal sequence
PrapGR10	TMHMM2.0	inside	     1    42
PrapGR10	TMHMM2.0	TMhelix	    43    65
PrapGR10	TMHMM2.0	outside	    66    79
PrapGR10	TMHMM2.0	TMhelix	    80   102
PrapGR10	TMHMM2.0	inside	   103   130
PrapGR10	TMHMM2.0	TMhelix	   131   153
PrapGR10	TMHMM2.0	outside	   154   178
PrapGR10	TMHMM2.0	TMhelix	   179   201
PrapGR10	TMHMM2.0	inside	   202   273
PrapGR10	TMHMM2.0	TMhelix	   274   296
PrapGR10	TMHMM2.0	outside	   297   427
```

# plot in postscript,
script for making the plot in gnuplot,
data for plot

---

```
# PrapGR66 Length: 377
# PrapGR66 Number of predicted TMHs:  5
# PrapGR66 Exp number of AAs in TMHs: 118.76713
# PrapGR66 Exp number, first 60 AAs:  22.31928
# PrapGR66 Total prob of N-in:        0.35534
# PrapGR66 POSSIBLE N-term signal sequence
PrapGR66	TMHMM2.0	outside	     1    19
PrapGR66	TMHMM2.0	TMhelix	    20    42
PrapGR66	TMHMM2.0	inside	    43   121
PrapGR66	TMHMM2.0	TMhelix	   122   144
PrapGR66	TMHMM2.0	outside	   145   158
PrapGR66	TMHMM2.0	TMhelix	   159   181
PrapGR66	TMHMM2.0	inside	   182   245
PrapGR66	TMHMM2.0	TMhelix	   246   268
PrapGR66	TMHMM2.0	outside	   269   282
PrapGR66	TMHMM2.0	TMhelix	   283   305
PrapGR66	TMHMM2.0	inside	   306   377
```

# plot in postscript,
script for making the plot in gnuplot,
data for plot

---

```
# PrapGR68.1 Length: 384
# PrapGR68.1 Number of predicted TMHs:  6
# PrapGR68.1 Exp number of AAs in TMHs: 153.85686
# PrapGR68.1 Exp number, first 60 AAs:  23.10635
# PrapGR68.1 Total prob of N-in:        0.08770
# PrapGR68.1 POSSIBLE N-term signal sequence
PrapGR68.1	TMHMM2.0	outside	     1    19
PrapGR68.1	TMHMM2.0	TMhelix	    20    39
PrapGR68.1	TMHMM2.0	inside	    40    59
PrapGR68.1	TMHMM2.0	TMhelix	    60    82
PrapGR68.1	TMHMM2.0	outside	    83    96
PrapGR68.1	TMHMM2.0	TMhelix	    97   116
PrapGR68.1	TMHMM2.0	inside	   117   146
PrapGR68.1	TMHMM2.0	TMhelix	   147   169
PrapGR68.1	TMHMM2.0	outside	   170   178
PrapGR68.1	TMHMM2.0	TMhelix	   179   201
PrapGR68.1	TMHMM2.0	inside	   202   357
PrapGR68.1	TMHMM2.0	TMhelix	   358   380
PrapGR68.1	TMHMM2.0	outside	   381   384
```

# plot in postscript,
script for making the plot in gnuplot,
data for plot

---

```
# PrapGR68.2 Length: 402
# PrapGR68.2 Number of predicted TMHs:  6
# PrapGR68.2 Exp number of AAs in TMHs: 140.96831
# PrapGR68.2 Exp number, first 60 AAs:  8.19321
# PrapGR68.2 Total prob of N-in:        0.58668
PrapGR68.2	TMHMM2.0	inside	     1    70
PrapGR68.2	TMHMM2.0	TMhelix	    71    93
PrapGR68.2	TMHMM2.0	outside	    94   107
PrapGR68.2	TMHMM2.0	TMhelix	   108   130
PrapGR68.2	TMHMM2.0	inside	   131   155
PrapGR68.2	TMHMM2.0	TMhelix	   156   178
PrapGR68.2	TMHMM2.0	outside	   179   197
PrapGR68.2	TMHMM2.0	TMhelix	   198   220
PrapGR68.2	TMHMM2.0	inside	   221   271
PrapGR68.2	TMHMM2.0	TMhelix	   272   294
PrapGR68.2	TMHMM2.0	outside	   295   374
PrapGR68.2	TMHMM2.0	TMhelix	   375   397
PrapGR68.2	TMHMM2.0	inside	   398   402
```

# plot in postscript,
script for making the plot in gnuplot,
data for plot

---

```
# PrapGR22 Length: 388
# PrapGR22 Number of predicted TMHs:  7
# PrapGR22 Exp number of AAs in TMHs: 157.21423
# PrapGR22 Exp number, first 60 AAs:  42.35482
# PrapGR22 Total prob of N-in:        0.92837
# PrapGR22 POSSIBLE N-term signal sequence
PrapGR22	TMHMM2.0	inside	     1     1
PrapGR22	TMHMM2.0	TMhelix	     2    24
PrapGR22	TMHMM2.0	outside	    25    33
PrapGR22	TMHMM2.0	TMhelix	    34    53
PrapGR22	TMHMM2.0	inside	    54   104
PrapGR22	TMHMM2.0	TMhelix	   105   127
PrapGR22	TMHMM2.0	outside	   128   163
PrapGR22	TMHMM2.0	TMhelix	   164   186
PrapGR22	TMHMM2.0	inside	   187   254
PrapGR22	TMHMM2.0	TMhelix	   255   277
PrapGR22	TMHMM2.0	outside	   278   286
PrapGR22	TMHMM2.0	TMhelix	   287   309
PrapGR22	TMHMM2.0	inside	   310   364
PrapGR22	TMHMM2.0	TMhelix	   365   387
PrapGR22	TMHMM2.0	outside	   388   388
```

# plot in postscript,
script for making the plot in gnuplot,
data for plot

---

```
# PrapGR7 Length: 373
# PrapGR7 Number of predicted TMHs:  6
# PrapGR7 Exp number of AAs in TMHs: 136.07939
# PrapGR7 Exp number, first 60 AAs:  29.57849
# PrapGR7 Total prob of N-in:        0.88739
# PrapGR7 POSSIBLE N-term signal sequence
PrapGR7	TMHMM2.0	inside	     1     8
PrapGR7	TMHMM2.0	TMhelix	     9    31
PrapGR7	TMHMM2.0	outside	    32    99
PrapGR7	TMHMM2.0	TMhelix	   100   122
PrapGR7	TMHMM2.0	inside	   123   142
PrapGR7	TMHMM2.0	TMhelix	   143   160
PrapGR7	TMHMM2.0	outside	   161   163
PrapGR7	TMHMM2.0	TMhelix	   164   186
PrapGR7	TMHMM2.0	inside	   187   281
PrapGR7	TMHMM2.0	TMhelix	   282   301
PrapGR7	TMHMM2.0	outside	   302   343
PrapGR7	TMHMM2.0	TMhelix	   344   366
PrapGR7	TMHMM2.0	inside	   367   373
```

# plot in postscript,
script for making the plot in gnuplot,
data for plot

---

```
# PrapGR6 Length: 419
# PrapGR6 Number of predicted TMHs:  8
# PrapGR6 Exp number of AAs in TMHs: 174.90462
# PrapGR6 Exp number, first 60 AAs:  29.26061
# PrapGR6 Total prob of N-in:        0.12093
# PrapGR6 POSSIBLE N-term signal sequence
PrapGR6	TMHMM2.0	outside	     1    19
PrapGR6	TMHMM2.0	TMhelix	    20    42
PrapGR6	TMHMM2.0	inside	    43    54
PrapGR6	TMHMM2.0	TMhelix	    55    77
PrapGR6	TMHMM2.0	outside	    78    86
PrapGR6	TMHMM2.0	TMhelix	    87   106
PrapGR6	TMHMM2.0	inside	   107   139
PrapGR6	TMHMM2.0	TMhelix	   140   162
PrapGR6	TMHMM2.0	outside	   163   197
PrapGR6	TMHMM2.0	TMhelix	   198   220
PrapGR6	TMHMM2.0	inside	   221   263
PrapGR6	TMHMM2.0	TMhelix	   264   286
PrapGR6	TMHMM2.0	outside	   287   312
PrapGR6	TMHMM2.0	TMhelix	   313   335
PrapGR6	TMHMM2.0	inside	   336   380
PrapGR6	TMHMM2.0	TMhelix	   381   403
PrapGR6	TMHMM2.0	outside	   404   419
```

# plot in postscript,
script for making the plot in gnuplot,
data for plot

---

```
# PrapGR5 Length: 412
# PrapGR5 Number of predicted TMHs:  7
# PrapGR5 Exp number of AAs in TMHs: 168.83285
# PrapGR5 Exp number, first 60 AAs:  22.72701
# PrapGR5 Total prob of N-in:        0.60123
# PrapGR5 POSSIBLE N-term signal sequence
PrapGR5	TMHMM2.0	inside	     1    48
PrapGR5	TMHMM2.0	TMhelix	    49    71
PrapGR5	TMHMM2.0	outside	    72    80
PrapGR5	TMHMM2.0	TMhelix	    81   103
PrapGR5	TMHMM2.0	inside	   104   123
PrapGR5	TMHMM2.0	TMhelix	   124   146
PrapGR5	TMHMM2.0	outside	   147   173
PrapGR5	TMHMM2.0	TMhelix	   174   196
PrapGR5	TMHMM2.0	inside	   197   259
PrapGR5	TMHMM2.0	TMhelix	   260   282
PrapGR5	TMHMM2.0	outside	   283   308
PrapGR5	TMHMM2.0	TMhelix	   309   331
PrapGR5	TMHMM2.0	inside	   332   379
PrapGR5	TMHMM2.0	TMhelix	   380   402
PrapGR5	TMHMM2.0	outside	   403   412
```

# plot in postscript,
script for making the plot in gnuplot,
data for plot

---

```
# PrapGR52 Length: 437
# PrapGR52 Number of predicted TMHs:  6
# PrapGR52 Exp number of AAs in TMHs: 139.67841
# PrapGR52 Exp number, first 60 AAs:  18.35339
# PrapGR52 Total prob of N-in:        0.98974
# PrapGR52 POSSIBLE N-term signal sequence
PrapGR52	TMHMM2.0	inside	     1    42
PrapGR52	TMHMM2.0	TMhelix	    43    65
PrapGR52	TMHMM2.0	outside	    66    90
PrapGR52	TMHMM2.0	TMhelix	    91   113
PrapGR52	TMHMM2.0	inside	   114   206
PrapGR52	TMHMM2.0	TMhelix	   207   229
PrapGR52	TMHMM2.0	outside	   230   288
PrapGR52	TMHMM2.0	TMhelix	   289   311
PrapGR52	TMHMM2.0	inside	   312   323
PrapGR52	TMHMM2.0	TMhelix	   324   346
PrapGR52	TMHMM2.0	outside	   347   388
PrapGR52	TMHMM2.0	TMhelix	   389   411
PrapGR52	TMHMM2.0	inside	   412   437
```

# plot in postscript,
script for making the plot in gnuplot,
data for plot

---

```
# PrapGR4 Length: 404
# PrapGR4 Number of predicted TMHs:  6
# PrapGR4 Exp number of AAs in TMHs: 126.35096
# PrapGR4 Exp number, first 60 AAs:  21.21884
# PrapGR4 Total prob of N-in:        0.98377
# PrapGR4 POSSIBLE N-term signal sequence
PrapGR4	TMHMM2.0	inside	     1    40
PrapGR4	TMHMM2.0	TMhelix	    41    63
PrapGR4	TMHMM2.0	outside	    64    77
PrapGR4	TMHMM2.0	TMhelix	    78   100
PrapGR4	TMHMM2.0	inside	   101   133
PrapGR4	TMHMM2.0	TMhelix	   134   153
PrapGR4	TMHMM2.0	outside	   154   201
PrapGR4	TMHMM2.0	TMhelix	   202   224
PrapGR4	TMHMM2.0	inside	   225   275
PrapGR4	TMHMM2.0	TMhelix	   276   298
PrapGR4	TMHMM2.0	outside	   299   307
PrapGR4	TMHMM2.0	TMhelix	   308   330
PrapGR4	TMHMM2.0	inside	   331   404
```

# plot in postscript,
script for making the plot in gnuplot,
data for plot

---

```
# PrapGR42 Length: 269
# PrapGR42 Number of predicted TMHs:  4
# PrapGR42 Exp number of AAs in TMHs: 89.29748
# PrapGR42 Exp number, first 60 AAs:  15.92421
# PrapGR42 Total prob of N-in:        0.96229
# PrapGR42 POSSIBLE N-term signal sequence
PrapGR42	TMHMM2.0	inside	     1    44
PrapGR42	TMHMM2.0	TMhelix	    45    67
PrapGR42	TMHMM2.0	outside	    68    81
PrapGR42	TMHMM2.0	TMhelix	    82   104
PrapGR42	TMHMM2.0	inside	   105   134
PrapGR42	TMHMM2.0	TMhelix	   135   157
PrapGR42	TMHMM2.0	outside	   158   166
PrapGR42	TMHMM2.0	TMhelix	   167   189
PrapGR42	TMHMM2.0	inside	   190   269
```

# plot in postscript,
script for making the plot in gnuplot,
data for plot

---

```
# PrapGR44.1 Length: 397
# PrapGR44.1 Number of predicted TMHs:  7
# PrapGR44.1 Exp number of AAs in TMHs: 155.39043
# PrapGR44.1 Exp number, first 60 AAs:  10.96812
# PrapGR44.1 Total prob of N-in:        0.95795
# PrapGR44.1 POSSIBLE N-term signal sequence
PrapGR44.1	TMHMM2.0	inside	     1    50
PrapGR44.1	TMHMM2.0	TMhelix	    51    70
PrapGR44.1	TMHMM2.0	outside	    71    79
PrapGR44.1	TMHMM2.0	TMhelix	    80   102
PrapGR44.1	TMHMM2.0	inside	   103   139
PrapGR44.1	TMHMM2.0	TMhelix	   140   162
PrapGR44.1	TMHMM2.0	outside	   163   171
PrapGR44.1	TMHMM2.0	TMhelix	   172   194
PrapGR44.1	TMHMM2.0	inside	   195   263
PrapGR44.1	TMHMM2.0	TMhelix	   264   286
PrapGR44.1	TMHMM2.0	outside	   287   300
PrapGR44.1	TMHMM2.0	TMhelix	   301   323
PrapGR44.1	TMHMM2.0	inside	   324   373
PrapGR44.1	TMHMM2.0	TMhelix	   374   396
PrapGR44.1	TMHMM2.0	outside	   397   397
```

# plot in postscript,
script for making the plot in gnuplot,
data for plot

---

```
# PrapGR44.2 Length: 396
# PrapGR44.2 Number of predicted TMHs:  7
# PrapGR44.2 Exp number of AAs in TMHs: 154.42479
# PrapGR44.2 Exp number, first 60 AAs:  12.19424
# PrapGR44.2 Total prob of N-in:        0.99624
# PrapGR44.2 POSSIBLE N-term signal sequence
PrapGR44.2	TMHMM2.0	inside	     1    50
PrapGR44.2	TMHMM2.0	TMhelix	    51    68
PrapGR44.2	TMHMM2.0	outside	    69    80
PrapGR44.2	TMHMM2.0	TMhelix	    81   103
PrapGR44.2	TMHMM2.0	inside	   104   136
PrapGR44.2	TMHMM2.0	TMhelix	   137   159
PrapGR44.2	TMHMM2.0	outside	   160   163
PrapGR44.2	TMHMM2.0	TMhelix	   164   186
PrapGR44.2	TMHMM2.0	inside	   187   266
PrapGR44.2	TMHMM2.0	TMhelix	   267   289
PrapGR44.2	TMHMM2.0	outside	   290   298
PrapGR44.2	TMHMM2.0	TMhelix	   299   321
PrapGR44.2	TMHMM2.0	inside	   322   372
PrapGR44.2	TMHMM2.0	TMhelix	   373   395
PrapGR44.2	TMHMM2.0	outside	   396   396
```

# plot in postscript,
script for making the plot in gnuplot,
data for plot

---

```
# PrapGR27 Length: 391
# PrapGR27 Number of predicted TMHs:  8
# PrapGR27 Exp number of AAs in TMHs: 174.15972
# PrapGR27 Exp number, first 60 AAs:  35.82364
# PrapGR27 Total prob of N-in:        0.15802
# PrapGR27 POSSIBLE N-term signal sequence
PrapGR27	TMHMM2.0	outside	     1    14
PrapGR27	TMHMM2.0	TMhelix	    15    37
PrapGR27	TMHMM2.0	inside	    38    43
PrapGR27	TMHMM2.0	TMhelix	    44    63
PrapGR27	TMHMM2.0	outside	    64    72
PrapGR27	TMHMM2.0	TMhelix	    73    95
PrapGR27	TMHMM2.0	inside	    96   124
PrapGR27	TMHMM2.0	TMhelix	   125   147
PrapGR27	TMHMM2.0	outside	   148   161
PrapGR27	TMHMM2.0	TMhelix	   162   184
PrapGR27	TMHMM2.0	inside	   185   248
PrapGR27	TMHMM2.0	TMhelix	   249   271
PrapGR27	TMHMM2.0	outside	   272   290
PrapGR27	TMHMM2.0	TMhelix	   291   313
PrapGR27	TMHMM2.0	inside	   314   367
PrapGR27	TMHMM2.0	TMhelix	   368   390
PrapGR27	TMHMM2.0	outside	   391   391
```

# plot in postscript,
script for making the plot in gnuplot,
data for plot

---

```
# PrapGR18 Length: 346
# PrapGR18 Number of predicted TMHs:  7
# PrapGR18 Exp number of AAs in TMHs: 149.97367
# PrapGR18 Exp number, first 60 AAs:  25.06739
# PrapGR18 Total prob of N-in:        0.08430
# PrapGR18 POSSIBLE N-term signal sequence
PrapGR18	TMHMM2.0	outside	     1    29
PrapGR18	TMHMM2.0	TMhelix	    30    52
PrapGR18	TMHMM2.0	inside	    53    58
PrapGR18	TMHMM2.0	TMhelix	    59    81
PrapGR18	TMHMM2.0	outside	    82   111
PrapGR18	TMHMM2.0	TMhelix	   112   134
PrapGR18	TMHMM2.0	inside	   135   146
PrapGR18	TMHMM2.0	TMhelix	   147   169
PrapGR18	TMHMM2.0	outside	   170   220
PrapGR18	TMHMM2.0	TMhelix	   221   240
PrapGR18	TMHMM2.0	inside	   241   246
PrapGR18	TMHMM2.0	TMhelix	   247   269
PrapGR18	TMHMM2.0	outside	   270   324
PrapGR18	TMHMM2.0	TMhelix	   325   344
PrapGR18	TMHMM2.0	inside	   345   346
```

# plot in postscript,
script for making the plot in gnuplot,
data for plot

---

```
# PrapGR61 Length: 340
# PrapGR61 Number of predicted TMHs:  6
# PrapGR61 Exp number of AAs in TMHs: 123.55363
# PrapGR61 Exp number, first 60 AAs:  21.85441
# PrapGR61 Total prob of N-in:        0.96903
# PrapGR61 POSSIBLE N-term signal sequence
PrapGR61	TMHMM2.0	inside	     1    34
PrapGR61	TMHMM2.0	TMhelix	    35    57
PrapGR61	TMHMM2.0	outside	    58    60
PrapGR61	TMHMM2.0	TMhelix	    61    78
PrapGR61	TMHMM2.0	inside	    79   106
PrapGR61	TMHMM2.0	TMhelix	   107   129
PrapGR61	TMHMM2.0	outside	   130   215
PrapGR61	TMHMM2.0	TMhelix	   216   235
PrapGR61	TMHMM2.0	inside	   236   241
PrapGR61	TMHMM2.0	TMhelix	   242   264
PrapGR61	TMHMM2.0	outside	   265   320
PrapGR61	TMHMM2.0	TMhelix	   321   338
PrapGR61	TMHMM2.0	inside	   339   340
```

# plot in postscript,
script for making the plot in gnuplot,
data for plot

---

```
# PrapGR65 Length: 354
# PrapGR65 Number of predicted TMHs:  7
# PrapGR65 Exp number of AAs in TMHs: 153.6354
# PrapGR65 Exp number, first 60 AAs:  22.76053
# PrapGR65 Total prob of N-in:        0.96939
# PrapGR65 POSSIBLE N-term signal sequence
PrapGR65	TMHMM2.0	inside	     1    31
PrapGR65	TMHMM2.0	TMhelix	    32    54
PrapGR65	TMHMM2.0	outside	    55    68
PrapGR65	TMHMM2.0	TMhelix	    69    88
PrapGR65	TMHMM2.0	inside	    89   114
PrapGR65	TMHMM2.0	TMhelix	   115   137
PrapGR65	TMHMM2.0	outside	   138   151
PrapGR65	TMHMM2.0	TMhelix	   152   169
PrapGR65	TMHMM2.0	inside	   170   221
PrapGR65	TMHMM2.0	TMhelix	   222   244
PrapGR65	TMHMM2.0	outside	   245   253
PrapGR65	TMHMM2.0	TMhelix	   254   276
PrapGR65	TMHMM2.0	inside	   277   323
PrapGR65	TMHMM2.0	TMhelix	   324   346
PrapGR65	TMHMM2.0	outside	   347   354
```

# plot in postscript,
script for making the plot in gnuplot,
data for plot

---

```
# PrapGR63 Length: 399
# PrapGR63 Number of predicted TMHs:  7
# PrapGR63 Exp number of AAs in TMHs: 140.3398
# PrapGR63 Exp number, first 60 AAs:  17.54379
# PrapGR63 Total prob of N-in:        0.76614
# PrapGR63 POSSIBLE N-term signal sequence
PrapGR63	TMHMM2.0	inside	     1    41
PrapGR63	TMHMM2.0	TMhelix	    42    64
PrapGR63	TMHMM2.0	outside	    65    78
PrapGR63	TMHMM2.0	TMhelix	    79   101
PrapGR63	TMHMM2.0	inside	   102   135
PrapGR63	TMHMM2.0	TMhelix	   136   155
PrapGR63	TMHMM2.0	outside	   156   164
PrapGR63	TMHMM2.0	TMhelix	   165   187
PrapGR63	TMHMM2.0	inside	   188   264
PrapGR63	TMHMM2.0	TMhelix	   265   287
PrapGR63	TMHMM2.0	outside	   288   296
PrapGR63	TMHMM2.0	TMhelix	   297   319
PrapGR63	TMHMM2.0	inside	   320   376
PrapGR63	TMHMM2.0	TMhelix	   377   396
PrapGR63	TMHMM2.0	outside	   397   399
```

# plot in postscript,
script for making the plot in gnuplot,
data for plot

---

```
# PrapGR12 Length: 345
# PrapGR12 Number of predicted TMHs:  7
# PrapGR12 Exp number of AAs in TMHs: 154.3397
# PrapGR12 Exp number, first 60 AAs:  33.04271
# PrapGR12 Total prob of N-in:        0.59517
# PrapGR12 POSSIBLE N-term signal sequence
PrapGR12	TMHMM2.0	inside	     1    24
PrapGR12	TMHMM2.0	TMhelix	    25    47
PrapGR12	TMHMM2.0	outside	    48    61
PrapGR12	TMHMM2.0	TMhelix	    62    82
PrapGR12	TMHMM2.0	inside	    83   102
PrapGR12	TMHMM2.0	TMhelix	   103   125
PrapGR12	TMHMM2.0	outside	   126   139
PrapGR12	TMHMM2.0	TMhelix	   140   162
PrapGR12	TMHMM2.0	inside	   163   212
PrapGR12	TMHMM2.0	TMhelix	   213   235
PrapGR12	TMHMM2.0	outside	   236   244
PrapGR12	TMHMM2.0	TMhelix	   245   267
PrapGR12	TMHMM2.0	inside	   268   315
PrapGR12	TMHMM2.0	TMhelix	   316   338
PrapGR12	TMHMM2.0	outside	   339   345
```

# plot in postscript,
script for making the plot in gnuplot,
data for plot

---

```
# PrapGR15 Length: 348
# PrapGR15 Number of predicted TMHs:  7
# PrapGR15 Exp number of AAs in TMHs: 159.10081
# PrapGR15 Exp number, first 60 AAs:  31.28765
# PrapGR15 Total prob of N-in:        0.40169
# PrapGR15 POSSIBLE N-term signal sequence
PrapGR15	TMHMM2.0	inside	     1    29
PrapGR15	TMHMM2.0	TMhelix	    30    52
PrapGR15	TMHMM2.0	outside	    53    66
PrapGR15	TMHMM2.0	TMhelix	    67    89
PrapGR15	TMHMM2.0	inside	    90   109
PrapGR15	TMHMM2.0	TMhelix	   110   132
PrapGR15	TMHMM2.0	outside	   133   141
PrapGR15	TMHMM2.0	TMhelix	   142   164
PrapGR15	TMHMM2.0	inside	   165   219
PrapGR15	TMHMM2.0	TMhelix	   220   242
PrapGR15	TMHMM2.0	outside	   243   251
PrapGR15	TMHMM2.0	TMhelix	   252   274
PrapGR15	TMHMM2.0	inside	   275   317
PrapGR15	TMHMM2.0	TMhelix	   318   340
PrapGR15	TMHMM2.0	outside	   341   348
```

# plot in postscript,
script for making the plot in gnuplot,
data for plot

---

```
# PrapGR17 Length: 221
# PrapGR17 Number of predicted TMHs:  2
# PrapGR17 Exp number of AAs in TMHs: 60.6595
# PrapGR17 Exp number, first 60 AAs:  5.19641
# PrapGR17 Total prob of N-in:        0.20592
PrapGR17	TMHMM2.0	outside	     1    84
PrapGR17	TMHMM2.0	TMhelix	    85   107
PrapGR17	TMHMM2.0	inside	   108   195
PrapGR17	TMHMM2.0	TMhelix	   196   218
PrapGR17	TMHMM2.0	outside	   219   221
```

# plot in postscript,
script for making the plot in gnuplot,
data for plot

---

```
# PrapGR13 Length: 356
# PrapGR13 Number of predicted TMHs:  7
# PrapGR13 Exp number of AAs in TMHs: 154.40811
# PrapGR13 Exp number, first 60 AAs:  20.56217
# PrapGR13 Total prob of N-in:        0.98993
# PrapGR13 POSSIBLE N-term signal sequence
PrapGR13	TMHMM2.0	inside	     1    31
PrapGR13	TMHMM2.0	TMhelix	    32    49
PrapGR13	TMHMM2.0	outside	    50    63
PrapGR13	TMHMM2.0	TMhelix	    64    86
PrapGR13	TMHMM2.0	inside	    87   106
PrapGR13	TMHMM2.0	TMhelix	   107   129
PrapGR13	TMHMM2.0	outside	   130   148
PrapGR13	TMHMM2.0	TMhelix	   149   171
PrapGR13	TMHMM2.0	inside	   172   227
PrapGR13	TMHMM2.0	TMhelix	   228   250
PrapGR13	TMHMM2.0	outside	   251   254
PrapGR13	TMHMM2.0	TMhelix	   255   277
PrapGR13	TMHMM2.0	inside	   278   332
PrapGR13	TMHMM2.0	TMhelix	   333   355
PrapGR13	TMHMM2.0	outside	   356   356
```

# plot in postscript,
script for making the plot in gnuplot,
data for plot

---

```
# PrapGR31 Length: 338
# PrapGR31 Number of predicted TMHs:  7
# PrapGR31 Exp number of AAs in TMHs: 152.8205
# PrapGR31 Exp number, first 60 AAs:  26.97997
# PrapGR31 Total prob of N-in:        0.97304
# PrapGR31 POSSIBLE N-term signal sequence
PrapGR31	TMHMM2.0	inside	     1    30
PrapGR31	TMHMM2.0	TMhelix	    31    53
PrapGR31	TMHMM2.0	outside	    54    56
PrapGR31	TMHMM2.0	TMhelix	    57    74
PrapGR31	TMHMM2.0	inside	    75    97
PrapGR31	TMHMM2.0	TMhelix	    98   117
PrapGR31	TMHMM2.0	outside	   118   126
PrapGR31	TMHMM2.0	TMhelix	   127   149
PrapGR31	TMHMM2.0	inside	   150   203
PrapGR31	TMHMM2.0	TMhelix	   204   226
PrapGR31	TMHMM2.0	outside	   227   240
PrapGR31	TMHMM2.0	TMhelix	   241   263
PrapGR31	TMHMM2.0	inside	   264   307
PrapGR31	TMHMM2.0	TMhelix	   308   330
PrapGR31	TMHMM2.0	outside	   331   338
```

# plot in postscript,
script for making the plot in gnuplot,
data for plot

---

```
# PrapGR64 Length: 285
# PrapGR64 Number of predicted TMHs:  6
# PrapGR64 Exp number of AAs in TMHs: 128.20404
# PrapGR64 Exp number, first 60 AAs:  25.30365
# PrapGR64 Total prob of N-in:        0.67485
# PrapGR64 POSSIBLE N-term signal sequence
PrapGR64	TMHMM2.0	inside	     1    33
PrapGR64	TMHMM2.0	TMhelix	    34    53
PrapGR64	TMHMM2.0	outside	    54    62
PrapGR64	TMHMM2.0	TMhelix	    63    85
PrapGR64	TMHMM2.0	inside	    86   109
PrapGR64	TMHMM2.0	TMhelix	   110   132
PrapGR64	TMHMM2.0	outside	   133   146
PrapGR64	TMHMM2.0	TMhelix	   147   169
PrapGR64	TMHMM2.0	inside	   170   225
PrapGR64	TMHMM2.0	TMhelix	   226   245
PrapGR64	TMHMM2.0	outside	   246   254
PrapGR64	TMHMM2.0	TMhelix	   255   277
PrapGR64	TMHMM2.0	inside	   278   285
```

# plot in postscript,
script for making the plot in gnuplot,
data for plot

---

```
# PrapGR14 Length: 204
# PrapGR14 Number of predicted TMHs:  3
# PrapGR14 Exp number of AAs in TMHs: 66.9817300000001
# PrapGR14 Exp number, first 60 AAs:  5.57698
# PrapGR14 Total prob of N-in:        0.72017
PrapGR14	TMHMM2.0	inside	     1    81
PrapGR14	TMHMM2.0	TMhelix	    82   101
PrapGR14	TMHMM2.0	outside	   102   105
PrapGR14	TMHMM2.0	TMhelix	   106   128
PrapGR14	TMHMM2.0	inside	   129   180
PrapGR14	TMHMM2.0	TMhelix	   181   203
PrapGR14	TMHMM2.0	outside	   204   204
```

# plot in postscript,
script for making the plot in gnuplot,
data for plot

---
