# Supplementary material for: Sympatric Pieris butterfly species exhibit a high conservation of chemoreceptors
Source: Front Cell Neurosci. 2023 May 11;17:1155405. doi: 10.3389/fncel.2023.1155405 (PMC10210156; doi:10.3389/fncel.2023.1155405)
Supplement: Supplementary file 5 [file Data_Sheet_2.ZIP › S5_Transmembrane domain prediction/Pbrassicae_Chemoreceptor_TOPCONS.html]

TOPCONS2 predictions


#### 

| No. | Length | numTM | SignalPeptide | RunTime(s) | SequenceName | Prediction | Source |
| --- | --- | --- | --- | --- | --- | --- | --- |
| 1 | 389 | 7 | No | 0.0 | PbraOR1 | Fig\_all Fig\_topcons  Dumped prediction  deltaG  Topology view | cached |
| 2 | 394 | 6 | No | 0.0 | PbraOR11 | Fig\_all Fig\_topcons  Dumped prediction  deltaG  Topology view | cached |
| 3 | 388 | 6 | No | 0.0 | PbraOR12 | Fig\_all Fig\_topcons  Dumped prediction  deltaG  Topology view | cached |
| 4 | 387 | 6 | No | 0.0 | PbraOR13 | Fig\_all Fig\_topcons  Dumped prediction  deltaG  Topology view | cached |
| 5 | 393 | 6 | No | 0.0 | PbraOR14 | Fig\_all Fig\_topcons  Dumped prediction  deltaG  Topology view | cached |
| 6 | 392 | 6 | No | 0.0 | PbraOR15 | Fig\_all Fig\_topcons  Dumped prediction  deltaG  Topology view | cached |
| 7 | 397 | 7 | No | 0.0 | PbraOR16 | Fig\_all Fig\_topcons  Dumped prediction  deltaG  Topology view | cached |
| 8 | 391 | 6 | No | 0.0 | PbraOR17 | Fig\_all Fig\_topcons  Dumped prediction  deltaG  Topology view | cached |
| 9 | 406 | 7 | No | 0.0 | PbraOR18 | Fig\_all Fig\_topcons  Dumped prediction  deltaG  Topology view | cached |
| 10 | 410 | 6 | No | 0.0 | PbraOR19 | Fig\_all Fig\_topcons  Dumped prediction  deltaG  Topology view | cached |
| 11 | 387 | 6 | No | 0.0 | PbraOR2 | Fig\_all Fig\_topcons  Dumped prediction  deltaG  Topology view | cached |
| 12 | 392 | 6 | No | 0.0 | PbraOR20 | Fig\_all Fig\_topcons  Dumped prediction  deltaG  Topology view | cached |
| 13 | 387 | 6 | No | 0.0 | PbraOR23 | Fig\_all Fig\_topcons  Dumped prediction  deltaG  Topology view | cached |
| 14 | 408 | 7 | No | 0.0 | PbraOR24 | Fig\_all Fig\_topcons  Dumped prediction  deltaG  Topology view | cached |
| 15 | 411 | 7 | No | 0.0 | PbraOR25 | Fig\_all Fig\_topcons  Dumped prediction  deltaG  Topology view | cached |
| 16 | 405 | 7 | No | 0.0 | PbraOR26 | Fig\_all Fig\_topcons  Dumped prediction  deltaG  Topology view | cached |
| 17 | 406 | 7 | No | 0.0 | PbraOR27 | Fig\_all Fig\_topcons  Dumped prediction  deltaG  Topology view | cached |
| 18 | 410 | 7 | No | 0.0 | PbraOR28 | Fig\_all Fig\_topcons  Dumped prediction  deltaG  Topology view | cached |
| 19 | 394 | 7 | No | 0.0 | PbraOR29 | Fig\_all Fig\_topcons  Dumped prediction  deltaG  Topology view | cached |
| 20 | 405 | 7 | No | 0.0 | PbraOR3 | Fig\_all Fig\_topcons  Dumped prediction  deltaG  Topology view | cached |
| 21 | 397 | 7 | No | 0.0 | PbraOR30 | Fig\_all Fig\_topcons  Dumped prediction  deltaG  Topology view | cached |
| 22 | 390 | 7 | No | 0.0 | PbraOR32 | Fig\_all Fig\_topcons  Dumped prediction  deltaG  Topology view | cached |
| 23 | 366 | 7 | No | 0.0 | PbraOR33 | Fig\_all Fig\_topcons  Dumped prediction  deltaG  Topology view | cached |
| 24 | 372 | 6 | No | 0.0 | PbraOR34 | Fig\_all Fig\_topcons  Dumped prediction  deltaG  Topology view | cached |
| 25 | 399 | 7 | No | 0.0 | PbraOR35 | Fig\_all Fig\_topcons  Dumped prediction  deltaG  Topology view | cached |
| 26 | 419 | 6 | No | 0.0 | PbraOR36 | Fig\_all Fig\_topcons  Dumped prediction  deltaG  Topology view | cached |
| 27 | 410 | 7 | No | 0.0 | PbraOR37 | Fig\_all Fig\_topcons  Dumped prediction  deltaG  Topology view | cached |
| 28 | 391 | 6 | No | 0.0 | PbraOR38 | Fig\_all Fig\_topcons  Dumped prediction  deltaG  Topology view | cached |
| 29 | 391 | 6 | No | 0.0 | PbraOR39 | Fig\_all Fig\_topcons  Dumped prediction  deltaG  Topology view | cached |
| 30 | 396 | 6 | No | 0.0 | PbraOR4 | Fig\_all Fig\_topcons  Dumped prediction  deltaG  Topology view | cached |
| 31 | 390 | 6 | No | 0.0 | PbraOR40 | Fig\_all Fig\_topcons  Dumped prediction  deltaG  Topology view | cached |
| 32 | 392 | 7 | No | 0.0 | PbraOR41 | Fig\_all Fig\_topcons  Dumped prediction  deltaG  Topology view | cached |
| 33 | 427 | 7 | No | 0.0 | PbraOR43 | Fig\_all Fig\_topcons  Dumped prediction  deltaG  Topology view | cached |
| 34 | 389 | 7 | No | 0.0 | PbraOR44 | Fig\_all Fig\_topcons  Dumped prediction  deltaG  Topology view | cached |
| 35 | 385 | 7 | No | 0.0 | PbraOR45a | Fig\_all Fig\_topcons  Dumped prediction  deltaG  Topology view | cached |
| 36 | 385 | 7 | No | 0.0 | PbraOR45b | Fig\_all Fig\_topcons  Dumped prediction  deltaG  Topology view | cached |
| 37 | 392 | 7 | No | 0.0 | PbraOR46 | Fig\_all Fig\_topcons  Dumped prediction  deltaG  Topology view | cached |
| 38 | 441 | 7 | No | 0.0 | PbraOR47 | Fig\_all Fig\_topcons  Dumped prediction  deltaG  Topology view | cached |
| 39 | 393 | 7 | No | 0.0 | PbraOR48 | Fig\_all Fig\_topcons  Dumped prediction  deltaG  Topology view | cached |
| 40 | 394 | 7 | No | 0.0 | PbraOR49 | Fig\_all Fig\_topcons  Dumped prediction  deltaG  Topology view | cached |
| 41 | 407 | 7 | No | 0.0 | PbraOR5 | Fig\_all Fig\_topcons  Dumped prediction  deltaG  Topology view | cached |
| 42 | 394 | 7 | No | 0.0 | PbraOR50 | Fig\_all Fig\_topcons  Dumped prediction  deltaG  Topology view | cached |
| 43 | 389 | 7 | No | 0.0 | PbraOR51 | Fig\_all Fig\_topcons  Dumped prediction  deltaG  Topology view | cached |
| 44 | 388 | 7 | No | 0.0 | PbraOR52 | Fig\_all Fig\_topcons  Dumped prediction  deltaG  Topology view | cached |
| 45 | 432 | 7 | No | 0.0 | PbraOR53 | Fig\_all Fig\_topcons  Dumped prediction  deltaG  Topology view | cached |
| 46 | 416 | 7 | No | 0.0 | PbraOR54 | Fig\_all Fig\_topcons  Dumped prediction  deltaG  Topology view | cached |
| 47 | 417 | 7 | No | 0.0 | PbraOR55 | Fig\_all Fig\_topcons  Dumped prediction  deltaG  Topology view | cached |
| 48 | 421 | 8 | No | 0.0 | PbraOR56 | Fig\_all Fig\_topcons  Dumped prediction  deltaG  Topology view | cached |
| 49 | 397 | 7 | No | 0.0 | PbraOR57 | Fig\_all Fig\_topcons  Dumped prediction  deltaG  Topology view | cached |
| 50 | 397 | 7 | No | 0.0 | PbraOR58 | Fig\_all Fig\_topcons  Dumped prediction  deltaG  Topology view | cached |
| 51 | 438 | 6 | No | 0.0 | PbraOR59 | Fig\_all Fig\_topcons  Dumped prediction  deltaG  Topology view | cached |
| 52 | 393 | 6 | No | 0.0 | PbraOR6 | Fig\_all Fig\_topcons  Dumped prediction  deltaG  Topology view | cached |
| 53 | 392 | 7 | No | 0.0 | PbraOR60 | Fig\_all Fig\_topcons  Dumped prediction  deltaG  Topology view | cached |
| 54 | 395 | 7 | No | 0.0 | PbraOR61 | Fig\_all Fig\_topcons  Dumped prediction  deltaG  Topology view | cached |
| 55 | 393 | 7 | No | 0.0 | PbraOR62 | Fig\_all Fig\_topcons  Dumped prediction  deltaG  Topology view | cached |
| 56 | 380 | 7 | No | 0.0 | PbraOR63 | Fig\_all Fig\_topcons  Dumped prediction  deltaG  Topology view | cached |
| 57 | 400 | 7 | No | 0.0 | PbraOR7 | Fig\_all Fig\_topcons  Dumped prediction  deltaG  Topology view | cached |
| 58 | 399 | 7 | No | 0.0 | PbraOR8 | Fig\_all Fig\_topcons  Dumped prediction  deltaG  Topology view | cached |
| 59 | 400 | 7 | No | 0.0 | PbraOR9 | Fig\_all Fig\_topcons  Dumped prediction  deltaG  Topology view | cached |
| 60 | 471 | 7 | No | 0.0 | PbraOrco | Fig\_all Fig\_topcons  Dumped prediction  deltaG  Topology view | cached |
| 61 | 457 | 7 | No | 11.9 | PbraGR1 | Fig\_all Fig\_topcons  Dumped prediction  deltaG  Topology view | newrun |
| 62 | 435 | 7 | No | 11.9 | PbraGR10 | Fig\_all Fig\_topcons  Dumped prediction  deltaG  Topology view | newrun |
| 63 | 420 | 8 | No | 10.1 | PbraGR16 | Fig\_all Fig\_topcons  Dumped prediction  deltaG  Topology view | newrun |
| 64 | 222 | 3 | No | 9.6 | PbraGR17 | Fig\_all Fig\_topcons  Dumped prediction  deltaG  Topology view | newrun |
| 65 | 430 | 7 | No | 18.3 | PbraGR2 | Fig\_all Fig\_topcons  Dumped prediction  deltaG  Topology view | newrun |
| 66 | 388 | 8 | No | 15.2 | PbraGR22 | Fig\_all Fig\_topcons  Dumped prediction  deltaG  Topology view | newrun |
| 67 | 391 | 7 | No | 14.3 | PbraGR27 | Fig\_all Fig\_topcons  Dumped prediction  deltaG  Topology view | newrun |
| 68 | 383 | 7 | No | 12.9 | PbraGR28 | Fig\_all Fig\_topcons  Dumped prediction  deltaG  Topology view | newrun |
| 69 | 474 | 7 | No | 12.1 | PbraGR3 | Fig\_all Fig\_topcons  Dumped prediction  deltaG  Topology view | newrun |
| 70 | 404 | 7 | No | 26.1 | PbraGR4 | Fig\_all Fig\_topcons  Dumped prediction  deltaG  Topology view | newrun |
| 71 | 279 | 5 | No | 15.3 | PbraGR46 | Fig\_all Fig\_topcons  Dumped prediction  deltaG  Topology view | newrun |
| 72 | 409 | 8 | No | 41.8 | PbraGR5 | Fig\_all Fig\_topcons  Dumped prediction  deltaG  Topology view | newrun |
| 73 | 437 | 7 | No | 27.0 | PbraGR52 | Fig\_all Fig\_topcons  Dumped prediction  deltaG  Topology view | newrun |
| 74 | 432 | 8 | No | 40.2 | PbraGR6 | Fig\_all Fig\_topcons  Dumped prediction  deltaG  Topology view | newrun |
| 75 | 400 | 6 | No | 16.4 | PbraGR63 | Fig\_all Fig\_topcons  Dumped prediction  deltaG  Topology view | newrun |
| 76 | 385 | 7 | No | 27.0 | PbraGR66 | Fig\_all Fig\_topcons  Dumped prediction  deltaG  Topology view | newrun |
| 77 | 384 | 8 | No | 25.6 | PbraGR68.1 | Fig\_all Fig\_topcons  Dumped prediction  deltaG  Topology view | newrun |
| 78 | 396 | 8 | No | 23.0 | PbraGR68.2 | Fig\_all Fig\_topcons  Dumped prediction  deltaG  Topology view | newrun |
| 79 | 374 | 7 | No | 23.3 | PbraGR7 | Fig\_all Fig\_topcons  Dumped prediction  deltaG  Topology view | newrun |
| 80 | 374 | 7 | No | 22.6 | PbraGR8.1 | Fig\_all Fig\_topcons  Dumped prediction  deltaG  Topology view | newrun |
| 81 | 385 | 6 | No | 9.7 | PbraGR8.2 | Fig\_all Fig\_topcons  Dumped prediction  deltaG  Topology view | newrun |
| 82 | 380 | 8 | No | 11.8 | PbraGR8.3 | Fig\_all Fig\_topcons  Dumped prediction  deltaG  Topology view | newrun |
| 83 | 375 | 6 | No | 12.9 | PbraGR8.4 | Fig\_all Fig\_topcons  Dumped prediction  deltaG  Topology view | newrun |
| 84 | 459 | 8 | No | 23.7 | PbraGR9 | Fig\_all Fig\_topcons  Dumped prediction  deltaG  Topology view | newrun |
| 85 | 802 | 3 | Yes | 49.6 | PbraIR21a | Fig\_all Fig\_topcons  Dumped prediction  deltaG  Topology view | newrun |
| 86 | 664 | 3 | No | 42.2 | PbraIR4 | Fig\_all Fig\_topcons  Dumped prediction  deltaG  Topology view | newrun |
| 87 | 593 | 3 | No | 38.4 | PbraIR41a | Fig\_all Fig\_topcons  Dumped prediction  deltaG  Topology view | newrun |
| 88 | 609 | 3 | No | 42.7 | PbraIR64a | Fig\_all Fig\_topcons  Dumped prediction  deltaG  Topology view | newrun |
| 89 | 695 | 4 | Yes | 41.1 | PbraIR68a | Fig\_all Fig\_topcons  Dumped prediction  deltaG  Topology view | newrun |
| 90 | 600 | 3 | No | 48.6 | PbraIR75d | Fig\_all Fig\_topcons  Dumped prediction  deltaG  Topology view | newrun |
| 91 | 607 | 3 | Yes | 46.4 | PbraIR75p.2 | Fig\_all Fig\_topcons  Dumped prediction  deltaG  Topology view | newrun |
| 92 | 639 | 3 | Yes | 53.6 | PbraIR87a | Fig\_all Fig\_topcons  Dumped prediction  deltaG  Topology view | newrun |
| 93 | 638 | 3 | Yes | 48.2 | PbraIR75p.1 | Fig\_all Fig\_topcons  Dumped prediction  deltaG  Topology view | newrun |
| 94 | 532 | 3 | No | 38.7 | PbraIR76b | Fig\_all Fig\_topcons  Dumped prediction  deltaG  Topology view | newrun |
| 95 | 619 | 3 | Yes | 45.7 | PbraIR75q.2 | Fig\_all Fig\_topcons  Dumped prediction  deltaG  Topology view | newrun |
| 96 | 922 | 3 | No | 63.8 | PbraIR25a1 | Fig\_all Fig\_topcons  Dumped prediction  deltaG  Topology view | newrun |
| 97 | 614 | 3 | No | 71.8 | PbraIR100f | Fig\_all Fig\_topcons  Dumped prediction  deltaG  Topology view | newrun |
| 98 | 668 | 3 | No | 52.9 | PbraIR40a | Fig\_all Fig\_topcons  Dumped prediction  deltaG  Topology view | newrun |
| 99 | 852 | 3 | Yes | 60.6 | PbraIR8a | Fig\_all Fig\_topcons  Dumped prediction  deltaG  Topology view | newrun |
| 100 | 867 | 3 | Yes | 48.6 | PbraIR93a | Fig\_all Fig\_topcons  Dumped prediction  deltaG  Topology view | newrun |
| 101 | 909 | 3 | No | 65.2 | PbraIR25a2 | Fig\_all Fig\_topcons  Dumped prediction  deltaG  Topology view | newrun |
| 102 | 608 | 3 | Yes | 52.0 | PbraIR75q.1 | Fig\_all Fig\_topcons  Dumped prediction  deltaG  Topology view | newrun |
| 103 | 204 | 3 | No | 447.8 | PbraGR14 | Fig\_all Fig\_topcons  Dumped prediction  deltaG  Topology view | newrun |
| 104 | 356 | 7 | No | 447.2 | PbraGR13 | Fig\_all Fig\_topcons  Dumped prediction  deltaG  Topology view | newrun |
| 105 | 338 | 7 | No | 418.5 | PbraGR31 | Fig\_all Fig\_topcons  Dumped prediction  deltaG  Topology view | newrun |
| 106 | 347 | 7 | No | 421.3 | PbraGR29 | Fig\_all Fig\_topcons  Dumped prediction  deltaG  Topology view | newrun |
| 107 | 346 | 7 | No | 442.5 | PbraGR18 | Fig\_all Fig\_topcons  Dumped prediction  deltaG  Topology view | newrun |
| 108 | 349 | 8 | No | 447.6 | PbraGR15 | Fig\_all Fig\_topcons  Dumped prediction  deltaG  Topology view | newrun |
| 109 | 345 | 8 | No | 444.9 | PbraGR12 | Fig\_all Fig\_topcons  Dumped prediction  deltaG  Topology view | newrun |
| 110 | 349 | 7 | No | 421.7 | PbraGR30 | Fig\_all Fig\_topcons  Dumped prediction  deltaG  Topology view | newrun |
| 111 | 401 | 3 | No | 843.7 | PbraIR2 | Fig\_all Fig\_topcons  Dumped prediction  deltaG  Topology view | newrun |
| 112 | 578 | 3 | Yes | 959.0 | PbraIR100d | Fig\_all Fig\_topcons  Dumped prediction  deltaG  Topology view | newrun |
| 113 | 580 | 3 | Yes | 961.0 | PbraIR100c | Fig\_all Fig\_topcons  Dumped prediction  deltaG  Topology view | newrun |
| 114 | 611 | 3 | No | 945.3 | PbraIR143 | Fig\_all Fig\_topcons  Dumped prediction  deltaG  Topology view | newrun |
| 115 | 283 | 6 | No | 998.5 | PbraGR64 | Fig\_all Fig\_topcons  Dumped prediction  deltaG  Topology view | newrun |
| 116 | 642 | 3 | Yes | 998.8 | PbraIR1.1 | Fig\_all Fig\_topcons  Dumped prediction  deltaG  Topology view | newrun |
| 117 | 585 | 4 | No | 973.4 | PbraIR31a | Fig\_all Fig\_topcons  Dumped prediction  deltaG  Topology view | newrun |
| 118 | 671 | 4 | No | 997.8 | PbraIR100a | Fig\_all Fig\_topcons  Dumped prediction  deltaG  Topology view | newrun |
| 119 | 645 | 3 | Yes | 997.3 | PbraIR1.2 | Fig\_all Fig\_topcons  Dumped prediction  deltaG  Topology view | newrun |
| 120 | 396 | 7 | No | 1030.4 | PbraGR44.2 | Fig\_all Fig\_topcons  Dumped prediction  deltaG  Topology view | newrun |
| 121 | 340 | 7 | No | 1022.3 | PbraGR61 | Fig\_all Fig\_topcons  Dumped prediction  deltaG  Topology view | newrun |
| 122 | 397 | 7 | No | 1030.6 | PbraGR44.1 | Fig\_all Fig\_topcons  Dumped prediction  deltaG  Topology view | newrun |
| 123 | 361 | 8 | No | 1032.9 | PbraGR54 | Fig\_all Fig\_topcons  Dumped prediction  deltaG  Topology view | newrun |
| 124 | 351 | 7 | No | 1023.4 | PbraGR65 | Fig\_all Fig\_topcons  Dumped prediction  deltaG  Topology view | newrun |
| 125 | 270 | 4 | No | 1026.1 | PbraGR42 | Fig\_all Fig\_topcons  Dumped prediction  deltaG  Topology view | newrun |
| 126 | 592 | 3 | No | 1132.3 | PbraIR7d.2 | Fig\_all Fig\_topcons  Dumped prediction  deltaG  Topology view | newrun |
| 127 | 595 | 3 | No | 1090.6 | PbraIR7d.3 | Fig\_all Fig\_topcons  Dumped prediction  deltaG  Topology view | newrun |
| 128 | 633 | 3 | Yes | 1092.2 | PbraIR85a | Fig\_all Fig\_topcons  Dumped prediction  deltaG  Topology view | newrun |
| 129 | 586 | 3 | No | 1134.0 | PbraIR7d.2.1 | Fig\_all Fig\_topcons  Dumped prediction  deltaG  Topology view | newrun |
| 130 | 594 | 4 | No | 1134.3 | PbraIR7d.2.2 | Fig\_all Fig\_topcons  Dumped prediction  deltaG  Topology view | newrun |
