# Supplementary material for: Sympatric Pieris butterfly species exhibit a high conservation of chemoreceptors
Source: Front Cell Neurosci. 2023 May 11;17:1155405. doi: 10.3389/fncel.2023.1155405 (PMC10210156; doi:10.3389/fncel.2023.1155405)
Supplement: Supplementary file 5 [file Data_Sheet_2.ZIP › S5_Transmembrane domain prediction/Prapae_Chemoreceptor_TOPCONS.html]

TOPCONS2 predictions


#### 

| No. | Length | numTM | SignalPeptide | RunTime(s) | SequenceName | Prediction | Source |
| --- | --- | --- | --- | --- | --- | --- | --- |
| 1 | 398 | 7 | No | 0.0 | PrapOR53 | Fig\_all Fig\_topcons  Dumped prediction  deltaG  Topology view | cached |
| 2 | 459 | 7 | No | 13.4 | PrapGR1 | Fig\_all Fig\_topcons  Dumped prediction  deltaG  Topology view | newrun |
| 3 | 427 | 7 | No | 10.9 | PrapGR10 | Fig\_all Fig\_topcons  Dumped prediction  deltaG  Topology view | newrun |
| 4 | 430 | 7 | No | 29.8 | PrapGR2 | Fig\_all Fig\_topcons  Dumped prediction  deltaG  Topology view | newrun |
| 5 | 388 | 7 | No | 20.9 | PrapGR22 | Fig\_all Fig\_topcons  Dumped prediction  deltaG  Topology view | newrun |
| 6 | 391 | 7 | No | 20.6 | PrapGR27 | Fig\_all Fig\_topcons  Dumped prediction  deltaG  Topology view | newrun |
| 7 | 383 | 8 | No | 20.3 | PrapGR28 | Fig\_all Fig\_topcons  Dumped prediction  deltaG  Topology view | newrun |
| 8 | 475 | 7 | No | 28.4 | PrapGR3 | Fig\_all Fig\_topcons  Dumped prediction  deltaG  Topology view | newrun |
| 9 | 404 | 7 | No | 27.7 | PrapGR4 | Fig\_all Fig\_topcons  Dumped prediction  deltaG  Topology view | newrun |
| 10 | 412 | 8 | No | 37.2 | PrapGR5 | Fig\_all Fig\_topcons  Dumped prediction  deltaG  Topology view | newrun |
| 11 | 437 | 7 | No | 25.4 | PrapGR52 | Fig\_all Fig\_topcons  Dumped prediction  deltaG  Topology view | newrun |
| 12 | 419 | 8 | No | 37.1 | PrapGR6 | Fig\_all Fig\_topcons  Dumped prediction  deltaG  Topology view | newrun |
| 13 | 402 | 8 | No | 18.7 | PrapGR68.2 | Fig\_all Fig\_topcons  Dumped prediction  deltaG  Topology view | newrun |
| 14 | 373 | 7 | No | 20.3 | PrapGR7 | Fig\_all Fig\_topcons  Dumped prediction  deltaG  Topology view | newrun |
| 15 | 374 | 7 | No | 14.9 | PrapGR8.1 | Fig\_all Fig\_topcons  Dumped prediction  deltaG  Topology view | newrun |
| 16 | 384 | 7 | No | 14.7 | PrapGR8.2 | Fig\_all Fig\_topcons  Dumped prediction  deltaG  Topology view | newrun |
| 17 | 379 | 6 | No | 14.8 | PrapGR8.3 | Fig\_all Fig\_topcons  Dumped prediction  deltaG  Topology view | newrun |
| 18 | 372 | 6 | No | 15.1 | PrapGR8.4 | Fig\_all Fig\_topcons  Dumped prediction  deltaG  Topology view | newrun |
| 19 | 454 | 8 | No | 25.0 | PrapGR9 | Fig\_all Fig\_topcons  Dumped prediction  deltaG  Topology view | newrun |
| 20 | 909 | 3 | No | 73.8 | PrapIR25a2 | Fig\_all Fig\_topcons  Dumped prediction  deltaG  Topology view | newrun |
| 21 | 640 | 4 | No | 55.0 | PrapIR4 | Fig\_all Fig\_topcons  Dumped prediction  deltaG  Topology view | newrun |
| 22 | 668 | 3 | No | 77.2 | PrapIR40a | Fig\_all Fig\_topcons  Dumped prediction  deltaG  Topology view | newrun |
| 23 | 590 | 3 | No | 58.4 | PrapIR41a | Fig\_all Fig\_topcons  Dumped prediction  deltaG  Topology view | newrun |
| 24 | 598 | 3 | No | 58.4 | PrapIR64a | Fig\_all Fig\_topcons  Dumped prediction  deltaG  Topology view | newrun |
| 25 | 639 | 3 | Yes | 71.2 | PrapIR75q.2 | Fig\_all Fig\_topcons  Dumped prediction  deltaG  Topology view | newrun |
| 26 | 638 | 3 | Yes | 64.0 | PrapIR75p.1 | Fig\_all Fig\_topcons  Dumped prediction  deltaG  Topology view | newrun |
| 27 | 615 | 3 | Yes | 71.0 | PrapIR75q.1 | Fig\_all Fig\_topcons  Dumped prediction  deltaG  Topology view | newrun |
| 28 | 389 | 7 | No | 32.3 | PrapOR1 | Fig\_all Fig\_topcons  Dumped prediction  deltaG  Topology view | newrun |
| 29 | 852 | 3 | Yes | 81.5 | PrapIR8a | Fig\_all Fig\_topcons  Dumped prediction  deltaG  Topology view | newrun |
| 30 | 532 | 3 | No | 60.3 | PrapIR76b1 | Fig\_all Fig\_topcons  Dumped prediction  deltaG  Topology view | newrun |
| 31 | 608 | 3 | Yes | 69.1 | PrapIR75p.2 | Fig\_all Fig\_topcons  Dumped prediction  deltaG  Topology view | newrun |
| 32 | 569 | 3 | Yes | 92.8 | PrapIR100e | Fig\_all Fig\_topcons  Dumped prediction  deltaG  Topology view | newrun |
| 33 | 532 | 3 | No | 51.3 | PrapIR76b2 | Fig\_all Fig\_topcons  Dumped prediction  deltaG  Topology view | newrun |
| 34 | 867 | 3 | Yes | 50.4 | PrapIR93a | Fig\_all Fig\_topcons  Dumped prediction  deltaG  Topology view | newrun |
| 35 | 609 | 4 | No | 35.8 | PrapIR100f | Fig\_all Fig\_topcons  Dumped prediction  deltaG  Topology view | newrun |
| 36 | 696 | 4 | Yes | 61.1 | PrapIR68a | Fig\_all Fig\_topcons  Dumped prediction  deltaG  Topology view | newrun |
| 37 | 388 | 6 | No | 30.5 | PrapOR12 | Fig\_all Fig\_topcons  Dumped prediction  deltaG  Topology view | newrun |
| 38 | 637 | 6 | No | 90.8 | PrapIR87a | Fig\_all Fig\_topcons  Dumped prediction  deltaG  Topology view | newrun |
| 39 | 599 | 3 | No | 54.6 | PrapIR75d | Fig\_all Fig\_topcons  Dumped prediction  deltaG  Topology view | newrun |
| 40 | 366 | 7 | No | 29.1 | PrapOR33 | Fig\_all Fig\_topcons  Dumped prediction  deltaG  Topology view | newrun |
| 41 | 372 | 6 | No | 29.3 | PrapOR34 | Fig\_all Fig\_topcons  Dumped prediction  deltaG  Topology view | newrun |
| 42 | 399 | 7 | No | 36.4 | PrapOR35 | Fig\_all Fig\_topcons  Dumped prediction  deltaG  Topology view | newrun |
| 43 | 419 | 7 | No | 26.7 | PrapOR36 | Fig\_all Fig\_topcons  Dumped prediction  deltaG  Topology view | newrun |
| 44 | 410 | 7 | No | 27.2 | PrapOR37 | Fig\_all Fig\_topcons  Dumped prediction  deltaG  Topology view | newrun |
| 45 | 391 | 6 | No | 31.4 | PrapOR38 | Fig\_all Fig\_topcons  Dumped prediction  deltaG  Topology view | newrun |
| 46 | 390 | 6 | No | 34.3 | PrapOR39 | Fig\_all Fig\_topcons  Dumped prediction  deltaG  Topology view | newrun |
| 47 | 394 | 6 | No | 45.3 | PrapOR11 | Fig\_all Fig\_topcons  Dumped prediction  deltaG  Topology view | newrun |
| 48 | 387 | 6 | No | 37.4 | PrapOR13 | Fig\_all Fig\_topcons  Dumped prediction  deltaG  Topology view | newrun |
| 49 | 204 | 3 | No | 7.4 | PrapGR14 | Fig\_all Fig\_topcons  Dumped prediction  deltaG  Topology view | newrun |
| 50 | 405 | 7 | No | 12.2 | PrapOR26 | Fig\_all Fig\_topcons  Dumped prediction  deltaG  Topology view | newrun |
| 51 | 408 | 7 | No | 12.5 | PrapOR24 | Fig\_all Fig\_topcons  Dumped prediction  deltaG  Topology view | newrun |
| 52 | 397 | 6 | No | 29.2 | PrapOR58 | Fig\_all Fig\_topcons  Dumped prediction  deltaG  Topology view | newrun |
| 53 | 434 | 7 | No | 31.7 | PrapOR59 | Fig\_all Fig\_topcons  Dumped prediction  deltaG  Topology view | newrun |
| 54 | 392 | 7 | No | 30.8 | PrapOR60 | Fig\_all Fig\_topcons  Dumped prediction  deltaG  Topology view | newrun |
| 55 | 411 | 7 | No | 11.8 | PrapOR25 | Fig\_all Fig\_topcons  Dumped prediction  deltaG  Topology view | newrun |
| 56 | 392 | 7 | No | 64.1 | PrapOR15 | Fig\_all Fig\_topcons  Dumped prediction  deltaG  Topology view | newrun |
| 57 | 420 | 7 | No | 11.5 | PrapGR16 | Fig\_all Fig\_topcons  Dumped prediction  deltaG  Topology view | newrun |
| 58 | 391 | 7 | No | 86.3 | PrapOR52 | Fig\_all Fig\_topcons  Dumped prediction  deltaG  Topology view | newrun |
| 59 | 395 | 7 | No | 52.4 | PrapOR16 | Fig\_all Fig\_topcons  Dumped prediction  deltaG  Topology view | newrun |
| 60 | 345 | 8 | No | 376.6 | PrapGR12 | Fig\_all Fig\_topcons  Dumped prediction  deltaG  Topology view | newrun |
| 61 | 392 | 6 | No | 54.8 | PrapOR54 | Fig\_all Fig\_topcons  Dumped prediction  deltaG  Topology view | newrun |
| 62 | 395 | 7 | No | 34.2 | PrapOR61 | Fig\_all Fig\_topcons  Dumped prediction  deltaG  Topology view | newrun |
| 63 | 396 | 7 | No | 34.6 | PrapOR62 | Fig\_all Fig\_topcons  Dumped prediction  deltaG  Topology view | newrun |
| 64 | 394 | 7 | No | 35.6 | PrapOR6 | Fig\_all Fig\_topcons  Dumped prediction  deltaG  Topology view | newrun |
| 65 | 390 | 7 | No | 25.4 | PrapOR17 | Fig\_all Fig\_topcons  Dumped prediction  deltaG  Topology view | newrun |
| 66 | 393 | 6 | No | 74.3 | PrapOR14 | Fig\_all Fig\_topcons  Dumped prediction  deltaG  Topology view | newrun |
| 67 | 380 | 7 | No | 32.3 | PrapOR63 | Fig\_all Fig\_topcons  Dumped prediction  deltaG  Topology view | newrun |
| 68 | 417 | 6 | No | 40.9 | PrapOR55 | Fig\_all Fig\_topcons  Dumped prediction  deltaG  Topology view | newrun |
| 69 | 404 | 7 | No | 28.2 | PrapOR18 | Fig\_all Fig\_topcons  Dumped prediction  deltaG  Topology view | newrun |
| 70 | 407 | 6 | No | 27.1 | PrapOR19 | Fig\_all Fig\_topcons  Dumped prediction  deltaG  Topology view | newrun |
| 71 | 392 | 6 | No | 21.8 | PrapOR20 | Fig\_all Fig\_topcons  Dumped prediction  deltaG  Topology view | newrun |
| 72 | 356 | 8 | No | 345.0 | PrapGR13 | Fig\_all Fig\_topcons  Dumped prediction  deltaG  Topology view | newrun |
| 73 | 387 | 6 | No | 38.5 | PrapOR2 | Fig\_all Fig\_topcons  Dumped prediction  deltaG  Topology view | newrun |
| 74 | 420 | 7 | No | 51.1 | PrapOR56 | Fig\_all Fig\_topcons  Dumped prediction  deltaG  Topology view | newrun |
| 75 | 387 | 6 | No | 34.7 | PrapOR23 | Fig\_all Fig\_topcons  Dumped prediction  deltaG  Topology view | newrun |
| 76 | 397 | 6 | No | 37.7 | PrapOR57 | Fig\_all Fig\_topcons  Dumped prediction  deltaG  Topology view | newrun |
| 77 | 400 | 7 | No | 51.5 | PrapOR9 | Fig\_all Fig\_topcons  Dumped prediction  deltaG  Topology view | newrun |
| 78 | 471 | 7 | No | 38.3 | PrapOrco | Fig\_all Fig\_topcons  Dumped prediction  deltaG  Topology view | newrun |
| 79 | 221 | 4 | No | 304.3 | PrapGR17 | Fig\_all Fig\_topcons  Dumped prediction  deltaG  Topology view | newrun |
| 80 | 346 | 6 | No | 327.0 | PrapGR18 | Fig\_all Fig\_topcons  Dumped prediction  deltaG  Topology view | newrun |
| 81 | 348 | 8 | No | 329.8 | PrapGR15 | Fig\_all Fig\_topcons  Dumped prediction  deltaG  Topology view | newrun |
| 82 | 266 | 5 | No | 677.7 | PrapGR46 | Fig\_all Fig\_topcons  Dumped prediction  deltaG  Topology view | newrun |
| 83 | 361 | 8 | No | 745.8 | PrapGR54 | Fig\_all Fig\_topcons  Dumped prediction  deltaG  Topology view | newrun |
| 84 | 396 | 7 | No | 794.7 | PrapGR44.2 | Fig\_all Fig\_topcons  Dumped prediction  deltaG  Topology view | newrun |
| 85 | 406 | 7 | No | 24.8 | PrapOR27 | Fig\_all Fig\_topcons  Dumped prediction  deltaG  Topology view | newrun |
| 86 | 410 | 7 | No | 24.0 | PrapOR28 | Fig\_all Fig\_topcons  Dumped prediction  deltaG  Topology view | newrun |
| 87 | 338 | 7 | No | 907.6 | PrapGR31 | Fig\_all Fig\_topcons  Dumped prediction  deltaG  Topology view | newrun |
| 88 | 394 | 7 | No | 23.3 | PrapOR29 | Fig\_all Fig\_topcons  Dumped prediction  deltaG  Topology view | newrun |
| 89 | 397 | 7 | No | 877.0 | PrapGR44.1 | Fig\_all Fig\_topcons  Dumped prediction  deltaG  Topology view | newrun |
| 90 | 390 | 7 | No | 22.3 | PrapOR32 | Fig\_all Fig\_topcons  Dumped prediction  deltaG  Topology view | newrun |
| 91 | 347 | 7 | No | 943.7 | PrapGR29 | Fig\_all Fig\_topcons  Dumped prediction  deltaG  Topology view | newrun |
| 92 | 269 | 4 | No | 894.7 | PrapGR42 | Fig\_all Fig\_topcons  Dumped prediction  deltaG  Topology view | newrun |
| 93 | 397 | 7 | No | 21.8 | PrapOR30 | Fig\_all Fig\_topcons  Dumped prediction  deltaG  Topology view | newrun |
| 94 | 405 | 7 | No | 27.1 | PrapOR3 | Fig\_all Fig\_topcons  Dumped prediction  deltaG  Topology view | newrun |
| 95 | 352 | 7 | No | 929.7 | PrapGR30 | Fig\_all Fig\_topcons  Dumped prediction  deltaG  Topology view | newrun |
| 96 | 340 | 7 | No | 890.6 | PrapGR61 | Fig\_all Fig\_topcons  Dumped prediction  deltaG  Topology view | newrun |
| 97 | 399 | 6 | No | 15.9 | PrapGR63 | Fig\_all Fig\_topcons  Dumped prediction  deltaG  Topology view | newrun |
| 98 | 610 | 3 | Yes | 918.5 | PrapIR143 | Fig\_all Fig\_topcons  Dumped prediction  deltaG  Topology view | newrun |
| 99 | 579 | 3 | No | 1070.9 | PrapIR100d | Fig\_all Fig\_topcons  Dumped prediction  deltaG  Topology view | newrun |
| 100 | 395 | 6 | No | 32.1 | PrapOR40 | Fig\_all Fig\_topcons  Dumped prediction  deltaG  Topology view | newrun |
| 101 | 384 | 8 | No | 19.9 | PrapGR68.1 | Fig\_all Fig\_topcons  Dumped prediction  deltaG  Topology view | newrun |
| 102 | 671 | 3 | No | 1075.9 | PrapIR100a | Fig\_all Fig\_topcons  Dumped prediction  deltaG  Topology view | newrun |
| 103 | 377 | 7 | No | 20.0 | PrapGR66 | Fig\_all Fig\_topcons  Dumped prediction  deltaG  Topology view | newrun |
| 104 | 580 | 3 | Yes | 1083.0 | PrapIR100c | Fig\_all Fig\_topcons  Dumped prediction  deltaG  Topology view | newrun |
| 105 | 396 | 6 | No | 29.7 | PrapOR4 | Fig\_all Fig\_topcons  Dumped prediction  deltaG  Topology view | newrun |
| 106 | 651 | 3 | Yes | 1097.7 | PrapIR1.1 | Fig\_all Fig\_topcons  Dumped prediction  deltaG  Topology view | newrun |
| 107 | 392 | 7 | No | 30.4 | PrapOR41 | Fig\_all Fig\_topcons  Dumped prediction  deltaG  Topology view | newrun |
| 108 | 427 | 7 | No | 36.9 | PrapOR43 | Fig\_all Fig\_topcons  Dumped prediction  deltaG  Topology view | newrun |
| 109 | 386 | 7 | No | 27.9 | PrapOR45a | Fig\_all Fig\_topcons  Dumped prediction  deltaG  Topology view | newrun |
| 110 | 417 | 6 | No | 21.3 | PrapOR47 | Fig\_all Fig\_topcons  Dumped prediction  deltaG  Topology view | newrun |
| 111 | 623 | 3 | Yes | 1094.7 | PrapIR1.2 | Fig\_all Fig\_topcons  Dumped prediction  deltaG  Topology view | newrun |
| 112 | 385 | 7 | No | 23.0 | PrapOR45b | Fig\_all Fig\_topcons  Dumped prediction  deltaG  Topology view | newrun |
| 113 | 392 | 7 | No | 33.2 | PrapOR46 | Fig\_all Fig\_topcons  Dumped prediction  deltaG  Topology view | newrun |
| 114 | 389 | 7 | No | 35.6 | PrapOR44 | Fig\_all Fig\_topcons  Dumped prediction  deltaG  Topology view | newrun |
| 115 | 922 | 3 | No | 50.6 | PrapIR25a1 | Fig\_all Fig\_topcons  Dumped prediction  deltaG  Topology view | newrun |
| 116 | 407 | 7 | No | 19.5 | PrapOR5 | Fig\_all Fig\_topcons  Dumped prediction  deltaG  Topology view | newrun |
| 117 | 800 | 3 | Yes | 71.9 | PrapIR21a | Fig\_all Fig\_topcons  Dumped prediction  deltaG  Topology view | newrun |
| 118 | 393 | 7 | No | 54.3 | PrapOR48 | Fig\_all Fig\_topcons  Dumped prediction  deltaG  Topology view | newrun |
| 119 | 394 | 7 | No | 23.0 | PrapOR50 | Fig\_all Fig\_topcons  Dumped prediction  deltaG  Topology view | newrun |
| 120 | 394 | 7 | No | 32.9 | PrapOR49 | Fig\_all Fig\_topcons  Dumped prediction  deltaG  Topology view | newrun |
| 121 | 389 | 7 | No | 33.2 | PrapOR51 | Fig\_all Fig\_topcons  Dumped prediction  deltaG  Topology view | newrun |
| 122 | 400 | 7 | No | 25.1 | PrapOR7 | Fig\_all Fig\_topcons  Dumped prediction  deltaG  Topology view | newrun |
| 123 | 399 | 7 | No | 24.6 | PrapOR8 | Fig\_all Fig\_topcons  Dumped prediction  deltaG  Topology view | newrun |
| 124 | 380 | 1 | Yes | 1217.9 | PrapIR7d.4 | Fig\_all Fig\_topcons  Dumped prediction  deltaG  Topology view | newrun |
| 125 | 633 | 3 | Yes | 1306.2 | PrapIR85a | Fig\_all Fig\_topcons  Dumped prediction  deltaG  Topology view | newrun |
| 126 | 285 | 7 | No | 509.3 | PrapGR64 | Fig\_all Fig\_topcons  Dumped prediction  deltaG  Topology view | newrun |
| 127 | 354 | 7 | No | 509.7 | PrapGR65 | Fig\_all Fig\_topcons  Dumped prediction  deltaG  Topology view | newrun |
| 128 | 400 | 3 | No | 562.3 | PrapIR2 | Fig\_all Fig\_topcons  Dumped prediction  deltaG  Topology view | newrun |
| 129 | 595 | 3 | No | 1479.2 | PrapIR7d.3 | Fig\_all Fig\_topcons  Dumped prediction  deltaG  Topology view | newrun |
| 130 | 596 | 4 | No | 1487.9 | PrapIR7d.2.2 | Fig\_all Fig\_topcons  Dumped prediction  deltaG  Topology view | newrun |
| 131 | 585 | 3 | No | 1667.7 | PrapIR31a | Fig\_all Fig\_topcons  Dumped prediction  deltaG  Topology view | newrun |
| 132 | 583 | 3 | No | 1514.2 | PrapIR7d.2.1 | Fig\_all Fig\_topcons  Dumped prediction  deltaG  Topology view | newrun |
| 133 | 593 | 3 | No | 1521.1 | PrapIR7d.2 | Fig\_all Fig\_topcons  Dumped prediction  deltaG  Topology view | newrun |
